# Cell-surface markers for colon adenoma and adenocarcinoma

**DOI:** 10.18632/oncotarget.7402

**Published:** 2016-02-15

**Authors:** Kamini Sewda, Domenico Coppola, Steven Enkemann, Binglin Yue, Jongphil Kim, Alexis S. Lopez, Jonathan W. Wojtkowiak, Valerie E. Stark, Brian Morse, David Shibata, Shivakumar Vignesh, David L. Morse

**Affiliations:** ^1^ Department of Cancer Imaging and Metabolism, H. Lee Moffitt Cancer Center & Research Institute, Tampa, FL 33612, USA; ^2^ Department of Anatomic Pathology, H. Lee Moffitt Cancer Center & Research Institute, Tampa, FL 33612, USA; ^3^ Department of Molecular Genomics, H. Lee Moffitt Cancer Center & Research Institute, Tampa, FL 33612, USA; ^4^ Department of Biostatistics and Bioinformatics, H. Lee Moffitt Cancer Center & Research Institute, Tampa, FL 33612, USA; ^5^ Department of Tissue Core, H. Lee Moffitt Cancer Center & Research Institute, Tampa, FL 33612, USA; ^6^ Department of Diagnostic Imaging, H. Lee Moffitt Cancer Center & Research Institute, Tampa, FL 33612, USA; ^7^ Department of Surgery, University of Tennessee Health Science Center, Memphis, TN 38163, USA; ^8^ Division of Gastroenterology and Hepatology, SUNY Health Sciences Center at Brooklyn, Brooklyn, NY 11203, USA

**Keywords:** colorectal cancer, cell surface targets, expression profiling, immunohistochemistry, screening

## Abstract

Early detection of colorectal cancer (CRC) is crucial for effective treatment. Among CRC screening techniques, optical colonoscopy is widely considered the gold standard. However, it is a costly and invasive procedure with a low rate of compliance. Our long-term goal is to develop molecular imaging agents for the non-invasive detection of CRC by molecular imaging-based colonoscopy using CT, MRI or fluorescence. To achieve this, cell surface targets must be identified and validated. Here, we report the discovery of cell-surface markers that distinguish CRC from surrounding tissues that could be used as molecular imaging targets. Profiling of mRNA expression microarray data from patient tissues including adenoma, adenocarcinoma, and normal gastrointestinal tissues was used to identify potential CRC specific cell-surface markers. Of the identified markers, six were selected for further validation (*CLDN1*, *GPR56*, *GRM8*, *LY6G6D/F*, *SLCO1B3* and *TLR4*). Protein expression was confirmed by immunohistochemistry of patient tissues. Except for *SLCO1B3*, diffuse and low expression was observed for each marker in normal colon tissues. The three markers with the greatest protein overexpression were CLDN1, LY6G6D/F and TLR4, where at least one of these markers was overexpressed in 97% of the CRC samples. GPR56, LY6G6D/F and SLCO1B3 protein expression was significantly correlated with the proximal tumor location and with expression of mismatch repair genes. Marker expression was further validated in CRC cell lines. Hence, three cell-surface markers were discovered that distinguish CRC from surrounding normal tissues. These markers can be used to develop imaging or therapeutic agents targeted to the luminal surface of CRC.

## INTRODUCTION

Colorectal cancer (CRC) is the third leading cause of cancer related deaths for both men and women in well-developed and industrialized countries [1]. Nearly all CRCs arise from benign colorectal adenomas that can be removed by endoscopic polypectomy, thus preventing the development of malignancy. These small, pre-malignant polyps are typically asymptomatic, thus increasing the need for an effective early detection-screening program to identify patients requiring therapeutic intervention. Effective screening can also detect CRC at earlier stages leading to improved overall survival, i.e. 90% 5 year survival [Colorectal Cancer Facts & Figures 2014-2016, American Cancer Society (ACS)]. Without screening, CRC is often detected at an advanced stage, where treatments are less effective. Patients diagnosed with metastatic CRC have a poor (13%) five-year survival rate (ACS).

Current recommendations for CRC screening emphasize optical endoscopy (i.e. sigmoidoscopy or colonoscopy) [2] or fecal immunochemical test for blood (FIT) [3]. However, there are several problems encountered when endoscopy is used as a first-line screening tool. One issue is the tremendous number of patients who would require screening in the US as the population ages. It is estimated that only ∼50% of individuals that meet the criteria for a screening colonoscopy in the US receive the exam [4]. It is likely that a significant factor in the screening gap is a lack of physicians with the necessary training to perform colonoscopy, primarily gastroenterologists in the US. Another problem with colonoscopy as a screening tool is that the exam must be of high quality to be effective. There are many factors that impact the quality of a colonoscopy: operator skill, bowel preparation prior to the procedure, lesion morphology (e.g. flat lesions are difficult to detect), and patient co-morbidities [5]. Further, colonoscopy provides a stronger correlation in reduced risk of death when lesions are located in the distal colon relative to proximal sites [6]. Proximal lesions are much more likely to have microsatellite instability (MSI), defects in mismatch repair [7] and are endoscopically more challenging because they tend to be flat in appearance, e.g, sessile serrated adenoma [8].

Clearly there is a need to address the challenges that have limited the optimal use of colonoscopy for primary screening of CRC. This has led to the use of non-invasive screening procedures for CRC using diagnostic imaging, either by computed tomography (CT) or magnetic resonance imaging (MRI) [9-11]. The bulk of the work using imaging to screen for CRC has been performed with CT colonography or “virtual colonoscopy,” which has been shown to be effective, with high sensitivity and specificity when compared to optical endoscopy [12, 13]. However, challenges to wide-scale adoption of CT-based screening include: low sensitivity for small or flat lesions, inadequate bowel preparation, lack of experience of interpreting physicians, exam cost and radiation exposure.

A targeted molecular imaging probe that can specifically label cancerous or pre-cancerous lesions in the colon, including flat proximal lesions, could alleviate many of the problems encountered in CRC screening. This probe could be optimized for CT or MRI, increasing the reliability of these techniques detecting small or flat lesions. This could dramatically minimize the impact of interpreter experience. A molecular probe could also be labeled with a fluorescent agent for use during colonoscopy [14-17]. This would make lesions far more conspicuous and improve lesion detection rates, which range from 6 to 27% depending on the study [18-22]. Further, such an approach could also aid in fluorescence guided surgery to improve margin detection [23-27], and could be used for targeted delivery of cytotoxic therapy [28].

Selection of optimal target markers is a known bottleneck in the development of clinically relevant cancer targeted molecular imaging and therapeutic agents. Hence, cancer marker discovery is an area of significant need. This is especially true of cell surface targets, which can lead to more degrees of freedom in chemistry of targeting agents. The goal of the study is to discover markers that are highly expressed on the CRC cell-surface, but are not expressed, or are expressed at relatively low levels on the surrounding non-neoplastic colon tissue. These CRC specific markers could be used to develop targeted molecular imaging probes that specifically deliver CT, MRI or fluorescent contrast agents to colon adenomas and adenocarcinomas but not to the surrounding tissue. Such molecular imaging agents could be used to greatly improve the specificity and sensitivity of detection for both imaging and colonoscopy approaches. The non-neoplastic (normal) colonic epithelial layer is naturally covered by a specific type of mucus throughout the colon and rectum. In the case of colonic adenomas, and adenocarcinomas, this mucus layer is decreased and altered, and is thus less likely to impede delivery [29, 30]. Although targeted imaging probes could be delivered intravenously, oral delivery of agents that are restricted to the gastrointestinal (GI) tract could decrease background signal and off-target effects which could also lead to increased sensitivity and specificity. Additionally, oral agents are easier to deliver and are less likely to have systemic effects.

Gene expression profiling has identified mRNAs that have elevated expression in CRC relative to surrounding normal tissues and unique gene signatures have been identified for different subtypes i.e. epithelial or mesenchymal [31]. Through profiling of mRNA expression microarray data and immunohistochemistry (IHC) of patient tissue samples, we report herein the discovery of cell surface markers that are highly expressed in colon adenomas and adenocarcinomas relative to normal tissues. Marker expression was also confirmed in CRC cell lines as a secondary validation. Hence, these markers may represent valid targets for CRC specific molecular imaging agents.

## RESULTS

### Cell-surface marker identification

**Figure 1:**
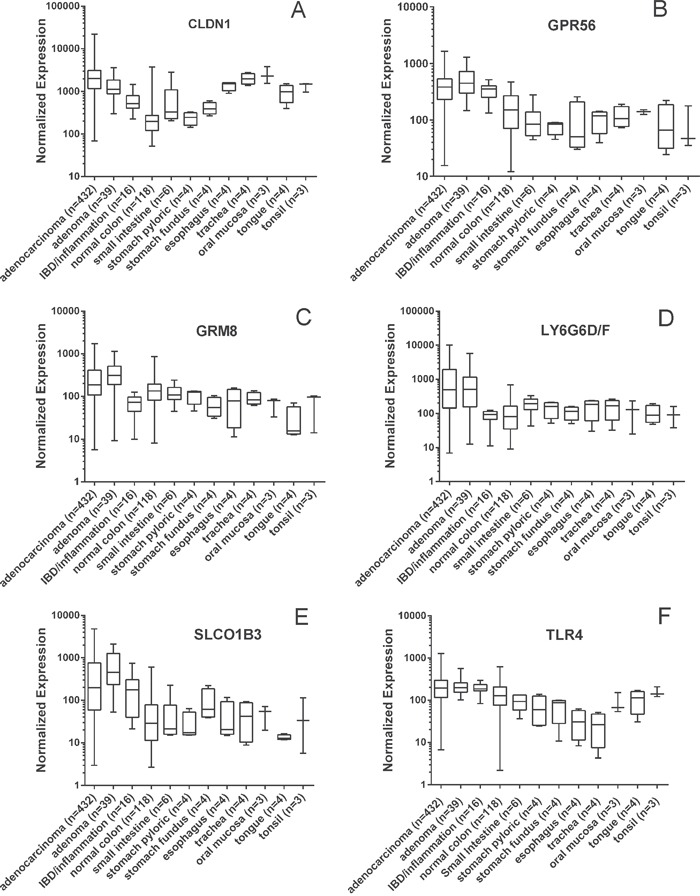
Microarray mRNA expression profiles of CRC cell-surface markers Values are presented as a whiskers/box plot with whiskers representing the full range of values, the box representing 50 percentile and middle line representing the median. Y-axis is log_10_ scale. For all six markers, the adenoma and adenocarcinoma values were significantly higher than the non-neoplastic normal colon values (p<0.01).

Profiling of mRNA expression microarray data from patient tissue samples was used to identify potential markers that could be further validated by confirming protein expression by IHC in patient tissue samples. Putative cell-surface genes (n=42) were identified as having significantly elevated mRNA expression in CRC adenomas and adenocarcinomas relative to non-neoplastic (normal) colon tissues (see Supplemental information, Table S1). Based on known biology relevant to cancer, the availability of known ligands, known structure activity relationships, or the level and breadth of expression in adenomas and adenocarcinomas relative to normal colon, six of the putative cell surface markers were selected for further validation by confirmation of protein expression in patient samples: *CLDN1*, *GPR56*, *GRM8*, *LY6G6D/F*, *SLCO1B1/3/7* and *TLR4*. *LY6G6D* was not distinguished from *LY6G6F* by the Affymetrix probes. *SLCO1B1*, *SLCO1B3* and *SLCO1B7* were not distinguished by the probes but *SLCO1B3* was selected for protein validation due to cancer relevance (see below). Figure 1 shows boxplots of the expression ranges for the six markers. Note the log scale on the Y-axis. In Figure 1A, the median values for *CLDN1* expression in adenocarcinoma (AC) and adenoma are higher than those of the non-neoplastic colon, small intestine and stomach. However, unlike the other markers, expression in oral and throat tissues are also high. For any of the six markers, the individual values in AC cover a broad range from very low to very high, indicating the possibility that no single marker will be highly expressed in every patient. Therefore, a combination of markers may be required to cover the entire range of colon cancer reactivity. Likewise, note that the set of normal colon samples also had a broad range of expression values for each marker, indicating that sometimes normal tissues have high expression that might hinder the resolution of individual markers. Two markers were identified as significantly overexpressed in inflammatory bowel disease (IBD) samples relative to CRC and normal GI tissues (Supplemental information, Figure S1). A number of markers had high expression in both IBD and CRC relative to normal GI tissues, e.g. *CLDN1*, *GPR56* and *TLR4* (Figure 1). These data suggest that in some instances inflammatory conditions may be responsible for the positivity of those samples.

Marker expression was evaluated relative to other tissues of the GI tract. Notably, *GPR56*, *GRM8*, *LY6G6D/F* and *SLCO1B1/3/7* had lower mean expression values in other GI tissues relative to CRC, while *TLR4* had elevated expression in tissues of the mouth and *CLDN1* had elevated expression in tissues of the mouth and esophagus (Figure 1). Expression of tissues and organs involved in systemic toxicity and clearance was also evaluated for the 6 selected markers (Supplemental information, Figure S2). Both *GRM8* and *LY6G6D/F* had low expression among these additional tissues implying that these two markers may be useful for systemic delivery of targeted imaging probes.

### Confirmation of marker protein expression

**Figure 2:**
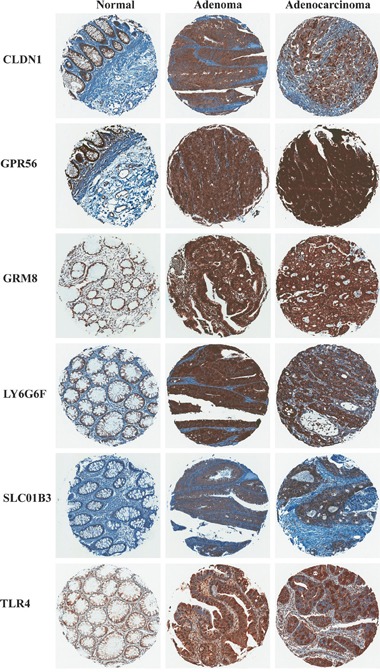
Representative images of marker immunohistochemical staining in patient samples of non-neoplastic normal colon, colon adenoma and adenocarcinoma

To confirm protein expression of the six markers selected above in CRC and normal colon tissues, immunohistochemistry (IHC) was performed for each marker using a tissue microarray containing colon adenoma, adenocarcinoma and normal colon tissue samples from patients (Figure 2). Proteins LY6G6D or LY6G6F were not distinguished from each other by the available antibodies. Table 1 reports the pathologist (A.S.L. and D.C.), scoring for protein expression of each marker.

**Table 1:** Immunohistochemical scoring of marker expression in patient tissue samples

Target	Tissue type	Patient Tissue Samples (n)	Pathology Score							
			0	1	2	3	4	6	9	% ≥ 4
**CLDN1**	**Normal**	**25**	1	1	8	15	0	0	0	0
	**Normal mucinous**^†^	**25**	0	12	13	0	0	0	0	0
	**Adenoma**	**24**	1	0	0	4	0	15	4	79
	**Adenocarcinoma**	**80**	0	0	6	20	8	28	18	68
**GPR56**	**Normal**	**13**	0	0	1	1	0	11	0	85
	**Normal mucinous**^†^	**13**	0	1	5	7	0	0	0	0
	**Adenoma**	**21**	0	0	0	4	0	8	9	81
	**Adenocarcinoma**	**68**	0	0	0	13	0	18	37	81
**GRM8**	**Normal**	**23**	0	0	0	17	0	6	0	26
	**Normal mucinous**^†^	**23**	0	3	11	6	3	0	0	13
	**Adenoma**	**22**	0	0	3	5	0	8	6	63
	**Adenocarcinoma**	**75**	0	0	10	26	4	29	6	52
**LY6G6D/F**	**Normal**	**11**	0	0	0	7	0	4	0	36
	**Normal mucinous**^†^	**11**	0	0	9	3	1	0	0	8
	**Adenoma**	**16**	0	0	0	2	2	6	6	88
	**Adenocarcinoma**	**59**	0	0	9	16	1	20	13	58
**SLCO1B3**	**Normal**	**16**	3	9	2	1	0	1	0	6
	**Normal mucinous**^†^	**16**	2	11	2	0	1	0	0	6
	**Adenoma**	**22**	2	1	8	9	0	2	0	9
	**Adenocarcinoma**	**75**	8	12	34	16	0	5	0	7
**TLR4**	**Normal**	**14**	0	0	11	2	0	0	0	0
	**Normal mucinous**^†^	**14**	0	3	10	0	1	0	0	7
	**Adenoma**	**16**	0	0	2	4	0	8	2	63
	**Adenocarcinoma**	**60**	2	0	16	15	1	20	6	45

† Normal mucinous marker expression is scored in epithelial cells of non-neoplastic (normal) colon tissue while considering heterogeneity of expression on the cell surface due to areas of mucinous secretion that do not express marker.

As described in Materials and Methods, the pathology scores are a multiple of staining intensity and the degree of epithelial cell positivity (i.e. heterogeneity of staining). Hence, scores ≤3 represent samples with either low staining levels, low staining coverage within the sample or both; and scores ≥4 represent samples with at the very least moderate staining levels and coverage. Based on the pathology scores, CLDN1 and TLR4 were the only two marker proteins that effectively distinguished adenomas and adenocarcinomas from normal colon samples by this scoring method. For CLDN1, 79% and 68% of adenoma and adenocarcinoma samples scored ≥4 respectively, and 0% of normal colon samples scored ≥4. Similarly for TLR4, 63% and 45% of adenomas and adenocarcinomas scored ≥4, with only 7% of normal samples scoring ≥4. GRM8 and LY6G6D/F were differentially expressed by this method, but had high percentages of expression in normal colon, i.e. were less able to distinguish CRC from normal tissues, these markers scored 26% and 36% ≥4 in normal colon tissue respectively, compared to 63% and 88% ≥4 for adenomas. GPR56 and SLCO1B3 did not distinguish CRC from normal colon tissue by pathology score alone.

**Figure 3:**
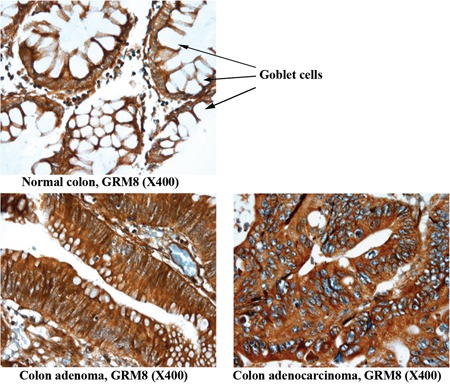
Representative images of non-neoplastic normal colon, colon adenoma and colon adenocarcinoma tissue staining of GRM8 Arrow indicates goblet cells containing mucin in normal colon epithelium. The mucin is digested and lost during the immunohistochemical procedure, leaving the cytoplasm empty and negative for the marker assessed. The conventional colonic adenocarcinoma cells produce less mucin and exhibit a higher IHC score.

The epithelial cell component of normal colon tissue is diffuse, with a high percentage of stromal cells in each sample. Adenomas and adenocarcinomas have a higher epithelial cell density which produces a greater density of staining of all six markers (Figure 2). Additionally, if the plasma membrane surface occupied by mucin secreting vesicles is considered by the pathologist (Figure 3 and Table 1, “normal mucinous”), the homogeneity of marker expression on the cell-surface is decreased and the staining scores for GPR56, GRM8, and LY6G6D/F in non-neoplastic colon tissue samples decreased, allowing better discrimination of CRC from surrounding tissues.

CRC coverage was increased by combining target markers. At least one of the two protein markers, claudin 1 or toll-like receptor 4, scored ≥4 in 83% of adenoma and adenocarcinoma samples in the CRC TMA. At least one of the three markers, CLDN1, TLR4, and LY6G6D/F, were expressed in 97% of the CRC samples. CLDN1, TLR4, and GPR56 together covered 92% of CRC samples based on a ≥4 score.

### Marker expression in proximal vs. distal colon and correlation with mismatch repair

The same TMA used for marker staining was also stained and used to determine pathology scores for the following proteins involved in mismatch repair: MLH1, PMS1, MSH2, PMS2 and MSH6. A subset of the adenocarcinoma tissue cores on the tissue microarray had annotations about the location of the lesion in the colon. Three markers had significantly higher protein expression in the proximal location relative to distal by two sample t-tests or the Satterthwaite test: GPR56, LY6G6D/F and SLCO1B3 (Supplemental Information, Table S2). For GPR56, the average pathology score in distal lesions is 1.2 (95%CI: −2.3, −0.09) units lower than proximal, p=0.034. For LY6G6D/F, the average pathology score in distal lesions is 1.6 (95%CI: −2.9, −0.3) units lower than proximal, p=0.019. For SLCO1B3, the average pathology score in distal lesions is 0.7 (95%CI: −1.3, −0.04) units lower than proximal, p=0.038.

The expression of mismatch repair proteins PMS2 and MSH6 were both positively correlated with GPR56, LY6G6D/F and SLCO1B3 protein expression, with p values ranging from <0.0001 to 0.02 (Supplemental Information, Table S3). Expression of mismatch repair protein PMS1 and LY6G6D/F protein expression were also positively correlated (p=0.01).

### Marker expression in cell lines

**Figure 4:**
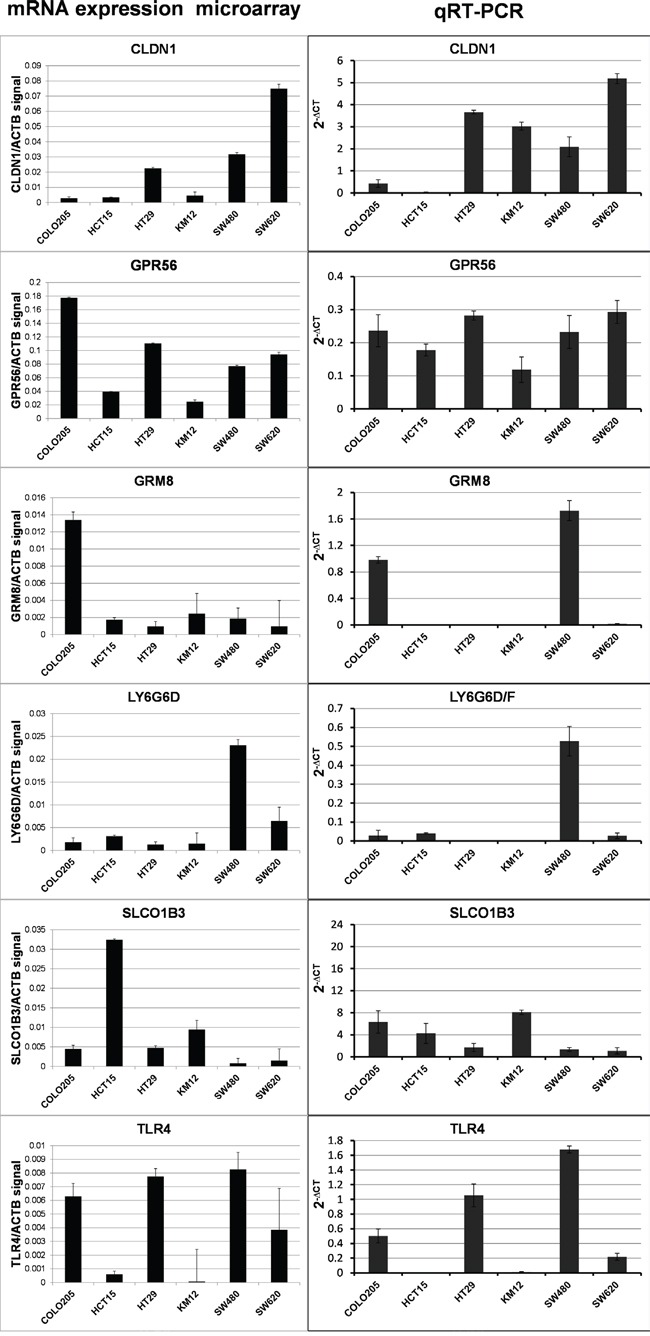
Results for marker mRNA expression in human colorectal cancer cell lines The ratio of normalized microarray signal of each marker/*ACTB* signal (LEFT COLUMN) and qRT-PCR *ACTB* normalized expression (RIGHT COLUMN).

We specifically designed the study using patient tissue samples for marker discovery, since interactions in the tumor microenvironment can alter the cell-surface protein compliment. However, further validation of marker expression in cell lines is both compelling and useful for development of in vivo tumor models for testing of targeted agents during future development. Hence, colon tumor cell lines were characterized for expression of the six markers. Microarray mRNA expression datasets for six different human colon carcinoma cell lines (COLO 205, HCT 15, HT 29, KM12, SW 480 and SW 620) were analyzed for marker mRNA expression. GPR56 expression was observed in all six lines and expression of each marker was observed in at least one of the six tumor lines (Figure 4). For a more quantitative measure of mRNA levels, qRT-PCR was performed to determine expression of each marker in these six lines and ACTB normalized expression values are reported in Figure 4. With a few minor exceptions, the qRT-PCR mRNA expression values were generally in agreement with the microarray mRNA expression values. CLDN1 and GPR56 were both broadly expressed among cell lines, while GRM8, LY6G6D/F, SLCO1B3 and TLR4 were expressed in a few individual lines.

**Figure 5:**
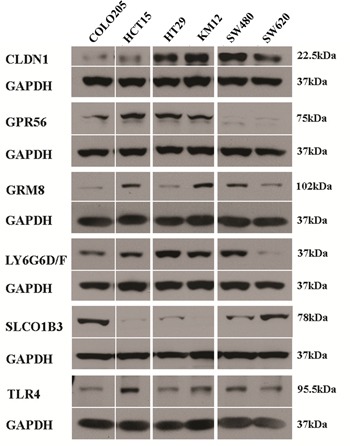
Western blot of marker expression in human CRC tumor cell lines GAPDH was used as loading control for each experiment. Protein expression is shown but gene names are used to conserve space.

Western blots were performed to determine marker protein expression in the six cell lines (Figure 5). Despite the general agreement in levels among the two mRNA expression datasets, the protein expression levels observed in the Western blots were not in agreement with the mRNA levels except for one marker. CLDN1 protein levels were comparable to *CLDN1* mRNA levels, suggesting that regulation of CLDN1 protein occurs at the level of transcription. As described above *GPR56* mRNA was broadly detected among the cell lines, but corresponding protein levels were only detected at high levels in 4 of the six cell lines. Protein expression generally did not correspond to mRNA expression for the remaining markers, i.e. GRM8, LY6G6D/F, SLCO1B3 and TLR4, indicating that protein levels for these markers are likely regulated after translation in this set of colon cancer cells.

**Figure 6:**
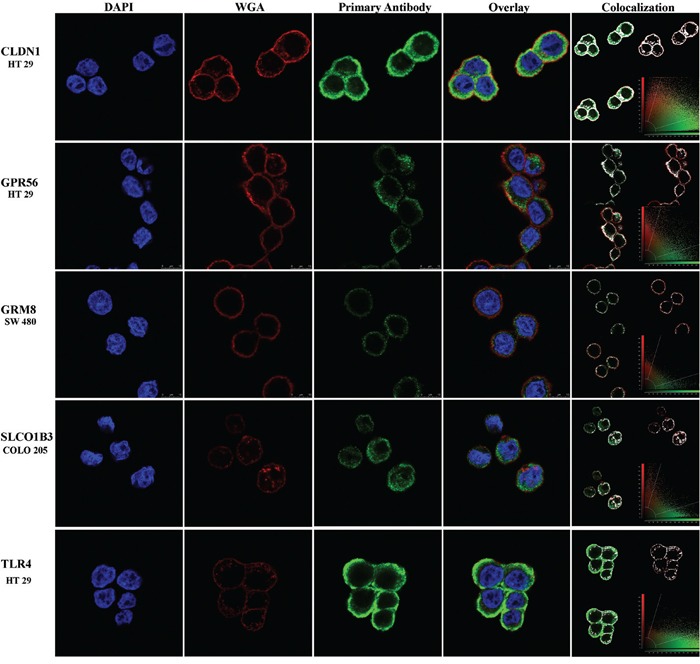
Cell-surface expression of marker proteins in human CRC tumor cell lines by immunocytochemistry Blue, red and green colors represent the DAPI nuclear stain, WGA cell membrane stain and cell-surface marker respectively. The yellow color in the overlay panel shows co-registration of cell membrane and marker expression signals. The colocalization panel shows the range of overlap of cell membrane and marker expression in white for better visualization. Protein expression is shown but gene names are used to conserve space.

To confirm protein expression on the cell-surface, ICC was performed using cells that had high protein levels for each marker and without the use of permeabilization buffer in sample preparation. By ICC, cell surface expression of CLDN1, GPR56 and TLR4 protein was observed in HT-29 cells; GRM8 in SW480 cells; and SLCO1B3 in COLO-205 cells (Figure 6). Surface expression of protein(s) LY6G6D/F was not observed on the surface of any of the cell lines surveyed.

## DISCUSSION

Recent studies of other types of cancer have shown that targeted molecular imaging (CT, PET/SPECT and fluorescence) has great potential and, hence, could be applied to the screening of CRC [32-39]. The goal of this work was to discover cell-surface markers that distinguish CRC from surrounding non-neoplastic (normal) GI tissues. These markers could be used to develop a novel agent that could tag colon adenomas and adenocarcinomas to improve detection with CT, MRI and optical colonoscopy. This could improve the accuracy of virtual colonoscopy using CT and MRI, and perhaps enable the wider use of imaging to screen for CRC. This molecular virtual colonoscopy or “molecular colonography” could be used to identify patients that are in need of standard colonoscopy for biopsy or removal of lesions that have malignant potential and would be anticipated to have lower cost and greater compliance. A targeted fluorescent agent could also improve the accuracy and efficacy of optical fluorescence colonoscopy by enabling easier and more accurate polyp localization.

Our findings identify six cell-surface markers with differentially high mRNA expression in patient samples of CRC compared to normal colon, and high and broad protein expression was observed for these markers among patient samples of colon adenoma and adenocarcinoma as confirmed by IHC. Claudin-1 (CLDN1) is involved in the formation of tight junctions [40] which are altered in colorectal and other cancers [41, 42]. CLDN1 expression and function are altered in cancer [43], including CRC [44-46]. CLDN1 expression is predictive and prognostic in colorectal [47, 48] and other cancers [49, 50]. G-protein-coupled receptor 56 (GPR56) is involved in cell adhesion and extracellular matrix interactions, and has a role in cancer progression [51]; is downregulated as mRNA in HRASV12 transformed Caco-2 CRC cells [52]; and is an orphan receptor with potential as a novel cancer drug discovery target [53]. Lymphocyte antigen 6 complex locus protein G6d, Ly6-D (LY6G6D), is a 133 residue truncated version of the 297 amino acid Ly6-F (LY6G6F). Both are O-glycosylated cell-surface proteins attached to the cell membrane with a glycosylphosphatidylinositol (GPI)-anchor [54-56]. Unlike LY6G6D, LY6G6F crosses the membrane with a single-pass. LY6G6D may be involved in cell-cell interactions and intracellular signal transduction [54-57]. Metabotropic glutamate receptor 8 (GRM8) is a G-protein-coupled receptor and mutations in GRM8 are observed in human cancers [58]. Solute carrier organic anion transporter family, member 1B3 (SLCO1B3) is expressed in a number of hormone-dependent cancer types including sub-types of CRC [59, 60]. Expression of SLCO1B3 in CRC alters p53 dependent pathways and may confer apoptotic resistance [61]. SLCO1B3 is associated with colon cancer and is involved in drug uptake into cancer cells [62-65]. Elevated toll-like receptor 4 (TLR4) expression is associated with a decreased mucus layer, inflammatory bowel disease and CRC progression [66-68]. Although these targets may have potential for use in conventional inhibitory targeted therapy, the rationale for this study is that these markers can be used as “landing pads” for delivery of cytotoxic agents [28] or therapeutic radionuclides [69] for treatment.

Three of the markers: GPR56, LY6G6D/F and SLCO1B3 had significantly higher expression in proximal adenocarcinomas and were positively correlated with mismatch repair protein expression, i.e. PMS2 and MSH6 with all three, and PMS1 with LY6G6D/F. MSH6 is a component of the MutSα (MSH2-MSH6) heterodimer which binds to the dsDNA mismatch and recruits the MutLα mismatch repair endonuclease (MSH1-PMS2) heterodimer [70]. MutLβ (MLH1-PMS1) heterodimer has a minor role in mismatch repair relative to MutLα [71]. Although the three surface markers are known to be involved in cancer-related intracellular signaling pathways, none have yet to be implicated in regulation of mismatch repair. Elevated expression of these mismatch repair proteins would seem to be counterintuitive, as proximal lesions are known to harbor mismatch repair defects.

For each of the six markers, high mRNA was observed in a set of cell lines and mRNA microarray data were largely in agreement with qRT-PCR results. Hence, in the future, the use of mRNA expression array data alone, without qRT-PCR, will likely be sufficient for studies to identify cell lines with likely protein expression of markers. For validation of protein expression in cell lines, Western blot was used to identify a set of cells with high protein expression of each marker. However, with the exception of claudin-1, the mRNA data were not generally in full agreement with the protein expression data in the same cell lines, where some cells with high mRNA had low protein and vice versa. This indicates that for most of these markers, post translational regulation dominates gene expression. By ICC, cell-surface expression was observed for each marker except for LY6G6D/F. However, this doesn't necessarily mean that surface expression will not be observed in CRC, since tumor microenvironmental factors in can also affect gene expression and sub-cellular localization.

Some staining of normal epithelial cells in tissues was observed for all six markers. In the case of CLDN1, staining in normal colon samples has also been reported by Abdelzaher et al. [48]. Four of the marker proteins, CLDN1, GRM8, LY6G6D/F and TLR4, had higher protein expression in CRC compared to the staining observed in epithelial cells of normal colon tissue. However, we remain confident that these markers will be useful for molecular imaging of CRC since epithelial cell density is low in surrounding normal tissues relative to CRC. Staining for these markers in normal tissue was relatively diffuse with a greater stromal component (Figure 2) compared to higher density staining in CRC. Also, normal colon epithelial cells are constantly secreting mucus and non-mucosal marker expression is restricted from a large percentage of the cell surface (Figure 3) [29, 30]. The epithelial layer of normal colon tissue is entirely covered by a thick mucosal layer which protects against pathogenic bacteria and helps in the movement of digested food by peristalsis [29]. This mucus layer is observed to be decreased and altered in areas of cancerous lesions, including adenomas and adenocarcinomas [29, 30]. Hence, the signal from bound imaging probes would be less in non-neoplastic colon tissue compared to the epithelial cells in adenomas and adenocarcinomas, the cytoplasm of which contains a lower amount of mucin. For example, GPR56 was not distinguishable by pathology scoring unless the cytoplasmic surface occupied by mucin is considered, and the discrimination by scoring was greatly improved for GRM8 and LY6G6D/F as these mucus secreting regions do not contain these cell-surface markers. When the percentage of the surface of epithelial cells that express the marker protein was considered in the pathology scoring, five of the six markers had much lower pathology scoring in the normal colon tissue samples compared to CRC. Obviously this would not apply when dealing with mucinous adenocarcinomas, a subtype of colorectal cancer producing a large amount of mucin. None of the tumors studied here were of the mucinous type, and this aspect deserves further investigation. Also, in the group of 44 identified markers (Supplemental information, Table S1) future studies could still identify additional markers with non-expression in normal colon epithelial cells.

None of the markers identified appear to be expressed in all CRC cases. CLDN1, GPR56 and LY6G6D/F had the broadest expression among adenoma samples with 79, 81 and 88% with pathology scores ≥4. Using the IHC score ≥4 as the cutoff for overexpression, these same three markers covered 97% of the CRC samples on the TMA. Since a score of ≥4 is a conservative estimate of expression, it is possible that as few as three markers will be needed to cover nearly all CRC in patients via a targeted imaging approach.

In conclusion, we have identified and confirmed protein expression of six cell-surface markers that are differentially expressed in colon adenomas and adenocarcinomas relative to surrounding normal colon tissue. These markers can potentially be used to develop molecular imaging probes for screening and detection of lesions by fluorescence endoscopy during standard colonoscopy, or by virtual colonoscopy with targeted image contrast, i.e. ‘molecular colonography’. A targeted fluorescent agent may improve the diagnostic utility of colonoscopy and help discriminate between advanced adenomas and adenocarcinomas from hyperplastic polyps and guide clinical decision making in patients with multiple comorbidities and are high risk for invasive procedures [14-17]. Additionally, targeted fluorescent agents may also be used for intraoperative guidance during surgery [23-27]. Although there is potential for development of targeted drugs that modulate the function of these markers, these markers could also serve as “landing pads” for delivery of targeted imaging or therapeutic agents to the cells and not necessarily to target the function of these proteins.

## MATERIALS AND METHODS

### Cell culture

COLO 205, HT 29, HCT 15 and SW 620 human colon cancer cell lines were obtained from the DCTD Tumor Cell Line Repository (NCI at Fredrick, MD), SW 480 cells were obtained from the ATCC (American Type Culture Collection, Manassas, VA) and KM12 cells were obtained from the MD Anderson Cancer Center (Dr. I. Fidler laboratory). Cells were cultured in RPMI-1640 media containing 300mg/L L-Glutamine (Life Technologies, Invitrogen), 10% fetal bovine serum (Atlanta Biologicals), 10,000 units/ml penicillin, and 10,000 μg/ml streptomycin, and were incubated in 5% CO_2_ at 37°C.

Cell lines were authenticated using short tandem repeat (STR) DNA typing according to ATCC's “Authentication of Human Cell Lines: Standardization of STR Profiling” (2012). Genomic DNA was amplified using the Promega GenePrint 10 System (P/N B9510) which targets ten tetrameric repeat loci. Amplicons were resolved by capillary electrophoresis on the Life Technologies - Applied Biosystems 3130XL Genetic Analyzer. The fragments were then analyzed for allele and repeat information using the GeneMarker software (SoftGenetics, State College, PA). Results were compared with STR databases from ATCC and DSMZ to establish percent identity. Cell lines were considered authenticated when the number of shared alleles across the eight core loci is ≥80%, as described by ATCC. Throughout this study, the morphology and growth characteristics of these cells were monitored by microscopy.

### Microarray mRNA expression profiling

The search for potential tumor markers began with an analysis of mRNA levels using gene expression arrays. The Gene Expression Omnibus (GEO) database was searched for microarray data derived from non-neoplastic (normal) colon tissues or diseased tissue including colon tumors. When datasets were found that were generated on Affymetrix U133 plus 2.0 arrays the CEL file data was downloaded, opened in Expression Console (Affymetrix), processed using the MAS 5.0 algorithm to create expression data, and normalized to a trimmed mean value of 500. Quality control metrics were evaluated for each sample and individual samples were removed for poor quality based on overall signal intensity, hybridization quality, RNA quality, and the percentage of probes that detected signal. The final dataset included 432 adenocarcinomas; 39 adenomas; 16 samples of inflamed (including inflammatory bowel disease, IBD) but non-neoplastic colon; non-neoplastic (normal) GI tissues (118 colon, 6 small intestine, 4 stomach, 4 esophagus, 4 trachea, 3 oral mucosa and 3 tonsil) and other normal tissues (4 heart, 8 kidney, 4 liver, 5 lung, 4 lymph node, 8 lymphocyte, 3 skin and 4 spleen). The GEO datasets contributing samples were GSE2109, GSE3526, GSE4107, GSE4183, GSE7307, GSE8671, GSE9254, GSE9452, GSE9686, and an additional 63 samples to be submitted upon publication.

The tumor samples were compared to the normal colon samples for evidence of differential gene expression. Unfortunately, with this many samples even small differences in the distribution of gene expression values can reach statistical significance by T-test. Nonetheless, all probe sets with no significant difference (p > 0.05) in expression were removed from further consideration. Any genes that did not appear to be expressed in any sample and any genes where expression in normal tissues appeared to be higher than in tumor tissues were also removed from consideration. At this point the list was then cross-referenced with a list of 5091 genes that encode membrane associated or secreted proteins, represented by 9763 Affymetrix probesets, that was manually curated using the Gene-Ontology hierarchy (Supplemental information, Table S4). This intersection was to trim the candidate list to markers that might be accessible to targeted agents in vivo. Since the cell surface list contains secreted proteins and proteins anchored on the cytoplasmic side of the plasma membrane, the resulting list was manually curated to include only genes with products that have potential cell-surface expression by database and literature review (e.g. UniProt, PubMed, etc.). As described in the Statistical Methods section below, analyses were then performed on the resulting list of 44 genes.

As another level of validation, Affymetrix expression microarray data generated in the Moffitt Microarray Lab for the 6 cell lines used in this study (vide supra) were analyzed for expression of markers that were confirmed in this study to have protein expression by IHC (vide infra).

### Immunohistochemistry (IHC) of tissue microarray (TMA)

The colon cancer tissue microarray (TMA) was constructed at the Moffitt Tissue Core and contains cores from 46 non-neoplastic (normal) colon samples, 26 colonic adenomas, 91 colonic adenocarcinomas and 26 human colon tumor cell line samples [72]. The formalin-fixed paraffin embedded tissue samples were first examined and categorized after hematoxylin and eosin (H&E) staining as being non-neoplastic colon, colon adenoma, or adenocarcinoma. Primary antibody optimizations were carried out by titrating antibodies at various dilutions on control tissues recommended by the manufacturer (Supporting information, Table S5). Slides were stained using a Ventana Discovery XT automated system (Ventana Medical Systems, Tucson) per the manufacturer's protocol using proprietary reagents. Slides were deparaffinized on the automated system with EZ Prep solution (Ventana). Antigen retrieval methods were used (Ventana). Primary antibodies were diluted using Dako diluent (Carpenteria, CA) at the optimal ratio listed in (Supplemental information, Table S5) and incubated for 32-60 min. The appropriate anti-mouse or anti-rabbit secondary antibody (Ventana) was used for a 16-20 min incubation. The Ventana OmniMap kit detection system was used first and then slides were counterstained with hematoxylin. Following staining, slides were dehydrated and coverslipped. Positive controls were used following the antibody manufacturer recommendations. Negative controls were included by using non-immune mouse or rabbit isotype IgG and omitting the antibodies during the primary antibody incubation step.

Slides were scored by two pathologists (D.C. and A.S.L.) and each sample given a numerical score using the following equation: Score (0-9) = Intensity X Cellularity; where Intensity scores of 0 = negative, 1 = weak, 2 = moderate and 3 = strong staining; and Cellularity scores represent the percentage of epithelial cell staining, with 0 = 0%, 1 = 1-33%, 2 = 34-66% and 3 = 66-100% staining observed throughout the sample. Normal tissues were scored both using the method described above, and also by considering sub-cellular expression levels by accounting for the per cell density of mucin secreting vesicles, which decreases the area available on the cell-surface for marker presentation.

### Quantitative real-time reverse-transcriptase polymerase chain reaction (qRT-PCR)

RNA extractions were performed on cells using the RNeasy^®^Mini Kit (Qiagen) following the manufacturer's instructions. RNA concentration and purity were determined by A_260_/A_280_ ratio using the Nanodrop Spectrophotometer, ND-1000. qRT-PCR was performed using the Smart Cycler (Cephid, Sunnyvale, CA) using a β-actin (ACTB) primer as an internal standard [73]. Primer sets were designed using the http://www.idtdna.com/site for each marker (Supplemental information, Table S6). The QuantiTect SYBR^®^Green RT-PCR Kit (Qiagen) was used for qRT-PCR. During each experiment, reactions were performed using template without the RT step and with no-template added as controls. The following conditions for thermocycling were used: Stage 1 was held at 50°C for 20 min for completion of the RT reaction; stage 2 was held at 95°C for 15 min for initial denaturing of the cDNA; stage 3 cycled 45 times through three temperatures for PCR amplification, starting with 94°C for 15 s, 60°C for 30 s and 72°C for 30 s; and stage 4 included a melt curve for quality control, starting at 60°C and ending at 95°C. Marker expression values were normalized using ACTB expression; Δ C_T_ = Target C_T_- ACTB C_T_. Each experiment was repeated 3 times to determine reproducibility.

### Western blot

Protein was isolated from the colon cancer cell lines cultured in three 75cm^2^ flasks per line at 70% to 80% confluence (∼2×10^6^ cells per flask) by first washing with phosphate buffered saline (DPBS). Then, cell lysates were prepared by incubating for 10 min at RT in insect cell lysis buffer (10 mM Tris pH 7.5, 130 mM NaCl, 1% Triton X-100, 10 mM NaF, 10 M sodium phosphate, 10 mM sodium pyrophosphate) followed by addition of 4X protease inhibitor cocktail (Cat# P2714, Sigma) to 1X final concentration. Lysate was collected by gentle scraping and stored on ice. Lysates were sonicated using an intermediate frequency level for 5 s followed by centrifugation at 4°C for 10 min at 13,000 rpm. Clear lysate supernatant was separated and protein concentration determined using the BCA protein assay (Thermo Scientific) and the Multiskan MCC/340 (Fisher Scientific) plate reader at 570 nm absorbance.

Western blotting was performed as follows: 25μg protein was fractionated by size on SDS/PAGE gels (Invitrogen) and then transferred to nitrocellulose membranes (Bio-Rad Laboratories). The membrane was blocked by incubation in 2% BSA for 1 h, followed by a 2 h incubation with primary antibody (Supplemental information, Table S5), followed by incubation with appropriate secondary antibody conjugated to horseradish peroxidase (ECL Plus Western Blotting Detection System, GE Healthcare Amersham). The antigen-antibody reaction resulted in chemiluminescence which was exposed on X-ray film.

### Immunocytochemistry (ICC)

Colon cancer cells were seeded on glass coverslips in twelve well plates at a density of 2×10^4^ cells per well. Cells attached overnight and were then fixed in fresh 4% paraformaldehyde (USB Corporation) for 20 min at room temperature, followed by 3 washes in DPBS (GIBCO). Fixed cells were rinsed three times for five min with 0.75% glycine in DPBS to quench the paraformaldehyde and blocked with 2% BSA in DPBS for 1 h followed by three 15 min washes. Primary antibodies (Supplemental information, Table S5) were diluted 1:50 in 2% BSA and 5 μg/ml WGA, added to fixed cells and incubated for 2 h followed by three 10 min washes. Secondary antibody (Alexa Fluor^®^ 488, anti-mouse and Alexa Fluor^®^ 488 goat anti-rabbit for monoclonal and polyclonal antibody respectively from Life Technologies, Invitrogen) incubations were performed using 1:2000 dilutions by 2% BSA in DPBS for 1 h, followed by three 10 min washes. The second wash buffer included DAPI (Fluoro Pure™grade, Molecular Probes, Invitrogen) nuclear stain at 1:10,000 dilution. Positive controls were used following the antibody manufacturer recommendations. Negative controls were included by using non-immune mouse sera and omitting the antibodies during the primary antibody incubation step. Coverslips were mounted on glass slides with Prolong^@^Gold-antifade mounting media (Invitrogen) and allowed to sit overnight in the dark at 4°C and then imaged on the confocal microscope (Leica) located in the Moffitt Analytic Microscopy Core.

### Statistical methods

Using expression array datasets, the two-sided multiple comparisons with a control (MCC) method was used identify cell-surface genes with elevated mRNA expression in colorectal adenomas or adenocarcinomas relative to normal colon specimens [74]. Since the goal was to identify cell-surface markers in CRC relative to normal tissues not to determine differences between adenomas and adenocarcinomas, the MCC method is more powerful than all pairwise comparisons. The method developed by Kim generated overall p values and (1-α) ×100% simultaneous confidence intervals for each of the 44 genes. The false discovery method was used to adjust for multiple testings. In addition, the unadjusted 95% simultaneous confidence intervals for each gene were computed to identify which genes are overexpressed in both adenoma and adenocarcinoma. Comparisons of mRNA expression microarray datasets containing patient samples of normal tissues other than colon, IBD or inflammation were performed by t-test. GraphPad Prism, Version 5.04, was used to generate the whiskers/box plots and for presentation of mRNA data. Box plot whiskers represent the minimum to maximum values in the group, the box represents the 50^th^ percentile, and the center line represents the median value. Cell surface proteins and mismatch repair proteins were evaluated for significant differential expression in the distal relative to the proximal lesion location in the colon using the two sample t-test or the Satterthwaite test. Cell surface markers with significant differential expression by location were evaluated for correlation with expression of mismatch repair proteins by calculating the Pearson correlation coefficients. All tests were two-sided and p values of <0.05 were considered statistically significant. Correlation coefficients of >0.05 were considered moderate.

## SUPPLEMENTARY FIGURES AND TABLES






