# Supplementary material for: Cell-surface markers for colon adenoma and adenocarcinoma
Source: Oncotarget. 2016 Feb 15;7(14):17773–89. doi: 10.18632/oncotarget.7402 (PMC4951249; doi:10.18632/oncotarget.7402)
Supplement: Supplementary file 1 [file oncotarget-07-17773-s001.pdf]

Cell-surface markers for colon adenoma and adenocarcinoma

SUPPLEMENTARY FIGURES AND TABLES

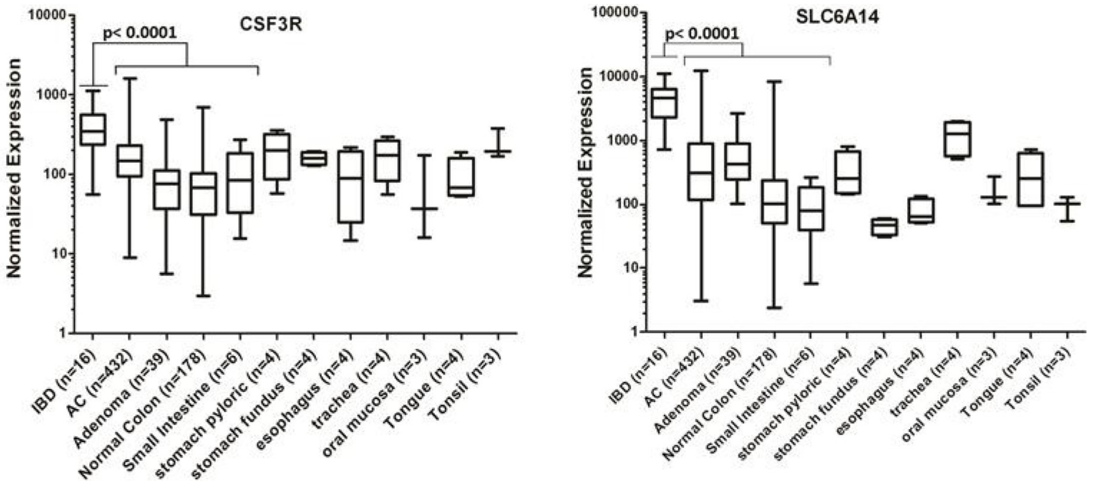

Figure S1: Microarray mRNA expression profiles of IBD cell-surface markers. Values are presented as a whiskers/box plot with whiskers representing the full range of values, the box representing 50 percentile and middle line representing the median. Y-axis is log10 scale.

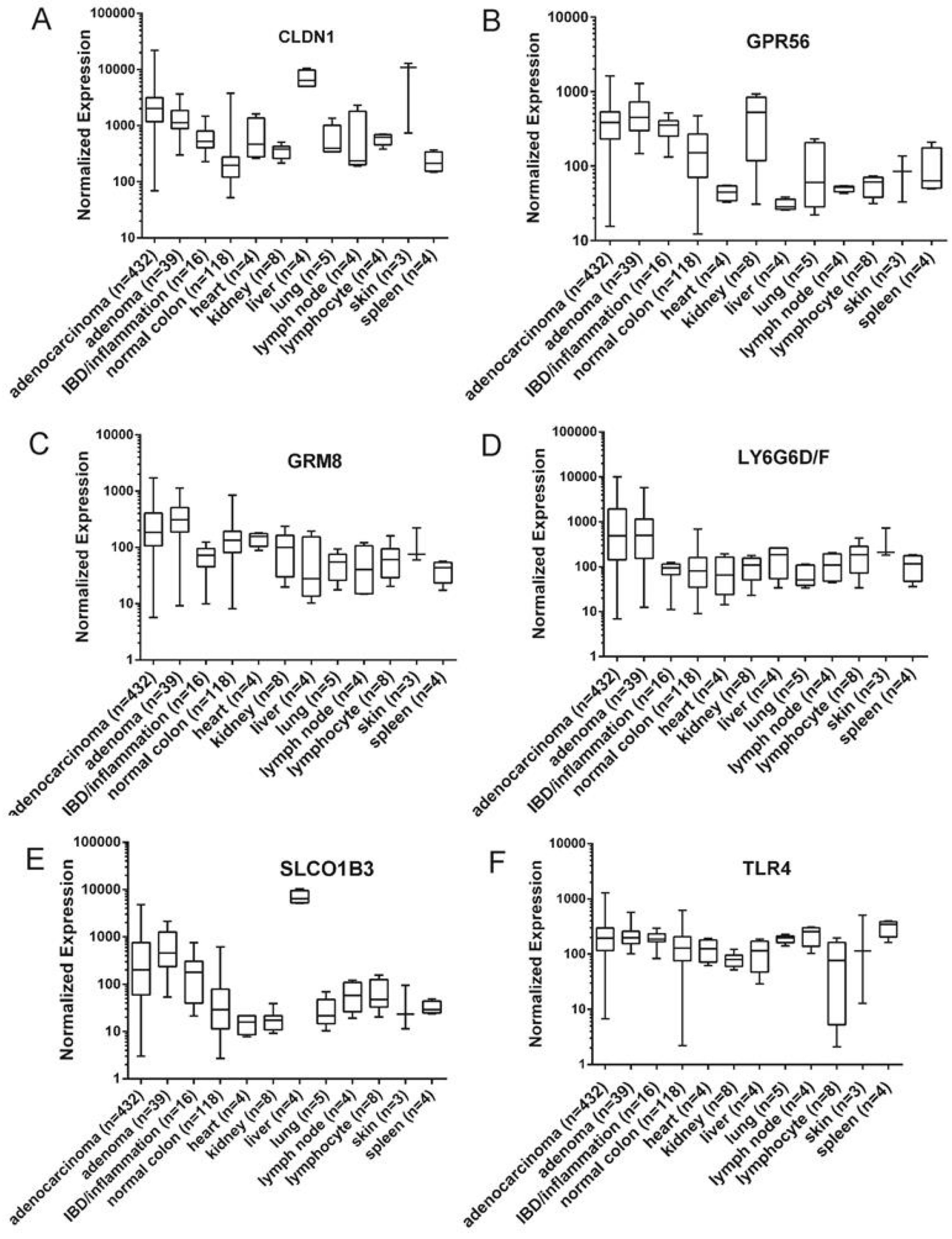

Figure S2: Microarray mRNA expression profiles of cell-surface markers in CRC and in non-neoplastic normal gastrointestinal tissues and tissues involved in systemic toxicity and clearance. Values are presented as a whiskers/box plot with whiskers representing the full range of values, the box representing 50 percentile and middle line representing the median. Y-axis is log10 scale.

Table S1: Normalized mRNA expression in colorectal adenomas, adenocarcinomas and normal colon tissues (sorted by p-value)

See Supplementary File 1

Table S2: Proximal vs. Distal location of marker proteins (two sample t-test using the Satterthwaite method)

Gene/Variable	Location/Class (n)	Mean	LowerCL Mean	UpperCL Mean	StdDev	P value*
CLDN1	distal (42)	5.4048	4.6351	6.1744	2.4699	0.8196
CLDN1	proximal (36)	5.5278	4.7584	6.2972	2.2739	.
GPR56	distal (37)	6.4865	5.6865	7.2865	2.3993	0.0342
GPR56	proximal (30)	7.7000	6.8846	8.5154	2.1838	.
GRM8	distal (40)	4.2750	3.6395	4.9105	1.9870	0.2942
GRM8	proximal (34)	4.7647	4.0719	5.4575	1.9857	.
LY6G6D/F	distal (32)	4.5938	3.6427	5.5448	2.6379	0.0185
LY6G6D/F	proximal (25)	6.1667	5.2376	7.0957	2.2001	.
SLCO1B3	distal (40)	1.7750	1.4636	2.0864	0.9737	0.0375
SLCO1B3	proximal (34)	2.4706	1.8865	3.0547	1.6740	.
TLR4	distal (33)	4.0000	3.1875	4.8125	2.2913	0.2706
TLR4	proximal (25)	4.7200	3.6709	5.7691	2.5417	.
MLH1	Distal (46)	3.7609	2.7706	4.7511	3.3346	0.4992
MLH1	proximal (35)	3.2857	2.2736	4.2978	2.9463	.
PMS1	distal (44)	4.8409	4.0964	5.5855	2.4490	0.5047
PMS1	proximal (34)	5.2059	4.3920	6.0198	2.3326	.
MSH2	distal (47)	5.4468	4.6420	6.2516	2.7411	0.1238
MSH2	proximal (35)	4.6000	3.8492	5.3508	2.1855	.
PMS2	distal (46)	5.6522	5.1201	6.1842	1.7916	0.2912
PMS2	proximal (35)	6.1143	5.4134	6.8152	2.0403	.
MSH6	distal (38)	6.3947	5.4920	7.2975	2.7464	0.0592
MSH6	proximal (33)	7.5455	6.7277	8.3632	2.3061	.

*Rows with significant values are shaded grey.

Table S3: Pearson Correlation Coefficients analysis for marker and mismatch repair proteins

Pearson Correlation Coefficient p value* Number of Observations			
	**GPR56**	**LY6G6D/F**	**SLCO1B3**
**MLH1**	0.17277<br>0.1722<br>64	0.21819<br>0.1202<br>52	0.22770<br>0.0599<br>69
**PMS1**	0.10902<br>0.3912<br>64	0.35373<br>0.0101<br>52	0.08314<br>0.5003<br>68
**MSH2**	0.03401<br>0.7964<br>60	-0.00463<br>0.9740<br>52	-0.00849<br>0.9456<br>67
**PMS2**	0.46501<br>0.0001<br>64	0.53076<br><.0001<br>52	0.25184<br>0.0368<br>69
**MSH6**	0.54341<br><0.0001<br>66	0.43072<br>0.0010<br>55	0.27499<br>0.0212<br>70

*Significant p values are shown in red font.

Table S4: List of cell-surface, plasma-membrane associated and secreted Affymetrix probesets

See Supplementary File 2

Table S5: Antibodies and dilutions for IHC, Western blot and ICC

Antibody	Product	Species	Dilution	Control Tissue	Size kDa
CLDN1	LS Bio C104910, LifeSpan BioSciences, Inc.	Monoclonal Mouse	1:600	Colon (N)	95
TLR4	Mab 14783, R&D Systems	Monoclonal Mouse	1:200	Prostate Cancer	75
LY6G6D/F	HPA008053, Sigma Aldrich	Polyclonal Rabbit	1:20	Normal testis	102
GPR56	TM7XN1/GPR56, Thermo Scientific	Polyclonal Rabbit	1:100	Brain GBM	18
GRM8	Ab53094, Abcam	Polyclonal Rabbit	1:100	Brain	78
SLCO1B3	HPA004943, Sigma Aldrich	Polyclonal Rabbit	1:1250	HepatoCa	37

*Note: LY6G6D is a truncated form of LY6G6F and the antibody used cross-reacts with both isoforms.

Table S6: Primer sets for qRT-PCR

Target Gene	Accession No.	Primer	Sequence (5' to 3')	Product Size (bp)
TLR4	NT_008470.19	forward<br>reverse	tgg cca ttg ctg cca aca tca t<br>tca aag ata cac cag cgg ctc t	113
GPR56		forward<br>reverse	agt tct ggg cct ttg gca ttc a<br>agc aca atg caa ggc aca cag t	
GRM8	AY608335	forward<br>reverse	tgt gtt tca gct atg cag ccc t<br>act tgg gcg ctg tga cag att t	90
CLDN1	NM_021101.4	forward<br>reverse	tgg aaa ggg tgt tgg cat tgg<br>gca gcc aaa tgc ctt gct caa	92
SLC01B3	NM_019844	forward<br>reverse	ttg gct ttg cac tgg gat ctc t<br>aac caa gcc acc aag ctc caa	121
LY6G6D	NM_021246	forward<br>reverse	atc ttg ctc agc tcc ctg ct<br>tca cgg cct ctt tgc aag aac t	97
