# Supplementary material for: Cell-surface markers for colon adenoma and adenocarcinoma
Source: Oncotarget. 2016 Feb 15;7(14):17773–89. doi: 10.18632/oncotarget.7402 (PMC4951249; doi:10.18632/oncotarget.7402)
Supplement: Supplementary file 2 [file oncotarget-07-17773-s002.docx]

**Table S1.** Normalized mRNA expression in colorectal adenomas, adenocarcinomas and normal colon tissues (sorted by p-value).

| **Gene Symbol** | **Mean** | | | ^†^**95% Simultaneous Confidence Interval** | | | | ***p-value** | ^‡^**Significant** |
| --- | --- | --- | --- | --- | --- | --- | --- | --- | --- |
|  | **Normal** | **Adenoma** | **Adeno-carcinoma** | **Adenoma to Normal** | | **Adenocarcinoma to Normal** | |  |  |
|  |  |  |  | **Lower** | **Upper** | **Lower** | **Upper** |  |  |
| *ADAM12* | 79.8 | 64.2 | 785.2 | -278.5 | 309.6 | 540.1 | 870.8 | <1E-17 | Yes |
| *FAP* | 100.4 | 107.5 | 1227.6 | -376.1 | 390.5 | 911.7 | 1342.8 | <1E-17 | Yes |
| *CDH11* | 113.2 | 105.3 | 465.9 | -155.0 | 170.7 | 261.1 | 444.3 | <1E-17 | Yes |
| *BMPR2* | 176.3 | 192.1 | 376.8 | -80.1 | 111.8 | 146.6 | 254.5 | <1E-17 | Yes |
| *DPEP1* | 87.8 | 1307.5 | 1956.1 | 427.6 | 2011.7 | 1422.8 | 2313.7 | <1E-17 | Yes |
| *COL1A1* | 278.6 | 262.9 | 2490.6 | -784.4 | 815.8 | 1762.0 | 2661.9 | <1E-17 | Yes |
| *TNFSF4* | 200.4 | 89.3 | 477.6 | -2.8 | 225.1 | 213.1 | 341.2 | <1E-17 | Yes |
| *PCDHB14* | 130.3 | 221.5 | 418.3 | -5.3 | 187.8 | 233.7 | 342.3 | <1E-17 | Yes |
| *NOX4* | 92.5 | 49.3 | 361.7 | -79.3 | 165.5 | 200.4 | 338.1 | <1E-17 | Yes |
| *PMEPA1* | 287.9 | 248.5 | 797.9 | -201.0 | 279.7 | 374.8 | 645.2 | <1E-17 | Yes |
| *TLR4* | 100.6 | 232.1 | 234.0 | 67.0 | 195.9 | 97.1 | 169.6 | <1E-17 | Yes |
| *MSR1* | 170.3 | 105.0 | 402.6 | -46.1 | 176.7 | 169.7 | 294.9 | <1E-17 | Yes |
| *FZD3* | 146.3 | 232.4 | 449.2 | -42.4 | 214.7 | 230.6 | 375.3 | <1E-17 | Yes |
| *CLEC5A* | 63.7 | 54.8 | 238.9 | -68.4 | 86.3 | 131.7 | 218.6 | <1E-17 | Yes |
| *CLDN1* | 274.7 | 1443.5 | 2469.6 | 380.6 | 1957.0 | 1751.7 | 2638.2 | <1E-17 | Yes |
| *FCGR1A&C* | 150.1 | 129.3 | 557.9 | -126.7 | 168.3 | 324.8 | 490.7 | <1E-17 | Yes |
| *EDNRA* | 131.5 | 86.7 | 641.5 | -180.6 | 270.2 | 383.2 | 636.7 | <1E-17 | Yes |
| *SLC6A6* | 33.2 | 191.7 | 156.9 | 100.0 | 217.0 | 90.8 | 156.6 | <1E-17 | Yes |
| *GPR56* | 173.3 | 520.2 | 409.1 | 251.7 | 442.0 | 182.2 | 289.2 | <1E-17 | Yes |
| *TGFBR1* | 117.9 | 163.7 | 275.5 | -32.1 | 123.7 | 113.8 | 201.4 | 5.55E-16 | Yes |
| *CLDN2* | 139.4 | 1231.5 | 1284.2 | 521.4 | 1662.7 | 823.8 | 1465.6 | 2.00E-15 | Yes |
| *LGR5* | 163.3 | 915.3 | 815.2 | 424.5 | 1079.5 | 467.8 | 836.2 | 6.11E-15 | Yes |
| *XKRX* | 55.5 | 135.5 | 179.9 | 16.5 | 143.5 | 88.7 | 160.1 | 5.48E-14 | Yes |
| *SERPINE1* | 115.3 | 186.4 | 962.2 | -364.0 | 506.2 | 602.2 | 1091.6 | 1.08E-13 | Yes |
| *LY6G6D* | 125.8 | 927.2 | 1250.9 | 217.3 | 1385.4 | 796.6 | 1453.5 | 2.09E-13 | Yes |
| *OLR1* | 101.5 | 69.6 | 427.2 | -137.3 | 201.2 | 230.5 | 420.8 | 2.12E-13 | Yes |
| *TREM1* | 111.1 | 132.2 | 472.9 | -172.3 | 214.5 | 253.0 | 470.6 | 9.74E-13 | Yes |
| *EREG* | 296.4 | 140.4 | 1465.7 | -477.2 | 789.0 | 813.3 | 1525.4 | 1.84E-12 | Yes |
| *TREM2* | 135.0 | 74.4 | 340.1 | -59.3 | 180.5 | 137.7 | 272.6 | 6.53E-11 | Yes |
| *SLCO1B1, 3 & 7* | 66.6 | 732.1 | 580.8 | 360.2 | 970.9 | 342.5 | 685.9 | 1.27E-10 | Yes |
| *FRMD5* | 107.0 | 196.1 | 246.8 | 5.7 | 172.6 | 92.9 | 186.7 | 1.58E-10 | Yes |
| *FN1* | 230.8 | 117.0 | 656.1 | -147.0 | 374.6 | 278.6 | 571.9 | 4.75E-10 | Yes |
| *LY6E* | 254.3 | 229.5 | 979.5 | -472.1 | 521.6 | 445.8 | 1004.6 | 2.55E-08 | Yes |
| *EGFR* | 156.3 | 95.1 | 423.9 | -122.3 | 244.6 | 164.5 | 370.8 | 2.58E-08 | Yes |
| *GRM8* | 154.0 | 378.8 | 295.5 | 118.9 | 330.8 | 82.0 | 201.2 | 3.61E-07 | Yes |
| *PCDHB2* | 73.1 | 64.1 | 167.3 | -63.9 | 81.9 | 53.2 | 135.2 | 8.66E-07 | Yes |
| *GPNMB* | 208.4 | 77.2 | 429.9 | -60.6 | 323.0 | 113.7 | 329.4 | 1.21E-05 | Yes |
| *MME* | 81.7 | 118.1 | 430.0 | -266.5 | 339.2 | 177.9 | 518.5 | 1.32E-05 | Yes |
| *F5* | 171.6 | 146.9 | 603.0 | -425.8 | 475.3 | 177.9 | 684.7 | 3.37E-04 | Yes |
| *PTPRN2* | 153.8 | 325.0 | 238.8 | 51.7 | 290.7 | 17.8 | 152.2 | 0.003 | Yes |
| *MUC17* | 376.4 | 441.2 | 785.8 | -476.3 | 605.9 | 105.1 | 713.7 | 0.006 | Yes |
| *TM4SF4* | 68.4 | 150.1 | 651.4 | -791.4 | 954.7 | 92.0 | 1073.9 | 0.016 | Yes |
| *FGB* | 53.4 | 47.1 | 590.4 | -950.1 | 962.8 | -0.9 | 1074.9 | 0.050 | No |
| *FGA* | 109.1 | 125.2 | 617.1 | -928.1 | 960.3 | -23.0 | 1039.0 | 0.063 | No |

† Unadjusted 95% simultaneous confidence interval.

* Unadjusted p-value for multiple comparisons with a control.

‡ The gene is significant with a 5% false discovery rate (FDR).
