# Supplementary material for: Cell-surface markers for colon adenoma and adenocarcinoma
Source: Oncotarget. 2016 Feb 15;7(14):17773–89. doi: 10.18632/oncotarget.7402 (PMC4951249; doi:10.18632/oncotarget.7402)
Supplement: Supplementary file 3 [file oncotarget-07-17773-s003.docx]

**Supplemental Tables**

**Table S4.** List of cell-surface, plasma-membrane associated and secreted Affymetrix probesets.

| **Gene**  **Symbol** | **Probe ID** | **GenBank ID** | **Entrez**  **Gene ID** | **UniGene ID** |
| --- | --- | --- | --- | --- |
| A1BG | 229819_at | NM_130786.2 | 1 | Hs.529161 |
| A2M | 217757_at | NM_000014.4 | 2 | Hs.212838 |
| A2M | 241206_at | NM_000014 | 2 | Hs.74561 |
| A2M | 1558450_at | NM_000014 | 2 | Hs.74561 |
| A2ML1 | 1553505_at | AK057908.1 | 144568 | Hs.334306 |
| A2ML1 | 1564307_a_at | NM_144670.2 | 144568 | Hs.334306 |
| AADACL2 | 240420_at | NM_207365.1 | 344752 | Hs.100206 |
| ABCA1 | 215869_at | NM_005502 | 19 | Hs.147259 |
| ABCA1 | 215876_at | NM_005502 | 19 | Hs.147259 |
| ABCA1 | 216066_at | NM_005502 | 19 | Hs.429294 |
| ABCA1 | 1570279_at | NM_005502 | 19 | Hs.147259 |
| ABCA1 | 203504_s_at | NM_005502.2 | 19 | Hs.429294 |
| ABCA1 | 203505_at | NM_005502 | 19 | Hs.429294 |
| ABCA10 | 223889_at | NM_080282 | 10349 | Hs.345473 |
| ABCA12 | 215465_at | NM_173076.2 | 26154 | Hs.134585 |
| ABCA13 | 1553295_at | NM_152701 | 154664 | Hs.226568 |
| ABCA13 | 1553604_at | NM_152701.2 | 154664 | Hs.226568 |
| ABCA13 | 1553605_a_at | NM_152701.2 | 154664 | Hs.226568 |
| ABCA13 | 1557297_at | NM_152701 | 154664 | Hs.226568 |
| ABCA2 | 210100_s_at | NM_212533.1 | 20 | Hs.421202 |
| ABCA2 | 212772_s_at | NM_001606.3 | 20 | Hs.421202 |
| ABCA3 | 204343_at | NM_001089.1 | 21 | Hs.26630 |
| ABCA4 | 1569102_at | NM_000350 | 24 | Hs.416707 |
| ABCA4 | 210082_at | NM_000350 | 24 | Hs.416707 |
| ABCA6 | 217504_at | NM_080284.2 | 23460 | Hs.195395 |
| ABCA7 | 219577_s_at | NM_033308.1 | 10347 | Hs.134514 |
| ABCA8 | 1565778_at | NM_007168 | 10351 | Hs.58351 |
| ABCA8 | 1565780_at | NM_007168 | 10351 | Hs.58351 |
| ABCA8 | 204719_at | NM_007168.2 | 10351 | Hs.58351 |
| ABCA9 | 235335_at | NM_080283 | 10350 | Hs.388917 |
| ABCA9 | 239185_at | NM_080283.3 | 10350 | Hs.131686 |
| ABCA9 | 242541_at | NM_080283.3 | 10350 | Hs.131686 |
| ABCB1 | 243951_at |  | 5243 | Hs.21330 |
| ABCB1 | 209993_at | NM_000927.3 | 5243 | Hs.489033 |
| ABCB11 | 208288_at | NM_003742.2 | 8647 | Hs.158316 |
| ABCB11 | 211224_s_at | NM_003742.2 | 8647 | Hs.158316 |
| ABCB4 | 1557641_at | NM_000443 | 5244 | Hs.73812 |
| ABCB4 | 1570505_at | NM_000443 | 5244 | Hs.287827 |
| ABCB4 | 207819_s_at | NM_000443.2 | 5244 | Hs.287827 |
| ABCB4 | 209994_s_at | NM_018850.1 | 5244 | Hs.287827 |
| ABCB5 | 243167_at | BC044248.1 | 340273 | Hs.404102 |
| ABCB5 | 1555371_at | NM_178559.3 | 340273 | Hs.404102 |
| ABCB5 | 1569072_s_at | BC044248.1 | 340273 | Hs.404102 |
| ABCC1 | 202804_at | CR749835.1 | 4363 | Hs.391464 |
| ABCC1 | 202805_s_at | NM_019898.1 | 4363 | Hs.391464 |
| ABCC10 | 213485_s_at | NM_033450.2 | 89845 | Hs.55879 |
| ABCC10 | 215873_x_at | NM_033450.2 | 89845 | Hs.55879 |
| ABCC11 | 224146_s_at | NM_032583.3 | 85320 | Hs.335891 |
| ABCC11 | 1554911_at | NM_032583 | 85320 | Hs.335891 |
| ABCC12 | 1552590_a_at | NM_033226.2 | 94160 | Hs.410111 |
| ABCC12 | 1553410_a_at | NM_033226.2 | 94160 | Hs.410111 |
| ABCC2 | 206155_at | NM_000392.1 | 1244 | Hs.368243 |
| ABCC3 | 214979_at | AK000791.1 | 8714 | Hs.463421 |
| ABCC3 | 230682_x_at | NM_003786 | 8714 | Hs.90786 |
| ABCC3 | 239217_x_at | NM_003786 | 8714 | Hs.463421 |
| ABCC3 | 242553_at | NM_003786 | 8714 | Hs.90786 |
| ABCC3 | 208161_s_at | NM_003786.2 | 8714 | Hs.463421 |
| ABCC3 | 209641_s_at | NM_003786.2 | 8714 | Hs.463421 |
| ABCC4 | 243928_s_at | NM_005845 | 10257 | Hs.307915 |
| ABCC4 | 1554918_a_at | NM_005845.2 | 10257 | Hs.508423 |
| ABCC4 | 1555039_a_at | NM_005845.2 | 10257 | Hs.508423 |
| ABCC4 | 1563279_at |  | 10257 | Hs.508423 |
| ABCC4 | 203196_at | NM_005845.2 | 10257 | Hs.508423 |
| ABCC5 | 226363_at | NM_001023587.1 | 10057 | Hs.368563 |
| ABCC5 | 209380_s_at | NM_005688.2 | 10057 | Hs.368563 |
| ABCC6 | 214033_at | XM_929580.1 | 653770 | Hs.442182 |
| ABCC6 | 215559_at | NM_001171 | 368 | Hs.442182 |
| ABCC6 | 208480_s_at | NM_001171.2 | 368 | Hs.442182 |
| ABCC8 | 210246_s_at | NM_000352.2 | 6833 | Hs.54470 |
| ABCC9 | 1557374_at | NM_005691 | 10060 | Hs.446050 |
| ABCC9 | 208462_s_at | NM_005691.1 | 10060 | Hs.446050 |
| ABCC9 | 208561_at | NM_020297.1 | 10060 | Hs.446050 |
| ABCC9 | 208562_s_at | NM_020297.1 | 10060 | Hs.446050 |
| ABCD2 | 207583_at | NM_005164.2 | 225 | Hs.117852 |
| ABCD4 | 203981_s_at | NM_020323.1 | 5826 | Hs.94395 |
| ABCD4 | 203982_s_at | NM_020326.2 | 5826 | Hs.94395 |
| ABCG1 | 204567_s_at | NM_004915.3 | 9619 | Hs.124649 |
| ABCG1 | 211113_s_at | NM_016818.2 | 9619 | Hs.124649 |
| ABCG2 | 209735_at | NM_004827.2 | 9429 | Hs.480218 |
| ABCG4 | 207593_at | NM_022169.3 | 64137 | Hs.126378 |
| ABCG5 | 220383_at | NM_022436.2 | 64240 | Hs.132992 |
| ABCG8 | 231751_at | NM_022437.2 | 64241 | Hs.413931 |
| ABHD1 | 236461_at | NM_032604 | 84696 | Hs.375791 |
| ABHD1 | 1560117_at | NM_032604 | 84696 | Hs.375791 |
| ABHD14A | 210006_at | NM_015407.3 | 25864 | Hs.534400 |
| ABHD3 | 213017_at | NM_138340.3 | 171586 | Hs.397978 |
| ABHD6 | 221552_at | NM_020676.4 | 57406 | Hs.476454 |
| ABHD6 | 221678_at | NM_020676 | 57406 | Hs.476454 |
| ABHD6 | 221679_s_at | NM_020676.4 | 57406 | Hs.476454 |
| ABHD6 | 45288_at | NM_020676.4 | 57406 | Hs.476454 |
| ABHD7 | 239579_at | NM_173567.2 | 253152 | Hs.201555 |
| ABO | 214504_at | NM_020469.2 | 28 | Hs.495420 |
| ABO | 216716_at | NM_020469 | 28 | Hs.113271 |
| ABO | 216929_x_at | NM_020469.2 | 28 | Hs.495420 |
| ABP1 | 203559_s_at | NM_001091.2 | 26 | Hs.521296 |
| ACBD5 | 225663_at | AB082527.1 | 91452 | Hs.530597 |
| ACBD5 | 1568877_a_at | AF505653.1 | 91452 | Hs.530597 |
| ACCN1 | 206690_at | NM_001094.4 | 40 | Hs.368417 |
| ACCN2 | 37953_s_at | NM_001095.2 | 41 | Hs.274361 |
| ACCN2 | 205156_s_at | NM_001095.2 | 41 | Hs.274361 |
| ACCN3 | 207561_s_at | NM_004769.1 | 9311 | Hs.98547 |
| ACCN4 | 221226_s_at | NM_018674.3 | 55515 | Hs.87469 |
| ACCN5 | 1564504_at | NM_017419.1 | 51802 | Hs.381349 |
| ACE | 227463_at | NM_000789 | 1636 | Hs.298469 |
| ACE | 209749_s_at | NM_152830.1 | 1636 | Hs.298469 |
| ACE2 | 219962_at | NM_021804.1 | 59272 | Hs.178098 |
| ACE2 | 222257_s_at | NM_021804.1 | 59272 | Hs.178098 |
| ACHE | 205377_s_at | NM_000665.3 | 43 | Hs.154495 |
| ACHE | 205378_s_at | NM_000665.3 | 43 | Hs.154495 |
| ACPL2 | 226925_at | NM_001037172.1 | 92370 | Hs.255491 |
| ACPP | 231711_at | NM_001099 | 55 | Hs.388677 |
| ACPP | 237030_at | NM_001099 | 55 | Hs.433060 |
| ACPP | 204393_s_at | NM_001099.2 | 55 | Hs.433060 |
| ACPT | 241715_x_at | NM_080790.1 | 93650 | Hs.293394 |
| ACRBP | 223717_s_at | NM_032489.2 | 84519 | Hs.123239 |
| ACRBP | 223718_at | NM_032489.2 | 84519 | Hs.123239 |
| ACRBP | 230833_at | NM_032489 | 84519 | Hs.123239 |
| ACSL4 | 202422_s_at | NM_004458.1 | 2182 | Hs.268785 |
| ACSL6 | 211207_s_at | NM_001009185.1 | 23305 | Hs.14945 |
| ACVR1 | 203935_at | NM_001105.2 | 90 | Hs.470316 |
| ACVR1B | 205209_at | NM_004302.3 | 91 | Hs.438918 |
| ACVR1B | 208218_s_at | NM_004302.3 | 91 | Hs.438918 |
| ACVR1B | 208219_at | NM_020328.2 | 91 | Hs.438918 |
| ACVR1B | 208222_at | NM_020327.2 | 91 | Hs.438918 |
| ACVR1B | 208223_s_at | NM_004302.3 | 91 | Hs.438918 |
| ACVR1B | 213198_at | NM_004302.3 | 91 | Hs.438918 |
| ACVR1C | 1552519_at | NM_145259.1 | 130399 | Hs.352338 |
| ACVR2A | 228416_at | NM_001616.3 | 92 | Hs.470174 |
| ACVR2A | 205327_s_at | NM_001616.3 | 92 | Hs.470174 |
| ACVR2B | 220028_at | NM_001106.2 | 93 | Hs.517775 |
| ACVRL1 | 210838_s_at | NM_000020.1 | 94 | Hs.410104 |
| ADA | 216705_s_at | NM_000022.2 | 100 | Hs.407135 |
| ADA | 204639_at | NM_000022.2 | 100 | Hs.407135 |
| ADAM10 | 214895_s_at | NM_001110.2 | 102 | Hs.172028 |
| ADAM10 | 202603_at | NM_001110 | 102 | Hs.172028 |
| ADAM10 | 202604_x_at | NM_001110.2 | 102 | Hs.172028 |
| ADAM11 | 239837_at | NM_002390.4 | 4185 | Hs.6088 |
| ADAM11 | 207880_at | NM_002390.4 | 4185 | Hs.6088 |
| ADAM12 | 213790_at | NM_003474 | 8038 | Hs.8850 |
| ADAM12 | 215613_at | NM_003474 | 8038 | Hs.8850 |
| ADAM12 | 226777_at | NM_003474 | 8038 | Hs.8850 |
| ADAM12 | 202952_s_at | NM_003474.3 | 8038 | Hs.386283 |
| ADAM12 | 204943_at | NM_021641.2 | 8038 | Hs.386283 |
| ADAM15 | 217007_s_at | NM_003815.3 | 8751 | Hs.312098 |
| ADAM15 | 1555896_a_at | NM_003815.3 | 8751 | Hs.312098 |
| ADAM17 | 237897_at | NM_003183 | 6868 | Hs.404914 |
| ADAM17 | 205745_x_at | NM_003183.4 | 6868 | Hs.404914 |
| ADAM17 | 205746_s_at | NM_003183.4 | 6868 | Hs.404914 |
| ADAM18 | 1568970_at | NM_014237 | 8749 | Hs.127930 |
| ADAM18 | 207597_at | NM_014237.1 | 8749 | Hs.127930 |
| ADAM19 | 221128_at | NM_023038.3 | 8728 | Hs.483944 |
| ADAM19 | 234208_at | NM_023038 | 8728 | 483944 |
| ADAM19 | 234641_at | NM_023038 | 8728 | 483944 |
| ADAM19 | 209765_at | NM_033274.2 | 8728 | Hs.483944 |
| ADAM2 | 207664_at | NM_001464.3 | 2515 | Hs.177959 |
| ADAM20 | 207422_at | NM_003814 | 8748 | Hs.177984 |
| ADAM20 | 207423_s_at | NM_003814.4 | 8748 | Hs.177984 |
| ADAM21 | 1552579_a_at | NM_003813.2 | 8747 | Hs.178748 |
| ADAM22 | 213411_at | NM_004194 | 53616 | Hs.256398 |
| ADAM22 | 244194_at | NM_004194 | 53616 | Hs.256398 |
| ADAM22 | 1555024_at | NM_004194 | 53616 | Hs.256398 |
| ADAM22 | 206615_s_at | NM_016351.3 | 53616 | Hs.256398 |
| ADAM22 | 206616_s_at | NM_004194.2 | 53616 | Hs.256398 |
| ADAM22 | 208226_x_at | NM_021723.2 | 53616 | Hs.256398 |
| ADAM22 | 208227_x_at | NM_016351.3 | 53616 | Hs.256398 |
| ADAM22 | 208237_x_at | NM_016351.3 | 53616 | Hs.256398 |
| ADAM23 | 244463_at | NM_003812 | 8745 | Hs.432317 |
| ADAM23 | 1559268_at | NM_003812 | 8745 | Hs.432317 |
| ADAM23 | 206046_at | NM_003812.2 | 8745 | Hs.370287 |
| ADAM28 | 241446_at | NM_014265 | 10863 | Hs.174030 |
| ADAM28 | 205997_at | NM_014265 | 10863 | Hs.174030 |
| ADAM28 | 208268_at | NM_014265 | 10863 | Hs.174030 |
| ADAM28 | 208269_s_at | NM_014265 | 10863 | Hs.174030 |
| ADAM29 | 221337_s_at | NM_014269.3 | 11086 | Hs.126838 |
| ADAM30 | 221446_at | NM_021794.2 | 11085 | Hs.283011 |
| ADAM30 | 243520_x_at | NM_021794.2 | 11085 | Hs.283011 |
| ADAM32 | 1552266_at | NM_145004.4 | 203102 | Hs.521545 |
| ADAM33 | 232570_s_at | NM_025220.2 | 80332 | Hs.173716 |
| ADAM33 | 233868_x_at | NM_025220.2 | 80332 | Hs.173716 |
| ADAM7 | 239079_at | AI700468 | 8756 | Hs.116147 |
| ADAM7 | 211238_at | NM_003817.1 | 8756 | Hs.166003 |
| ADAM7 | 211239_s_at | NM_003817.1 | 8756 | Hs.166003 |
| ADAM8 | 205179_s_at | NM_001109.3 | 101 | Hs.501574 |
| ADAM8 | 205180_s_at | NM_001109.3 | 101 | Hs.501574 |
| ADAM9 | 1555326_a_at | NM_003816.2 | 8754 | Hs.2442 |
| ADAM9 | 1570042_a_at | NM_003816 | 8754 | Hs.2442 |
| ADAM9 | 202381_at | NM_003816.2 | 8754 | Hs.2442 |
| ADAMDEC1 | 206134_at | NM_014479.2 | 27299 | Hs.521459 |
| ADAMTS1 | 222162_s_at | NM_006988.3 | 9510 | Hs.534115 |
| ADAMTS1 | 222486_s_at | NM_006988.3 | 9510 | Hs.534115 |
| ADAMTS10 | 230341_x_at | NM_030957.2 | 81794 | Hs.465818 |
| ADAMTS10 | 232133_at | NM_030957.2 | 81794 | Hs.465818 |
| ADAMTS10 | 234490_at | AK024563 | 81794 | 465818 |
| ADAMTS10 | 234574_at | AK024563 | 81794 | 465818 |
| ADAMTS12 | 221421_s_at | NM_030955.2 | 81792 | Hs.481865 |
| ADAMTS13 | 220208_at | NM_139026.2 | 11093 | Hs.131433 |
| ADAMTS13 | 223844_at | NM_139025.2 | 11093 | Hs.131433 |
| ADAMTS14 | 230167_at | NM_080722 | 140766 | Hs.352156 |
| ADAMTS15 | 1553427_at | NM_139055.1 | 170689 | Hs.534221 |
| ADAMTS16 | 238125_at | NM_139056 | 170690 | Hs.387861 |
| ADAMTS17 | 1552725_s_at | NM_139057.1 | 170691 | Hs.513200 |
| ADAMTS18 | 242823_at | NM_139054 | 170692 | Hs.283570 |
| ADAMTS18 | 1553234_at | NM_139054.2 | 170692 | Hs.188746 |
| ADAMTS19 | 1553179_at | NM_133638.2 | 171019 | Hs.23751 |
| ADAMTS19 | 1553180_at | NM_133638.2 | 171019 | Hs.23751 |
| ADAMTS2 | 214454_at | NM_014244.1 | 9509 | Hs.120330 |
| ADAMTS2 | 214535_s_at | NM_021599.1 | 9509 | Hs.120330 |
| ADAMTS2 | 236901_at | NM_014244 | 9509 | Hs.120330 |
| ADAMTS20 | 220717_at | NM_025003 | 80070 | Hs.287554 |
| ADAMTS20 | 1553409_at | NM_025003.2 | 80070 | Hs.287554 |
| ADAMTS3 | 214913_at | NM_014243.1 | 9508 | Hs.27916 |
| ADAMTS4 | 1555380_at | NM_005099 | 9507 | Hs.211604 |
| ADAMTS5 | 219935_at | NM_007038.2 | 11096 | Hs.58324 |
| ADAMTS5 | 229357_at | NM_007038.2 | 11096 | Hs.58324 |
| ADAMTS5 | 235368_at | NM_007038.2 | 11096 | Hs.58324 |
| ADAMTS5 | 1558636_s_at | NM_007038.2 | 11096 | Hs.58324 |
| ADAMTS6 | 220866_at | NM_014273 | 11174 | Hs.482291 |
| ADAMTS6 | 237411_at | NM_197941.2 | 11174 | Hs.482291 |
| ADAMTS6 | 1570351_at | BC020916.1 | 11174 | Hs.558460 |
| ADAMTS7 | 220705_s_at | NM_014272.2 | 11173 | Hs.16441 |
| ADAMTS7 | 220706_at | NM_014272 | 11173 | Hs.16441 |
| ADAMTS7 | 228911_at | NM_014272.2 | 11173 | Hs.559224 |
| ADAMTS8 | 220676_at | NM_007037 | 11095 | Hs.271605 |
| ADAMTS8 | 220677_s_at | NM_007037.3 | 11095 | Hs.271605 |
| ADAMTS8 | 235649_at | NM_007037.3 | 11095 | Hs.271605 |
| ADAMTS9 | 220287_at | NM_020249 | 56999 | Hs.476604 |
| ADAMTS9 | 226814_at | NM_182920.1 | 56999 | Hs.476604 |
| ADAMTS9 | 233785_at | NM_020249 | 56999 | Hs.476604 |
| ADAMTS9 | 233928_at | NM_020249 | 56999 | Hs.318751 |
| ADAMTS9 | 1554697_at | NM_182920.1 | 56999 | Hs.476604 |
| ADAMTSL1 | 224371_at | NM_139238.1 | 92949 | Hs.522019 |
| ADAMTSL1 | 237217_at | NM_052866 | 92949 | Hs.148564 |
| ADAMTSL1 | 1552457_a_at | NM_052866.2 | 92949 | Hs.522019 |
| ADAMTSL1 | 1552808_at | NM_139264.1 | 92949 | Hs.522019 |
| ADAMTSL2 | 206629_at | NM_014694.2 | 9719 | Hs.522543 |
| ADAMTSL3 | 213974_at | NM_207517.1 | 57188 | Hs.459162 |
| ADAMTSL3 | 1559748_at | NM_207517.1 | 57188 | Hs.459162 |
| ADAMTSL4 | 220578_at | AF289612.1 | 54507 | Hs.516243 |
| ADAMTSL4 | 226071_at | NM_019032.4 | 54507 | Hs.516243 |
| ADCK1 | 227482_at | NM_020421.2 | 57143 | Hs.413208 |
| ADCK2 | 221893_s_at | NM_052853.3 | 90956 | Hs.534141 |
| ADCK2 | 221894_at | NM_052853.3 | 90956 | Hs.534141 |
| ADCK2 | 222090_at | NM_052853 | 90956 | Hs.210397 |
| ADCK2 | 222117_s_at | NM_052853 | 90956 | Hs.534141 |
| ADCK2 | 44120_at | NM_052853.3 | 90956 | Hs.534141 |
| ADCK4 | 220434_at | NM_024876 | 79934 | Hs.130712 |
| ADCK4 | 227324_at | NM_024876.2 | 79934 | Hs.130712 |
| ADCK4 | 242609_x_at | AW511797 | 79934 | 130712 |
| ADCK4 | 1556925_at | NM_024876 | 79934 | Hs.130712 |
| ADCK5 | 228524_at | NM_174922.3 | 203054 | Hs.283374 |
| ADCY1 | 213245_at | NM_021116.1 | 107 | Hs.192215 |
| ADCY1 | 215348_at | NM_021116.1 | 107 | Hs.192215 |
| ADCY1 | 232062_at | NM_021116.1 | 107 | Hs.192215 |
| ADCY1 | 235049_at | NM_021116.1 | 107 | Hs.192215 |
| ADCY2 | 217687_at | NM_020546.1 | 108 | Hs.481545 |
| ADCY2 | 217688_at | NM_020546 | 108 | Hs.414591 |
| ADCY2 | 213217_at | NM_020546.1 | 108 | Hs.481545 |
| ADCY2 | 213219_at | NM_020546.1 | 108 | Hs.481545 |
| ADCY3 | 235441_at | NM_004036 | 109 | Hs.188402 |
| ADCY3 | 209320_at | NM_004036.2 | 109 | Hs.467898 |
| ADCY3 | 209321_s_at | NM_004036.2 | 109 | Hs.467898 |
| ADCY4 | 230800_at | NM_139247.2 | 196883 | Hs.443428 |
| ADCY5 | 228182_at | NM_183357 | 111 | Hs.129493 |
| ADCY6 | 209195_s_at | NM_015270.2 | 112 | Hs.525401 |
| ADCY7 | 228289_at | NM_001114 | 113 | Hs.172199 |
| ADCY7 | 238545_at | NM_001114 | 113 | Hs.172199 |
| ADCY7 | 1554827_a_at | NM_001114.2 | 113 | Hs.513578 |
| ADCY7 | 203741_s_at | NM_001114.2 | 113 | Hs.513578 |
| ADCY8 | 206811_at | NM_001115.1 | 114 | Hs.414631 |
| ADCY9 | 215400_x_at | NM_001116 | 115 | Hs.20196 |
| ADCY9 | 204497_at | NM_001116.2 | 115 | Hs.391860 |
| ADCY9 | 204498_s_at | NM_001116.2 | 115 | Hs.391860 |
| ADCYAP1 | 230237_at | NM_001117 | 116 | Hs.68137 |
| ADCYAP1 | 206281_at | NM_001117.2 | 116 | Hs.531719 |
| ADCYAP1R1 | 226690_at | NM_001118 | 117 | Hs.377783 |
| ADCYAP1R1 | 207151_at | NM_001118.3 | 117 | Hs.377783 |
| ADFP | 209122_at | NM_001122.2 | 123 | Hs.3416 |
| ADIPOQ | 207175_at | NM_004797.2 | 9370 | Hs.80485 |
| ADIPOR1 | 217748_at | NM_015999.2 | 51094 | Hs.5298 |
| ADIPOR2 | 201346_at | NM_024551.2 | 79602 | Hs.371642 |
| ADM | 202912_at | NM_001124.1 | 133 | Hs.441047 |
| ADM2 | 220538_at | NM_024866.3 | 79924 | Hs.449099 |
| ADMR | 1552440_at | NM_007264.3 | 11318 | Hs.483909 |
| ADORA1 | 216220_s_at | NM_000674.1 | 134 | Hs.77867 |
| ADORA1 | 205481_at | NM_000674.1 | 134 | Hs.77867 |
| ADORA2A | 1556669_a_at | NM_000675 | 135 | Hs.197029 |
| ADORA2A | 205013_s_at | NM_000675.3 | 135 | Hs.197029 |
| ADORA2B | 205891_at | NM_000676.2 | 136 | Hs.167046 |
| ADORA3 | 223660_at | NM_020683.5 | 140 | Hs.281342 |
| ADORA3 | 206171_at | NM_000677.2 | 140 | Hs.281342 |
| ADPGK | 220980_s_at | NM_031284.3 | 83440 | Hs.513013 |
| ADPGK | 224455_s_at | NM_031284.3 | 83440 | Hs.513013 |
| ADPN | 220675_s_at | NM_025225.2 | 80339 | Hs.377087 |
| ADPN | 233030_at | NM_025225.2 | 80339 | Hs.377087 |
| ADRA1A | 211489_at | NM_033302.1 | 148 | Hs.52931 |
| ADRA1A | 211490_at | NM_033304.1 | 148 | Hs.52931 |
| ADRA1A | 211491_at | NM_033303.2 | 148 | Hs.52931 |
| ADRA1A | 211492_s_at | NM_000680.1 | 148 | Hs.52931 |
| ADRA1B | 207589_at | NM_000679.3 | 147 | Hs.368632 |
| ADRA1D | 210960_at | NM_000678.2 | 146 | Hs.557 |
| ADRA1D | 210961_s_at | NM_000678.2 | 146 | Hs.557 |
| ADRA2A | 209869_at | NM_000681.2 | 150 | Hs.249159 |
| ADRA2B | 208544_at | NM_000682 | 151 | Hs.247686 |
| ADRA2C | 206128_at | NM_000683.3 | 152 | Hs.123022 |
| ADRB1 | 229277_at | NM_000684 | 153 | Hs.99913 |
| ADRB1 | 229309_at | NM_000684 | 153 | Hs.99913 |
| ADRB1 | 208214_at | NM_000684 | 153 | Hs.99913 |
| ADRB2 | 206170_at | NM_000024.3 | 154 | Hs.2551 |
| ADRB3 | 217303_s_at | NM_000025.1 | 155 | Hs.2549 |
| ADRB3 | 206812_at | NM_000025.1 | 155 | Hs.2549 |
| ADRM1 | 201281_at | NM_175573.1 | 11047 | Hs.90107 |
| AEBP1 | 201792_at | NM_001129.3 | 165 | Hs.439463 |
| AFM | 206840_at | NM_001133.2 | 173 | Hs.168718 |
| AFP | 204694_at | NM_001134.1 | 174 | Hs.518808 |
| AGC1 | 1554950_at | NM_001135 | 176 | Hs.2159 |
| AGC1 | 205679_x_at | NM_013227.1 | 176 | Hs.2159 |
| AGC1 | 207692_s_at | NM_001135.1 | 176 | Hs.2159 |
| AGC1 | 217161_x_at | NM_013227.1 | 176 | Hs.2159 |
| AGER | 217046_s_at | NM_001136.3 | 177 | Hs.184 |
| AGER | 210081_at | NM_001136.3 | 177 | Hs.184 |
| AGGF1 | 218534_s_at | NM_018046.3 | 55109 | Hs.213393 |
| AGGF1 | 222661_at | NM_018046.3 | 55109 | Hs.213393 |
| AGGF1 | 208042_at | U84971.1 | 55109 | Hs.213393 |
| AGGF1 | 210710_at | BC002828.2 | 55109 | Hs.213393 |
| AGPAT1 | 215535_s_at | NM_032741.3 | 10554 | Hs.409230 |
| AGPAT1 | 32836_at | NM_032741.3 | 10554 | Hs.409230 |
| AGPAT3 | 219723_x_at | NM_001037553.1 | 56894 | Hs.248785 |
| AGPAT3 | 223182_s_at | NM_001037553.1 | 56894 | Hs.248785 |
| AGPAT3 | 223183_at | NM_001037553.1 | 56894 | Hs.248785 |
| AGPAT3 | 223184_s_at | NM_001037553.1 | 56894 | Hs.248785 |
| AGPAT3 | 224282_s_at | NM_001037553.1 | 56894 | Hs.248785 |
| AGPAT3 | 225440_at | NM_001037553.1 | 56894 | Hs.248785 |
| AGPAT4 | 219693_at | NM_001012733.1 | 56895 | Hs.353175 |
| AGPAT4 | 228667_at | NM_001012733.1 | 56895 | Hs.353175 |
| AGR2 | 228969_at | NM_006408.2 | 10551 | Hs.530009 |
| AGR2 | 209173_at | NM_006408.2 | 10551 | Hs.530009 |
| AGR3 | 228241_at | NM_176813.3 | 155465 | Hs.100686 |
| AGRN | 217410_at | AK021586 | 284602 | Hs.273330 |
| AGRN | 217419_x_at | NM_198576.2 | 375790 | Hs.273330 |
| AGRN | 212283_at | NM_198576.2 | 375790 | Hs.273330 |
| AGRN | 212285_s_at | NM_198576.2 | 375790 | Hs.273330 |
| AGRP | 207193_at | NM_001138.1 | 181 | Hs.104633 |
| AGT | 202834_at | NM_000029.2 | 183 | Hs.19383 |
| AGTR1 | 205357_s_at | NM_000685.3 | 185 | Hs.477887 |
| AGTR1 | 208016_s_at | NM_000685.3 | 185 | Hs.477887 |
| AGTR2 | 222321_at | NM_000686 | 186 | Hs.405348 |
| AGTR2 | 207293_s_at | NM_000686.3 | 186 | Hs.405348 |
| AGTR2 | 207294_at | NM_000686.3 | 186 | Hs.405348 |
| AGTRAP | 225059_at | NM_001040194.1 | 57085 | Hs.464438 |
| AGTRAP | 238135_at | NM_020350 | 57085 | Hs.12854 |
| AGTRAP | 1555736_a_at | NM_001040195.1 | 57085 | Hs.464438 |
| AGTRL1 | 213592_at | NM_005161.3 | 187 | Hs.438311 |
| AHSG | 204551_s_at | NM_001622.1 | 197 | Hs.324746 |
| AHSG | 210929_s_at | NM_001622.1 | 197 | Hs.324746 |
| AIFM3 | 244084_at | NM_144704.1 | 150209 | Hs.163543 |
| AIG1 | 223136_at | NM_016108.2 | 51390 | Hs.478469 |
| AIG1 | 230520_at | BX640703.1 | 51390 | Hs.478469 |
| AIG1 | 232810_at | NM_016108 | 51390 | Hs.478469 |
| AJAP1 | 215789_s_at | AF175409.1 | 55966 | Hs.25924 |
| AJAP1 | 215790_at | BC041648.1 | 55966 | Hs.25924 |
| AJAP1 | 206460_at | AF052143.1 | 55966 | Hs.25924 |
| ALB | 214837_at | NM_000477 | 213 | Hs.418167 |
| ALB | 1565228_s_at | NM_000477.3 | 213 | Hs.418167 |
| ALB | 1568589_at | NM_000477 | 213 | Hs.418167 |
| ALB | 211298_s_at | NM_000477.3 | 213 | Hs.418167 |
| ALCAM | 1569362_at | NM_001627 | 214 | Hs.10247 |
| ALCAM | 201951_at | NM_001627.2 | 214 | Hs.150693 |
| ALCAM | 201952_at | NM_001627.2 | 214 | Hs.150693 |
| ALG1 | 223355_at | NM_019109.3 | 56052 | Hs.406461 |
| ALK | 208211_s_at | NM_004304.3 | 238 | Hs.196534 |
| ALK | 208212_s_at | NM_004304.3 | 238 | Hs.196534 |
| ALKBH5 | 228034_x_at | NM_017758 | 54890 | Hs.356770 |
| ALKBH5 | 234302_s_at | NM_017758.2 | 54890 | Hs.462392 |
| ALKBH5 | 1553101_a_at | NM_017758.2 | 54890 | Hs.462392 |
| ALOX5AP | 204174_at | NM_001629.2 | 241 | Hs.507658 |
| ALPI | 207140_at | NM_001631.2 | 248 | Hs.37009 |
| ALPI | 211618_s_at | NM_001631.2 | 248 | Hs.37009 |
| ALPL | 215783_s_at | NM_000478.2 | 249 | Hs.75431 |
| ALPL | 1557924_s_at | NM_000478.2 | 249 | Hs.75431 |
| ALPP | 204664_at | NM_001632.3 | 250 | Hs.284255 |
| ALPP | 211619_s_at | NM_001632.3 | 250 | Hs.284255 |
| ALPPL2 | 216377_x_at | NM_031313.1 | 251 | Hs.333509 |
| ALPPL2 | 210431_at | NM_031313.1 | 251 | Hs.333509 |
| ALS2CR19 | 1553188_s_at | NM_152526.3 | 117583 | Hs.271903 |
| ALS2CR19 | 1553190_s_at | NM_057177.4 | 117583 | Hs.271903 |
| ALS2CR19 | 1555113_at | NM_057177 | 117583 | Hs.271903 |
| ALS2CR19 | 228411_at | NM_057177 | 117583 | Hs.271903 |
| ALS2CR19 | 239192_at | NM_057177 | 117583 | Hs.26981 |
| ALS2CR4 | 228255_at | NM_152388 | 65062 | Hs.12319 |
| ALS2CR4 | 1553956_at | NM_152388.1 | 65062 | Hs.12319 |
| AMAC1 | 1553471_at | NM_152462.1 | 146861 | Hs.514814 |
| AMAC1L2 | 1553340_s_at | NM_054028.1 | 83650 | Hs.458397 |
| AMBN | 221114_at | NM_016519.4 | 258 | Hs.272396 |
| AMBP | 205477_s_at | NM_001633.2 | 259 | Hs.436911 |
| AMELX | 208410_x_at | NM_001142.2 | 265 | Hs.46329 |
| AMELY | 208220_x_at | NM_001143.1 | 266 | Hs.1238 |
| AMFR | 202203_s_at | NM_001144.4 | 267 | Hs.295137 |
| AMFR | 202204_s_at | NM_001144.4 | 267 | Hs.295137 |
| AMH | 206516_at | NM_000479.2 | 268 | Hs.112432 |
| AMHR2 | 206892_at | NM_020547.1 | 269 | Hs.437877 |
| AMICA1 | 228094_at | NM_153206.1 | 120425 | Hs.16291 |
| AMIGO1 | 226718_at | AB032989.1 | 57463 | Hs.12264 |
| AMIGO2 | 222108_at | NM_181847.2 | 347902 | Hs.121520 |
| AMIGO3 | 229717_at | NM_198722.1 | 29925 | Hs.517970 |
| AMN | 220989_s_at | NM_030943.1 | 81693 | Hs.534494 |
| AMN | 223587_s_at | NM_030943.1 | 81693 | Hs.534494 |
| AMN | 1563792_at | NM_030943 | 81693 | Hs.236720 |
| AMOTL2 | 203002_at | NM_016201.2 | 51421 | Hs.426312 |
| AMY2B | 228023_x_at | NM_020978 | 280 | Hs.558299 |
| AMY2B | 208498_s_at | NM_020978.3 | 280 | Hs.558299 |
| ANG | 205141_at | NM_001145.2 | 283 | Hs.283749 |
| ANGPT1 | 1552939_at | NM_001146 | 284 | Hs.369675 |
| ANGPT1 | 205608_s_at | NM_001146.3 | 284 | Hs.369675 |
| ANGPT1 | 205609_at | NM_001146.3 | 284 | Hs.369675 |
| ANGPT2 | 205572_at | NM_001147.1 | 285 | Hs.553484 |
| ANGPT2 | 211148_s_at | NM_001147.1 | 285 | Hs.553484 |
| ANGPT4 | 221134_at | NM_015985.2 | 51378 | Hs.278973 |
| ANGPTL1 | 224339_s_at | NM_004673.3 | 9068 | Hs.555903 |
| ANGPTL1 | 231773_at | NM_004673.3 | 9068 | Hs.555903 |
| ANGPTL1 | 239183_at | NM_004673.3 | 9068 | Hs.555903 |
| ANGPTL2 | 219514_at | NM_012098.2 | 23452 | Hs.521731 |
| ANGPTL2 | 213001_at | NM_012098.2 | 23452 | Hs.521731 |
| ANGPTL2 | 213004_at | NM_012098.2 | 23452 | Hs.521731 |
| ANGPTL3 | 219803_at | NM_014495.2 | 27329 | Hs.209153 |
| ANGPTL3 | 231684_at | NM_014495 | 27329 | Hs.209153 |
| ANGPTL3 | 243799_x_at | NM_014495 | 27329 | Hs.209153 |
| ANGPTL4 | 221009_s_at | NM_001039667.1 | 51129 | Hs.9613 |
| ANGPTL4 | 223333_s_at | NM_001039667.1 | 51129 | Hs.9613 |
| ANGPTL6 | 223967_at | NM_031917.2 | 83854 | Hs.546452 |
| ANGPTL7 | 206423_at | NM_021146.2 | 10218 | Hs.146559 |
| ANKAR | 1552438_a_at | NM_144708.2 | 150709 | Hs.516651 |
| ANKH | 220076_at | NM_054027 | 56172 | Hs.5062 |
| ANKH | 223092_at | NM_054027.3 | 56172 | Hs.156727 |
| ANKH | 223093_at | NM_054027.3 | 56172 | Hs.156727 |
| ANKH | 223094_s_at | NM_054027.3 | 56172 | Hs.156727 |
| ANKH | 229176_at | NM_054027 | 56172 | Hs.156727 |
| ANKH | 236228_at | NM_054027 | 56172 | Hs.156727 |
| ANKH | 1560369_at | NM_054027 | 56172 | Hs.156727 |
| ANKH | 1560370_x_at | NM_054027 | 56172 | Hs.156727 |
| ANKRD21 | 1553474_at | NM_174981.2 | 317754 | Hs.442712 |
| ANKRD46 | 212731_at | NM_198401.2 | 157567 | Hs.530199 |
| ANPEP | 234458_at | NM_001150 | 290 | Hs.1239 |
| ANPEP | 234576_at | NM_001150 | 290 | Hs.1239 |
| ANPEP | 202888_s_at | NM_001150.1 | 290 | Hs.1239 |
| ANTXR1 | 220092_s_at | NM_018153.2 | 84168 | Hs.165859 |
| ANTXR1 | 220093_at | AK001463.1 | 84168 | Hs.165859 |
| ANTXR1 | 224694_at | NM_032208.1 | 84168 | Hs.165859 |
| ANTXR1 | 227660_at | NM_018153.2 | 84168 | Hs.165859 |
| ANTXR1 | 234832_at | NM_018153 | 84168 | Hs.165859 |
| ANTXR2 | 225524_at | NM_058172.3 | 118429 | Hs.162963 |
| ANTXR2 | 1555536_at | AY040326.1 | 118429 | Hs.162963 |
| ANXA13 | 208323_s_at | NM_004306.2 | 312 | Hs.181107 |
| ANXA2 | 213503_x_at | NM_001002857.1 | 302 | Hs.437110 |
| ANXA2 | 1568126_at | NM_004039 | 302 | Hs.437110 |
| ANXA2 | 201590_x_at | NM_001002857.1 | 302 | Hs.437110 |
| ANXA2 | 210427_x_at | NM_001002857.1 | 302 | Hs.437110 |
| ANXA2P2 | 208816_x_at | M62898.1 | 304 | Hs.534301 |
| ANXA9 | 210085_s_at | NM_003568.1 | 8416 | Hs.430324 |
| ANXA9 | 211712_s_at | NM_003568.1 | 8416 | Hs.430324 |
| AOC3 | 204894_s_at | NM_003734.2 | 8639 | Hs.198241 |
| AP1G1 | 225754_at | NM_001128.5 | 164 | Hs.461253 |
| AP1G1 | 225771_at | NM_001128.5 | 164 | Hs.461253 |
| AP1G1 | 203350_at | NM_001030007.1 | 164 | Hs.461253 |
| AP2M1 | 200613_at | NM_001025205.1 | 1173 | Hs.518460 |
| APBA1 | 206679_at | NM_001163.2 | 320 | Hs.70843 |
| APBA2 | 209870_s_at | NM_005503.2 | 321 | Hs.525718 |
| APBA2 | 209871_s_at | NM_005503.2 | 321 | Hs.525718 |
| APBA3 | 215148_s_at | NM_004886.3 | 9546 | Hs.465607 |
| APBA3 | 205146_x_at | NM_004886.3 | 9546 | Hs.465607 |
| APBB1 | 202652_at | NM_001164.2 | 322 | Hs.372840 |
| APC2 | 217174_s_at | NM_005883.2 | 10297 | Hs.446376 |
| APC2 | 227965_at | NM_005883 | 10297 | Hs.446376 |
| APC2 | 205320_at | NM_005883.2 | 10297 | Hs.446376 |
| APCDD1 | 225016_at | NM_153000.3 | 147495 | Hs.293274 |
| APCDD1L | 235548_at | NM_153360.1 | 164284 | Hs.119286 |
| APCS | 206350_at | NM_001639.2 | 325 | Hs.507080 |
| APH1A | 218389_s_at | NM_016022.1 | 51107 | Hs.108408 |
| APH1A | 1554417_s_at | NM_016022.1 | 51107 | Hs.108408 |
| APH1B | 221036_s_at | NM_031301.2 | 83464 | Hs.511703 |
| APLN | 222856_at | NM_017413.3 | 8862 | Hs.303084 |
| APLP1 | 209462_at | NM_001024807.1 | 333 | Hs.74565 |
| APLP2 | 214875_x_at | NM_001642.1 | 334 | Hs.370247 |
| APLP2 | 228520_s_at | NM_001642 | 334 | Hs.279518 |
| APLP2 | 208248_x_at | NM_001642.1 | 334 | Hs.370247 |
| APLP2 | 208702_x_at | NM_001642.1 | 334 | Hs.370247 |
| APLP2 | 208703_s_at | NM_001642.1 | 334 | Hs.370247 |
| APLP2 | 208704_x_at | NM_001642.1 | 334 | Hs.370247 |
| APLP2 | 211404_s_at | NM_001642.1 | 334 | Hs.370247 |
| APOA1 | 217073_x_at | NM_000039.1 | 335 | Hs.93194 |
| APOA1 | 204450_x_at | NM_000039.1 | 335 | Hs.93194 |
| APOA1BP | 225427_s_at | NM_144772.2 | 128240 | Hs.528320 |
| APOA2 | 219465_at | NM_001643.1 | 336 | Hs.237658 |
| APOA2 | 219466_s_at | NM_001643.1 | 336 | Hs.237658 |
| APOA4 | 206894_at | NM_000482.3 | 337 | Hs.1247 |
| APOA5 | 224243_at | NM_052968.3 | 116519 | Hs.283923 |
| APOA5 | 224244_s_at | NM_052968.3 | 116519 | Hs.283923 |
| APOB | 223579_s_at | AB208846.1 | 338 | Hs.120759 |
| APOB | 205108_s_at | NM_000384 | 338 | Hs.120759 |
| APOC1 | 213553_x_at | NM_001645 | 341 | Hs.268571 |
| APOC1 | 204416_x_at | NM_001645.3 | 341 | Hs.110675 |
| APOC2 | 231561_s_at | NM_000483 | 344 | Hs.75615 |
| APOC2 | 231562_at | NM_000483 | 344 | Hs.75615 |
| APOC2 | 204561_x_at | NM_000483.3 | 344 | Hs.75615 |
| APOC3 | 231587_at | NM_000040 | 345 | Hs.73849 |
| APOC3 | 205820_s_at | NM_000040.1 | 345 | Hs.73849 |
| APOC4 | 206738_at | NM_001646.1 | 346 | Hs.491896 |
| APOC4 | 212884_x_at | NM_001646 | 346 | Hs.110675 |
| APOD | 201525_at | NM_001647.2 | 347 | Hs.522555 |
| APOE | 203381_s_at | NM_000041.2 | 348 | Hs.515465 |
| APOE | 203382_s_at | NM_000041.2 | 348 | Hs.515465 |
| APOF | 207262_at | NM_001638.2 | 319 | Hs.534302 |
| APOH | 231359_at | NM_000042 | 350 | Hs.1252 |
| APOH | 205216_s_at | NM_000042.1 | 350 | Hs.445358 |
| APOL1 | 209546_s_at | NM_145343.1 | 8542 | Hs.114309 |
| APOL2 | 221013_s_at | NM_030882.2 | 23780 | Hs.474740 |
| APOL2 | 221653_x_at | NM_030882.2 | 23780 | Hs.474740 |
| APOL3 | 221087_s_at | NM_030644.1 | 80833 | Hs.474737 |
| APOL4 | 223801_s_at | NM_030643.3 | 80832 | Hs.115099 |
| APOL4 | 224491_at | NM_030643 | 80832 | Hs.115099 |
| APOL4 | 1555600_s_at | NM_030643.3 | 80832 | Hs.115099 |
| APOL5 | 220478_at | NM_030642.1 | 80831 | Hs.326561 |
| APOL6 | 219716_at | NM_030641.3 | 80830 | Hs.257352 |
| APOL6 | 241869_at | NM_030641.3 | 80830 | Hs.257352 |
| APOL6 | 1557116_at | NM_030641.3 | 80830 | Hs.257352 |
| APOL6 | 1557236_at | NM_030641.3 | 80830 | Hs.257352 |
| APOLD1 | 221031_s_at | NM_030817.1 | 81575 | Hs.23388 |
| APOM | 214910_s_at | NM_019101.2 | 55937 | Hs.534468 |
| APOM | 205682_x_at | NM_019101.2 | 55937 | Hs.534468 |
| APP | 214953_s_at | NM_000484.2 | 351 | Hs.406461 |
| APP | 200602_at | NM_000484.2 | 351 | Hs.406461 |
| APP | 211277_x_at | NM_000484 | 351 | Hs.434980 |
| APRG1 | 237368_at | NM_178338 | 339883 | Hs.434887 |
| AQP1 | 207542_s_at | NM_198098.1 | 358 | Hs.76152 |
| AQP1 | 209047_at | NM_198098.1 | 358 | Hs.76152 |
| AQP10 | 1555338_s_at | NM_080429.2 | 89872 | Hs.259048 |
| AQP11 | 229526_at | NM_173039.1 | 282679 | Hs.503345 |
| AQP12A | 1554344_s_at | NM_198998.1 | 375318 | Hs.534650 |
| AQP12B | 1559575_a_at | BC041460.1 | 285192 | Hs.437167 |
| AQP2 | 236630_at | NM_000486.3 | 359 | Hs.130730 |
| AQP2 | 240285_at | NM_000486 | 359 | Hs.130730 |
| AQP2 | 206672_at | NM_000486.3 | 359 | Hs.130730 |
| AQP3 | 39248_at | NM_004925.3 | 360 | Hs.234642 |
| AQP3 | 39249_at | NM_004925.3 | 360 | Hs.234642 |
| AQP3 | 203747_at | NM_004925 | 360 | Hs.234642 |
| AQP4 | 226228_at | NM_001650.4 | 361 | Hs.315369 |
| AQP4 | 210066_s_at | NM_001650.4 | 361 | Hs.315369 |
| AQP4 | 210067_at | NM_001650.4 | 361 | Hs.315369 |
| AQP4 | 210068_s_at | NM_001650.4 | 361 | Hs.315369 |
| AQP4 | 210906_x_at | NM_001650 | 361 | Hs.315369 |
| AQP5 | 213611_at | NM_001651 | 362 | Hs.298023 |
| AQP6 | 216219_at | NM_001652.3 | 363 | Hs.54505 |
| AQP6 | 208435_s_at | NM_001652.3 | 363 | Hs.54505 |
| AQP7 | 206955_at | NM_001170.1 | 364 | Hs.455323 |
| AQP8 | 206784_at | NM_001169.2 | 343 | Hs.176658 |
| AQP9 | 205568_at | NM_020980.2 | 366 | Hs.104624 |
| AREG | 215564_at | NM_001657 | 374 | Hs.270833 |
| AREG | 1557285_at | NM_001657 | 374 | Hs.270833 |
| AREG | 205239_at | NM_001657.2 | 374 | Hs.270833 |
| ARIH2 | 1559532_at | XM_929378.1 | 646450 | Hs.241558 |
| ARL6IP2 | 1553603_s_at | NM_022374.1 | 64225 | Hs.190440 |
| ARL6IP2 | 222700_at | NM_022374 | 64225 | Hs.190440 |
| ARL6IP2 | 235848_x_at | NM_022374 | 64225 | Hs.370800 |
| ARL6IP2 | 237968_at | NM_022374 | 64225 | Hs.370800 |
| ARL6IP6 | 225707_at | NM_152522.2 | 151188 | Hs.516468 |
| ARL6IP6 | 225709_at | AK023109.1 | 151188 | Hs.516468 |
| ARMCX1 | 218694_at | NM_016608.1 | 51309 | Hs.9728 |
| ARMCX2 | 203404_at | NM_177949.1 | 9823 | Hs.48924 |
| ARMCX4 | 1552327_at | NM_152583.2 | 158947 | Hs.546509 |
| ARMCX4 | 1557726_at | BC036206.1 | 158947 | Hs.546509 |
| ARMCX4 | 1564399_a_at | NM_152583.2 | 158947 | Hs.546509 |
| ARMCX4 | 207799_x_at | NM_152583 | 158947 | Hs.399873 |
| ARMCX6 | 214749_s_at | NM_001009584.1 | 54470 | Hs.83530 |
| ARMET | 202655_at | NM_006010.2 | 7873 | Hs.436446 |
| ARMETL1 | 1568696_at | NM_001029954.2 | 441549 | Hs.559067 |
| ARSA | 236541_at | NM_000487 | 410 | Hs.88251 |
| ARSA | 204443_at | NM_000487.3 | 410 | Hs.88251 |
| ARSF | 214490_at | NM_004042.3 | 416 | Hs.101674 |
| ART1 | 1570479_at | NM_004314 | 417 | Hs.382188 |
| ART1 | 1570480_s_at | NM_004314.1 | 417 | Hs.382188 |
| ART1 | 207919_at | NM_004314.1 | 417 | Hs.382188 |
| ART3 | 210147_at | NM_001179 | 419 | Hs.518814 |
| ART5 | 1552524_at | NM_053017.2 | 116969 | Hs.125680 |
| ARTN | 216052_x_at | NM_003976.2 | 9048 | Hs.194689 |
| ARTN | 207675_x_at | NM_003976.2 | 9048 | Hs.194689 |
| ARTN | 210237_at | NM_003976.2 | 9048 | Hs.194689 |
| ARV1 | 223223_at | NM_022786.1 | 64801 | Hs.275736 |
| ARV1 | 238140_at | NM_022786 | 64801 | Hs.438645 |
| ARVCF | 216204_at | NM_001670 | 421 | Hs.326730 |
| ARVCF | 217516_x_at | NM_001670.1 | 421 | Hs.326730 |
| ARVCF | 1556149_at | NM_001670 | 421 | Hs.326730 |
| ARVCF | 205784_x_at | NM_001670.1 | 421 | Hs.326730 |
| ASGR1 | 206743_s_at | NM_001671.2 | 432 | Hs.12056 |
| ASGR2 | 206130_s_at | NM_080912.1 | 433 | Hs.16247 |
| ASH1L | 218554_s_at | NM_018489 | 55870 | Hs.491060 |
| ASH1L | 222667_s_at | NM_018489 | 55870 | Hs.491060 |
| ASH1L | 226447_at | NM_018489 | 55870 | Hs.445445 |
| ASIP | 214498_at | NM_001672.2 | 434 | Hs.432400 |
| ASIP | 1570138_at | NM_001672 | 434 | Hs.361642 |
| ASPN | 219087_at | NM_017680.3 | 54829 | Hs.435655 |
| ASPN | 224396_s_at | NM_017680.3 | 54829 | Hs.435655 |
| ASTN | 213197_at | NM_004319.1 | 460 | Hs.495897 |
| ASTN2 | 215407_s_at | NM_014010.3 | 23245 | Hs.209217 |
| ASTN2 | 1554816_at | NM_014010 | 23245 | Hs.209217 |
| ASTN2 | 209693_at | NM_014010.3 | 23245 | Hs.209217 |
| ASZ1 | 1569729_a_at | NM_130768.1 | 136991 | Hs.352412 |
| ATG4C | 228190_at | AK027773.1 | 84938 | Hs.7353 |
| ATP10A | 214255_at | NM_024490.2 | 57194 | Hs.128041 |
| ATP10A | 214256_at | NM_024490.2 | 57194 | Hs.128041 |
| ATP10A | 1568743_at | NM_024490 | 57194 | Hs.128041 |
| ATP10B | 214070_s_at | AW006935 | 23120 | Hs.109358 |
| ATP10B | 220920_at | AK025130.1 | 23120 | Hs.109358 |
| ATP10D | 213238_at | NM_020453.2 | 57205 | Hs.437241 |
| ATP11A | 213582_at | BF439472 | 23250 | Hs.29189 |
| ATP11A | 215842_s_at | NM_032189.2 | 23250 | Hs.29189 |
| ATP11A | 216488_s_at | NM_015205.1 | 23250 | Hs.29189 |
| ATP11A | 230875_s_at | NM_015205.1 | 23250 | Hs.29189 |
| ATP11A | 241603_at | NM_015205.1 | 23250 | Hs.29189 |
| ATP11A | 241604_at | BE745453 | 23250 | Hs.29189 |
| ATP11B | 238811_at | NM_014616.1 | 23200 | Hs.478429 |
| ATP11B | 1554556_a_at | BC033880 | 23200 | Hs.478429 |
| ATP11B | 1554557_at | BC033880 | 23200 | Hs.478429 |
| ATP11B | 1564063_a_at | AK093727 | 23200 | Hs.478429 |
| ATP11B | 1564064_a_at | NM_014616.1 | 23200 | Hs.478429 |
| ATP11B | 212536_at | NM_014616.1 | 23200 | Hs.478429 |
| ATP11C | 226785_at | NM_001010986.1 | 286410 | Hs.88252 |
| ATP11C | 242690_at | NM_001010986.1 | 286410 | Hs.88252 |
| ATP12A | 207367_at | NM_001676.4 | 479 | Hs.147111 |
| ATP13A1 | 218052_s_at | NM_020410.1 | 57130 | Hs.501794 |
| ATP13A2 | 218608_at | NM_022089.1 | 23400 | Hs.128866 |
| ATP13A3 | 219558_at | XM_942079.1 | 79572 | Hs.529609 |
| ATP13A3 | 212297_at | XM_942079.1 | 79572 | Hs.529609 |
| ATP13A4 | 233535_at | AL512736.1 | 84239 | Hs.450092 |
| ATP13A4 | 1557136_at | NM_032279.2 | 84239 | Hs.561100 |
| ATP13A4 | 1559571_a_at | NM_032279.2 | 84239 | Hs.450092 |
| ATP13A5 | 243585_at | AY358667.1 | 344905 | Hs.531335 |
| ATP1A1 | 220948_s_at | NM_000701.6 | 476 | Hs.371889 |
| ATP1A1 | 236623_at | NM_000701 | 476 | Hs.371889 |
| ATP1A1 | 244606_at | NM_000701 | 476 | Hs.371889 |
| ATP1A2 | 203295_s_at | NM_000702.2 | 477 | Hs.34114 |
| ATP1A2 | 203296_s_at | NM_000702.2 | 477 | Hs.34114 |
| ATP1A3 | 214432_at | NM_152296.3 | 478 | Hs.515427 |
| ATP1A4 | 1558612_a_at | NM_001001734.1 | 480 | Hs.367953 |
| ATP1A4 | 1564241_at | AK098076 | 480 | Hs.367953 |
| ATP1A4 | 1565346_a_at | NM_144699.2 | 480 | Hs.367953 |
| ATP1B1 | 201242_s_at | NM_001677.3 | 481 | Hs.291196 |
| ATP1B1 | 201243_s_at | NM_001677.3 | 481 | Hs.291196 |
| ATP1B2 | 204311_at | NM_001678.3 | 482 | Hs.78854 |
| ATP1B3 | 226570_at | NM_001679 | 483 | Hs.76941 |
| ATP1B3 | 229709_at | NM_001679 | 483 | Hs.76941 |
| ATP1B3 | 208836_at | NM_001679.2 | 483 | Hs.477789 |
| ATP1B4 | 220556_at | NM_012069.2 | 23439 | Hs.292599 |
| ATP1B4 | 243737_at | NM_012069 | 23439 | Hs.292599 |
| ATP2A2 | 239996_x_at | NM_001681 | 488 | Hs.374535 |
| ATP2A2 | 209186_at | NM_170665.2 | 488 | Hs.506759 |
| ATP2A2 | 212361_s_at | NM_001681.2 | 488 | Hs.506759 |
| ATP2A2 | 212362_at | NM_001681 | 488 | Hs.374535 |
| ATP2A3 | 230453_s_at | NM_005173 | 489 | Hs.5541 |
| ATP2A3 | 207521_s_at | NM_005173.2 | 489 | Hs.513870 |
| ATP2A3 | 207522_s_at | NM_005173.2 | 489 | Hs.513870 |
| ATP2A3 | 213036_x_at | NM_005173.2 | 489 | Hs.513870 |
| ATP2A3 | 213042_s_at | NM_005173.2 | 489 | Hs.513870 |
| ATP2B1 | 215716_s_at | NM_001001323.1 | 490 | Hs.506276 |
| ATP2B1 | 209281_s_at | NM_001001323.1 | 490 | Hs.506276 |
| ATP2B1 | 212930_at | NM_001682.2 | 490 | Hs.506276 |
| ATP2B2 | 216120_s_at | NM_001001331.1 | 491 | Hs.268942 |
| ATP2B2 | 234714_x_at | NM_001683 | 491 | Hs.268942 |
| ATP2B2 | 234741_at | NM_001683 | 491 | Hs.89512 |
| ATP2B2 | 204685_s_at | NM_001001331.1 | 491 | Hs.268942 |
| ATP2B2 | 211586_s_at | NM_001001331.1 | 491 | Hs.268942 |
| ATP2B3 | 215911_x_at | NM_021949 | 492 | Hs.533956 |
| ATP2B3 | 216312_at | NM_021949 | 492 | Hs.103124 |
| ATP2B3 | 242036_x_at | NM_001001344.1 | 492 | Hs.533956 |
| ATP2B3 | 207026_s_at | NM_001001344.1 | 492 | Hs.533956 |
| ATP2B4 | 205410_s_at | NM_001001396.1 | 493 | Hs.343522 |
| ATP2B4 | 210711_at | XR_001001.1 | 84719 | Hs.343522 |
| ATP2B4 | 212135_s_at | NM_001001396.1 | 493 | Hs.343522 |
| ATP2B4 | 212136_at | NM_001001396.1 | 493 | Hs.343522 |
| ATP2C2 | 206043_s_at | NM_014861.1 | 9914 | Hs.6168 |
| ATP4A | 207139_at | NM_000704.2 | 495 | Hs.36992 |
| ATP4B | 1563405_at | NM_000705 | 496 | Hs.434202 |
| ATP4B | 1563407_x_at | NM_000705 | 496 | Hs.434202 |
| ATP4B | 207546_at | NM_000705.2 | 496 | Hs.434202 |
| ATP5A1 | 213738_s_at | NM_001001937.1 | 498 | Hs.298280 |
| ATP5A1 | 1569891_at | NM_004046 | 498 | Hs.298280 |
| ATP5B | 201322_at | NM_001686.3 | 506 | Hs.406510 |
| ATP5O | 216954_x_at | NM_001697 | 539 | Hs.409140 |
| ATP5O | 1564482_at | NM_001697 | 539 | Hs.409140 |
| ATP5O | 200818_at | NM_001697.2 | 539 | Hs.409140 |
| ATP6AP2 | 201443_s_at | NM_005765.2 | 10159 | Hs.495960 |
| ATP6AP2 | 201444_s_at | NM_005765.2 | 10159 | Hs.495960 |
| ATP6V0A1 | 1559374_at | NM_005177 | 535 | Hs.267871 |
| ATP6V0A1 | 1559375_s_at | NM_005177 | 535 | Hs.267871 |
| ATP6V0A1 | 205095_s_at | NM_005177.3 | 535 | Hs.463074 |
| ATP6V0A1 | 212383_at | NM_005177.3 | 535 | Hs.463074 |
| ATP6V0A2 | 217603_at | NM_012463 | 23545 | Hs.201939 |
| ATP6V0A2 | 235255_at | NM_012463 | 23545 | Hs.201939 |
| ATP6V0A2 | 1555114_at | NM_012463 | 23545 | Hs.201939 |
| ATP6V0A2 | 205704_s_at | NM_012463.2 | 23545 | Hs.201939 |
| ATP6V0A4 | 220197_at | NM_130840.2 | 50617 | Hs.98967 |
| ATP6V1A | 223964_x_at | NM_001690 | 523 | Hs.409131 |
| ATP6V1A | 201971_s_at | NM_001690.2 | 523 | Hs.477155 |
| ATP6V1A | 201972_at | NM_001690.2 | 523 | Hs.477155 |
| ATP7A | 205197_s_at | NM_000052.2 | 538 | Hs.496414 |
| ATP7A | 205198_s_at | NM_000052 | 538 | Hs.496414 |
| ATP7B | 204624_at | NM_001005918.1 | 540 | Hs.492280 |
| ATP8A1 | 231484_at | NM_006095 | 10396 | Hs.291385 |
| ATP8A1 | 1569773_at | NM_006095 | 10396 | Hs.435052 |
| ATP8A1 | 210192_at | NM_006095.1 | 10396 | Hs.435052 |
| ATP8A1 | 213106_at | NM_006095 | 10396 | Hs.435052 |
| ATP8A2 | 219659_at | NM_016529 | 51761 | Hs.444957 |
| ATP8A2 | 219660_s_at | NM_016529.3 | 51761 | Hs.444957 |
| ATP8A2 | 231395_at | NM_016529 | 51761 | Hs.130489 |
| ATP8B1 | 214594_x_at | NM_005603 | 5205 | Hs.418426 |
| ATP8B1 | 226302_at | NM_005603 | 5205 | Hs.418426 |
| ATP8B1 | 238055_at | NM_005603 | 5205 | Hs.418426 |
| ATP8B2 | 216873_s_at | NM_020452.2 | 57198 | Hs.435700 |
| ATP8B2 | 226771_at | NM_020452.2 | 57198 | Hs.435700 |
| ATP8B3 | 215319_at | NM_138813.2 | 148229 | Hs.306212 |
| ATP8B3 | 239457_at | NM_138813.2 | 148229 | Hs.306212 |
| ATP8B3 | 1554704_at | BC033179 | 148229 | Hs.306212 |
| ATP8B4 | 220416_at | NM_024837.2 | 79895 | Hs.511311 |
| ATP9A | 216129_at | AL117659 | 10079 | Hs.406434 |
| ATP9A | 212062_at | NM_006045.1 | 10079 | Hs.368002 |
| ATP9B | 214010_s_at | NM_198531.3 | 374868 | Hs.465475 |
| ATP9B | 214934_at | NM_198531.3 | 374868 | Hs.465475 |
| ATP9B | 236762_at | BE818251 | 11071 | Hs.321101 |
| ATP9B | 237766_at | NM_198531.3 | 374868 | Hs.465475 |
| ATP9B | 1564344_at | AK097757.1 | 374868 | Hs.465475 |
| ATP9B | 1568723_at | BC022535.1 | 374868 | Hs.465475 |
| ATPIF1 | 218671_s_at | NM_178190.1 | 93974 | Hs.241336 |
| ATPIF1 | 223338_s_at | NM_178191.1 | 93974 | Hs.241336 |
| ATPIF1 | 223339_at | NM_178191.1 | 93974 | Hs.241336 |
| ATPIF1 | 1557383_a_at | NM_016311 | 93974 | Hs.241336 |
| ATRN | 211852_s_at | NM_139322.1 | 8455 | Hs.276252 |
| ATRN | 212517_at | NM_139321.1 | 8455 | Hs.276252 |
| ATRNL1 | 213744_at | AB011106.2 | 26033 | Hs.501127 |
| ATRNL1 | 213745_at | AB011106.2 | 26033 | Hs.501127 |
| ATRNL1 | 1569796_s_at | AY442317.1 | 26033 | Hs.501127 |
| AVP | 207848_at | NM_000490.3 | 551 | Hs.89648 |
| AVPR1A | 206250_x_at | NM_000706.3 | 552 | Hs.2131 |
| AVPR1A | 206251_s_at | NM_000706.3 | 552 | Hs.2131 |
| AVPR1A | 206252_s_at | NM_000706.3 | 552 | Hs.2131 |
| AVPR1B | 208260_at | NM_000707.2 | 553 | Hs.1372 |
| AVPR2 | 208108_s_at | NM_000054.2 | 554 | Hs.3109 |
| AVPR2 | 208111_at | NM_000054.2 | 554 | Hs.3109 |
| AXL | 202685_s_at | NM_001699.3 | 558 | Hs.466791 |
| AXL | 202686_s_at | NM_021913.2 | 558 | Hs.466791 |
| AZGP1 | 215060_at | NM_001185 | 563 | Hs.407861 |
| AZGP1 | 217014_s_at | NM_001185.2 | 563 | Hs.546239 |
| AZGP1 | 1563539_at | NM_001185 | 563 | Hs.407861 |
| AZGP1 | 209309_at | NM_001185.2 | 563 | Hs.546239 |
| AZU1 | 214575_s_at | NM_001700.3 | 566 | Hs.72885 |
| B2M | 216231_s_at | NM_004048.2 | 567 | Hs.534255 |
| B2M | 232311_at | NM_004048 | 567 | Hs.48516 |
| B2M | 201891_s_at | NM_004048.2 | 567 | Hs.534255 |
| B3GNT3 | 204856_at | NM_014256.3 | 10331 | Hs.69009 |
| B4GALT1 | 216627_s_at | NM_001497.2 | 2683 | Hs.272011 |
| B4GALT1 | 228498_at | NM_001497 | 2683 | Hs.396798 |
| B4GALT1 | 229403_at | NM_001497 | 2683 | Hs.396798 |
| B4GALT1 | 238987_at | NM_001497 | 2683 | Hs.396798 |
| B4GALT1 | 201882_x_at | NM_001497.2 | 2683 | Hs.272011 |
| B4GALT1 | 201883_s_at | NM_001497.2 | 2683 | Hs.272011 |
| B4GALT1 | 211631_x_at | NM_001497.2 | 2683 | Hs.272011 |
| BACE1 | 217904_s_at | NM_012104.3 | 23621 | Hs.504003 |
| BACE1 | 222462_s_at | NM_138971.2 | 23621 | Hs.504003 |
| BACE1 | 222463_s_at | NM_012104.3 | 23621 | Hs.504003 |
| BACE1 | 224335_s_at | NM_012104.3 | 23621 | Hs.504003 |
| BACE2 | 217867_x_at | NM_012105.3 | 25825 | Hs.529408 |
| BACE2 | 222446_s_at | NM_138991.1 | 25825 | Hs.529408 |
| BAGE | 1555369_at | AF527554.1 | 574 | Hs.2355 |
| BAGE | 1555603_at | AF527551.1 | 574 | Hs.2355 |
| BAGE | 1555605_x_at | AF527551.1 | 574 | Hs.545789 |
| BAGE | 207712_at | NM_001187.1 | 574 | Hs.2355 |
| BAGE4 | 1555409_a_at | NM_181704.1 | 85317 | Hs.270492 |
| BAI1 | 206083_at | NM_001702.1 | 575 | Hs.194654 |
| BAI2 | 204966_at | NM_001703.1 | 576 | Hs.524138 |
| BAI3 | 205638_at | NM_001704.1 | 577 | Hs.13261 |
| BAI3 | 211568_at | NM_001704 | 577 | Hs.334087 |
| BAIAP3 | 216356_x_at | NM_003933.3 | 8938 | Hs.458427 |
| BAIAP3 | 204874_x_at | NM_003933 | 8938 | Hs.458427 |
| BAMBI | 203304_at | NM_012342.2 | 25805 | Hs.533336 |
| BASP1 | 228589_at | NM_006317 | 10409 | Hs.79516 |
| BASP1 | 202391_at | NM_006317.3 | 10409 | Hs.201641 |
| BAT5 | 224756_s_at | NM_021160.1 | 7920 | Hs.388188 |
| BAT5 | 230221_at | NM_021160 | 7920 | Hs.388188 |
| BBS1 | 218471_s_at | NM_024649.4 | 582 | Hs.502915 |
| BBS1 | 222643_s_at | NM_024649.4 | 582 | Hs.502915 |
| BBS1 | 229142_s_at | NM_024649 | 582 | Hs.129213 |
| BCAP31 | 200837_at | NM_005745.6 | 10134 | Hs.531539 |
| BCHE | 205433_at | NM_000055.1 | 590 | Hs.420483 |
| BCL2L11 | 222343_at | NM_006538.2 | 10018 | Hs.469658 |
| BCL2L11 | 1553088_a_at | NM_138621.2 | 10018 | Hs.469658 |
| BCL2L11 | 1553096_s_at | NM_006538.2 | 10018 | Hs.469658 |
| BCL2L11 | 1555372_at | NM_138622 | 10018 | Hs.469658 |
| BCL2L11 | 1558143_a_at | NM_006538.2 | 10018 | Hs.469658 |
| BCL2L11 | 1561844_at | NM_006538 | 10018 | Hs.416702 |
| BCL2L11 | 208536_s_at | NM_138622.2 | 10018 | Hs.469658 |
| BDKRB1 | 207510_at | NM_000710.2 | 623 | Hs.553486 |
| BDKRB2 | 205870_at | NM_000623.2 | 624 | Hs.525572 |
| BDNF | 239367_at | NM_001709.3 | 627 | Hs.502182 |
| BDNF | 1567359_at | NM_001709 | 627 | Hs.439027 |
| BDNF | 206382_s_at | NM_001709.3 | 627 | Hs.502182 |
| BF | 211920_at | NM_001710 | 629 | Hs.69771 |
| BFAR | 218056_at | NM_016561.1 | 51283 | Hs.435556 |
| BGLAP | 206956_at | NM_199173.2 | 632 | Hs.512679 |
| BGN | 213905_x_at | NM_001711.3 | 633 | Hs.821 |
| BGN | 201261_x_at | NM_001711.3 | 633 | Hs.821 |
| BGN | 201262_s_at | NM_001711.3 | 633 | Hs.821 |
| BIA2 | 241881_at | NM_015431 | 25893 | Hs.323858 |
| BICD2 | 1553021_s_at | NM_001003800.1 | 23299 | Hs.436939 |
| BICD2 | 209203_s_at | NM_001003800.1 | 23299 | Hs.436939 |
| BICD2 | 212702_s_at | NM_001003800.1 | 23299 | Hs.436939 |
| BICD2 | 213154_s_at | NM_001003800.1 | 23299 | Hs.436939 |
| BLCAP | 201032_at | NM_006698.2 | 10904 | Hs.472651 |
| BLR1 | 206126_at | NM_001716.2 | 643 | Hs.113916 |
| BLR1 | 216734_s_at | NM_001716.2 | 643 | Hs.113916 |
| BM88 | 219591_at | NM_016564 | 51286 | Hs.22140 |
| BMP1 | 1569001_at | AF318323.1 | 649 | Hs.1274 |
| BMP1 | 1569002_x_at | AF318323.1 | 649 | Hs.1274 |
| BMP1 | 202701_at | NM_006129.2 | 649 | Hs.1274 |
| BMP1 | 205574_x_at | NM_001199.1 | 649 | Hs.1274 |
| BMP1 | 206725_x_at | NM_006128.1 | 649 | Hs.1274 |
| BMP1 | 207595_s_at | NM_001199.1 | 649 | Hs.1274 |
| BMP10 | 208292_at | NM_014482.1 | 27302 | Hs.158317 |
| BMP15 | 221332_at | NM_005448.1 | 9210 | Hs.532692 |
| BMP2 | 205289_at | NM_001200.2 | 650 | Hs.73853 |
| BMP2 | 205290_s_at | NM_001200.2 | 650 | Hs.73853 |
| BMP3 | 208244_at | NM_001201.1 | 651 | Hs.121507 |
| BMP4 | 211518_s_at | NM_130850.1 | 652 | Hs.68879 |
| BMP5 | 205430_at | NM_021073 | 653 | Hs.1104 |
| BMP5 | 205431_s_at | NM_021073.2 | 653 | Hs.296648 |
| BMP6 | 241141_at | NM_001718 | 654 | Hs.285671 |
| BMP6 | 206176_at | NM_001718.2 | 654 | Hs.285671 |
| BMP7 | 209590_at | NM_001719 | 655 | Hs.170195 |
| BMP7 | 209591_s_at | NM_001719.1 | 655 | Hs.473163 |
| BMP7 | 211259_s_at | NM_001719.1 | 655 | Hs.473163 |
| BMP7 | 211260_at | NM_001719 | 655 | Hs.473163 |
| BMP8 | 235275_at | NM_001720 | 656 | Hs.409964 |
| BMP8A | 220203_at | NM_181809.2 | 353500 | Hs.494158 |
| BMP8A | 220204_s_at | NM_181809.2 | 353500 | Hs.494158 |
| BMP8A | 207865_s_at | NM_181809.2 | 353500 | Hs.494158 |
| BMP8A | 207866_at | NM_181809.2 | 353500 | Hs.494158 |
| BMPER | 241986_at | NM_133468.2 | 168667 | Hs.209226 |
| BMPR1A | 213578_at | NM_004329.2 | 657 | Hs.524477 |
| BMPR1A | 204832_s_at | NM_004329.2 | 657 | Hs.524477 |
| BMPR1B | 210523_at | NM_001203.1 | 658 | Hs.480321 |
| BMPR2 | 225144_at | NM_001204.5 | 659 | Hs.471119 |
| BMPR2 | 231873_at | NM_001204.5 | 659 | Hs.471119 |
| BMPR2 | 238516_at | NM_001204 | 659 | Hs.53250 |
| BMPR2 | 1556075_at | NM_001204 | 659 | Hs.53250 |
| BMPR2 | 209920_at | NM_001204.5 | 659 | Hs.471119 |
| BMPR2 | 210214_s_at | NM_001204.5 | 659 | Hs.471119 |
| BOC | 224184_s_at | NM_033254.2 | 91653 | Hs.556004 |
| BOC | 225990_at | NM_033254.2 | 91653 | Hs.556004 |
| BOC | 230035_at | NM_033254 | 91653 | Hs.556004 |
| BPI | 205557_at | NM_001725.1 | 671 | Hs.529019 |
| BPIL1 | 232698_at | NM_025227.1 | 80341 | Hs.257045 |
| BPIL2 | 1555773_at | NM_174932 | 254240 | Hs.372939 |
| BPIL3 | 1555535_at | NM_174897.1 | 128859 | Hs.375090 |
| BRI3 | 223376_s_at | NM_015379.3 | 25798 | Hs.270570 |
| BRI3 | 229798_s_at | NM_015379 | 25798 | Hs.410497 |
| BRI3BP | 231810_at | NM_080626 | 140707 | Hs.131886 |
| BRS3 | 1552818_a_at | NM_001727.1 | 680 | Hs.121484 |
| BRWD2 | 218090_s_at | NM_018117.10 | 55717 | Hs.144447 |
| BRWD2 | 229694_at | AK124522.1 | 55717 | Hs.144447 |
| BSG | 237667_at | NM_001728 | 682 | Hs.445233 |
| BSG | 208677_s_at | NM_001728.2 | 682 | Hs.501293 |
| BSND | 1552548_at | NM_057176.2 | 7809 | Hs.151291 |
| BSND | 1552549_a_at | NM_057176.2 | 7809 | Hs.151291 |
| BST1 | 205715_at | NM_004334.1 | 683 | Hs.169998 |
| BST2 | 201641_at | NM_004335.2 | 684 | Hs.118110 |
| BTBD11 | 228570_at | NM_001017523.1 | 121551 | Hs.271272 |
| BTBD11 | 238692_at | NM_001017523.1 | 121551 | Hs.506644 |
| BTC | 241412_at | NM_001729 | 685 | Hs.73105 |
| BTC | 207326_at | NM_001729.1 | 685 | Hs.558302 |
| BTD | 214116_at | NM_000060 | 686 | Hs.78885 |
| BTD | 214117_s_at | NM_000060.2 | 686 | Hs.517830 |
| BTD | 204167_at | NM_000060.2 | 686 | Hs.517830 |
| BTLA | 236226_at | NM_181780.2 | 151888 | Hs.445162 |
| BTN1A1 | 207395_at | NM_001732.1 | 696 | Hs.153058 |
| BTN2A1 | 215493_x_at | NM_007049.2 | 11120 | Hs.159028 |
| BTN2A1 | 203944_x_at | NM_007049.2 | 11120 | Hs.159028 |
| BTN2A1 | 211256_x_at | NM_007049 | 11120 | Hs.159028 |
| BTN2A2 | 1564684_at | NM_006995 | 10385 | Hs.91813 |
| BTN2A2 | 205298_s_at | NM_181531.1 | 10385 | Hs.373938 |
| BTN2A2 | 205299_s_at | NM_006995.3 | 10385 | Hs.373938 |
| BTN2A3 | 208585_at | NM_024018.1 | 54718 | Hs.370522 |
| BTN3A1 | 207485_x_at | NM_007048.4 | 11119 | Hs.191510 |
| BTN3A1 | 209770_at | NM_007048.4 | 11119 | Hs.191510 |
| BTN3A2 | 209846_s_at | NM_007047.3 | 11118 | Hs.376046 |
| BTN3A2 | 212613_at | NM_007047.3 | 11118 | Hs.376046 |
| BTN3A3 | 38241_at | NM_006994.3 | 10384 | Hs.167741 |
| BTN3A3 | 204820_s_at | NM_006994.3 | 10384 | Hs.167741 |
| BTN3A3 | 204821_at | NM_006994.3 | 10384 | Hs.167741 |
| BTNL2 | 221457_s_at | NM_019602.1 | 56244 | Hs.534471 |
| BTNL3 | 217207_s_at | NM_197975.1 | 10917 | Hs.225949 |
| BTNL8 | 220421_at | NM_001040462.1 | 79908 | Hs.189109 |
| BTNL8 | 234632_x_at | NM_001040462 | 79908 | 632607 |
| BTNL8 | 234640_x_at | NM_001040462 | 79908 | 632607 |
| BTNL9 | 228434_at | NM_152547.2 | 153579 | Hs.546502 |
| BTNL9 | 1553279_at | AK057097.1 | 153579 | Hs.546502 |
| BVES | 223853_at | NM_007073.3 | 11149 | Hs.221660 |
| BVES | 228783_at | NM_147147.2 | 11149 | Hs.221660 |
| BZRPL1 | 215449_at | NM_001010873.1 | 222642 | Hs.357392 |
| C10orf125 | 230259_at | NM_198472.1 | 282969 | Hs.155823 |
| C10orf128 | 228372_at | XM_931121.1 | 170371 | Hs.385493 |
| C10orf128 | 1561890_at | XM_926220.1 | 170371 | Hs.385493 |
| C10orf13 | 238453_at | NM_152429.2 | 143282 | Hs.466120 |
| C10orf25 | 1552422_at | NM_001039380.1 | 220979 | Hs.194303 |
| C10orf26 | 202808_at | AF070644.1 | 54838 | Hs.500897 |
| C10orf31 | 1560255_at | NM_001012713.1 | 414196 | Hs.309288 |
| C10orf35 | 226313_at | NM_145306.1 | 219738 | Hs.522992 |
| C10orf38 | 212771_at | NM_001010924.1 | 221061 | Hs.66762 |
| C10orf42 | 225320_at | NM_138357.1 | 90550 | Hs.128021 |
| C10orf49 | 236800_at | NM_145314.1 | 221044 | Hs.112998 |
| C10orf54 | 225372_at | NM_022153.1 | 64115 | Hs.546421 |
| C10orf54 | 225373_at | NM_022153.1 | 64115 | Hs.546421 |
| C10orf57 | 218174_s_at | NM_025125.2 | 80195 | Hs.169982 |
| C10orf57 | 222545_s_at | NM_025125.2 | 80195 | Hs.169982 |
| C10orf58 | 224435_at | NM_032333.2 | 84293 | Hs.500333 |
| C10orf58 | 228155_at | NM_032333.2 | 84293 | Hs.500333 |
| C10orf59 | 220564_at | NM_018363.1 | 55328 | Hs.149849 |
| C10orf59 | 223824_at | NM_001031709.1 | 55328 | Hs.149849 |
| C10orf61 | 212121_at | NM_001013840.1 | 26123 | Hs.438991 |
| C10orf61 | 212123_at | NM_001013840.1 | 26123 | Hs.438991 |
| C10orf64 | 1553863_at | BC034937.1 | 159491 | Hs.287379 |
| C10orf72 | 244057_s_at | NM_001031746.1 | 196740 | Hs.522928 |
| C10orf72 | 244058_at | NM_001031746.1 | 196740 | Hs.522928 |
| C10orf72 | 1553458_at | NM_144984.1 | 196740 | Hs.522928 |
| C10orf74 | 225785_at | BC018658.1 | 221035 | Hs.499833 |
| C10orf74 | 235016_at | NM_001001330.1 | 221035 | Hs.499833 |
| C10orf76 | 218891_at | NM_024541.1 | 79591 | Hs.16004 |
| C10orf76 | 55662_at | NM_024541.1 | 79591 | Hs.16004 |
| C10orf77 | 219745_at | NM_024789.3 | 79847 | Hs.309069 |
| C10orf99 | 227735_s_at | NM_207373.1 | 387695 | Hs.298713 |
| C10orf99 | 227736_at | NM_207373.1 | 387695 | Hs.298713 |
| C11orf10 | 218213_s_at | NM_014206.1 | 746 | Hs.437779 |
| C11orf10 | 207481_at | NM_014206 | 746 | Hs.437779 |
| C11orf11 | 214128_at | NM_006133.1 | 747 | Hs.241564 |
| C11orf2 | 217969_at | NM_013265.2 | 738 | Hs.277517 |
| C11orf24 | 218299_at | NM_022338.2 | 53838 | Hs.303025 |
| C11orf24 | 52164_at | NM_022338.2 | 53838 | Hs.303025 |
| C11orf38 | 243268_at | NM_212555.1 | 399967 | Hs.369185 |
| C11orf44 | 1553417_at | NM_173580.1 | 283171 | Hs.376151 |
| C11orf45 | 1553787_at | NM_145013.1 | 219833 | Hs.351133 |
| C11orf64 | 1554764_a_at | NM_174939.2 | 283197 | Hs.372650 |
| C11orf64 | 1554765_a_at | NM_174939.2 | 283197 | Hs.372650 |
| C11orf64 | 1563145_at | NM_174939 | 283197 | Hs.372650 |
| C12orf23 | 224759_s_at | NM_152261.1 | 90488 | Hs.257664 |
| C12orf28 | 1556267_at | NM_182530.1 | 196446 | Hs.253773 |
| C12orf3 | 234718_at | NM_020373 | 57101 | Hs.64616 |
| C12orf49 | 218867_s_at | NM_024738.1 | 79794 | Hs.528681 |
| C12orf49 | 222767_s_at | NM_024738.1 | 79794 | Hs.528681 |
| C12orf49 | 234983_at | NM_024738 | 79794 | Hs.120170 |
| C12orf51 | 1557529_at | NM_173813.2 | 283450 | Hs.558719 |
| C12orf51 | 1561130_at | NM_173813 | 283450 | Hs.379848 |
| C12orf53 | 228165_at | NM_153685.2 | 196500 | Hs.44067 |
| C12orf53 | 1553566_at | NM_153685.2 | 196500 | Hs.44067 |
| C12orf62 | 225772_s_at | NM_032901.2 | 84987 | Hs.388645 |
| C13orf16 | 236853_at | NM_152324.1 | 121793 | Hs.210677 |
| C13orf6 | 234993_at | NM_032859.2 | 84945 | Hs.183528 |
| C13orf6 | 235348_at | NM_032859.2 | 84945 | Hs.183528 |
| C14orf100 | 223215_s_at | NM_016475.2 | 51528 | Hs.446850 |
| C14orf101 | 219757_s_at | NM_017799.3 | 54916 | Hs.497253 |
| C14orf101 | 225675_at | NM_017799.3 | 54916 | Hs.497253 |
| C14orf109 | 213246_at | AI346504 | 26175 | Hs.275352 |
| C14orf118 | 219720_s_at | NM_017926.2 | 55668 | Hs.410231 |
| C14orf118 | 229514_at | NM_017926 | 55668 | Hs.410231 |
| C14orf118 | 229520_s_at | NM_017926 | 55668 | Hs.410231 |
| C14orf118 | 229673_at | NM_017926 | 55668 | Hs.352500 |
| C14orf118 | 231970_at | NM_017926 | 55668 | Hs.352500 |
| C14orf132 | 218820_at | NM_020215.2 | 56967 | Hs.6434 |
| C14orf132 | 231859_at | NM_020215.2 | 56967 | Hs.6434 |
| C14orf147 | 213508_at | NM_138288.2 | 171546 | Hs.269909 |
| C14orf147 | 212460_at | NM_138288.2 | 171546 | Hs.269909 |
| C14orf154 | 212465_at | NM_032233.2 | 84193 | Hs.510407 |
| C14orf154 | 229940_at | NM_199123.1 | 84193 | Hs.510407 |
| C14orf161 | 1570470_at | BC016315.1 | 79820 | Hs.131755 |
| C14orf161 | 220293_at | NM_024764.2 | 79820 | Hs.131755 |
| C14orf37 | 1557176_a_at | NM_001001872.2 | 145407 | Hs.535420 |
| C14orf58 | 1569536_at | NM_017791 | 55640 | Hs.509966 |
| C14orf58 | 219316_s_at | NM_017791.1 | 55640 | Hs.509966 |
| C14orf58 | 222866_s_at | NM_017791.1 | 55640 | Hs.509966 |
| C14orf83 | 227544_at | AK056646.1 | 161145 | Hs.509707 |
| C14orf83 | 238820_at | BC104940.1 | 161145 | Hs.509707 |
| C14orf83 | 1558177_at | AK090706.1 | 161145 | Hs.509707 |
| C14orf90 | 231216_at | BE671450 | 122625 | Hs.149974 |
| C14orf93 | 219009_at | NM_021944.1 | 60686 | Hs.255874 |
| C14orf93 | 239306_at | NM_021944 | 60686 | Hs.435536 |
| C15orf27 | 1552400_a_at | NM_152335.1 | 123591 | Hs.458965 |
| C16orf24 | 219709_x_at | NM_023933.1 | 65990 | Hs.166244 |
| C16orf30 | 219315_s_at | NM_024600.1 | 79652 | Hs.459652 |
| C16orf30 | 222865_x_at | NM_024600.1 | 79652 | Hs.459652 |
| C16orf30 | 228947_x_at | NM_024600.1 | 79652 | Hs.459652 |
| C16orf42 | 213104_at | NM_001001410.2 | 115939 | Hs.134846 |
| C16orf42 | 213105_s_at | NM_001001410.2 | 115939 | Hs.134846 |
| C16orf54 | 1559584_a_at | NM_175900.2 | 283897 | Hs.331095 |
| C16orf58 | 217891_at | NM_022744.1 | 64755 | Hs.9003 |
| C16orf58 | 222190_s_at | NM_022744.1 | 64755 | Hs.9003 |
| C16orf7 | 1558728_at | NM_004913 | 9605 | Hs.164410 |
| C16orf7 | 205781_at | NM_004913 | 9605 | Hs.164410 |
| C16orf77 | 237046_x_at | NM_152456.1 | 146433 | Hs.461214 |
| C16orf79 | 1553990_at | NM_182563.2 | 283870 | Hs.558724 |
| C16orf9 | 224749_at | NM_032039.1 | 83986 | Hs.513225 |
| C17orf28 | 225981_at | NM_030630.1 | 283987 | Hs.11067 |
| C17orf28 | 244593_at | NM_030630.1 | 283987 | Hs.11067 |
| C17orf32 | 225374_at | NM_152464.1 | 147007 | Hs.368878 |
| C17orf32 | 225375_at | NM_152464.1 | 147007 | Hs.368878 |
| C17orf56 | 227668_at | NM_144679.1 | 146705 | Hs.66219 |
| C17orf56 | 238980_x_at | NM_144679.1 | 146705 | Hs.66219 |
| C17orf62 | 218130_at | NM_001033046.1 | 79415 | Hs.163113 |
| C17orf69 | 1553399_a_at | NM_152466.1 | 147081 | Hs.128813 |
| C17orf69 | 1553400_a_at | NM_152466.1 | 147081 | Hs.128813 |
| C17orf77 | 1553298_at | NM_152460.2 | 146723 | Hs.350775 |
| C17orf78 | 1553486_a_at | NM_173625.3 | 284099 | Hs.439154 |
| C17orf80 | 221048_x_at | NM_017941.3 | 55028 | Hs.12929 |
| C17orf80 | 223351_at | NM_017941 | 55028 | Hs.12929 |
| C17orf80 | 223352_s_at | NM_017941.3 | 55028 | Hs.12929 |
| C18orf1 | 233138_at | NM_004338 | 753 | Hs.149363 |
| C18orf1 | 207996_s_at | NM_001003674.1 | 753 | Hs.149363 |
| C18orf1 | 209573_s_at | NM_001003674.1 | 753 | Hs.149363 |
| C18orf1 | 209574_s_at | NM_001003674.1 | 753 | Hs.149363 |
| C18orf15 | 1553435_at | AK055900.1 | 147276 | Hs.550158 |
| C18orf19 | 230739_at | NM_152352 | 125228 | Hs.13034 |
| C18orf19 | 235022_at | NM_152352.1 | 125228 | Hs.13034 |
| C18orf20 | 1553934_at | NM_152728.1 | 221241 | Hs.567323 |
| C18orf30 | 1562488_at | NM_207347 | 284221 | Hs.565176 |
| C18orf30 | 1563584_at | AK095053.1 | 284221 | Hs.565176 |
| C18orf4 | 232235_at | NM_032160.2 | 92126 | Hs.124673 |
| C18orf4 | 232825_s_at | NM_032160.2 | 92126 | Hs.124673 |
| C18orf4 | 244852_at | NM_032160.2 | 92126 | Hs.124673 |
| C18orf45 | 224493_x_at | AK126247.1 | 85019 | Hs.137562 |
| C18orf45 | 244495_x_at | NM_032933 | 85019 | Hs.137562 |
| C18orf50 | 1570552_at | AF363068.1 | 619463 | Hs.560596 |
| C18orf54 | 229442_at | NM_173529.3 | 162681 | Hs.208701 |
| C18orf54 | 244324_at | NM_173529.3 | 162681 | Hs.208701 |
| C18orf54 | 1553651_at | NM_173529.3 | 162681 | Hs.208701 |
| C18orf54 | 1553652_a_at | NM_173529.3 | 162681 | Hs.208701 |
| C18orf58 | 1564015_at | NM_173817.1 | 284222 | Hs.436902 |
| C19orf12 | 223983_s_at | NM_031448.2 | 83636 | Hs.529094 |
| C19orf12 | 225863_s_at | NM_001031726.1 | 83636 | Hs.529094 |
| C19orf12 | 227704_at | CR615514.1 | 83636 | Hs.529094 |
| C19orf15 | 221316_at | NM_021185.3 | 57828 | Hs.324335 |
| C19orf15 | 231261_at | NM_021185.3 | 57828 | Hs.324335 |
| C19orf15 | 234353_at | NM_021185 | 57828 | Hs.324335 |
| C19orf18 | 236847_at | NM_152474.2 | 147685 | Hs.134209 |
| C19orf24 | 221587_s_at | NM_017914.2 | 55009 | Hs.25489 |
| C19orf26 | 1552354_at | NM_152769.1 | 255057 | Hs.346575 |
| C19orf26 | 1552355_s_at | NM_152769.1 | 255057 | Hs.346575 |
| C19orf27 | 221267_s_at | NM_031213.2 | 81926 | Hs.458458 |
| C19orf28 | 220178_at | AF218008.1 | 126321 | Hs.245372 |
| C19orf28 | 226516_at | NM_174983.2 | 126321 | Hs.380252 |
| C19orf32 | 226597_at | NM_138393.1 | 92840 | Hs.76277 |
| C19orf36 | 214296_x_at | BC010443.1 | 113177 | Hs.424049 |
| C19orf36 | 215734_at | NM_001031735.2 | 113177 | Hs.424049 |
| C19orf41 | 240559_at | NM_152358.2 | 126123 | Hs.414175 |
| C19orf42 | 219097_x_at | NM_024104.2 | 79086 | Hs.356467 |
| C19orf42 | 221988_at | NM_024104 | 79086 | Hs.356467 |
| C19orf42 | 224712_x_at | AK025602.1 | 79086 | Hs.356467 |
| C19orf42 | 224717_s_at | NM_024104.2 | 79086 | Hs.356467 |
| C19orf42 | 229650_s_at | NM_024104 | 79086 | Hs.356467 |
| C19orf49 | 227327_at | NM_178121.2 | 90198 | Hs.558582 |
| C19orf56 | 217780_at | NM_016145.1 | 51398 | Hs.108969 |
| C19orf6 | 213986_s_at | NM_033420.3 | 91304 | Hs.515003 |
| C19orf6 | 225247_at | NM_001033026.1 | 91304 | Hs.515003 |
| C19orf6 | 230089_s_at | NM_033420 | 91304 | Hs.380962 |
| C19orf6 | 212574_x_at | NM_001033026.1 | 91304 | Hs.515003 |
| C19orf6 | 212575_at | NM_001033026.1 | 91304 | Hs.515003 |
| C19orf63 | 224727_at | NM_175063.4 | 284361 | Hs.515550 |
| C1GALT1 | 219439_at | NM_020156.1 | 56913 | Hs.239666 |
| C1GALT1C1 | 219283_at | NM_152692.3 | 29071 | Hs.134856 |
| C1GALT1C1 | 238989_at | NM_014158 | 29071 | Hs.537003 |
| C1orf101 | 233578_at | AK027205.1 | 257044 | Hs.459534 |
| C1orf101 | 240771_at | NM_173807 | 257044 | Hs.459534 |
| C1orf102 | 227359_at | NM_145047.3 | 127700 | Hs.202207 |
| C1orf115 | 218546_at | NM_024709.2 | 79762 | Hs.519839 |
| C1orf119 | 222495_at | NM_020141.3 | 56900 | Hs.82933 |
| C1orf151 | 224867_at | NM_001032363.1 | 440574 | Hs.558995 |
| C1orf151 | 228096_at | AK096240.1 | 440574 | Hs.558995 |
| C1orf159 | 219337_at | NM_017891.2 | 54991 | Hs.235095 |
| C1orf160 | 221512_at | NM_032125.1 | 84065 | Hs.469171 |
| C1orf160 | 52078_at | NM_032125.1 | 84065 | Hs.469171 |
| C1orf162 | 228532_at | NM_174896.2 | 128346 | Hs.288010 |
| C1orf175 | 214147_at | NM_001039464.1 | 374977 | Hs.476894 |
| C1orf179 | 1554668_a_at | NM_176782.1 | 338094 | Hs.554944 |
| C1orf186 | 222334_at | AW979289 | - | - |
| C1orf186 | 230381_at | NM_001007544.1 | 440712 | Hs.527097 |
| C1orf187 | 1554340_a_at | NM_198545.3 | 374946 | Hs.371716 |
| C1orf187 | 1556826_s_at | AK075558.1 | 374946 | Hs.371716 |
| C1orf2 | 1555515_a_at | NM_198264.1 | 10712 | Hs.348308 |
| C1orf2 | 203550_s_at | NM_006589.2 | 10712 | Hs.348308 |
| C1orf210 | 1554246_at | NM_182517.1 | 149466 | Hs.158963 |
| C1orf215 | 1552803_a_at | NM_152497.1 | 149421 | Hs.468736 |
| C1orf27 | 218721_s_at | NM_017847.3 | 54953 | Hs.371210 |
| C1orf27 | 222720_x_at | NM_017847.3 | 54953 | Hs.371210 |
| C1orf37 | 212164_at | NM_138391 | 92703 | Hs.497443 |
| C1orf54 | 219506_at | NM_024579.1 | 79630 | Hs.91283 |
| C1orf56 | 221222_s_at | NM_017860.3 | 54964 | Hs.549171 |
| C1orf56 | 223459_s_at | NM_017860.3 | 54964 | Hs.549171 |
| C1orf66 | 218914_at | NM_015997.2 | 51093 | Hs.512597 |
| C1orf71 | 225550_at | BX647629.1 | 163882 | Hs.368353 |
| C1orf71 | 225551_at | NM_152609.1 | 163882 | Hs.368353 |
| C1orf71 | 1554660_a_at | NM_152609.1 | 163882 | Hs.368353 |
| C1orf71 | 1554661_s_at | NM_152609.1 | 163882 | Hs.368353 |
| C1orf71 | 1570106_at | BC035193 | 163882 | 368353 |
| C1orf75 | 218814_s_at | NM_018252.1 | 55248 | Hs.445386 |
| C1orf75 | 222752_s_at | NM_018252.1 | 55248 | Hs.445386 |
| C1orf76 | 222095_s_at | NM_173509.2 | 148753 | Hs.400696 |
| C1orf78 | 220134_x_at | NM_018166.1 | 55194 | Hs.87016 |
| C1orf82 | 219504_s_at | NM_024813.1 | 79871 | Hs.444421 |
| C1orf82 | 222893_s_at | NM_024813.1 | 79871 | Hs.444421 |
| C1orf85 | 225401_at | NM_144580.1 | 112770 | Hs.202522 |
| C1orf85 | 1558692_at | NM_144580 | 112770 | Hs.202522 |
| C1orf85 | 1558693_s_at | NM_144580.1 | 112770 | Hs.202522 |
| C1orf9 | 203429_s_at | NM_014283.2 | 51430 | Hs.204559 |
| C1orf91 | 223656_s_at | AK075260.1 | 56063 | Hs.272299 |
| C1orf91 | 223676_at | AK075260.1 | 56063 | Hs.272299 |
| C1orf91 | 234006_s_at | AF258548.1 | 56063 | Hs.272299 |
| C1orf95 | 232104_at | NM_001003665.1 | 375057 | Hs.116827 |
| C1orf98 | 1562909_at | BC040731.1 | 554279 | Hs.434694 |
| C1QA | 218232_at | NM_015991.2 | 712 | Hs.9641 |
| C1QB | 202953_at | NM_000491.3 | 713 | Hs.8986 |
| C1QBP | 214214_s_at | NM_001212.3 | 708 | Hs.555866 |
| C1QBP | 1556088_at | NM_001212 | 708 | Hs.78614 |
| C1QBP | 208910_s_at | NM_001212.3 | 708 | Hs.555866 |
| C1QDC2 | 243292_at | XM_928940.1 | 388581 | Hs.559715 |
| C1QG | 225353_s_at | NM_172369.2 | 714 | Hs.467753 |
| C1QL1 | 205575_at | NM_006688.3 | 10882 | Hs.134012 |
| C1QR1 | 202877_s_at | NM_012072.3 | 22918 | Hs.97199 |
| C1QR1 | 202878_s_at | NM_012072.3 | 22918 | Hs.97199 |
| C1QTNF1 | 220975_s_at | NM_030968.2 | 114897 | Hs.201398 |
| C1QTNF1 | 224197_s_at | NM_030968.2 | 114897 | Hs.201398 |
| C1QTNF2 | 223749_at | NM_031908.4 | 114898 | Hs.110062 |
| C1QTNF4 | 223708_at | NM_031909.1 | 114900 | Hs.119302 |
| C1QTNF5 | 223499_at | NM_015645.2 | 114902 | Hs.157211 |
| C1QTNF6 | 223571_at | NM_031910.3 | 114904 | Hs.22011 |
| C1QTNF6 | 242444_at | NM_031910.3 | 114904 | Hs.22011 |
| C1QTNF7 | 223877_at | NM_031911.3 | 114905 | Hs.153714 |
| C1QTNF7 | 239349_at | NM_031911 | 114905 | Hs.153714 |
| C1QTNF9 | 1554233_at | NM_178540.3 | 338872 | Hs.362854 |
| C1R | 212067_s_at | NM_001733.4 | 715 | Hs.524224 |
| C1RL | 218983_at | NM_016546.1 | 51279 | Hs.525264 |
| C1RL | 233645_s_at | NM_016546.1 | 51279 | Hs.525264 |
| C1S | 1555229_a_at | NM_001734.2 | 716 | Hs.458355 |
| C1S | 208747_s_at | NM_001734.2 | 716 | Hs.458355 |
| C2 | 1554533_at | NM_000063 | 717 | Hs.408903 |
| C2 | 203052_at | NM_000063.3 | 717 | Hs.408903 |
| C20orf103 | 219463_at | NM_012261.2 | 24141 | Hs.22920 |
| C20orf107 | 232513_x_at | NM_001013646.2 | 388799 | Hs.287759 |
| C20orf108 | 224690_at | NM_080821.2 | 116151 | Hs.143736 |
| C20orf108 | 224693_at | NM_080821.2 | 116151 | Hs.143736 |
| C20orf114 | 226067_at | NM_033197.2 | 92747 | Hs.65551 |
| C20orf116 | 218159_at | NM_023935.1 | 65992 | Hs.471975 |
| C20orf134 | 232619_at | NM_001024675.1 | 170487 | Hs.324104 |
| C20orf141 | 231360_at | NM_080739.2 | 128653 | Hs.352187 |
| C20orf141 | 1554041_at | BC021178.2 | 128653 | Hs.352187 |
| C20orf141 | 1554042_s_at | BC021178.2 | 128653 | Hs.352187 |
| C20orf161 | 1553960_at | NM_033421.2 | 90203 | Hs.472854 |
| C20orf161 | 1553961_s_at | NM_033421.2 | 90203 | Hs.472854 |
| C20orf161 | 226595_at | NM_033421.2 | 90203 | Hs.472854 |
| C20orf165 | 1553752_at | NM_080608.3 | 128497 | Hs.128235 |
| C20orf186 | 233523_at | NM_182519.1 | 149954 | Hs.38961 |
| C20orf3 | 227857_at | NM_020531 | 57136 | Hs.22391 |
| C20orf3 | 228309_at | NM_020531 | 57136 | Hs.22391 |
| C20orf3 | 206656_s_at | NM_020531.2 | 57136 | Hs.472330 |
| C20orf30 | 220477_s_at | NM_001009924.1 | 29058 | Hs.472024 |
| C20orf30 | 224584_at | NM_001009923.1 | 29058 | Hs.472024 |
| C20orf31 | 218282_at | NM_018217.1 | 55741 | Hs.356273 |
| C20orf39 | 219310_at | NM_024893.1 | 79953 | Hs.124638 |
| C20orf46 | 219958_at | NM_018354.1 | 55321 | Hs.516834 |
| C20orf50 | 234018_s_at | AL137678 | 80343 | Hs.404127 |
| C20orf54 | 228236_at | NM_033409.2 | 113278 | Hs.283865 |
| C20orf54 | 233451_at | NM_033409 | 113278 | Hs.283865 |
| C20orf58 | 228017_s_at | NM_152864.2 | 128414 | Hs.46627 |
| C20orf58 | 228018_at | NM_152864.2 | 128414 | Hs.46627 |
| C20orf58 | 230668_at | NM_152864.2 | 128414 | Hs.46627 |
| C20orf58 | 230771_at | NM_152864 | 128414 | Hs.46627 |
| C20orf59 | 219559_at | NM_022082.2 | 63910 | Hs.512686 |
| C20orf59 | 232922_s_at | NM_022082.2 | 63910 | Hs.512686 |
| C20orf59 | 233328_x_at | NM_022082.2 | 63910 | Hs.512686 |
| C20orf59 | 243676_at | NM_022082 | 63910 | Hs.353013 |
| C20orf70 | 237099_at | NM_080574.2 | 140683 | Hs.147647 |
| C20orf75 | 1553169_at | NM_152611.2 | 164312 | Hs.149133 |
| C20orf75 | 1553171_x_at | NM_152611.2 | 164312 | Hs.149133 |
| C20orf82 | 232888_at | AL133463 | 140862 | Hs.348297 |
| C20orf82 | 235182_at | AI816793 | 140862 | Hs.348297 |
| C20orf98 | 218359_at | NM_024958.1 | 80023 | Hs.416024 |
| C21orf25 | 212875_s_at | NM_199050.1 | 25966 | Hs.473894 |
| C21orf29 | 1552570_at | NM_144991.2 | 54084 | Hs.554818 |
| C21orf29 | 1555048_a_at | NM_144991.2 | 54084 | Hs.554818 |
| C21orf29 | 1555049_at | NM_144991 | 54084 | Hs.554818 |
| C21orf51 | 1554430_at | NM_058182 | 54065 | Hs.303798 |
| C21orf51 | 1554432_x_at | NM_058182 | 54065 | Hs.303798 |
| C21orf51 | 228239_at | NM_058182 | 54065 | Hs.303798 |
| C21orf63 | 227188_at | NM_058187.3 | 59271 | Hs.208358 |
| C22orf24 | 207701_at | AL050256.1 | 25775 | Hs.556574 |
| C22orf3 | 204402_at | NM_012265.1 | 25807 | Hs.106730 |
| C22orf3 | 217622_at | NM_012265 | 25807 | Hs.106730 |
| C22orf5 | 202027_at | NM_012264.3 | 25829 | Hs.182626 |
| C22orf8 | 219629_at | NM_017911.1 | 55007 | Hs.265018 |
| C22orf8 | 226475_at | AK125027.1 | 55007 | Hs.265018 |
| C2orf17 | 218037_at | NM_024293.2 | 79137 | Hs.516707 |
| C2orf17 | 221983_at | AL833630.1 | 79137 | Hs.516707 |
| C2orf17 | 221984_s_at | NM_024293.2 | 79137 | Hs.516707 |
| C2orf24 | 200070_at | NM_015680.3 | 27013 | Hs.4973 |
| C2orf24 | 207511_s_at | NM_015680.3 | 27013 | Hs.4973 |
| C2orf28 | 219329_s_at | NM_016085.3 | 51374 | Hs.9527 |
| C2orf40 | 223623_at | NM_032411.1 | 84417 | Hs.43125 |
| C2orf7 | 223312_at | NM_032319.1 | 84279 | Hs.61268 |
| C3 | 217767_at | NM_000064.1 | 718 | Hs.529053 |
| C3AR1 | 209906_at | NM_004054.2 | 719 | Hs.527839 |
| C3orf17 | 225279_s_at | NM_001025072.2 | 25871 | Hs.477134 |
| C3orf17 | 225281_at | NM_001025072.2 | 25871 | Hs.477134 |
| C3orf18 | 219114_at | NM_016210.2 | 51161 | Hs.517860 |
| C3orf20 | 224273_at | NM_032137.3 | 84077 | Hs.506062 |
| C3orf21 | 226891_at | NM_152531.3 | 152002 | Hs.478741 |
| C3orf28 | 220942_x_at | NM_014367.3 | 26355 | Hs.5243 |
| C3orf28 | 221533_at | NM_014367 | 26355 | Hs.5243 |
| C3orf28 | 223193_x_at | NM_014367.3 | 26355 | Hs.5243 |
| C3orf28 | 224345_x_at | NM_014367.3 | 26355 | Hs.5243 |
| C3orf33 | 1554176_a_at | NM_173657.1 | 285315 | Hs.350846 |
| C3orf4 | 1554149_at | NM_001040182.1 | 56650 | Hs.107393 |
| C3orf4 | 208925_at | NM_001040183.1 | 56650 | Hs.107393 |
| C3orf4 | 239146_at | NM_019895 | 56650 | Hs.107393 |
| C3orf45 | 237024_at | NM_153215.1 | 132228 | Hs.534543 |
| C3orf58 | 226464_at | NM_173552.2 | 205428 | Hs.288954 |
| C3orf58 | 228079_at | NM_173552.2 | 205428 | Hs.288954 |
| C3orf64 | 221935_s_at | NM_173654.1 | 285203 | Hs.518059 |
| C4A | 235856_at | NM_007293 | 720 | Hs.150833 |
| C4A | 1559036_at | NM_007293 | 720 | Hs.150833 |
| C4B | 214428_x_at | NM_001002029.1 | 721 | Hs.546241 |
| C4B | 208451_s_at | NM_001002029.1 | 721 | Hs.546241 |
| C4BPA | 205654_at | NM_000715.3 | 722 | Hs.1012 |
| C4BPB | 241200_x_at | N68486 |  | Hs.193379 |
| C4BPB | 208209_s_at | NM_001017366.1 | 725 | Hs.99886 |
| C4orf13 | 1555108_at | BC023288.1 | 84068 | Hs.146162 |
| C4orf13 | 224126_at | NM_032128.2 | 84068 | Hs.146162 |
| C4orf13 | 235143_at | AK075364.1 | 84068 | Hs.146162 |
| C4orf26 | 236984_at | NM_178497.2 | 152816 | Hs.24510 |
| C4orf29 | 219980_at | NM_001039717.1 | 80167 | Hs.445817 |
| C4orf29 | 236240_at | NM_025097 | 80167 | Hs.445817 |
| C4orf31 | 215059_at | AA053967 |  | Hs.51515 |
| C4orf32 | 227856_at | NM_152400.1 | 132720 | Hs.23439 |
| C4orf34 | 224990_at | NM_174921.1 | 201895 | Hs.205952 |
| C4orf7 | 229152_at | NM_152997.2 | 260436 | Hs.320147 |
| C5 | 205500_at | NM_001735.2 | 727 | Hs.494997 |
| C5orf14 | 220495_s_at | NM_024715.3 | 79770 | Hs.106534 |
| C5orf14 | 227873_at | NM_024715.3 | 79770 | Hs.106534 |
| C5orf15 | 203024_s_at | NM_020199.1 | 56951 | Hs.355177 |
| C5orf16 | 1564031_a_at | NM_173828.3 | 285613 | Hs.558747 |
| C5orf18 | 208872_s_at | NM_005669.3 | 7905 | Hs.429608 |
| C5orf18 | 208873_s_at | NM_005669.3 | 7905 | Hs.429608 |
| C5orf19 | 205331_s_at | NM_016606.2 | 51308 | Hs.416090 |
| C5orf21 | 212936_at | NM_032042.3 | 83989 | Hs.567308 |
| C5orf26 | 225698_at | NM_053000.1 | 114915 | Hs.12082 |
| C5orf28 | 219029_at | NM_022483.3 | 64417 | Hs.558531 |
| C5orf28 | 238635_at | NM_022483 | 64417 | Hs.558531 |
| C5orf29 | 1552386_at | NM_152687.1 | 202309 | Hs.547697 |
| C5R1 | 220088_at | NM_001736.2 | 728 | Hs.2161 |
| C6 | 210168_at | NM_000065.1 | 729 | Hs.481992 |
| C6orf1 | 226306_at | NM_001008703.1 | 221491 | Hs.381300 |
| C6orf10 | 207523_at | NM_006781.2 | 10665 | Hs.57692 |
| C6orf105 | 229070_at | NM_032744.1 | 84830 | Hs.126409 |
| C6orf120 | 221786_at | NM_001029863.1 | 387263 | Hs.435933 |
| C6orf120 | 221787_at | NM_001029863.1 | 387263 | Hs.435933 |
| C6orf128 | 237016_at | NM_145316.2 | 221468 | Hs.520101 |
| C6orf129 | 225723_at | NM_138493.1 | 154467 | Hs.284207 |
| C6orf148 | 230055_at | NM_030568 | 80759 | Hs.556720 |
| C6orf15 | 221100_at | NM_014070.1 | 29113 | Hs.272214 |
| C6orf162 | 213312_at | NM_020425 | 57150 | Hs.70769 |
| C6orf162 | 213314_at | NM_020425 | 57150 | Hs.70769 |
| C6orf162 | 233438_at | NM_020425 | 57150 | Hs.70769 |
| C6orf162 | 236178_at | NM_020425 | 57150 | Hs.70769 |
| C6orf174 | 1569146_s_at | NM_001012279.1 | 387104 | Hs.319247 |
| C6orf188 | 232217_at | NM_153711.2 | 254228 | Hs.558706 |
| C6orf189 | 228875_at | XM_927381.1 | 221303 | Hs.126712 |
| C6orf192 | 226301_at | NM_052831.2 | 116843 | Hs.347144 |
| C6orf25 | 221342_at | NM_138274.1 | 80739 | Hs.247879 |
| C6orf27 | 221301_at | NM_025258.1 | 80737 | Hs.558553 |
| C6orf35 | 218453_s_at | NM_018452.2 | 55836 | Hs.157212 |
| C6orf35 | 222638_s_at | NM_018452.2 | 55836 | Hs.157212 |
| C6orf50 | 224183_at | AF210650.1 | 57052 | Hs.150858 |
| C6orf57 | 238504_at | NM_145267 | 135154 | Hs.71367 |
| C6orf58 | 238383_at | NM_001010905.1 | 352999 |  |
| C6orf68 | 225070_at | XM_372205.4 | 389850 | Hs.496078 |
| C6orf68 | 225071_at | XM_372205.4 | 389850 | Hs.496078 |
| C6orf69 | 214849_at | NM_173562.3 | 222658 | Hs.188757 |
| C6orf69 | 223176_at | NM_173562.3 | 222658 | Hs.188757 |
| C6orf69 | 228299_at | NM_173562 | 222658 | Hs.188757 |
| C6orf70 | 227656_at | NM_018341.1 | 55780 | Hs.47546 |
| C6orf71 | 231070_at | NM_203395.1 | 389434 | Hs.310225 |
| C6orf72 | 225576_at | NM_138785.1 | 116254 | Hs.438872 |
| C6orf78 | 1553388_at | NM_153036 | 221301 | Hs.350750 |
| C6orf82 | 221488_s_at | NM_001014838.1 | 51596 | Hs.520070 |
| C6orf85 | 1568718_at | NM_021945.4 | 63027 | Hs.132340 |
| C6orf85 | 1568719_s_at | NM_021945.4 | 63027 | Hs.132340 |
| C6orf85 | 223194_s_at | NM_021945 | 63027 | Hs.132340 |
| C6orf85 | 233206_at | AL110245.1 | 63027 | Hs.132340 |
| C6orf89 | 224977_at | NM_152734 | 221477 | Hs.433381 |
| C6orf89 | 224987_at | NM_152734 | 221477 | Hs.433381 |
| C6orf89 | 224988_at | NM_152734 | 221477 | Hs.433381 |
| C6orf89 | 225729_at | NM_152734 | 221477 | Hs.433381 |
| C6orf89 | 1556359_at | NM_152734 | 221477 | Hs.433381 |
| C7 | 235979_at | NM_000587 | 730 | Hs.78065 |
| C7 | 202992_at | NM_000587.2 | 730 | Hs.78065 |
| C7orf13 | 223630_at | NM_032625.1 | 129790 | Hs.124854 |
| C7orf19 | 1558426_x_at | NM_025156 | 80228 | Hs.363308 |
| C7orf19 | 218811_at | NM_025156 | 80228 | Hs.363308 |
| C7orf19 | 218812_s_at | NM_032831.1 | 80228 | Hs.363308 |
| C7orf19 | 230347_at | NM_025156 | 80228 | Hs.289053 |
| C7orf21 | 223153_x_at | NM_031434.2 | 83590 | Hs.238513 |
| C7orf23 | 204215_at | NM_024315.2 | 79161 | Hs.196129 |
| C7orf34 | 231435_at | NM_178829.2 | 135927 | Hs.239676 |
| C7orf34 | 1555757_at | NM_178829 | 135927 | Hs.239676 |
| C7orf38 | 238609_at | NM_145111.2 | 221786 | Hs.558692 |
| C7orf44 | 206497_at | NM_018224 | 55744 | Hs.289007 |
| C7orf44 | 209445_x_at | NM_018224.2 | 55744 | Hs.289007 |
| C7orf44 | 209446_s_at | NM_018224 | 55744 | Hs.289007 |
| C7orf45 | 1553329_at | NM_145268.2 | 136263 | Hs.351816 |
| C7orf45 | 1553330_at | NM_145268.2 | 136263 | Hs.351816 |
| C7orf53 | 239203_at | NM_182597.1 | 286006 | Hs.396189 |
| C7orf53 | 1569852_at | NM_182597.1 | 286006 | Hs.396189 |
| C7orf9 | 221322_at | NM_022150.3 | 64111 | Hs.60473 |
| C8A | 206305_s_at | NM_000562.1 | 731 | Hs.93210 |
| C8B | 206979_at | NM_000066.2 | 732 | Hs.391835 |
| C8G | 210324_at | NM_000606.1 | 733 | Hs.1285 |
| C8orf20 | 218777_at | AF218014.1 | 80346 | Hs.289063 |
| C8orf49 | 1564315_at | NM_001031839.1 | 606553 | Hs.545529 |
| C8orf5 | 236047_at | NM_182754 | 203080 | Hs.547796 |
| C8orf5 | 243260_x_at | NM_182754 | 203080 | Hs.197096 |
| C8orf55 | 218500_at | NM_016647.2 | 51337 | Hs.368402 |
| C8orf57 | 223614_at | AL136588.1 | 84257 | Hs.492187 |
| C9 | 206727_at | NM_001737 | 735 | Hs.1290 |
| C9orf11 | 232868_at | NM_020641.1 | 54586 | Hs.163070 |
| C9orf11 | 1554981_at | BC014307 | 54586 | Hs.163070 |
| C9orf11 | 1554982_a_at | NM_020641.1 | 54586 | Hs.163070 |
| C9orf123 | 224860_at | NM_033428.1 | 90871 | Hs.7517 |
| C9orf123 | 224879_at | NM_033428.1 | 90871 | Hs.7517 |
| C9orf125 | 224458_at | NM_032342.1 | 84302 | Hs.388742 |
| C9orf126 | 241754_at | NM_173690.1 | 286205 | Hs.59504 |
| C9orf127 | 229729_at | BC043384.1 | 51754 | Hs.493808 |
| C9orf127 | 207839_s_at | NM_016446.2 | 51754 | Hs.493808 |
| C9orf135 | 243610_at | NM_001010940.1 | 138255 | Hs.444459 |
| C9orf165 | 228403_at | NM_198573.2 | 375704 | Hs.522085 |
| C9orf19 | 225602_at | NM_022343.2 | 152007 | Hs.493819 |
| C9orf19 | 225604_s_at | NM_022343.2 | 152007 | Hs.493819 |
| C9orf26 | 209821_at | NM_033439.2 | 90865 | Hs.348390 |
| C9orf4 | 239097_at | AW296997 | 23732 | Hs.347537 |
| C9orf4 | 1558414_at | BI438189 | 23732 | Hs.347537 |
| C9orf47 | 231286_at | NM_001001938.1 | 286223 | Hs.292737 |
| C9orf47 | 1564274_at | NM_001001938.1 | 286223 | Hs.292737 |
| C9orf5 | 223005_s_at | NM_032012.1 | 23731 | Hs.308074 |
| C9orf5 | 223006_s_at | NM_032012.1 | 23731 | Hs.308074 |
| C9orf5 | 223007_s_at | NM_032012.1 | 23731 | Hs.308074 |
| C9orf5 | 223008_s_at | NM_032012.1 | 23731 | Hs.308074 |
| C9orf5 | 227831_at | NM_032012 | 23731 | Hs.418097 |
| C9orf5 | 237841_at | AI022702 |  | Hs.21627 |
| C9orf55 | 205684_s_at | NM_017925.4 | 55667 | Hs.249591 |
| C9orf55 | 226867_at | NM_017925.4 | 55667 | Hs.249591 |
| C9orf55 | 234968_at | AK000627.1 | 55667 | Hs.249591 |
| C9orf57 | 240316_at | XM_059954.7 | 138240 | Hs.371235 |
| C9orf61 | 213900_at | NM_004816.2 | 9413 | Hs.118003 |
| C9orf7 | 219223_at | NM_017586.1 | 11094 | Hs.62003 |
| C9orf7 | 61874_at | NM_017586.1 | 11094 | Hs.62003 |
| C9orf71 | 1553809_a_at | NM_153237.1 | 169693 | Hs.567318 |
| C9orf77 | 220285_at | NM_016014.2 | 51104 | Hs.380389 |
| C9orf77 | 227551_at | NM_001025780.1 | 51104 | Hs.380389 |
| C9orf79 | 1559559_at | NM_178828.3 | 286234 | Hs.130672 |
| C9orf79 | 1563868_a_at | NM_178828.3 | 286234 | Hs.130672 |
| C9orf91 | 221865_at | BC010029.2 | 203197 | Hs.522357 |
| CA11 | 209726_at | NM_001217.3 | 770 | Hs.428446 |
| CA12 | 214164_x_at | NM_001218.3 | 771 | Hs.210995 |
| CA12 | 215867_x_at | NM_001218.3 | 771 | Hs.210995 |
| CA12 | 203963_at | NM_001218.3 | 771 | Hs.210995 |
| CA12 | 204508_s_at | NM_001218.3 | 771 | Hs.210995 |
| CA12 | 204509_at | AK000158.1 | 771 | Hs.210995 |
| CA12 | 210735_s_at | NM_001218.3 | 771 | Hs.210995 |
| CA14 | 219464_at | NM_012113.1 | 23632 | Hs.528988 |
| CA14 | 237327_at | NM_012113 | 23632 | Hs.192491 |
| CA4 | 206208_at | NM_000717.2 | 762 | Hs.89485 |
| CA4 | 206209_s_at | NM_000717.2 | 762 | Hs.89485 |
| CA6 | 1555572_at | NM_001215 | 765 | Hs.100322 |
| CA6 | 206873_at | NM_001215.2 | 765 | Hs.100322 |
| CA9 | 205199_at | NM_001216.1 | 768 | Hs.63287 |
| CABP4 | 1552890_a_at | NM_145200.2 | 57010 | Hs.143036 |
| CABP4 | 1554201_at | NM_145200.2 | 57010 | Hs.143036 |
| CABP4 | 1554202_x_at | NM_145200.2 | 57010 | Hs.143036 |
| CACHD1 | 225627_s_at | NM_020925.1 | 57685 | Hs.443891 |
| CACNA1A | 214933_at | NM_000068 | 773 | Hs.408449 |
| CACNA1A | 206399_x_at | NM_023035.1 | 773 | Hs.408449 |
| CACNA1A | 210770_s_at | NM_000068.2 | 773 | Hs.408449 |
| CACNA1B | 235781_at | NM_000718 | 774 | Hs.495522 |
| CACNA1B | 207162_s_at | NM_000718.1 | 774 | Hs.495522 |
| CACNA1C | 220827_at | NM_000719 | 775 | Hs.272243 |
| CACNA1C | 208020_s_at | NM_000719.4 | 775 | Hs.372570 |
| CACNA1C | 211592_s_at | NM_000719.4 | 775 | Hs.372570 |
| CACNA1D | 228560_at | NM_000720 | 776 | Hs.399966 |
| CACNA1D | 1555993_at | NM_000720 | 776 | Hs.399966 |
| CACNA1D | 207998_s_at | NM_000720.1 | 776 | Hs.476358 |
| CACNA1D | 210108_at | NM_000720 | 776 | Hs.399966 |
| CACNA1E | 236013_at | NM_000721.2 | 777 | Hs.437444 |
| CACNA1E | 240650_at | NM_000721 | 777 | Hs.437444 |
| CACNA1E | 242410_s_at | AB209499.1 | 777 | Hs.437444 |
| CACNA1E | 208432_s_at | NM_000721.2 | 777 | Hs.437444 |
| CACNA1F | 208377_s_at | NM_005183.2 | 778 | Hs.139263 |
| CACNA1G | 1559948_at | NM_018896 | 8913 | Hs.194746 |
| CACNA1G | 207869_s_at | NM_018896 | 8913 | Hs.194746 |
| CACNA1G | 210380_s_at | NM_018896.3 | 8913 | Hs.194746 |
| CACNA1G | 211314_at | NM_198397.1 | 8913 | Hs.194746 |
| CACNA1G | 211315_s_at | NM_018896.3 | 8913 | Hs.194746 |
| CACNA1G | 211802_x_at | NM_018896.3 | 8913 | Hs.194746 |
| CACNA1H | 205845_at | NM_001005407.1 | 8912 | Hs.459642 |
| CACNA1I | 221631_at | NM_001003406.1 | 8911 | Hs.125116 |
| CACNA1I | 208299_at | NM_021096 | 8911 | Hs.125116 |
| CACNA1I | 211830_s_at | NM_001003406.1 | 8911 | Hs.125116 |
| CACNA1S | 217515_s_at | NM_000069 | 779 | Hs.1294 |
| CACNA2D1 | 207050_at | NM_000722.2 | 781 | Hs.282151 |
| CACNA2D2 | 229636_at | NM_006030 | 9254 | Hs.389415 |
| CACNA2D2 | 242806_at | NM_006030 | 9254 | Hs.389415 |
| CACNA2D2 | 204811_s_at | NM_001005505.1 | 9254 | Hs.476273 |
| CACNA2D3 | 219714_s_at | NM_018398.2 | 55799 | Hs.128594 |
| CACNA2D4 | 228083_at | NM_001005737.1 | 93589 | Hs.13768 |
| CACNA2D4 | 1552690_a_at | NM_001005737.1 | 93589 | Hs.13768 |
| CACNB1 | 206996_x_at | NM_199247.1 | 782 | Hs.635 |
| CACNB1 | 210185_at | NM_000723.3 | 782 | Hs.635 |
| CACNB1 | 210967_x_at | NM_199247.1 | 782 | Hs.635 |
| CACNB2 | 213714_at | NM_000724.2 | 783 | Hs.59093 |
| CACNB2 | 215365_at | AK022426.1 | 783 | Hs.59093 |
| CACNB2 | 1555098_a_at | NM_000724.2 | 783 | Hs.59093 |
| CACNB2 | 1559419_at | AK021994.1 | 783 | Hs.59093 |
| CACNB2 | 1559420_x_at | AK021994.1 | 783 | Hs.59093 |
| CACNB2 | 207776_s_at | NM_000724.2 | 783 | Hs.59093 |
| CACNB3 | 34726_at | NM_000725.2 | 784 | Hs.250712 |
| CACNB3 | 209530_at | NM_000725.2 | 784 | Hs.250712 |
| CACNG1 | 206612_at | NM_000727.2 | 786 | Hs.147989 |
| CACNG2 | 214495_at | NM_006078.2 | 10369 | Hs.197693 |
| CACNG3 | 206384_at | NM_006539.2 | 10368 | Hs.7235 |
| CACNG4 | 221585_at | NM_014405.2 | 27092 | Hs.514423 |
| CACNG4 | 231737_at | NM_014405.2 | 27092 | Hs.514423 |
| CACNG4 | 62987_r_at | NM_014405.2 | 27092 | Hs.514423 |
| CACNG5 | 221401_at | NM_014404.1 | 27091 | Hs.278907 |
| CACNG5 | 1552602_at | NM_145811.1 | 27091 | Hs.278907 |
| CACNG6 | 224291_at | NM_145814.1 | 59285 | Hs.326764 |
| CACNG6 | 1552863_a_at | NM_145815.1 | 59285 | Hs.326764 |
| CACNG7 | 224137_at | NM_031896.3 | 59284 | Hs.532740 |
| CACNG8 | 234750_at | NM_031895 | 59283 | Hs.326762 |
| CACNG8 | 234756_at | NM_031895.4 | 59283 | Hs.467273 |
| CALCA | 217495_x_at | NM_001033953.1 | 796 | Hs.37058 |
| CALCA | 217561_at | NM_001033953.1 | 796 | Hs.37058 |
| CALCA | 210727_at | NM_001033952.1 | 796 | Hs.37058 |
| CALCA | 210728_s_at | NM_001033952.1 | 796 | Hs.37058 |
| CALCB | 214636_at | NM_000728.3 | 797 | Hs.534305 |
| CALCR | 207886_s_at | NM_001742.2 | 799 | Hs.489127 |
| CALCR | 207887_s_at | NM_001742.2 | 799 | Hs.489127 |
| CALCRL | 234996_at | NM_005795 | 10203 | Hs.152175 |
| CALCRL | 206331_at | NM_005795.3 | 10203 | Hs.470882 |
| CALCRL | 210815_s_at | NM_005795.3 | 10203 | Hs.470882 |
| CALR | 214315_x_at | NM_004343.2 | 811 | Hs.515162 |
| CALR | 214316_x_at | NM_004343 | 811 | Hs.353170 |
| CALR | 1556931_at | NM_004343 | 811 | Hs.353170 |
| CALR | 200935_at | NM_004343.2 | 811 | Hs.515162 |
| CALR | 212953_x_at | NM_004343.2 | 811 | Hs.515162 |
| CAMLG | 203538_at | NM_001745.2 | 819 | Hs.529846 |
| CAMP | 210244_at | NM_004345.3 | 820 | Hs.51120 |
| CAPN10 | 219333_s_at | NM_021251.3 | 11132 | Hs.112218 |
| CAPN10 | 221040_at | NM_023089.1 | 11132 | Hs.112218 |
| CAPNS1 | 200001_at | NM_001003962.1 | 826 | Hs.515371 |
| CARTPT | 206339_at | NM_004291.2 | 9607 | Hs.1707 |
| CASC4 | 224619_at | NM_138423.2 | 113201 | Hs.512867 |
| CASC4 | 1552719_at | BC012124.1 | 113201 | Hs.512867 |
| CASC4 | 1559635_at | AK098265.1 | 113201 | Hs.512867 |
| CASC4 | 1570639_at | NM_138423 | 113201 | 512867 |
| CASD1 | 219342_at | NM_022900.3 | 64921 | Hs.260041 |
| CASP3 | 202763_at | NM_004346.3 | 836 | Hs.141125 |
| CASR | 240886_at | NM_000388 | 846 | Hs.55327 |
| CASR | 242744_s_at | NM_000388.2 | 846 | Hs.435615 |
| CASR | 242745_at | NM_000388 | 846 | Hs.55327 |
| CASR | 210577_at | NM_000388.2 | 846 | Hs.435615 |
| CASR | 211384_s_at | NM_000388.2 | 846 | Hs.435615 |
| CATSPER1 | 1552335_at | NM_053054.2 | 117144 | Hs.189105 |
| CATSPER2 | 217588_at | NM_172097.1 | 117155 | Hs.389181 |
| CATSPER2 | 1553323_a_at | NM_054020.2 | 117155 | Hs.389181 |
| CATSPER2 | 1561405_s_at | NM_054020.2 | 117155 | Hs.389181 |
| CATSPER3 | 230981_at | NM_178019.1 | 347732 | Hs.444355 |
| CAV1 | 203065_s_at | NM_001753.3 | 857 | Hs.74034 |
| CAV1 | 212097_at | NM_001753.3 | 857 | Hs.74034 |
| CAV2 | 203323_at | NM_001233.3 | 858 | Hs.212332 |
| CAV2 | 203324_s_at | NM_001233.3 | 858 | Hs.212332 |
| CAV3 | 208204_s_at | NM_001234.3 | 859 | Hs.98303 |
| CBARA1 | 216903_s_at | NM_006077.1 | 10367 | Hs.524367 |
| CBLN1 | 205747_at | NM_004352.1 | 869 | Hs.458423 |
| CBLN2 | 242301_at | NM_182511.2 | 147381 | Hs.514946 |
| CBLN4 | 234024_at | NM_080617.4 | 140689 | Hs.126141 |
| CBLN4 | 242524_at | NM_080617.4 | 140689 | Hs.126141 |
| CCBE1 | 242324_x_at | NM_133459.1 | 147372 | Hs.34333 |
| CCBE1 | 243805_at | NM_133459.1 | 147372 | Hs.34333 |
| CCBL2 | 209472_at | NM_001008661.1 | 56267 | Hs.481898 |
| CCBP2 | 239452_at | NM_001296 | 1238 | Hs.24286 |
| CCBP2 | 1568850_at | XM_933281.1 | 645943 | Hs.24286 |
| CCBP2 | 206887_at | NM_001296.3 | 1238 | Hs.24286 |
| CCDC107 | 229063_s_at | NM_174923.1 | 203260 | Hs.534579 |
| CCDC108 | 239508_x_at | NM_194302.2 | 255101 | Hs.528323 |
| CCDC108 | 1553886_at | NM_152389.2 | 255101 | Hs.147762 |
| CCDC108 | 1556969_at | BC039688.1 | 255101 | Hs.147762 |
| CCDC108 | 1557879_at | NM_152389.2 | 255101 | Hs.147762 |
| CCDC109B | 218802_at | NM_017918.3 | 55013 | Hs.234149 |
| CCDC126 | 228061_at | NM_138771.3 | 90693 | Hs.232296 |
| CCDC126 | 228087_at | NM_138771.3 | 90693 | Hs.232296 |
| CCDC134 | 220077_at | NM_024821.2 | 79879 | Hs.474991 |
| CCDC35 | 232240_at | XM_933488.1 | 387750 | Hs.567350 |
| CCDC47 | 217814_at | NM_020198.1 | 57003 | Hs.202011 |
| CCDC47 | 222432_s_at | NM_020198.1 | 57003 | Hs.202011 |
| CCDC51 | 218722_s_at | NM_024661.3 | 79714 | Hs.187657 |
| CCDC80 | 225241_at | NM_199512.1 | 151887 | Hs.477128 |
| CCDC80 | 225242_s_at | NM_199511.1 | 151887 | Hs.477128 |
| CCDC80 | 227061_at | AI088063 |  | Hs.86538 |
| CCDC80 | 243864_at | NM_199512.1 | 151887 | Hs.477128 |
| CCKAR | 211173_at | NM_000730 | 886 | Hs.129 |
| CCKAR | 211174_s_at | NM_000730.2 | 886 | Hs.129 |
| CCKBR | 234475_x_at | NM_000731 | 887 | Hs.203 |
| CCKBR | 210381_s_at | NM_176875.2 | 887 | Hs.203 |
| CCL1 | 207533_at | NM_002981.1 | 6346 | Hs.72918 |
| CCL11 | 210133_at | NM_002986.2 | 6356 | Hs.54460 |
| CCL13 | 206407_s_at | NM_005408.2 | 6357 | Hs.414629 |
| CCL14 | 205392_s_at | NM_004166.3 | 6358 | Hs.272493 |
| CCL14 | 210390_s_at | NM_004166.3 | 6358 | Hs.272493 |
| CCL16 | 207354_at | NM_004590.2 | 6360 | Hs.10458 |
| CCL17 | 207900_at | NM_002987.2 | 6361 | Hs.546294 |
| CCL18 | 32128_at | NM_002988.2 | 6362 | Hs.143961 |
| CCL18 | 209924_at | NM_002988.2 | 6362 | Hs.143961 |
| CCL19 | 210072_at | NM_006274.2 | 6363 | Hs.50002 |
| CCL2 | 216598_s_at | NM_002982.3 | 6347 | Hs.303649 |
| CCL20 | 205476_at | NM_004591.1 | 6364 | Hs.75498 |
| CCL21 | 204606_at | NM_002989.2 | 6366 | Hs.57907 |
| CCL22 | 207861_at | NM_002990.3 | 6367 | Hs.534347 |
| CCL23 | 210548_at | NM_005064.3 | 6368 | Hs.169191 |
| CCL23 | 210549_s_at | NM_005064.3 | 6368 | Hs.169191 |
| CCL24 | 221463_at | NM_002991.2 | 6369 | Hs.247838 |
| CCL25 | 206988_at | NM_005624 | 6370 | Hs.310511 |
| CCL26 | 223710_at | NM_006072.4 | 10344 | Hs.131342 |
| CCL27 | 230327_at | NM_006664 | 10850 | Hs.225948 |
| CCL27 | 207955_at | NM_006664.2 | 10850 | Hs.558456 |
| CCL28 | 224027_at | NM_019846 | 56477 | Hs.334633 |
| CCL28 | 224240_s_at | NM_019846 | 56477 | Hs.334633 |
| CCL3L1 | 205114_s_at | NM_021006.4 | 6349 | Hs.512683 |
| CCL4 | 204103_at | NM_002984.2 | 6351 | Hs.75703 |
| CCL5 | 235100_at | NM_002985 | 6352 | Hs.241392 |
| CCL5 | 1405_i_at | NM_002985.2 | 6352 | Hs.514821 |
| CCL5 | 1555759_a_at | NM_002985.2 | 6352 | Hs.514821 |
| CCL5 | 1561006_at | NM_002985 | 6352 | Hs.241392 |
| CCL5 | 204655_at | NM_002985 | 6352 | Hs.514821 |
| CCL7 | 208075_s_at | NM_006273.2 | 6354 | Hs.251526 |
| CCL8 | 214038_at | NM_005623.2 | 6355 | Hs.271387 |
| CCPG1 | 214151_s_at | NM_020739.2 | 9236 | Hs.285051 |
| CCPG1 | 214152_at | NM_004748.3 | 9236 | Hs.285051 |
| CCPG1 | 221156_x_at | BC027621.1 | 9236 | Hs.285051 |
| CCPG1 | 221511_x_at | NM_004748.3 | 9236 | Hs.285051 |
| CCPG1 | 222156_x_at | NM_004748.3 | 9236 | Hs.285051 |
| CCR1 | 205098_at | NM_001295.2 | 1230 | Hs.301921 |
| CCR1 | 205099_s_at | NM_001295.2 | 1230 | Hs.301921 |
| CCR10 | 220565_at | NM_016602.1 | 2826 | Hs.278446 |
| CCR2 | 206978_at | NM_000647 | 1231 | Hs.395 |
| CCR2 | 207794_at | NM_000647 | 1231 | Hs.511794 |
| CCR3 | 208304_at | NM_001837.2 | 1232 | Hs.506190 |
| CCR4 | 208376_at | NM_005508.4 | 1233 | Hs.184926 |
| CCR5 | 206991_s_at | NM_000579.1 | 1234 | Hs.450802 |
| CCR6 | 206983_at | NM_004367.3 | 1235 | Hs.46468 |
| CCR7 | 206337_at | NM_001838.2 | 1236 | Hs.370036 |
| CCR8 | 208059_at | NM_005201.2 | 1237 | Hs.113222 |
| CCR9 | 207445_s_at | NM_006641.2 | 10803 | Hs.225946 |
| CCRL1 | 220351_at | NM_178445.1 | 51554 | Hs.310512 |
| CCRL2 | 211434_s_at | NM_003965.4 | 9034 | Hs.458436 |
| CD109 | 226545_at | NM_133493 | 135228 | Hs.94030 |
| CD109 | 229900_at | NM_133493.1 | 135228 | Hs.399891 |
| CD109 | 239719_at | NM_133493.1 | 135228 | Hs.399891 |
| CD14 | 201743_at | NM_000591.2 | 929 | Hs.163867 |
| CD151 | 204306_s_at | NM_001039490.1 | 977 | Hs.512857 |
| CD160 | 207840_at | NM_007053.2 | 11126 | Hs.488237 |
| CD163 | 215049_x_at | NM_004244.3 | 9332 | Hs.504641 |
| CD163 | 216233_at | NM_004244 | 9332 | Hs.504641 |
| CD163 | 203645_s_at | NM_004244.3 | 9332 | Hs.504641 |
| CD163L1 | 223655_at | NM_174941.4 | 283316 | Hs.49636 |
| CD164 | 208405_s_at | NM_006016.3 | 8763 | Hs.520313 |
| CD164 | 208653_s_at | NM_006016.3 | 8763 | Hs.520313 |
| CD164 | 208654_s_at | NM_006016.3 | 8763 | Hs.520313 |
| CD177 | 219669_at | AJ290452.1 | 57126 | Hs.232165 |
| CD180 | 206206_at | NM_005582.1 | 4064 | Hs.87205 |
| CD19 | 206398_s_at | NM_001770.4 | 930 | Hs.96023 |
| CD1A | 210325_at | NM_001763.1 | 909 | Hs.1309 |
| CD1B | 206749_at | NM_001764.1 | 910 | Hs.1310 |
| CD1C | 205987_at | NM_001765.1 | 911 | Hs.1311 |
| CD1D | 205789_at | NM_001766.2 | 912 | Hs.1799 |
| CD1E | 215784_at | NM_030893 | 913 | Hs.249217 |
| CD1E | 208592_s_at | NM_030893.1 | 913 | Hs.249217 |
| CD2 | 205831_at | NM_001767.2 | 914 | Hs.523500 |
| CD200 | 209582_s_at | NM_001004196.2 | 4345 | Hs.79015 |
| CD200 | 209583_s_at | NM_001004196.2 | 4345 | Hs.79015 |
| CD200R1 | 1552875_a_at | NM_138806.3 | 131450 | Hs.309158 |
| CD200R1 | 1553395_a_at | NM_138806.3 | 131450 | Hs.309158 |
| CD207 | 220428_at | NM_015717.2 | 50489 | Hs.199731 |
| CD209 | 1555729_a_at | NM_021155.2 | 30835 | Hs.278694 |
| CD209 | 207277_at | NM_021155.2 | 30835 | Hs.278694 |
| CD209 | 207278_s_at | NM_021155.2 | 30835 | Hs.278694 |
| CD22 | 217422_s_at | NM_001771.1 | 933 | Hs.262150 |
| CD22 | 220674_at | AK026467.1 | 79978 | Hs.262150 |
| CD22 | 38521_at | NM_001771.1 | 933 | Hs.262150 |
| CD22 | 204581_at | NM_001771.1 | 933 | Hs.262150 |
| CD226 | 207315_at | NM_006566.1 | 10666 | Hs.369661 |
| CD24 | 216379_x_at | NM_013230.2 | 934 | Hs.375108 |
| CD24 | 266_s_at | NM_013230.2 | 934 | Hs.375108 |
| CD24 | 208650_s_at | NM_013230.2 | 934 | Hs.375108 |
| CD24 | 208651_x_at | NM_013230.2 | 934 | Hs.375108 |
| CD24 | 209771_x_at | NM_013230.2 | 934 | Hs.375108 |
| CD24 | 209772_s_at | NM_013230.2 | 934 | Hs.375108 |
| CD244 | 220307_at | NM_016382.2 | 51744 | Hs.157872 |
| CD244 | 234320_at | NM_016382 | 51744 | Hs.157872 |
| CD248 | 219025_at | NM_020404.2 | 57124 | Hs.195727 |
| CD274 | 223834_at | NM_014143.2 | 29126 | Hs.521989 |
| CD276 | 224859_at | NM_001024736.1 | 80381 | Hs.77873 |
| CD276 | 1552914_a_at | NM_001024736.1 | 80381 | Hs.77873 |
| CD276 | 1559583_at | AK074849.1 | 80381 | Hs.77873 |
| CD28 | 206545_at | NM_006139.1 | 940 | Hs.1987 |
| CD28 | 211856_x_at | NM_006139.1 | 940 | Hs.1987 |
| CD28 | 211861_x_at | NM_006139.1 | 940 | Hs.1987 |
| CD2AP | 203593_at | NM_012120.1 | 23607 | Hs.485518 |
| CD300A | 217072_at | NM_007261 | 11314 | Hs.9688 |
| CD300A | 217078_s_at | NM_007261.2 | 11314 | Hs.9688 |
| CD300A | 209933_s_at | NM_007261.2 | 11314 | Hs.9688 |
| CD300C | 207270_x_at | NM_006678.2 | 10871 | Hs.2605 |
| CD300LB | 1554173_at | NM_174892.1 | 124599 | Hs.313343 |
| CD300LB | 1554175_at | NM_174892.1 | 124599 | Hs.313343 |
| CD300LF | 1553043_a_at | NM_139018.2 | 146722 | Hs.235768 |
| CD300LG | 1552509_a_at | NM_145273.2 | 146894 | Hs.147313 |
| CD300LG | 1555636_at | AF427619.1 | 146894 | Hs.147313 |
| CD300LG | 1555656_at | AF427620.1 | 146894 | Hs.147313 |
| CD302 | 203799_at | NM_014880.3 | 9936 | Hs.130014 |
| CD33 | 206120_at | NM_001772.2 | 945 | Hs.83731 |
| CD33L3 | 215856_at | AK025833.1 | 284266 | Hs.287692 |
| CD34 | 209543_s_at | NM_001025109.1 | 947 | Hs.374990 |
| CD36 | 228766_at | NM_000072 | 948 | Hs.120949 |
| CD36 | 242197_x_at | NM_000072 | 948 | Hs.443120 |
| CD36 | 206488_s_at | NM_000072.2 | 948 | Hs.120949 |
| CD36 | 209554_at | NM_000072 | 948 | Hs.443120 |
| CD36 | 209555_s_at | NM_001001548.1 | 948 | Hs.120949 |
| CD37 | 204192_at | NM_001040031.1 | 951 | Hs.166556 |
| CD38 | 234187_at | NM_001775 | 952 | Hs.174944 |
| CD38 | 205692_s_at | NM_001775.2 | 952 | Hs.479214 |
| CD3D | 213539_at | NM_000732 | 915 | Hs.504048 |
| CD3E | 205456_at | NM_000733.2 | 916 | Hs.3003 |
| CD3G | 206804_at | NM_000073.1 | 917 | Hs.2259 |
| CD3Z | 210031_at | NM_000734.2 | 919 | Hs.156445 |
| CD4 | 216424_at | NM_000616 | 920 | Hs.17483 |
| CD4 | 203547_at | NM_000616.3 | 920 | Hs.17483 |
| CD40 | 215346_at | NM_001250.4 | 958 | Hs.472860 |
| CD40 | 222292_at | AB209660.1 | 958 | Hs.472860 |
| CD40 | 35150_at | NM_152854.2 | 958 | Hs.472860 |
| CD40 | 205153_s_at | NM_001250.4 | 958 | Hs.472860 |
| CD40LG | 207892_at | NM_000074.2 | 959 | Hs.652 |
| CD44 | 216056_at | NM_000610 | 960 | Hs.306278 |
| CD44 | 216062_at | NM_000610 | 960 | Hs.306278 |
| CD44 | 217523_at | NM_000610 | 960 | Hs.502328 |
| CD44 | 229221_at | NM_000610 | 960 | Hs.306278 |
| CD44 | 234411_x_at | NM_000610 | 960 | Hs.306278 |
| CD44 | 234418_x_at | NM_000610 | 960 | Hs.306278 |
| CD44 | 1557905_s_at | NM_000610.3 | 960 | Hs.502328 |
| CD44 | 1565868_at | AF086543.1 | 960 | Hs.502328 |
| CD44 | 204489_s_at | NM_000610.3 | 960 | Hs.502328 |
| CD44 | 204490_s_at | NM_001001389.1 | 960 | Hs.502328 |
| CD44 | 209835_x_at | NM_001001391.1 | 960 | Hs.502328 |
| CD44 | 210916_s_at | NM_000610.3 | 960 | Hs.502328 |
| CD44 | 212014_x_at | NM_001001389.1 | 960 | Hs.502328 |
| CD44 | 212063_at | NM_000610.3 | 960 | Hs.502328 |
| CD47 | 213856_at | NM_001777 | 961 | Hs.446414 |
| CD47 | 213857_s_at | NM_001025079.1 | 961 | Hs.446414 |
| CD47 | 226016_at | NM_001025079.1 | 961 | Hs.446414 |
| CD47 | 227259_at | NM_001025079.1 | 961 | Hs.446414 |
| CD47 | 242974_at | BC042889.1 | 961 | Hs.446414 |
| CD47 | 211075_s_at | NM_001025079.1 | 961 | Hs.446414 |
| CD47 | 213055_at | NM_001025079.1 | 961 | Hs.446414 |
| CD48 | 233137_at | NM_001778 | 962 | Hs.901 |
| CD48 | 237759_at | NM_001778 | 962 | Hs.243564 |
| CD48 | 204118_at | NM_001778.2 | 962 | Hs.243564 |
| CD5 | 230489_at | NM_014207.2 | 921 | Hs.58685 |
| CD5 | 206485_at | NM_014207.2 | 921 | Hs.58685 |
| CD52 | 34210_at | NM_001803.2 | 1043 | Hs.276770 |
| CD52 | 204661_at | NM_001803.2 | 1043 | Hs.276770 |
| CD53 | 237606_at | NM_000560 | 963 | Hs.443057 |
| CD53 | 242946_at | NM_000560 | 963 | Hs.443057 |
| CD53 | 203416_at | NM_000560.3 | 963 | Hs.443057 |
| CD58 | 216942_s_at | NM_001779.1 | 965 | Hs.34341 |
| CD58 | 222061_at | NM_001779 | 965 | Hs.75626 |
| CD58 | 205173_x_at | NM_001779.1 | 965 | Hs.34341 |
| CD58 | 211744_s_at | NM_001779.1 | 965 | Hs.34341 |
| CD59 | 228748_at | NM_000611.4 | 966 | Hs.278573 |
| CD59 | 200983_x_at | NM_000611.4 | 966 | Hs.278573 |
| CD59 | 200984_s_at | NM_000611.4 | 966 | Hs.278573 |
| CD59 | 200985_s_at | NM_000611.4 | 966 | Hs.278573 |
| CD59 | 212463_at | NM_000611.4 | 966 | Hs.278573 |
| CD5L | 206680_at | NM_005894.1 | 922 | Hs.134035 |
| CD6 | 213958_at | NM_006725.2 | 923 | Hs.502710 |
| CD6 | 1566447_at | NM_006725 | 923 | Hs.436949 |
| CD6 | 1566448_at | NM_006725 | 923 | Hs.436949 |
| CD6 | 208602_x_at | NM_006725.2 | 923 | Hs.502710 |
| CD6 | 211893_x_at | NM_006725.2 | 923 | Hs.502710 |
| CD6 | 211900_x_at | NM_006725.2 | 923 | Hs.502710 |
| CD63 | 235487_at | NM_001780 | 967 | Hs.445570 |
| CD63 | 200663_at | NM_001040034.1 | 967 | Hs.445570 |
| CD68 | 203507_at | NM_001251 | 968 | Hs.246381 |
| CD69 | 209795_at | NM_001781.1 | 969 | Hs.208854 |
| CD7 | 214049_x_at | NM_006137.6 | 924 | Hs.36972 |
| CD7 | 214551_s_at | NM_006137.6 | 924 | Hs.36972 |
| CD72 | 215925_s_at | NM_001782.1 | 971 | Hs.116481 |
| CD74 | 1567627_at | NM_004355 | 972 | Hs.446471 |
| CD74 | 1567628_at | NM_001025159.1 | 972 | Hs.436568 |
| CD74 | 209619_at | NM_001025159.1 | 972 | Hs.436568 |
| CD79A | 1555779_a_at | NM_001783.3 | 973 | Hs.79630 |
| CD79A | 205049_s_at | NM_001783.3 | 973 | Hs.79630 |
| CD79B | 1555746_at | NM_000626 | 974 | Hs.89575 |
| CD79B | 1555748_x_at | NM_000626 | 974 | Hs.89575 |
| CD79B | 205297_s_at | NM_001039933.1 | 974 | Hs.89575 |
| CD80 | 1554519_at | NM_005191.2 | 941 | Hs.838 |
| CD80 | 1555689_at | NM_005191 | 941 | Hs.838 |
| CD80 | 207176_s_at | NM_005191 | 941 | Hs.838 |
| CD81 | 200675_at | NM_004356.3 | 975 | Hs.54457 |
| CD82 | 203904_x_at | NM_001024844.1 | 3732 | Hs.527778 |
| CD83 | 204440_at | NM_001040280.1 | 9308 | Hs.484703 |
| CD84 | 244352_at | NM_003874 | 8832 | Hs.398093 |
| CD84 | 205988_at | NM_003874.1 | 8832 | Hs.398093 |
| CD84 | 211188_at | NM_003874 | 8832 | Hs.398093 |
| CD84 | 211189_x_at | NM_003874 | 8832 | Hs.398093 |
| CD84 | 211190_x_at | NM_003874.1 | 8832 | Hs.398093 |
| CD84 | 211191_at | NM_003874 | 8832 | Hs.398093 |
| CD84 | 211192_s_at | NM_003874.1 | 8832 | Hs.398093 |
| CD86 | 205685_at | NM_006889.3 | 942 | Hs.171182 |
| CD86 | 205686_s_at | NM_006889.3 | 942 | Hs.171182 |
| CD86 | 210895_s_at | NM_006889.3 | 942 | Hs.171182 |
| CD8A | 205758_at | NM_001768.5 | 925 | Hs.85258 |
| CD8B1 | 207979_s_at | NM_004931.3 | 926 | Hs.405667 |
| CD8B1 | 215332_s_at | NM_172102.2 | 926 | Hs.405667 |
| CD9 | 201005_at | NM_001769.2 | 928 | Hs.114286 |
| CD96 | 1555120_at | BC020749.1 | 10225 | Hs.142023 |
| CD96 | 206761_at | NM_005816.4 | 10225 | Hs.142023 |
| CD97 | 202910_s_at | NM_001025160.1 | 976 | Hs.466039 |
| CD99 | 201028_s_at | NM_002414.3 | 4267 | Hs.495605 |
| CD99 | 201029_s_at | NM_002414.3 | 4267 | Hs.495605 |
| CD99L2 | 223041_at | NM_031462.2 | 83692 | Hs.522805 |
| CD99L2 | 233825_s_at | NM_031462.2 | 83692 | Hs.522805 |
| CD99L2 | 233844_at | NM_031462 | 83692 | Hs.169388 |
| CD99L2 | 1554758_a_at | NM_031462.2 | 83692 | Hs.522805 |
| CDA | 205627_at | NM_001785.2 | 978 | Hs.466910 |
| CDA08 | 1556151_at | NM_030790 | 81533 | Hs.23047 |
| CDAN1 | 228516_at | NM_138477.2 | 146059 | Hs.437819 |
| CDAN1 | 231080_at | NM_138477 | 146059 | Hs.437819 |
| CDC91L1 | 225903_at | NM_080476.4 | 128869 | Hs.253319 |
| CDCP1 | 218451_at | NM_022842.3 | 64866 | Hs.476093 |
| CDCP1 | 234932_s_at | NM_022842.3 | 64866 | Hs.476093 |
| CDCP1 | 1554110_at | NM_178181.1 | 64866 | Hs.476093 |
| CDH1 | 201130_s_at | NM_004360.2 | 999 | Hs.461086 |
| CDH1 | 201131_s_at | NM_004360.2 | 999 | Hs.461086 |
| CDH10 | 220115_s_at | NM_006727.2 | 1008 | Hs.92489 |
| CDH11 | 236179_at | NM_001797 | 1009 | Hs.443435 |
| CDH11 | 239286_at | NM_001797 | 1009 | Hs.443435 |
| CDH11 | 239769_at | NM_001797 | 1009 | Hs.443435 |
| CDH11 | 207172_s_at | NM_001797.2 | 1009 | Hs.116471 |
| CDH11 | 207173_x_at | NM_001797.2 | 1009 | Hs.116471 |
| CDH12 | 207149_at | NM_004061.2 | 1010 | Hs.113684 |
| CDH13 | 244091_at | NM_001257 | 1012 | Hs.199852 |
| CDH13 | 204726_at | NM_001257 | 1012 | Hs.63984 |
| CDH15 | 206327_s_at | NM_004933.2 | 1013 | Hs.148090 |
| CDH15 | 206328_at | NM_004933.2 | 1013 | Hs.148090 |
| CDH16 | 206517_at | NM_004062.2 | 1014 | Hs.513660 |
| CDH17 | 209847_at | NM_004063.2 | 1015 | Hs.89436 |
| CDH18 | 206280_at | NM_004934.2 | 1016 | Hs.317632 |
| CDH19 | 1555403_a_at | NM_021153.2 | 28513 | Hs.42771 |
| CDH19 | 206898_at | NM_021153.2 | 28513 | Hs.42771 |
| CDH2 | 203440_at | NM_001792.2 | 1000 | Hs.464829 |
| CDH2 | 203441_s_at | NM_001792.2 | 1000 | Hs.464829 |
| CDH20 | 210913_at | NM_031891.2 | 28316 | Hs.147340 |
| CDH22 | 215181_at | NM_021248.1 | 64405 | Hs.472861 |
| CDH22 | 1569679_at | NM_021248 | 64405 | Hs.382126 |
| CDH23 | 224527_at | NM_022124.2 | 64072 | Hs.567294 |
| CDH23 | 232845_at | NM_022124 | 64072 | Hs.567294 |
| CDH23 | 232846_s_at | NM_022124.2 | 64072 | Hs.567294 |
| CDH23 | 1552436_a_at | NM_022124.2 | 64072 | Hs.567294 |
| CDH24 | 230157_at | NM_022478 | 64403 | Hs.155912 |
| CDH24 | 1553166_at | NM_022478 | 64403 | Hs.155912 |
| CDH26 | 232306_at | NM_021810.3 | 60437 | Hs.54973 |
| CDH26 | 233391_at | NM_021810 | 60437 | Hs.54973 |
| CDH26 | 233662_at | NM_021810.3 | 60437 | Hs.54973 |
| CDH26 | 233663_s_at | NM_177980.1 | 60437 | Hs.54973 |
| CDH3 | 203256_at | NM_001793.3 | 1001 | Hs.191842 |
| CDH4 | 220227_at | NM_024883 | 1002 | Hs.217754 |
| CDH4 | 1563587_at | NM_001794 | 1002 | Hs.217754 |
| CDH4 | 206866_at | NM_001794.2 | 1002 | Hs.217754 |
| CDH5 | 204677_at | NM_001795.2 | 1003 | Hs.76206 |
| CDH6 | 205532_s_at | NM_004932.2 | 1004 | Hs.171054 |
| CDH6 | 205533_s_at | NM_004932.2 | 1004 | Hs.171054 |
| CDH6 | 210601_at | NM_004932 | 1004 | Hs.171054 |
| CDH6 | 210602_s_at | NM_004932.2 | 1004 | Hs.171054 |
| CDH7 | 220679_s_at | NM_004361.2 | 1005 | Hs.130306 |
| CDH7 | 1554700_at | NM_004361 | 1005 | Hs.130306 |
| CDH8 | 217574_at | NM_001796 | 1006 | Hs.388928 |
| CDH8 | 241500_at | AI374911 |  | Hs.387405 |
| CDH8 | 1564291_at | AK026763 | 1006 | 368322 |
| CDH8 | 210518_at | NM_001796.2 | 1006 | Hs.368322 |
| CDH9 | 207729_at | NM_016279.3 | 1007 | Hs.272212 |
| CDIPT | 201253_s_at | NM_006319.2 | 10423 | Hs.121549 |
| CDKAL1 | 214877_at | NM_017774 | 54901 | Hs.484844 |
| CDKAL1 | 220039_s_at | NM_017774.2 | 54901 | Hs.484844 |
| CDKAL1 | 241732_at | NM_017774 |  | Hs.484844 |
| CDKAL1 | 1561025_at | NM_017774 |  | Hs.484844 |
| CDKAL1 | 1561026_a_at | NM_017774 |  | Hs.484844 |
| CDKAL1 | 210698_at | NM_017774 |  | Hs.484844 |
| CDON | 227526_at | NM_016952.3 | 50937 | Hs.38034 |
| CDON | 1563677_at | NM_016952 | 50937 | Hs.377022 |
| CDON | 207230_at | NM_016952.3 | 50937 | Hs.38034 |
| CDSN | 206192_at | NM_001264.3 | 1041 | Hs.310958 |
| CDSN | 206193_s_at | NM_001264 | 1041 | Hs.310958 |
| CEACAM1 | 206576_s_at | NM_001024912.1 | 634 | Hs.512682 |
| CEACAM1 | 209498_at | NM_001024912.1 | 634 | Hs.512682 |
| CEACAM1 | 210610_at | NM_001712 | 634 | Hs.512682 |
| CEACAM1 | 211883_x_at | NM_001712.3 | 634 | Hs.512682 |
| CEACAM1 | 211889_x_at | NM_001712.3 | 634 | Hs.512682 |
| CEACAM19 | 230504_at | BC083499.1 | 56971 | Hs.416925 |
| CEACAM21 | 214907_at | NM_033543.2 | 90273 | Hs.528704 |
| CEACAM21 | 216605_s_at | NM_033543.2 | 90273 | Hs.528704 |
| CEACAM3 | 208052_x_at | NM_001815.1 | 1084 | Hs.11 |
| CEACAM3 | 210789_x_at | NM_001815.1 | 1084 | Hs.11 |
| CEACAM4 | 207205_at | NM_001817 | 1089 | Hs.12 |
| CEACAM5 | 217291_at | Z21818 | 1048 | 466814 |
| CEACAM5 | 201884_at | NM_004363 | 1048 | Hs.220529 |
| CEACAM6 | 203757_s_at | NM_002483.3 | 4680 | Hs.466814 |
| CEACAM6 | 211657_at | NM_002483.3 | 4680 | Hs.466814 |
| CEACAM7 | 206198_s_at | NM_006890.1 | 1087 | Hs.74466 |
| CEACAM7 | 206199_at | NM_006890.1 | 1087 | Hs.74466 |
| CEACAM7 | 211848_s_at | NM_006890.1 | 1087 | Hs.74466 |
| CEACAM8 | 206676_at | NM_001816.2 | 1088 | Hs.41 |
| CECR1 | 219505_at | NM_017424.2 | 51816 | Hs.170310 |
| CECR9 | 1567686_at | AF307449 | 30847 | 542791 |
| CECR9 | 1567687_at | AF307449.1 | 30847 | Hs.542791 |
| CEL | 1553970_s_at | NM_001807.2 | 1056 | Hs.533258 |
| CEL | 205910_s_at | NM_001807.2 | 1056 | Hs.533258 |
| CELSR1 | 217262_s_at | NM_014246.1 | 9620 | Hs.252387 |
| CELSR1 | 41660_at | NM_014246.1 | 9620 | Hs.252387 |
| CELSR1 | 204539_s_at | NM_014246.1 | 9620 | Hs.252387 |
| CELSR2 | 36499_at | NM_001408.1 | 1952 | Hs.57652 |
| CELSR2 | 204029_at | NM_001408.1 | 1952 | Hs.57652 |
| CELSR3 | 40020_at | NM_001407.1 | 1951 | Hs.533070 |
| CELSR3 | 205165_at | NM_001407.1 | 1951 | Hs.533070 |
| CER1 | 221378_at | NM_005454.2 | 9350 | Hs.248204 |
| CerCAM | 227381_at | NM_016174 | 51148 | Hs.23954 |
| CES4 | 206824_at | NM_016280.1 | 51716 | Hs.535486 |
| CES7 | 1553465_a_at | NM_145024.1 | 221223 | Hs.350800 |
| CETP | 206210_s_at | NM_000078.1 | 1071 | Hs.89538 |
| CFH | 213800_at | NM_001014975.1 | 3075 | Hs.363396 |
| CFH | 215388_s_at | NM_000186.2 | 3075 | Hs.363396 |
| CFHL2 | 206910_x_at | NM_005666.2 | 3080 | Hs.154224 |
| CFHL3 | 1554459_s_at | BC020687.1 | 10878 | Hs.2637 |
| CFHL3 | 1570228_at | NM_021023 | 10878 | Hs.2637 |
| CFHL3 | 207874_s_at | NM_021023.3 | 10878 | Hs.2637 |
| CFHL5 | 208088_s_at | NM_030787.1 | 81494 | Hs.282594 |
| CFL1 | 231194_at | AK055496.1 | 254122 | Hs.533600 |
| CFTR | 215702_s_at | NM_000492.3 | 1080 | Hs.489786 |
| CFTR | 215703_at | NM_000492 | 1080 | Hs.489786 |
| CFTR | 217026_at | M96936 | 1080 | Hs.489786 |
| CFTR | 234702_x_at | NM_000492 | 1080 | Hs.489786 |
| CFTR | 234706_x_at | NM_000492 | 1080 | Hs.489783 |
| CFTR | 205043_at | NM_000492.3 | 1080 | Hs.489786 |
| CGA | 233615_at |  | 1081 | Hs.119689 |
| CGA | 204637_at | NM_000735.2 | 1081 | Hs.119689 |
| CGI-67 | 228872_at | NM_016014 | 51104 | Hs.380389 |
| CHAD | 206869_at | NM_001267.1 | 1101 | Hs.97220 |
| CHGA | 204697_s_at | NM_001275.2 | 1113 | Hs.150793 |
| CHGB | 204260_at | NM_001819.1 | 1114 | Hs.516874 |
| CHI3L1 | 216546_s_at | AJ251847 | 1116 | 382202 |
| CHI3L1 | 209395_at | NM_001276.1 | 1116 | Hs.382202 |
| CHI3L1 | 209396_s_at | NM_001276.1 | 1116 | Hs.382202 |
| CHIA | 220630_s_at | NM_201653.1 | 27159 | Hs.128814 |
| CHIC1 | 228345_at | CR936642.1 | 53344 | Hs.496323 |
| CHIC1 | 1557786_s_at | NM_001039840.1 | 53344 | Hs.496323 |
| CHIC1 | 1559481_at | NM_001039840.1 | 53344 | Hs.496323 |
| CHIC2 | 219492_at | NM_012110.2 | 26511 | Hs.335393 |
| CHL1 | 234583_at | NM_006614 | 10752 | Hs.388344 |
| CHL1 | 243432_at | XM_931148.1 | 642891 | Hs.388344 |
| CHL1 | 204591_at | NM_006614.2 | 10752 | Hs.148909 |
| CHMP1B | 218178_s_at | NM_020412.3 | 57132 | Hs.551551 |
| CHMP2A | 202121_s_at | NM_014453.2 | 27243 | Hs.12107 |
| CHMP6 | 218743_at | NM_024591.3 | 79643 | Hs.514560 |
| CHODL | 219867_at | NM_024944.2 | 140578 | Hs.283725 |
| CHRD | 221674_s_at | NM_003741.2 | 8646 | Hs.166186 |
| CHRD | 211248_s_at | NM_003741.2 | 8646 | Hs.166186 |
| CHRDL1 | 209763_at | AK092245.1 | 91851 | Hs.496587 |
| CHRDL2 | 223987_at | NM_015424.3 | 25884 | Hs.432379 |
| CHRFAM7A | 230861_at | NM_139320 | 89832 | Hs.353211 |
| CHRM1 | 231783_at | NM_000738.2 | 1128 | Hs.247917 |
| CHRM2 | 221330_at | NM_001006628.1 | 1129 | Hs.527965 |
| CHRM3 | 1553705_a_at | NM_000740 | 1131 | Hs.7138 |
| CHRM3 | 1559633_a_at | NM_000740.2 | 1131 | Hs.559103 |
| CHRM3 | 1559634_at | AK056349.1 | 1131 | Hs.7138 |
| CHRM3 | 1564339_a_at | NM_000740.2 | 1131 | Hs.559103 |
| CHRM4 | 221357_at | NM_000741.2 | 1132 | Hs.248100 |
| CHRM5 | 221347_at | NM_012125.2 | 1133 | Hs.128216 |
| CHRNA1 | 206633_at | NM_000079.2 | 1134 | Hs.434419 |
| CHRNA1 | 211039_at | NM_000079 | 1134 | Hs.434419 |
| CHRNA10 | 220210_at | NM_020402.2 | 57053 | Hs.157714 |
| CHRNA10 | 239717_at | NM_020402 | 57053 | Hs.157714 |
| CHRNA10 | 1568675_at | NM_020402 | 57053 | Hs.157714 |
| CHRNA2 | 207868_at | NM_000742.1 | 1135 | Hs.558310 |
| CHRNA3 | 210221_at | NM_000743.2 | 1136 | Hs.1614 |
| CHRNA3 | 211587_x_at | NM_000743 | 1136 | Hs.1614 |
| CHRNA3 | 211772_x_at | NM_000743.2 | 1136 | Hs.1614 |
| CHRNA4 | 216900_s_at | NM_000744.3 | 1137 | Hs.10734 |
| CHRNA4 | 206735_at | NM_000744.3 | 1137 | Hs.10734 |
| CHRNA4 | 206736_x_at | NM_000744.3 | 1137 | Hs.10734 |
| CHRNA5 | 206533_at | NM_000745.2 | 1138 | Hs.1614 |
| CHRNA6 | 207568_at | NM_004198.2 | 8973 | Hs.103128 |
| CHRNA7 | 210123_s_at | NM_000746.3 | 1139 | Hs.511772 |
| CHRNA9 | 221107_at | NM_017581.2 | 55584 | Hs.272278 |
| CHRNB1 | 206703_at | NM_000747.2 | 1140 | Hs.330386 |
| CHRNB2 | 241389_at | NM_000748 | 1141 | Hs.2306 |
| CHRNB2 | 206635_at | NM_000748.1 | 1141 | Hs.2306 |
| CHRNB3 | 207859_s_at | NM_000749 | 1142 | Hs.96094 |
| CHRNB4 | 207516_at | NM_000750.2 | 1143 | Hs.54397 |
| CHRND | 207024_at | NM_000751.1 | 1144 | Hs.156289 |
| CHRNE | 207274_at | NM_000080.2 | 1145 | Hs.313227 |
| CHRNG | 221355_at | NM_005199.4 | 1146 | Hs.248101 |
| CILP | 206227_at | NM_003613.2 | 8483 | Hs.442180 |
| CILP2 | 1552288_at | AK127735.1 | 148113 | Hs.279574 |
| CILP2 | 1552289_a_at | AK127735.1 | 148113 | Hs.279574 |
| CKLF | 219161_s_at | NM_001040138.1 | 51192 | Hs.15159 |
| CKLF | 221058_s_at | NM_181640.1 | 51192 | Hs.15159 |
| CKLF | 223451_s_at | NM_001040138.1 | 51192 | Hs.15159 |
| CLCA2 | 217528_at | NM_006536.4 | 9635 | Hs.241551 |
| CLCA2 | 206164_at | NM_006536 | 9635 | Hs.241551 |
| CLCA2 | 206165_s_at | NM_006536.4 | 9635 | Hs.241551 |
| CLCA2 | 206166_s_at | NM_006536.4 | 9635 | Hs.241551 |
| CLCA3 | 220810_at | NM_004921.2 | 9629 | Hs.546328 |
| CLCA4 | 220026_at | NM_012128.2 | 22802 | Hs.555012 |
| CLCF1 | 219500_at | NM_013246.2 | 23529 | Hs.502977 |
| CLCN1 | 208437_at | NM_000083.1 | 1180 | Hs.121483 |
| CLCN2 | 213499_at | NM_004366 | 1181 | Hs.567231 |
| CLCN3 | 201732_s_at | NM_173872.2 | 1182 | Hs.481186 |
| CLCN3 | 201735_s_at | NM_001829.2 | 1182 | Hs.481186 |
| CLCN4 | 214769_at | NM_001830 | 1183 | Hs.417091 |
| CLCN4 | 217556_at | NM_001830 | 1183 | Hs.417091 |
| CLCN4 | 231066_s_at | NM_001830 | 1183 | Hs.417091 |
| CLCN4 | 205148_s_at | NM_001830.2 | 1183 | Hs.495674 |
| CLCN4 | 205149_s_at | NM_001830.2 | 1183 | Hs.495674 |
| CLCN5 | 206704_at | NM_000084.1 | 1184 | Hs.535985 |
| CLCN6 | 203950_s_at | NM_021735.1 | 1185 | Hs.193043 |
| CLCN7 | 221961_at | NM_001287 | 1186 | Hs.459649 |
| CLCN7 | 38069_at | NM_001287.3 | 1186 | Hs.459649 |
| CLCN7 | 209235_at | NM_001287.3 | 1186 | Hs.459649 |
| CLCNKB | 1554748_at | NM_000085 | 1188 | Hs.352243 |
| CLCNKB | 1554749_s_at | NM_000085.1 | 1188 | Hs.352243 |
| CLCNKB | 205985_x_at | NM_000085.1 | 1188 | Hs.352243 |
| CLCNKB | 207047_s_at | NM_000085.1 | 1188 | Hs.352243 |
| CLDN1 | 218182_s_at | NM_021101.3 | 9076 | Hs.439060 |
| CLDN1 | 222549_at | NM_021101.3 | 9076 | Hs.439060 |
| CLDN10 | 1556687_a_at | NM_006984 | 9071 | Hs.157002 |
| CLDN10 | 205328_at | NM_006984.3 | 9071 | Hs.157002 |
| CLDN11 | 228335_at | NM_005602.4 | 5010 | Hs.31595 |
| CLDN11 | 206908_s_at | NM_005602.4 | 5010 | Hs.31595 |
| CLDN11 | 206909_at | NM_005602 | 5010 | Hs.31595 |
| CLDN12 | 223249_at | NM_012129.2 | 9069 | Hs.258576 |
| CLDN14 | 210689_at | NM_012130.2 | 23562 | Hs.505146 |
| CLDN15 | 219640_at | NM_014343.1 | 24146 | Hs.423968 |
| CLDN15 | 1552845_at | NM_138429.1 | 24146 | Hs.38738 |
| CLDN16 | 220332_at | NM_006580.2 | 10686 | Hs.251391 |
| CLDN17 | 221328_at | NM_012131.1 | 26285 | Hs.258589 |
| CLDN18 | 214135_at | NM_001002026.2 | 51208 | Hs.240182 |
| CLDN18 | 221132_at | NM_016369.3 | 51208 | Hs.240182 |
| CLDN18 | 221133_s_at | NM_001002026.2 | 51208 | Hs.240182 |
| CLDN18 | 232578_at | NM_001002026.2 | 51208 | Hs.240182 |
| CLDN19 | 1552535_at | NM_148960.1 | 149461 | Hs.496270 |
| CLDN19 | 1554804_a_at | NM_148960.1 | 149461 | Hs.496270 |
| CLDN19 | 1554805_at | NM_148960 | 149461 | Hs.496270 |
| CLDN2 | 223509_at | NM_020384.2 | 9075 | Hs.522746 |
| CLDN20 | 1554812_at | NM_001001346.1 | 49861 | Hs.352244 |
| CLDN23 | 228706_s_at | NM_194284.1 | 137075 | Hs.183617 |
| CLDN23 | 228707_at | NM_194284.1 | 137075 | Hs.183617 |
| CLDN3 | 203953_s_at | NM_001306.2 | 1365 | Hs.25640 |
| CLDN3 | 203954_x_at | NM_001306.2 | 1365 | Hs.25640 |
| CLDN4 | 1569421_at | AK126315.1 | 1364 | Hs.520942 |
| CLDN4 | 201428_at | NM_001305.3 | 1364 | Hs.520942 |
| CLDN5 | 204482_at | NM_003277.2 | 7122 | Hs.505337 |
| CLDN6 | 237810_at | NM_021195.3 | 9074 | Hs.533779 |
| CLDN6 | 208474_at | NM_021195.3 | 9074 | Hs.533779 |
| CLDN7 | 202790_at | NM_001307.3 | 1366 | Hs.513915 |
| CLDN8 | 214598_at | NM_199328.1 | 9073 | Hs.162209 |
| CLDN9 | 214635_at | NM_020982.2 | 9080 | Hs.296949 |
| CLEC10A | 206682_at | NM_006344.2 | 10462 | Hs.54403 |
| CLEC11A | 205131_x_at | NM_002975.2 | 6320 | Hs.512680 |
| CLEC11A | 210783_x_at | NM_002975.2 | 6320 | Hs.512680 |
| CLEC11A | 211709_s_at | NM_002975.2 | 6320 | Hs.512680 |
| CLEC12A | 1552398_a_at | NM_138337.4 | 160364 | Hs.190519 |
| CLEC12A | 1569401_at | BC027967.1 | 160364 | Hs.190519 |
| CLEC12B | 231357_at | NM_205852.1 | 387837 | Hs.127937 |
| CLEC14A | 226244_at | NM_175060.1 | 161198 | Hs.525307 |
| CLEC1A | 219761_at | NM_016511.2 | 51267 | Hs.29549 |
| CLEC1B | 220496_at | NM_016509.2 | 51266 | Hs.409794 |
| CLEC2B | 1556209_at | NM_005127.2 | 9976 | Hs.85201 |
| CLEC2B | 209732_at | NM_005127.2 | 9976 | Hs.85201 |
| CLEC2D | 220132_s_at | NM_001004420.1 | 29121 | Hs.268326 |
| CLEC2D | 233500_x_at | NM_001004419.1 | 29121 | Hs.268326 |
| CLEC2L | 232173_at | AK057548.1 | 154790 | Hs.57806 |
| CLEC3B | 205200_at | NM_003278.1 | 7123 | Hs.476092 |
| CLEC4A | 219947_at | NM_194447.1 | 50856 | Hs.504657 |
| CLEC4A | 221724_s_at | NM_016184.2 | 50856 | Hs.504657 |
| CLEC4C | 1552552_s_at | NM_203503.1 | 170482 | Hs.351812 |
| CLEC4C | 1555687_a_at | NM_130441.2 | 170482 | Hs.564493 |
| CLEC4E | 219859_at | NM_014358.2 | 26253 | Hs.236516 |
| CLEC4E | 222934_s_at | NM_014358.2 | 26253 | Hs.236516 |
| CLEC4F | 1552410_at | NM_173535.2 | 165530 | Hs.354972 |
| CLEC4G | 1559065_a_at | NM_198492.1 | 339390 | Hs.220649 |
| CLEC4M | 207995_s_at | NM_214677.1 | 10332 | Hs.421437 |
| CLEC4M | 210481_s_at | NM_014257.3 | 10332 | Hs.421437 |
| CLEC5A | 219890_at | NM_013252.2 | 23601 | Hs.446235 |
| CLEC7A | 221698_s_at | NM_022570.4 | 64581 | Hs.143929 |
| CLEC7A | 1554406_a_at | NM_197947.2 | 64581 | Hs.143929 |
| CLEC7A | 1555213_a_at | AF400601.1 | 64581 | Hs.143929 |
| CLEC7A | 1555214_a_at | NM_022570.4 | 64581 | Hs.143929 |
| CLEC7A | 1555756_a_at | NM_022570.4 | 64581 | Hs.143929 |
| CLECL1 | 244413_at | NM_172004.2 | 160365 | Hs.434223 |
| CLIC1 | 208659_at | NM_001288.4 | 1192 | Hs.414565 |
| CLIC6 | 227742_at | NM_053277.1 | 54102 | Hs.473695 |
| CLIC6 | 242913_at | NM_053277.1 | 54102 | Hs.473695 |
| CLINT1 | 201768_s_at | NM_014666.2 | 9685 | Hs.132853 |
| CLINT1 | 201769_at | NM_014666.2 | 9685 | Hs.132853 |
| CLMN | 213839_at | AB007969.1 | 57237 | Hs.301478 |
| CLMN | 221042_s_at | NM_024734.2 | 79789 | Hs.301478 |
| CLMN | 225757_s_at | NM_024734.2 | 79789 | Hs.301478 |
| CLMN | 225759_x_at | NM_024734 | 79789 | Hs.301478 |
| CLN3 | 209275_s_at | NM_000086 | 1201 | Hs.558312 |
| CLN3 | 210859_x_at | NM_000086 | 1201 | Hs.558312 |
| CLNS1A | 209143_s_at | NM_001293.1 | 1207 | Hs.430733 |
| CLPS | 206131_at | NM_001832.2 | 1208 | Hs.1340 |
| CLPTM1 | 201640_x_at | NM_001294.1 | 1209 | Hs.444441 |
| CLPTM1 | 211136_s_at | NM_001294.1 | 1209 | Hs.444441 |
| CLSTN1 | 201561_s_at | NM_001009566.1 | 22883 | Hs.29665 |
| CLSTN2 | 219414_at | NM_022131.1 | 64084 | Hs.158529 |
| CLSTN3 | 204375_at | NM_014718 | 9746 | Hs.535378 |
| CLTA | 216293_at | NM_001833 | 1211 | Hs.207052 |
| CLTA | 216295_s_at | NM_001833.1 | 1211 | Hs.522114 |
| CLTA | 216296_at | NM_001833 | 1211 | Hs.207052 |
| CLTA | 200960_x_at | NM_007096.1 | 1211 | Hs.522114 |
| CLTA | 204050_s_at | NM_007096.1 | 1211 | Hs.522114 |
| CLTC | 220855_at | NM_004859 | 1213 | Hs.187416 |
| CLTC | 239871_at | NM_004859 | 1213 | Hs.187416 |
| CLTC | 200614_at | NM_004859.2 | 1213 | Hs.491351 |
| CLTC | 210498_at | NM_004859 | 1213 | Hs.187416 |
| CLU | 222043_at | NM_001831.2 | 1191 | Hs.436657 |
| CLU | 231498_at | NM_001831 | 1191 | Hs.436657 |
| CLU | 231499_s_at | NM_001831 | 1191 | Hs.436657 |
| CLU | 1559228_at | NM_001831 | 1191 | Hs.436657 |
| CLU | 208791_at | NM_203339.1 | 1191 | Hs.436657 |
| CLU | 208792_s_at | NM_001831.2 | 1191 | Hs.436657 |
| CLUL1 | 1561375_at | NM_014410 | 27098 | Hs.26886 |
| CLUL1 | 206556_at | NM_014410.4 | 27098 | Hs.274959 |
| CMA1 | 214533_at | NM_001836.2 | 1215 | Hs.135626 |
| CMKLR1 | 207652_s_at | NM_004072.1 | 1240 | Hs.506659 |
| CMKLR1 | 210659_at | NM_004072 | 1240 | Hs.506659 |
| CMKOR1 | 1559114_a_at | NM_020311 | 57007 | Hs.231853 |
| CMKOR1 | 212977_at | NM_020311.1 | 57007 | Hs.471751 |
| CMTM1 | 231219_at | NM_181271.1 | 113540 | Hs.558593 |
| CMTM1 | 1555738_at | NM_181289.1 | 113540 | Hs.558593 |
| CMTM2 | 229967_at | NM_144673.2 | 146225 | Hs.195685 |
| CMTM3 | 224733_at | NM_144601.2 | 123920 | Hs.298198 |
| CMTM3 | 1555704_at | AY166714.1 | 123920 | Hs.298198 |
| CMTM3 | 1555705_a_at | NM_181555.1 | 123920 | Hs.298198 |
| CMTM4 | 224998_at | NM_181521.2 | 146223 | Hs.325825 |
| CMTM4 | 225009_at | NM_178818.2 | 146223 | Hs.325825 |
| CMTM4 | 1554677_s_at | NM_181521.2 | 146223 | Hs.325825 |
| CMTM5 | 230942_at | NM_138460.2 | 116173 | Hs.99272 |
| CMTM6 | 217947_at | NM_017801.2 | 54918 | Hs.380627 |
| CMTM6 | 223047_at | NM_017801.2 | 54918 | Hs.380627 |
| CMTM7 | 226017_at | NM_138410.2 | 112616 | Hs.440494 |
| CMTM7 | 1560754_at | AL832450.1 | 112616 | Hs.440494 |
| CMTM8 | 234263_at | AK000115 | 152189 | 154986 |
| CMTM8 | 235099_at | NM_178868.3 | 152189 | Hs.154986 |
| CNDP1 | 223699_at | NM_032649.5 | 84735 | Hs.400613 |
| CNGA1 | 1554840_at | AF547222.1 | 280665 | Hs.1323 |
| CNGA1 | 206417_at | NM_000087.2 | 1259 | Hs.1323 |
| CNGA3 | 207261_at | NM_001298.1 | 1261 | Hs.234785 |
| CNGA4 | 1562273_at | NM_001037329.1 | 1262 | Hs.434618 |
| CNGB1 | 207342_at | NM_001297.1 | 1258 | Hs.147062 |
| CNGB1 | 210769_at | NM_001297 | 1258 | Hs.147062 |
| CNGB1 | 211099_s_at | NM_001297.1 | 1258 | Hs.147062 |
| CNGB3 | 220304_s_at | NM_019098.2 | 54714 | Hs.154433 |
| CNGB3 | 222959_at | NM_019098.2 | 54714 | Hs.154433 |
| CNIH | 201653_at | NM_001009551.1 | 10175 | Hs.294603 |
| CNIH3 | 214841_at | NM_152495.1 | 149111 | Hs.97338 |
| CNNM1 | 220166_at | NM_020348.1 | 26507 | Hs.274579 |
| CNNM2 | 1554522_at | NM_199077.1 | 54805 | Hs.500903 |
| CNNM2 | 1554523_a_at | NM_199076.1 | 54805 | Hs.500903 |
| CNNM2 | 206818_s_at | NM_199076.1 | 54805 | Hs.500903 |
| CNNM2 | 209874_x_at | NM_017649.3 | 54805 | Hs.500903 |
| CNNM3 | 220739_s_at | NM_017623.4 | 26505 | Hs.150895 |
| CNNM3 | 229031_at | NM_017623 | 26505 | Hs.414042 |
| CNNM3 | 240339_at | NM_017623 | 26505 | Hs.414042 |
| CNNM4 | 218900_at | NM_020184.3 | 26504 | Hs.175043 |
| CNR1 | 213436_at | NM_016083.3 | 1268 | Hs.75110 |
| CNR1 | 1560225_at | NM_001840 | 1268 | Hs.75110 |
| CNR1 | 207940_x_at | NM_016083.3 | 1268 | Hs.75110 |
| CNR1 | 208243_s_at | NM_016083.3 | 1268 | Hs.75110 |
| CNR2 | 206586_at | NM_001841.1 | 1269 | Hs.73037 |
| CNTFR | 205723_at | NM_001842.3 | 1271 | Hs.129966 |
| CNTN1 | 227202_at | NM_001843 | 1272 | Hs.143434 |
| CNTN1 | 227209_at | NM_001843 | 1272 | Hs.143434 |
| CNTN1 | 1554784_at | NM_001843 | 1272 | Hs.143434 |
| CNTN1 | 211203_s_at | NM_175038.1 | 1272 | Hs.143434 |
| CNTN2 | 230045_at | NM_005076.2 | 6900 | Hs.519220 |
| CNTN2 | 206970_at | NM_005076.2 | 6900 | Hs.519220 |
| CNTN3 | 229831_at | NM_020872.1 | 5067 | Hs.12723 |
| CNTN4 | 229084_at | NM_175607.1 | 152330 | Hs.298705 |
| CNTN4 | 237177_at | NM_175613.1 | 152330 | Hs.298705 |
| CNTN5 | 244632_at | NM_014361 | 53942 | Hs.234790 |
| CNTN5 | 207452_s_at | NM_175566.1 | 53942 | Hs.268707 |
| CNTN6 | 207195_at | NM_014461.2 | 27255 | Hs.387300 |
| CNTNAP1 | 219400_at | NM_003632.1 | 8506 | Hs.408730 |
| CNTNAP2 | 215145_s_at | NM_014141.4 | 26047 | Hs.567264 |
| CNTNAP2 | 219300_s_at | NM_014141.4 | 26047 | Hs.567264 |
| CNTNAP2 | 219301_s_at | NM_014141.4 | 26047 | Hs.567264 |
| CNTNAP2 | 219302_s_at | NM_014141.4 | 26047 | Hs.567264 |
| CNTNAP3 | 223796_at | NM_033655.2 | 79937 | Hs.128474 |
| CNTNAP3 | 233202_at | AB051501.2 | 79937 | Hs.128474 |
| CNTNAP3B | 220436_at | XM_497089.2 | 389734 | Hs.130635 |
| CNTNAP4 | 232388_at | AK054786.1 | 85445 | Hs.461389 |
| CNTNAP4 | 1553441_at | NM_138994.1 | 85445 | Hs.461389 |
| CNTNAP4 | 1553442_a_at | NM_033401.2 | 85445 | Hs.461389 |
| CNTNAP4 | 1554377_a_at | NM_033401.2 | 85445 | Hs.461389 |
| CNTNAP5 | 1553013_at | NM_130773.2 | 129684 | Hs.21975 |
| CNTNAP5 | 1553418_a_at | NM_130773.2 | 129684 | Hs.21975 |
| COCH | 1554241_at | NM_004086 | 1690 | Hs.21016 |
| COCH | 1554242_a_at | NM_004086.1 | 1690 | Hs.21016 |
| COCH | 205229_s_at | NM_004086.1 | 1690 | Hs.21016 |
| COL10A1 | 217428_s_at | X98568 | 1300 | 520339 |
| COL10A1 | 205941_s_at | NM_000493 | 1300 | Hs.179729 |
| COL11A1 | 229271_x_at | NM_001854 | 1301 | Hs.439168 |
| COL11A1 | 37892_at | NM_001854 | 1301 | Hs.523446 |
| COL11A1 | 204320_at | NM_001854 | 1301 | Hs.523446 |
| COL11A2 | 213870_at | NM_080679.1 | 1302 | Hs.390171 |
| COL11A2 | 216993_s_at | NM_080679.1 | 1302 | Hs.390171 |
| COL12A1 | 225664_at | NM_004370.5 | 1303 | Hs.101302 |
| COL12A1 | 231766_s_at | NM_080645.2 | 1303 | Hs.101302 |
| COL12A1 | 231879_at | NM_004370.5 | 1303 | Hs.101302 |
| COL12A1 | 233109_at | NM_004370 | 1303 | Hs.101302 |
| COL12A1 | 234951_s_at | NM_004370.5 | 1303 | Hs.101302 |
| COL13A1 | 208535_x_at | NM_005203 | 1305 | Hs.211933 |
| COL13A1 | 211343_s_at | NM_005203.3 | 1305 | Hs.211933 |
| COL13A1 | 211809_x_at | NM_080815.2 | 1305 | Hs.211933 |
| COL14A1 | 216865_at | M64108 | 7373 | Hs.409662 |
| COL14A1 | 216866_s_at | NM_021110.1 | 7373 | Hs.409662 |
| COL14A1 | 212865_s_at | NM_021110.1 | 7373 | Hs.409662 |
| COL15A1 | 203477_at | NM_001855.2 | 1306 | Hs.409034 |
| COL16A1 | 204345_at | NM_001856 | 1307 | Hs.368921 |
| COL17A1 | 204636_at | NM_130778.1 | 1308 | Hs.117938 |
| COL18A1 | 1555952_at | NM_016214 | 80781 | Hs.413175 |
| COL18A1 | 1555953_at | NM_016214 | 80781 | Hs.517356 |
| COL18A1 | 1568732_at | NM_030582 | 80781 | 517356 |
| COL18A1 | 209081_s_at | NM_030582.2 | 80781 | Hs.517356 |
| COL18A1 | 209082_s_at | NM_030582.2 | 80781 | Hs.517356 |
| COL19A1 | 211011_at | NM_001858.4 | 1310 | Hs.444842 |
| COL1A1 | 217430_x_at | AB209597.1 | 1277 | Hs.172928 |
| COL1A1 | 1556499_s_at | NM_000088 | 1277 | Hs.172928 |
| COL1A1 | 202310_s_at | NM_000088 | 1277 | Hs.172928 |
| COL1A1 | 202311_s_at | NM_000088 | 1277 | Hs.172928 |
| COL1A1 | 202312_s_at | NM_000088 | 1277 | Hs.172928 |
| COL1A2 | 229218_at | NM_000089 | 1278 | Hs.232115 |
| COL1A2 | 202403_s_at | NM_000089.3 | 1278 | Hs.489142 |
| COL1A2 | 202404_s_at | NM_000089.3 | 1278 | Hs.489142 |
| COL20A1 | 232638_at | AB040943.1 | 57642 | Hs.271285 |
| COL20A1 | 232733_s_at | NM_020882.1 | 57642 | Hs.271285 |
| COL21A1 | 208096_s_at | NM_030820.3 | 81578 | Hs.47629 |
| COL23A1 | 229168_at | NM_173465.2 | 91522 | Hs.413494 |
| COL24A1 | 238732_at | NM_152890.4 | 255631 | Hs.47312 |
| COL25A1 | 224388_s_at | NM_032518.2 | 84570 | Hs.112925 |
| COL25A1 | 224389_s_at | NM_032518.2 | 84570 | Hs.112925 |
| COL25A1 | 1555253_at | NM_032518 | 84570 | Hs.112925 |
| COL27A1 | 225288_at | NM_032888 | 85301 | Hs.59892 |
| COL27A1 | 225292_at | NM_032888 | 85301 | Hs.494892 |
| COL27A1 | 225293_at | NM_032888 | 85301 | Hs.494892 |
| COL27A1 | 230603_at | NM_032888.2 | 85301 | Hs.494892 |
| COL27A1 | 237452_at | NM_032888 | 85301 | Hs.59892 |
| COL2A1 | 213492_at | NM_001844.3 | 1280 | Hs.408182 |
| COL2A1 | 217404_s_at | NM_033150.1 | 1280 | Hs.408182 |
| COL3A1 | 215076_s_at | NM_000090 | 1281 | Hs.443625 |
| COL3A1 | 215077_at | NM_000090 | 1281 | Hs.443625 |
| COL3A1 | 201852_x_at | NM_000090 | 1281 | Hs.443625 |
| COL3A1 | 211161_s_at | X14420.1 | 1281 | Hs.443625 |
| COL4A1 | 211980_at | NM_001845.3 | 1282 | Hs.17441 |
| COL4A1 | 211981_at | NM_001845.3 | 1282 | Hs.17441 |
| COL4A2 | 211964_at | NM_001846.1 | 1284 | Hs.508716 |
| COL4A2 | 211966_at | NM_001846.1 | 1284 | Hs.508716 |
| COL4A3 | 214641_at | NM_000091.3 | 1285 | Hs.471525 |
| COL4A3 | 216893_s_at | NM_031362.2 | 1285 | Hs.471525 |
| COL4A3 | 216898_s_at | NM_000091.3 | 1285 | Hs.471525 |
| COL4A3 | 222073_at | NM_000091.3 | 1285 | Hs.471525 |
| COL4A4 | 214602_at | NM_000092.3 | 1286 | Hs.282938 |
| COL4A4 | 241565_at | NM_000092 | 1286 | Hs.282938 |
| COL4A5 | 234387_at | NM_033380.1 | 1287 | Hs.369089 |
| COL4A5 | 1563536_at | NM_000495 | 1287 | 369089 |
| COL4A5 | 213110_s_at | NM_000495.3 | 1287 | Hs.369089 |
| COL4A6 | 213992_at | NM_001847.1 | 1288 | Hs.145586 |
| COL4A6 | 1564654_at | NM_001847 | 1288 | 145586 |
| COL4A6 | 210945_at | NM_001847 | 1288 | Hs.145586 |
| COL4A6 | 211473_s_at | NM_033641.1 | 1288 | Hs.145586 |
| COL5A1 | 203325_s_at | NM_000093.3 | 1289 | Hs.210283 |
| COL5A1 | 212488_at | NM_000093.3 | 1289 | Hs.210283 |
| COL5A1 | 212489_at | NM_000093.3 | 1289 | Hs.210283 |
| COL5A2 | 221729_at | NM_000393.3 | 1290 | Hs.445827 |
| COL5A2 | 221730_at | NM_000393.3 | 1290 | Hs.445827 |
| COL5A3 | 218975_at | NM_015719.2 | 50509 | Hs.235368 |
| COL5A3 | 52255_s_at | NM_015719.2 | 50509 | Hs.235368 |
| COL5A3 | 1562979_at | NM_015719 | 50509 | Hs.235368 |
| COL6A1 | 213428_s_at | NM_001848.2 | 1291 | Hs.474053 |
| COL6A1 | 212091_s_at | NM_001848.2 | 1291 | Hs.474053 |
| COL6A1 | 212937_s_at | NM_001848.2 | 1291 | Hs.474053 |
| COL6A1 | 212938_at | NM_001848.2 | 1291 | Hs.474053 |
| COL6A1 | 212940_at | NM_001848.2 | 1291 | Hs.474053 |
| COL6A2 | 213290_at | NM_058175.1 | 1292 | Hs.420269 |
| COL6A2 | 209156_s_at | NM_001849.2 | 1292 | Hs.420269 |
| COL6A3 | 201438_at | NM_057166.2 | 1293 | Hs.233240 |
| COL7A1 | 217312_s_at | NM_000094.2 | 1294 | Hs.476218 |
| COL7A1 | 204136_at | NM_000094.2 | 1294 | Hs.476218 |
| COL8A1 | 214587_at | NM_001850 | 1295 | Hs.114599 |
| COL8A1 | 221152_at | NM_001850 | 1295 | Hs.134830 |
| COL8A1 | 226237_at | NM_001850 | 1295 | Hs.114599 |
| COL8A2 | 221900_at | NM_005202.1 | 1296 | Hs.353001 |
| COL8A2 | 52651_at | NM_005202.1 | 1296 | Hs.353001 |
| COL9A1 | 222008_at | NM_078485.2 | 1297 | Hs.149809 |
| COL9A1 | 243932_at | NM_001851 | 1297 | Hs.149809 |
| COL9A1 | 1555527_at | NM_001851 | 1297 | Hs.149809 |
| COL9A2 | 213622_at | NM_001852.3 | 1298 | Hs.418012 |
| COL9A2 | 232542_at | NM_001852 | 1298 | Hs.418012 |
| COL9A3 | 237427_at | NM_001853 | 1299 | Hs.126248 |
| COL9A3 | 204724_s_at | NM_001853.2 | 1299 | Hs.126248 |
| COLEC10 | 207420_at | NM_006438.2 | 10584 | Hs.176615 |
| COLEC11 | 219873_at | NM_024027.3 | 78989 | Hs.32603 |
| COLEC12 | 221019_s_at | NM_130386.1 | 81035 | Hs.464422 |
| COLQ | 206073_at | NM_080540.2 | 8292 | Hs.146735 |
| COMP | 205713_s_at | NM_000095.2 | 1311 | Hs.1584 |
| COMT | 213981_at | AK130031.1 | 1312 | Hs.370408 |
| COMT | 208817_at | NM_000754.2 | 1312 | Hs.370408 |
| COMT | 208818_s_at | NM_000754.2 | 1312 | Hs.370408 |
| COMTD1 | 226870_at | NM_144589.2 | 118881 | Hs.355333 |
| COPA | 214336_s_at | NM_004371.2 | 1314 | Hs.162121 |
| COPA | 214337_at | NM_004371 | 1314 | Hs.162121 |
| COPA | 1559862_at | NM_004371 | 1314 | Hs.162121 |
| COPA | 208684_at | NM_004371.2 | 1314 | Hs.162121 |
| CORIN | 220356_at | NM_006587.2 | 10699 | Hs.518618 |
| CORIN | 239260_at | NM_006587 | 10699 | Hs.518618 |
| CORIN | 239261_s_at | NM_006587.2 | 10699 | Hs.518618 |
| CORT | 210182_at | NM_001302.3 | 1325 | Hs.412311 |
| COVA1 | 204643_s_at | NM_006375.2 | 10495 | Hs.171458 |
| COVA1 | 204644_at | NM_182314.1 | 10495 | Hs.171458 |
| COVA1 | 32042_at | NM_182314.1 | 10495 | Hs.171458 |
| CP | 214282_at | NM_000096 | 1356 | Hs.282557 |
| CP | 228143_at | NM_000096 | 1356 | Hs.282557 |
| CP | 1558034_s_at | NM_000096.1 | 1356 | Hs.554736 |
| CP | 204846_at | NM_000096.1 | 1356 | Hs.554736 |
| CPA1 | 205615_at | NM_001868.1 | 1357 | Hs.2879 |
| CPA2 | 206212_at | NM_001869.1 | 1358 | Hs.490038 |
| CPA4 | 205832_at | NM_016352 | 51200 | Hs.93764 |
| CPA5 | 240744_at | NM_080385 | 93979 | Hs.436058 |
| CPA6 | 224028_at | NM_020361 | 57094 | Hs.133085 |
| CPA6 | 1552511_a_at | NM_020361.2 | 57094 | Hs.133085 |
| CPAMD8 | 227721_at | NM_015692.1 | 27151 | Hs.529075 |
| CPB1 | 205509_at | NM_001871.2 | 1360 | Hs.477891 |
| CPB2 | 206651_s_at | NM_001872.2 | 1361 | Hs.512937 |
| CPE | 201116_s_at | NM_001873.1 | 1363 | Hs.75360 |
| CPE | 201117_s_at | NM_001873.1 | 1363 | Hs.75360 |
| CPLX3 | 219775_s_at | NM_001030005.2 | 594855 | Hs.187694 |
| CPLX3 | 222927_s_at | NM_001030005.2 | 594855 | Hs.187694 |
| CPM | 217557_s_at | NM_001874 | 1368 | Hs.334873 |
| CPM | 235019_at | NM_001005502.1 | 1368 | Hs.484551 |
| CPM | 235706_at | NM_001005502.1 | 1368 | Hs.484551 |
| CPM | 241765_at | NM_001005502.1 | 1368 | Hs.484551 |
| CPM | 243403_x_at | NM_001005502.1 | 1368 | Hs.484551 |
| CPM | 206100_at | NM_001005502.1 | 1368 | Hs.484551 |
| CPN1 | 206256_at | NM_001308.1 | 1369 | Hs.2246 |
| CPN2 | 216223_at | J05158 | 1370 | Hs.528368 |
| CPO | 1553506_at | NM_173077.1 | 130749 | Hs.218011 |
| CPXM | 227860_at | NM_019609.3 | 56265 | Hs.532685 |
| CPXM2 | 226824_at | NM_198148.1 | 119587 | Hs.307574 |
| CPXM2 | 236144_at | NM_198148.1 | 119587 | Hs.307574 |
| CPXM2 | 1570026_at | BC027623.1 | 119587 | Hs.307574 |
| CPZ | 211062_s_at | NM_001014447.1 | 8532 | Hs.78068 |
| CR1 | 217484_at | NM_000573 | 1378 | Hs.334019 |
| CR1 | 217552_x_at | NM_000651.4 | 1378 | Hs.334019 |
| CR1 | 239205_s_at | NM_000573.3 | 1378 | Hs.334019 |
| CR1 | 244313_at | NM_000573.3 | 1378 | Hs.334019 |
| CR1 | 206244_at | NM_000573.3 | 1378 | Hs.334019 |
| CR1 | 208488_s_at | NM_000573.3 | 1378 | Hs.334019 |
| CR1L | 239206_at | XM_936931.1 | 1379 | Hs.149414 |
| CR2 | 205544_s_at | NM_001006658.1 | 1380 | Hs.445757 |
| CRB1 | 220522_at | NM_201253.1 | 23418 | Hs.126135 |
| CRB1 | 244403_at | NM_201253.1 | 23418 | Hs.126135 |
| CRB2 | 1552506_at | NM_173689.4 | 286204 | Hs.546538 |
| CRB3 | 232609_at | NM_174881.2 | 92359 | Hs.150319 |
| CREBL1 | 1554487_a_at | NM_004381.3 | 1388 | Hs.42853 |
| CREBL1 | 203168_at | NM_004381.3 | 1388 | Hs.42853 |
| CREBL1 | 216208_s_at | NM_004381.3 | 1388 | Hs.42853 |
| CREG1 | 201200_at | NM_003851.2 | 8804 | Hs.5710 |
| CRELD1 | 203368_at | NM_015513.2 | 78987 | Hs.9383 |
| CRELD2 | 218358_at | NM_024324.2 | 79174 | Hs.211282 |
| CRH | 205629_s_at | NM_000756.1 | 1392 | Hs.75294 |
| CRH | 205630_at | NM_000756.1 | 1392 | Hs.75294 |
| CRHR1 | 214619_at | NM_004382.2 | 1394 | Hs.417628 |
| CRHR1 | 208593_x_at | NM_004382.2 | 1394 | Hs.417628 |
| CRHR1 | 211897_s_at | NM_004382.2 | 1394 | Hs.417628 |
| CRHR2 | 207897_at | NM_001883.2 | 1395 | Hs.546246 |
| CRHR2 | 211510_s_at | NM_001883.2 | 1395 | Hs.561726 |
| CRIM1 | 228496_s_at | NM_016441 | 51232 | Hs.170752 |
| CRIM1 | 1559332_at | NM_016441 | 51232 | Hs.170752 |
| CRIM1 | 202551_s_at | NM_016441.1 | 51232 | Hs.332847 |
| CRIM1 | 202552_s_at | NM_016441.1 | 51232 | Hs.332847 |
| CRIM2 | 1557621_at | AK095464.1 | 375616 | Hs.443744 |
| CRISP1 | 216314_at | NM_001131.2 | 167 | Hs.109620 |
| CRISP1 | 207032_s_at | NM_001131.2 | 167 | Hs.109620 |
| CRISP2 | 210262_at | NM_003296.1 | 7180 | Hs.154163 |
| CRISP3 | 207802_at | NM_006061.1 | 10321 | Hs.404466 |
| CRISPLD1 | 223475_at | NM_031461.3 | 83690 | Hs.436542 |
| CRISPLD2 | 221541_at | NM_031476.1 | 83716 | Hs.513779 |
| CRISPLD2 | 1555809_at | BC007689.2 | 83716 | Hs.513779 |
| CRLF1 | 206315_at | NM_004750.2 | 9244 | Hs.114948 |
| CRLF2 | 208303_s_at | NM_022148.2 | 64109 | Hs.287729 |
| CRLF3 | 235803_at | NM_015986 | 51379 | Hs.213016 |
| CRLF3 | 205474_at | NM_015986.2 | 51379 | Hs.567280 |
| CRP | 37020_at | NM_000567.2 | 1401 | Hs.76452 |
| CRP | 205753_at | NM_000567.2 | 1401 | Hs.76452 |
| CRR9 | 229416_at | NM_030782 | 81037 | Hs.444673 |
| CRTAC1 | 221204_s_at | NM_018058.4 | 55118 | Hs.500736 |
| CRTAC1 | 1555958_at | NM_018058 | 55118 | Hs.500736 |
| CRTAM | 206914_at | NM_019604.2 | 56253 | Hs.159523 |
| CRTAP | 226656_at | NM_006371.3 | 10491 | Hs.517888 |
| CRTAP | 227138_at | NM_006371.3 | 10491 | Hs.517888 |
| CRTAP | 1554464_a_at | NM_006371.3 | 10491 | Hs.517888 |
| CRTAP | 1555889_a_at | NM_006371.3 | 10491 | Hs.517888 |
| CRTAP | 201380_at | NM_006371.3 | 10491 | Hs.517888 |
| CSEN | 1555694_a_at | NM_013434.4 | 30818 | Hs.437376 |
| CSEN | 1569355_at | NM_013434 | 30818 | Hs.306828 |
| CSEN | 228269_x_at | NM_001034914.1 | 30818 | Hs.437376 |
| CSEN | 231774_at | NM_001034914.1 | 30818 | Hs.437376 |
| CSEN | 233688_at | NM_013434 | 30818 | Hs.437376 |
| CSEN | 234198_at | NM_013434 | 30818 | Hs.306828 |
| CSEN | 234647_at | NM_013434 | 30818 | Hs.306828 |
| CSF1 | 207082_at | NM_172212.1 | 1435 | Hs.173894 |
| CSF1 | 209716_at | NM_000757 | 1435 | Hs.173894 |
| CSF1 | 210557_x_at | NM_000757.3 | 1435 | Hs.173894 |
| CSF1 | 211839_s_at | NM_000757.3 | 1435 | Hs.173894 |
| CSF1R | 203104_at | NM_005211.2 | 1436 | Hs.483829 |
| CSF2 | 210229_s_at | NM_000758.2 | 1437 | Hs.1349 |
| CSF2RA | 207085_x_at | NM_006140.3 | 1438 | Hs.520937 |
| CSF2RA | 210340_s_at | NM_006140.3 | 1438 | Hs.520937 |
| CSF2RA | 211286_x_at | NM_006140.3 | 1438 | Hs.520937 |
| CSF2RA | 211287_x_at | NM_006140 | 1438 | Hs.520937 |
| CSF2RB | 205159_at | NM_000395 | 1439 | Hs.285401 |
| CSF3 | 207442_at | NM_172219.1 | 1440 | Hs.2233 |
| CSF3R | 1553297_a_at | NM_000760.2 | 1441 | Hs.524517 |
| CSF3R | 203591_s_at | NM_000760.2 | 1441 | Hs.524517 |
| CSH1 | 203807_x_at | NM_001317.3 | 1442 | Hs.347963 |
| CSH1 | 206475_x_at | NM_022640.2 | 1442 | Hs.347963 |
| CSH1 | 208341_x_at | NM_001317.3 | 1442 | Hs.347963 |
| CSH1 | 208342_x_at | NM_001317.3 | 1442 | Hs.347963 |
| CSH1 | 208356_x_at | NM_001317.3 | 1442 | Hs.347963 |
| CSHL1 | 205958_x_at | NM_001318.2 | 1444 | Hs.447490 |
| CSHL1 | 207285_x_at | NM_022580.1 | 1444 | Hs.447490 |
| CSHL1 | 208293_x_at | NM_001318.2 | 1444 | Hs.447490 |
| CSHL1 | 208294_x_at | NM_001318.2 | 1444 | Hs.447490 |
| CSHL1 | 208295_x_at | NM_001318.2 | 1444 | Hs.447490 |
| CSK | 202329_at | NM_004383.1 | 1445 | Hs.77793 |
| CSMD1 | 241960_at | NM_033225 | 64478 | Hs.415199 |
| CSMD1 | 1553405_a_at | NM_033225.3 | 64478 | Hs.518774 |
| CSMD1 | 1562329_at | NM_033225 | 64478 | Hs.518774 |
| CSMD1 | 1562330_s_at | NM_033225.3 | 64478 | Hs.518774 |
| CSMD2 | 233145_at | NM_052896 | 114784 | Hs.127736 |
| CSMD2 | 1557143_at | NM_052896.3 | 114784 | Hs.556009 |
| CSMD2 | 1570169_at | BC031871.2 | 114784 | Hs.127736 |
| CSMD3 | 240228_at | NM_052900.2 | 114788 | Hs.91381 |
| CSN1S1 | 208350_at | NM_001025104.1 | 1446 | Hs.3155 |
| CSN2 | 207951_at | NM_001891.1 | 1447 | Hs.2242 |
| CSN3 | 207803_s_at | NM_005212.1 | 1448 | Hs.54415 |
| CSPG2 | 204619_s_at | NM_004385 | 1462 | Hs.434488 |
| CSPG2 | 204620_s_at | NM_004385.2 | 1462 | Hs.443681 |
| CSPG2 | 211571_s_at | NM_004385.2 | 1462 | Hs.443681 |
| CSPG2 | 215646_s_at | NM_004385.2 | 1462 | Hs.443681 |
| CSPG2 | 221731_x_at | NM_004385 | 1462 | Hs.443681 |
| CSPG3 | 205143_at | NM_004386.1 | 1463 | Hs.169047 |
| CSPG4 | 214297_at | NM_001897 | 1464 | Hs.436301 |
| CSPG4 | 204736_s_at | NM_001897.3 | 1464 | Hs.513044 |
| CSPG4 | 211461_at | NR_002194.1 | 84664 |  |
| CSPG5 | 39966_at | NM_006574.2 | 10675 | Hs.45127 |
| CSPG5 | 205344_at | NM_006574.2 | 10675 | Hs.45127 |
| CST1 | 206224_at | NM_001898.2 | 1469 | Hs.123114 |
| CST2 | 208555_x_at | NM_001322.2 | 1470 | Hs.516939 |
| CST3 | 201360_at | NM_000099.2 | 1471 | Hs.304682 |
| CST4 | 206994_at | NM_001899.2 | 1472 | Hs.56319 |
| CST5 | 207925_at | NM_001900.4 | 1473 | Hs.121489 |
| CST6 | 231248_at | NM_001323 | 1474 | Hs.139389 |
| CST6 | 206595_at | NM_001323.2 | 1474 | Hs.139389 |
| CST7 | 210140_at | NM_003650.2 | 8530 | Hs.143212 |
| CST8 | 220627_at | NM_005492.2 | 10047 | Hs.121602 |
| CST9 | 1554386_at | NM_001008693.1 | 128822 | Hs.558623 |
| CST9L | 230829_at | NM_080610.1 | 128821 | Hs.121554 |
| CSTL1 | 234803_at | NM_138283.1 | 128817 | Hs.352134 |
| CTAG2 | 215733_x_at | NM_020994.2 | 30848 | Hs.87225 |
| CTAG2 | 207337_at | NM_020994.2 | 30848 | Hs.87225 |
| CTAGE1 | 220957_at | NM_172241.2 | 64693 | Hs.406709 |
| CTF1 | 206813_at | NM_001330.2 | 1489 | Hs.483811 |
| CTGF | 209101_at | NM_001901 | 1490 | Hs.410037 |
| CTHRC1 | 225681_at | NM_138455.2 | 115908 | Hs.405614 |
| CTLA4 | 221331_x_at | NM_005214.3 | 1493 | Hs.247824 |
| CTLA4 | 231794_at | NM_001037631.1 | 1493 | Hs.247824 |
| CTLA4 | 234362_s_at | NM_005214.3 | 1493 | Hs.247824 |
| CTLA4 | 234895_at | NM_005214 | 1493 | Hs.247824 |
| CTLA4 | 236341_at | NM_001037631.1 | 1493 | Hs.247824 |
| CTRB2 | 214411_x_at | NM_001025200.2 | 440387 | Hs.74502 |
| CTRB2 | 205971_s_at | NM_001025200.2 | 440387 | Hs.74502 |
| CTRL | 214377_s_at | NM_001907.1 | 1506 | Hs.405774 |
| CTSB | 213274_s_at | NM_001908.3 | 1508 | Hs.520898 |
| CTSB | 213275_x_at | NM_147780.2 | 1508 | Hs.520898 |
| CTSB | 227961_at | NM_001908.3 | 1508 | Hs.520898 |
| CTSB | 243157_at |  | 1508 | Hs.135226 |
| CTSB | 200838_at | NM_001908.3 | 1508 | Hs.520898 |
| CTSB | 200839_s_at | NM_001908.3 | 1508 | Hs.520898 |
| CTSG | 205653_at | NM_001911.2 | 1511 | Hs.421724 |
| CTTNBP2 | 232136_s_at | NM_033427.2 | 83992 | Hs.335033 |
| CTXN1 | 228126_x_at | NM_206833.1 | 404217 | Hs.250879 |
| CUBN | 206775_at | NM_001081.2 | 8029 | Hs.434365 |
| CUZD1 | 220275_at | NM_022034.3 | 50624 | Hs.114648 |
| CX3CL1 | 823_at | NM_002996.3 | 6376 | Hs.531668 |
| CX3CL1 | 203687_at | NM_002996.3 | 6376 | Hs.531668 |
| CX3CR1 | 1568934_at | NM_001337 | 1524 | Hs.78913 |
| CX3CR1 | 205898_at | NM_001337.3 | 1524 | Hs.78913 |
| CXADR | 239155_at | NM_001338 | 1525 | Hs.79187 |
| CXADR | 1555716_a_at | NM_001338.3 | 1525 | Hs.473417 |
| CXADR | 203917_at | NM_001338.3 | 1525 | Hs.473417 |
| CXCL1 | 204470_at | NM_001511.1 | 2919 | Hs.789 |
| CXCL10 | 204533_at | NM_001565.1 | 3627 | Hs.413924 |
| CXCL11 | 210163_at | NM_005409.3 | 6373 | Hs.518814 |
| CXCL11 | 211122_s_at | NM_005409.3 | 6373 | Hs.518814 |
| CXCL12 | 203666_at | NM_000609.4 | 6387 | Hs.522891 |
| CXCL12 | 209687_at | NM_199168.2 | 6387 | Hs.522891 |
| CXCL13 | 205242_at | NM_006419.1 | 10563 | Hs.100431 |
| CXCL14 | 218002_s_at | NM_004887.3 | 9547 | Hs.483444 |
| CXCL14 | 222484_s_at | NM_004887.3 | 9547 | Hs.483444 |
| CXCL14 | 237038_at | NM_004887 | 9547 | Hs.483444 |
| CXCL16 | 223454_at | NM_022059.1 | 58191 | Hs.82407 |
| CXCL17 | 226960_at | NM_198477.1 | 284340 | Hs.445586 |
| CXCL2 | 230101_at | NM_002089 | 2920 | Hs.75765 |
| CXCL2 | 1569203_at | NM_002089 | 2920 | Hs.75765 |
| CXCL2 | 209774_x_at | NM_002089.1 | 2920 | Hs.75765 |
| CXCL3 | 207850_at | NM_002090.2 | 2921 | Hs.89690 |
| CXCL5 | 214974_x_at | NM_002994.3 | 6374 | Hs.89714 |
| CXCL5 | 215101_s_at | NM_002994.3 | 6374 | Hs.89714 |
| CXCL5 | 207852_at | NM_002994 | 6374 | Hs.89714 |
| CXCL6 | 206336_at | NM_002993.2 | 6372 | Hs.164021 |
| CXCL9 | 203915_at | NM_002416.1 | 4283 | Hs.77367 |
| CXCR3 | 217119_s_at | NM_001504.1 | 2833 | Hs.198252 |
| CXCR3 | 207681_at | NM_001504.1 | 2833 | Hs.198252 |
| CXCR4 | 217028_at | NM_001008540.1 | 7852 | Hs.421986 |
| CXCR4 | 209201_x_at | NM_003467.2 | 7852 | Hs.421986 |
| CXCR4 | 211919_s_at | NM_003467.2 | 7852 | Hs.421986 |
| CXCR6 | 206974_at | NM_006564.1 | 10663 | Hs.34526 |
| CXCR6 | 211469_s_at | NM_006564.1 | 10663 | Hs.34526 |
| CXorf12 | 204340_at | NM_003492.1 | 8269 | Hs.23119 |
| CXorf2 | 207364_at | NM_001586.1 | 1527 | Hs.115365 |
| CXorf59 | 1553466_at | NM_173695.1 | 286464 | Hs.376425 |
| CXorf61 | 1559258_a_at | NM_001017978.1 | 203413 | Hs.97892 |
| CYB561 | 217200_x_at | NM_001017916 | 1534 | 355264 |
| CYB561 | 207986_x_at | NM_001915 | 1534 | Hs.355264 |
| CYB561 | 209163_at | NM_001017916.1 | 1534 | Hs.355264 |
| CYB561 | 209164_s_at | NM_001017916.1 | 1534 | Hs.355264 |
| CYB561 | 210816_s_at | NM_001017916.1 | 1534 | Hs.355264 |
| CYB561D1 | 227981_at | BX647509.1 | 284613 | Hs.514682 |
| CYB561D2 | 209665_at | NM_007022.3 | 11068 | Hs.149443 |
| CYB5D2 | 225804_at | NM_144611.2 | 124936 | Hs.513871 |
| CYBB | 217431_x_at | NM_000397 | 1536 | Hs.292356 |
| CYBB | 233538_s_at | NM_000397 | 1536 | Hs.88974 |
| CYBB | 203922_s_at | NM_000397.2 | 1536 | Hs.292356 |
| CYBB | 203923_s_at | NM_000397.2 | 1536 | Hs.292356 |
| CYBRD1 | 217889_s_at | NM_024843.2 | 79901 | Hs.221941 |
| CYBRD1 | 222453_at | NM_024843.2 | 79901 | Hs.221941 |
| CYFIP2 | 215785_s_at | NM_001037332.1 | 26999 | Hs.519702 |
| CYFIP2 | 220999_s_at | NM_001037332.1 | 26999 | Hs.519702 |
| CYP20A1 | 219565_at | NM_020674.2 | 57404 | Hs.446065 |
| CYP20A1 | 244732_at | NM_020674 | 57404 | Hs.446065 |
| CYP4Z2P | 1553434_at | NR_002788.1 | 163720 | Hs.176588 |
| CYR61 | 201289_at | NM_001554.3 | 3491 | Hs.8867 |
| CYR61 | 210764_s_at | NM_001554.3 | 3491 | Hs.8867 |
| CYSLTR1 | 216288_at | NM_006639 | 10800 | Hs.201300 |
| CYSLTR1 | 230866_at | NM_006639 | 10800 | Hs.201300 |
| CYSLTR1 | 231747_at | NM_006639.2 | 10800 | Hs.201300 |
| CYSLTR2 | 220813_at | NM_020377.2 | 57105 | Hs.253706 |
| CYTL1 | 219837_s_at | NM_018659.2 | 54360 | Hs.13872 |
| CYYR1 | 228665_at | NM_052954.2 | 116159 | Hs.37445 |
| CYYR1 | 235044_at | NM_052954.2 | 116159 | Hs.37445 |
| DAF | 1555950_a_at | NM_000574.2 | 1604 | Hs.527653 |
| DAF | 201925_s_at | NM_000574.2 | 1604 | Hs.527653 |
| DAF | 201926_s_at | NM_000574.2 | 1604 | Hs.527653 |
| DAG1 | 205417_s_at | NM_004393.1 | 1605 | Hs.76111 |
| DAG1 | 212128_s_at | NM_004393.1 | 1605 | Hs.76111 |
| DAGLB | 225828_at | NM_139179.1 | 221955 | Hs.487498 |
| DAGLB | 225832_s_at | NM_139179.1 | 221955 | Hs.487498 |
| DAGLB | 225833_at | NM_139179.1 | 221955 | Hs.487498 |
| DAGLB | 226640_at | NM_139179.1 | 221955 | Hs.487498 |
| DAND5 | 1562772_a_at | NM_152654.2 | 199699 | Hs.331981 |
| DBH | 230488_s_at | AF129263.1 | 138948 | Hs.223858 |
| DBH | 234916_at | NM_000787 | 1621 | Hs.223858 |
| DBH | 206450_at | NM_000787.2 | 1621 | Hs.223858 |
| DC2 | 223001_at | NM_021227.2 | 58505 | Hs.445803 |
| DCBLD1 | 226609_at | NM_173674 | 285761 | Hs.567333 |
| DCBLD1 | 1553768_a_at | NM_173674.1 | 285761 | Hs.567333 |
| DCBLD2 | 213865_at | NM_080927.3 | 131566 | Hs.203691 |
| DCBLD2 | 224911_s_at | NM_080927.3 | 131566 | Hs.203691 |
| DCBLD2 | 239446_x_at | NM_080927.3 | 131566 | Hs.203691 |
| DCC | 206939_at | NM_005215.1 | 1630 | Hs.172562 |
| DCD | 1553946_at | NM_053283.2 | 117159 | Hs.350570 |
| DCHS1 | 218892_at | NM_003737.2 | 8642 | Hs.199850 |
| DCHS1 | 222101_s_at | NM_003737.2 | 8642 | Hs.199850 |
| DCHS2 | 220373_at | AK095103.1 | 54798 | Hs.148847 |
| DCHS2 | 242764_at | NM_017639.2 | 54798 | Hs.148847 |
| DCLK1 | 229800_at | AI129626 |  | Hs.444377 |
| DCN | 240556_at | NM_001920 | 1634 | Hs.156316 |
| DCN | 242605_at | NM_001920 | 1634 | Hs.156316 |
| DCN | 201893_x_at | NM_133505.2 | 1634 | Hs.156316 |
| DCN | 209335_at | NM_001920.3 | 1634 | Hs.156316 |
| DCN | 211813_x_at | NM_001920.3 | 1634 | Hs.156316 |
| DCN | 211896_s_at | NM_133503.2 | 1634 | Hs.156316 |
| DCST1 | 1553860_at | NM_152494.1 | 149095 | Hs.505904 |
| DCST2 | 242988_at | NM_144622 | 127579 | Hs.567314 |
| DCXR | 217973_at | NM_016286.2 | 51181 | Hs.9857 |
| DDC | 214347_s_at | NM_000790.2 | 1644 | Hs.359698 |
| DDC | 1564459_at | NM_000790 | 1644 | Hs.408106 |
| DDC | 205311_at | NM_000790.2 | 1644 | Hs.359698 |
| DDR1 | 1007_s_at | NM_001954.3 | 780 | Hs.520004 |
| DDR1 | 207169_x_at | NM_001954.3 | 780 | Hs.520004 |
| DDR1 | 208779_x_at | NM_001954.3 | 780 | Hs.520004 |
| DDR1 | 210749_x_at | NM_001954.3 | 780 | Hs.520004 |
| DDR2 | 205168_at | NM_001014796.1 | 4921 | Hs.275757 |
| DEAF1 | 230059_at | NM_021008 | 10522 | Hs.6574 |
| DEAF1 | 1559132_at | NM_021008 | 10522 | Hs.6574 |
| DEAF1 | 209407_s_at | NM_021008.2 | 10522 | Hs.243994 |
| DEFA3 | 205033_s_at | NM_005217.2 | 1668 | Hs.294176 |
| DEFA4 | 207269_at | NM_001925.1 | 1669 | Hs.128581 |
| DEFA5 | 207529_at | NM_021010.1 | 1670 | Hs.72887 |
| DEFA6 | 207814_at | NM_001926.2 | 1671 | Hs.711 |
| DEFB1 | 210397_at | NM_005218.3 | 1672 | Hs.32949 |
| DEFB103A | 224239_at | NM_018661.2 | 55894 | Hs.283082 |
| DEFB104A | 1553521_at | XM_928786.1 | 140596 | Hs.559507 |
| DEFB105A | 1553002_at | NM_152250.1 | 245908 | Hs.381378 |
| DEFB106A | 1552411_at | XM_928779.1 | 245909 | Hs.511958 |
| DEFB106A | 1552412_a_at | XM_928779.1 | 245909 | Hs.511958 |
| DEFB107A | 1563450_at | NM_001037668.1 | 245910 | Hs.520874 |
| DEFB114 | 1567878_at | AY122470 | 245928 | Hs.381372 |
| DEFB118 | 233221_at | NM_054112.1 | 117285 | Hs.274124 |
| DEFB119 | 231155_at | NM_153289.2 | 245932 | Hs.516998 |
| DEFB119 | 1553093_a_at | NM_153323.3 | 245932 | Hs.516998 |
| DEFB123 | 229772_at | NM_153324.2 | 245936 | Hs.122509 |
| DEFB124 | 1568375_at | AY122476 | 245937 | Hs.381373 |
| DEFB124 | 1568377_x_at | NM_001037500.1 | 245937 | Hs.381373 |
| DEFB125 | 1552998_at | NM_153325.2 | 245938 | Hs.380220 |
| DEFB126 | 221414_s_at | NM_030931.2 | 81623 | Hs.124211 |
| DEFB126 | 233166_at | NM_030931 | 81623 | Hs.124211 |
| DEFB127 | 233343_at | NM_139074.2 | 140850 | Hs.99362 |
| DEFB129 | 233160_at | NM_080831.2 | 140881 | Hs.112087 |
| DEFB32 | 243311_at | NM_207469.1 | 400830 | Hs.516819 |
| DEFB4 | 207356_at | NM_004942.2 | 1673 | Hs.105924 |
| DEGS1 | 207431_s_at | NM_003676.2 | 8560 | Hs.299878 |
| DEGS1 | 209250_at | NM_003676.2 | 8560 | Hs.299878 |
| DF | 205382_s_at | NM_001928.2 | 1675 | Hs.155597 |
| DGCR2 | 214198_s_at | NM_005137.1 | 9993 | Hs.517357 |
| DGCR2 | 227028_s_at | NM_005137 | 9993 | Hs.415359 |
| DGCR2 | 202099_s_at | NM_005137.1 | 9993 | Hs.517357 |
| DGKE | 1554621_at | NM_003647 | 8526 | Hs.239514 |
| DGKE | 1554623_x_at | NM_003647 | 8526 | Hs.239514 |
| DGKE | 207518_at | NM_003647.1 | 8526 | Hs.239514 |
| DHCR7 | 201790_s_at | NM_001360.1 | 1717 | Hs.503134 |
| DHCR7 | 201791_s_at | NM_001360.1 | 1717 | Hs.503134 |
| DHH | 1552730_at | NM_021044.2 | 50846 | Hs.524382 |
| DHRS3 | 202481_at | NM_004753.4 | 9249 | Hs.289347 |
| DHRS8 | 217989_at | NM_016245.2 | 51170 | Hs.282984 |
| DIO2 | 231240_at | NM_000793 | 1734 | Hs.436020 |
| DIO2 | 203699_s_at | NM_001007023.1 | 1734 | Hs.202354 |
| DIO2 | 203700_s_at | NM_000793 | 1734 | Hs.202354 |
| DIO2 | 210819_x_at | NM_000793 | 1734 | Hs.202354 |
| DIO2 | 211215_x_at | NM_000793.3 | 1734 | Hs.202354 |
| DIO3 | 207154_at | NM_001362.2 | 1735 | Hs.49322 |
| DIRC2 | 226026_at | NM_032839.1 | 84925 | Hs.477346 |
| DISP1 | 228184_at | NM_032890.2 | 84976 | Hs.528817 |
| DISP2 | 229579_s_at | NM_033510.1 | 85455 | Hs.355645 |
| DKFZp313G1735 | 1569132_s_at | BC015723 | 153642 | Hs.151380 |
| DKFZp313G1735 | 1569133_x_at | BC015723 | 153642 | Hs.151380 |
| DKFZp313G1735 | 239147_at | AL832711.1 | 153642 | Hs.163914 |
| DKFZp313M0720 | 240947_at | N36984 | 196527 | Hs.410941 |
| DKFZP434G156 | 226971_at | NM_022742 | 64753 | Hs.7973 |
| DKFZP564D172 | 223882_at | NM_032042 | 83989 | Hs.567308 |
| DKFZP564J0863 | 223452_s_at | NM_015459.3 | 25923 | Hs.356719 |
| DKFZP564J0863 | 223453_s_at | NM_015459 | 25923 | Hs.381206 |
| DKFZp761O2018 | 232313_at | XM_044062.10 | 92293 | Hs.49599 |
| DKFZp762F0713 | 242592_at | XM_290615.5 | 283554 | Hs.416214 |
| DKK1 | 204602_at | NM_012242.2 | 22943 | Hs.40499 |
| DKK2 | 219908_at | NM_014421.2 | 27123 | Hs.211869 |
| DKK2 | 224199_at | NM_014421 | 27123 | Hs.211869 |
| DKK3 | 214247_s_at | NM_001018057.1 | 27122 | Hs.292156 |
| DKK3 | 230508_at | NM_013253 | 27122 | Hs.130865 |
| DKK3 | 202196_s_at | NM_001018057.1 | 27122 | Hs.292156 |
| DKK3 | 207875_at | NM_013253 | 27122 | Hs.130865 |
| DKK4 | 206619_at | NM_014420.2 | 27121 | Hs.159311 |
| DKKL1 | 220284_at | NM_014419.3 | 27120 | Hs.515855 |
| DLG1 | 215988_s_at | NM_004087 | 1739 | Hs.389893 |
| DLG1 | 217208_s_at | NM_004087.1 | 1739 | Hs.292549 |
| DLG1 | 229703_at | NM_004087 | 1739 | Hs.389893 |
| DLG1 | 230229_at | NM_004087 | 1739 | Hs.389893 |
| DLG1 | 202514_at | NM_004087 | 1739 | Hs.292549 |
| DLG1 | 202515_at | NM_004087 | 1739 | Hs.292549 |
| DLG1 | 202516_s_at | NM_004087.1 | 1739 | Hs.292549 |
| DLG2 | 228973_at | NM_001364.2 | 1740 | Hs.503453 |
| DLG2 | 234480_at | AL137340 | 1740 | 503453 |
| DLG2 | 234525_at | AL137340 | 1740 | 503453 |
| DLG2 | 1566947_at | AL831894 | 1740 | 503453 |
| DLG2 | 1566948_at | AL831894 | 1740 | 503453 |
| DLG2 | 206253_at | NM_001364.2 | 1740 | Hs.503453 |
| DLK1 | 209560_s_at | NM_001032997.1 | 8788 | Hs.533717 |
| DLL1 | 224215_s_at | NM_005618.2 | 28514 | Hs.379912 |
| DLL1 | 227938_s_at | NM_005618.2 | 28514 | Hs.379912 |
| DLL3 | 219537_x_at | NM_016941.2 | 10683 | Hs.127792 |
| DLL3 | 222898_s_at | NM_203486.1 | 10683 | Hs.127792 |
| DLL3 | 229755_x_at | NM_016941 | 10683 | Hs.127792 |
| DLL3 | 230568_x_at | NM_016941 | 10683 | Hs.127792 |
| DLL4 | 223525_at | NM_019074.2 | 54567 | Hs.511076 |
| DMBT1 | 208250_s_at | NM_004406.1 | 1755 | Hs.279611 |
| DMKN | 226926_at | NM_033317.2 | 93099 | Hs.417795 |
| DMP1 | 217067_s_at | NM_004407.1 | 1758 | Hs.128556 |
| DMP1 | 208175_s_at | NM_004407.1 | 1758 | Hs.128556 |
| DNAJB14 | 219237_s_at | NM_024920.3 | 79982 | Hs.512743 |
| DNAJB14 | 222850_s_at | NM_001031723.1 | 79982 | Hs.512743 |
| DNAJC10 | 221781_s_at | NM_018981.1 | 54431 | Hs.516632 |
| DNAJC10 | 221782_at | NM_018981.1 | 54431 | Hs.516632 |
| DNAJC10 | 225174_at | NM_018981.1 | 54431 | Hs.516632 |
| DNAJC15 | 218435_at | NM_013238.2 | 29103 | Hs.438830 |
| DNAJC15 | 227808_at | NM_013238.2 | 29103 | Hs.438830 |
| DNAJC16 | 217358_at | BX648982.1 | 23341 | Hs.521764 |
| DNAJC16 | 242947_at | BF447963 | 23341 | Hs.521764 |
| DNAJC16 | 212908_at | NM_015291.2 | 23341 | Hs.521764 |
| DNAJC16 | 212911_at | NM_015291.2 | 23341 | Hs.521764 |
| DNAJC18 | 227166_at | NM_152686.2 | 202052 | Hs.483537 |
| DNAJC18 | 227169_at | NM_152686.2 | 202052 | Hs.483537 |
| DNASE1 | 240144_at | BX648257.1 | 1773 | Hs.30345 |
| DNASE1 | 241854_at | BX648257.1 | 1773 | Hs.558320 |
| DNASE1 | 243127_x_at | BX648257.1 | 1773 | Hs.30345 |
| DNASE1 | 1557189_at | AK095412.1 | 1773 | Hs.558320 |
| DNASE1 | 1558546_at | NM_005223.3 | 1773 | Hs.30345 |
| DNASE1 | 210165_at | NM_005223.3 | 1773 | Hs.30345 |
| DNASE1L2 | 207192_at | NM_001374.2 | 1775 | Hs.103503 |
| DNB5 | 230458_at | XM_937695.1 | 648626 | Hs.128740 |
| DNER | 226281_at | NM_139072.2 | 92737 | Hs.234074 |
| DNHD1 | 229631_at | NM_144666.1 | 144132 | Hs.424183 |
| DO | 207220_at | NM_021071.2 | 420 | Hs.13776 |
| DOC2B | 207311_at | NM_003585.1 | 8447 | Hs.551705 |
| DOK1 | 216835_s_at | NM_001381.2 | 1796 | Hs.103854 |
| DOK1 | 211121_s_at | NM_001381.2 | 1796 | Hs.103854 |
| DOK2 | 214054_at | NM_003974.2 | 9046 | Hs.71215 |
| DOK4 | 207747_s_at | NM_018110.2 | 55715 | Hs.279832 |
| DOK4 | 209690_s_at | NM_018110.2 | 55715 | Hs.279832 |
| DOK4 | 209691_s_at | NM_018110.2 | 55715 | Hs.279832 |
| DOK5 | 214844_s_at | NM_018431.3 | 55816 | Hs.473133 |
| DOK5 | 1554863_s_at | NM_177959.2 | 55816 | Hs.473133 |
| DOK7 | 240633_at | NM_173660.2 | 285489 | Hs.122110 |
| DPCR1 | 231646_at | NM_080870.2 | 135656 | Hs.485071 |
| DPCR1 | 241137_at | NM_080870.2 | 135656 | Hs.485071 |
| DPEP1 | 205983_at | NM_004413.1 | 1800 | Hs.109 |
| DPEP2 | 219452_at | NM_022355.1 | 64174 | Hs.372633 |
| DPEP3 | 220179_at | NM_022357.1 | 64180 | Hs.302028 |
| DPP10 | 228598_at | NM_001004360.2 | 57628 | Hs.176247 |
| DPP4 | 203716_s_at | NM_001935.3 | 1803 | Hs.368912 |
| DPP4 | 203717_at | NM_001935 | 1803 | Hs.368912 |
| DPP4 | 211478_s_at | NM_001935.3 | 1803 | Hs.368912 |
| DPP6 | 228546_at | NM_001039350.1 | 1804 | Hs.490684 |
| DPP6 | 207789_s_at | NM_001039350.1 | 1804 | Hs.490684 |
| DPT | 207977_s_at | NM_001937.3 | 1805 | Hs.80552 |
| DPT | 213068_at | NM_001937.3 | 1805 | Hs.80552 |
| DPT | 213071_at | NM_001937.3 | 1805 | Hs.80552 |
| DPY19L1 | 1560915_at | AJ011911.1 | 23333 | Hs.510645 |
| DPY19L1 | 1560916_a_at | XM_935726.1 | 23333 | Hs.408623 |
| DPY19L1 | 1570355_a_at | AJ011911.1 | 23333 | Hs.408623 |
| DPY19L1 | 212792_at | XM_371891.2 | 23333 | Hs.408623 |
| DPY19L2 | 230158_at | NM_173812.4 | 283417 | Hs.533644 |
| DPY19L2 | 238784_at | NM_173812.4 | 283417 | Hs.533644 |
| DPY19L3 | 225633_at | AK024775.1 | 147991 | Hs.194392 |
| DPY19L4 | 213391_at | NM_181787.1 | 286148 | Hs.165539 |
| DRD1 | 214652_at | NM_000794.3 | 1812 | Hs.2624 |
| DRD1IP | 219896_at | NM_015722.2 | 50632 | Hs.148680 |
| DRD2 | 216924_s_at | NM_016574.2 | 1813 | Hs.73893 |
| DRD2 | 216938_x_at | NM_000795.2 | 1813 | Hs.73893 |
| DRD2 | 206590_x_at | NM_000795.2 | 1813 | Hs.73893 |
| DRD2 | 211624_s_at | NM_000795.2 | 1813 | Hs.73893 |
| DRD3 | 214559_at | NM_000796 | 1814 | Hs.121478 |
| DRD3 | 211625_s_at | NM_000796.3 | 1814 | Hs.121478 |
| DRD4 | 208215_x_at | NM_000797.2 | 1815 | Hs.99922 |
| DRD5 | 208486_at | NM_000798.3 | 1816 | Hs.380681 |
| DSC1 | 1556333_at | NM_004948 | 1823 | Hs.348436 |
| DSC1 | 1556334_s_at | NM_004948 | 1823 | Hs.348436 |
| DSC1 | 207324_s_at | NM_024421.1 | 1823 | Hs.558154 |
| DSC2 | 226817_at | NM_004949.2 | 1824 | Hs.95612 |
| DSC2 | 204750_s_at | NM_004949.2 | 1824 | Hs.95612 |
| DSC2 | 204751_x_at | NM_004949.2 | 1824 | Hs.95612 |
| DSC3 | 244107_at | AW189097 | 1825 | Hs.41690 |
| DSC3 | 206032_at | NM_001941 | 1825 | Hs.41690 |
| DSC3 | 206033_s_at | NM_001941.2 | 1825 | Hs.41690 |
| DSCAM | 237268_at | NM_206887.1 | 1826 | Hs.397800 |
| DSCAM | 211484_s_at | NM_001389.3 | 1826 | Hs.397800 |
| DSCAML1 | 232059_at | NM_020693.2 | 57453 | Hs.137183 |
| DSCAML1 | 234908_s_at | NM_020693.2 | 57453 | Hs.137183 |
| DSG1 | 206642_at | NM_001942.1 | 1828 | Hs.2633 |
| DSG2 | 217901_at | NM_001943 | 1829 | Hs.412597 |
| DSG2 | 243081_at | NM_001943 | 1829 | Hs.412597 |
| DSG2 | 1553105_s_at | NM_001943.1 | 1829 | Hs.412597 |
| DSG3 | 235075_at | NM_001944 | 1830 | Hs.1925 |
| DSG3 | 205595_at | NM_001944.1 | 1830 | Hs.1925 |
| DSG4 | 1561330_at | NM_177986 | 147409 | Hs.407618 |
| DSPG3 | 206439_at | NM_004950.3 | 1833 | Hs.435680 |
| DSPP | 221681_s_at | NM_014208.2 | 1834 | Hs.447902 |
| DST | 215016_x_at | NM_183380.1 | 667 | Hs.485616 |
| DST | 216918_s_at | NM_001723.3 | 667 | Hs.485616 |
| DST | 220154_at | AF083131.1 | 667 | Hs.485616 |
| DST | 232098_at | AK025142.1 | 667 | Hs.485616 |
| DST | 1553191_at | NM_020388.2 | 667 | Hs.485616 |
| DST | 204455_at | NM_001723.3 | 667 | Hs.485616 |
| DST | 212253_x_at | NM_183380.1 | 667 | Hs.485616 |
| DST | 212254_s_at | NM_015548.2 | 667 | Hs.485616 |
| DUOX1 | 215800_at | NM_017434 | 53905 | Hs.272813 |
| DUOX1 | 219597_s_at | NM_017434.3 | 53905 | Hs.272813 |
| DUOX1 | 1565795_at | NM_017434 | 53905 | Hs.272813 |
| DUOX2 | 219727_at | NM_014080.3 | 50506 | Hs.71377 |
| DUOXA1 | 1554648_a_at | NM_144565 | 90527 | Hs.356664 |
| DUOXA1 | 1555404_a_at | NM_144565.2 | 90527 | Hs.356664 |
| DYSF | 218660_at | NM_003494.2 | 8291 | Hs.252180 |
| EBAG9 | 204274_at | NM_004215.3 | 9166 | Hs.409368 |
| EBAG9 | 204278_s_at | NM_004215.3 | 9166 | Hs.409368 |
| EBI2 | 205419_at | NM_004951.3 | 1880 | Hs.508545 |
| EBI3 | 219424_at | NM_005755.2 | 10148 | Hs.501452 |
| EBP | 213787_s_at | NM_006579.1 | 10682 | Hs.522636 |
| EBP | 202735_at | NM_006579.1 | 10682 | Hs.522636 |
| ECE1 | 201750_s_at | NM_001397.1 | 1889 | Hs.195080 |
| ECEL1 | 219914_at | NM_004826.1 | 9427 | Hs.26880 |
| ECM1 | 209365_s_at | NM_022664.1 | 1893 | Hs.81071 |
| ECM2 | 1568779_a_at | NM_001393 | 1842 | Hs.117060 |
| ECM2 | 206101_at | NM_001393.2 | 1842 | Hs.117060 |
| EDA | 206217_at | NM_001005609.1 | 1896 | Hs.105407 |
| EDA | 211127_x_at | NM_001005614.1 | 1896 | Hs.105407 |
| EDA | 211128_at | NM_001005611.1 | 1896 | Hs.105407 |
| EDA | 211129_x_at | NM_001005612.1 | 1896 | Hs.105407 |
| EDA | 211130_x_at | NM_001005612.1 | 1896 | Hs.105407 |
| EDA | 211131_s_at | NM_001005614.1 | 1896 | Hs.105407 |
| EDA2R | 221399_at | NM_021783.2 | 60401 | Hs.302017 |
| EDAR | 220048_at | NM_022336.1 | 10913 | Hs.171971 |
| EDG1 | 204642_at | NM_001400 | 1901 | Hs.154210 |
| EDG2 | 204036_at | NM_001401.3 | 1902 | Hs.126667 |
| EDG2 | 204037_at | NM_001401 | 1902 | Hs.126667 |
| EDG2 | 204038_s_at | NM_001401.3 | 1902 | Hs.126667 |
| EDG3 | 228176_at | NM_005226.2 | 1903 | Hs.292737 |
| EDG3 | 231741_at | NM_005226.2 | 1903 | Hs.292737 |
| EDG4 | 206722_s_at | NM_004720.4 | 9170 | Hs.122575 |
| EDG4 | 206723_s_at | NM_004720.4 | 9170 | Hs.122575 |
| EDG5 | 208537_at | NM_004230.2 | 9294 | Hs.458474 |
| EDG6 | 206437_at | NM_003775.2 | 8698 | Hs.515061 |
| EDG7 | 220816_at | NM_012152.1 | 23566 | Hs.527909 |
| EDG8 | 221417_x_at | NM_030760.3 | 53637 | Hs.501561 |
| EDG8 | 230464_at | NM_030760.3 | 53637 | Hs.501561 |
| EDG8 | 233743_x_at | NM_030760.3 | 53637 | Hs.501561 |
| EDIL3 | 225275_at | NM_005711 | 10085 | Hs.482730 |
| EDIL3 | 1558643_s_at | NM_005711.3 | 10085 | Hs.482730 |
| EDIL3 | 207379_at | NM_005711.3 | 10085 | Hs.482730 |
| EDN1 | 218995_s_at | NM_001955.2 | 1906 | Hs.511899 |
| EDN1 | 222802_at | NM_001955 | 1906 | Hs.437313 |
| EDN1 | 1564630_at | NM_001955 | 1906 | Hs.511899 |
| EDN2 | 206758_at | NM_001956.2 | 1907 | Hs.1407 |
| EDN3 | 217154_s_at | NM_207032.1 | 1908 | Hs.1408 |
| EDN3 | 208399_s_at | NM_207032.1 | 1908 | Hs.1408 |
| EDNRA | 216235_s_at | NM_001957.1 | 1909 | Hs.183713 |
| EDNRA | 243555_at | NM_001957 | 1909 | Hs.211202 |
| EDNRA | 244832_at | NM_001957 | 1909 | Hs.211202 |
| EDNRA | 204463_s_at | NM_001957.1 | 1909 | Hs.183713 |
| EDNRA | 204464_s_at | NM_001957.1 | 1909 | Hs.183713 |
| EDNRB | 204271_s_at | NM_000115.1 | 1910 | Hs.82002 |
| EDNRB | 204273_at | NM_000115.1 | 1910 | Hs.82002 |
| EDNRB | 206701_x_at | NM_000115.1 | 1910 | Hs.82002 |
| EFEMP1 | 228421_s_at | NM_004105 | 2202 | Hs.76224 |
| EFEMP1 | 201842_s_at | NM_001039348.1 | 2202 | Hs.76224 |
| EFEMP1 | 201843_s_at | NM_004105.3 | 2202 | Hs.76224 |
| EFEMP2 | 206580_s_at | NM_016938.2 | 30008 | Hs.381870 |
| EFEMP2 | 209356_x_at | NM_016938.2 | 30008 | Hs.381870 |
| EFHA2 | 238458_at | NM_181723.1 | 286097 | Hs.403594 |
| EFNA1 | 202023_at | NM_182685.1 | 1942 | Hs.516664 |
| EFNA2 | 1553573_s_at | NM_001405.2 | 1943 | Hs.532655 |
| EFNA2 | 208256_at | NM_001405 | 1943 | Hs.532655 |
| EFNA3 | 210132_at | NM_004952.3 | 1944 | Hs.516656 |
| EFNA4 | 205107_s_at | NM_005227.2 | 1945 | Hs.449913 |
| EFNA5 | 207301_at | NM_001962.1 | 1946 | Hs.128518 |
| EFNB1 | 202711_at | NM_004429.3 | 1947 | Hs.144700 |
| EFNB2 | 202668_at | NM_004093.2 | 1948 | Hs.149239 |
| EFNB2 | 202669_s_at | NM_004093.2 | 1948 | Hs.149239 |
| EFNB3 | 205031_at | NM_001406.3 | 1949 | Hs.26988 |
| EFNB3 | 210883_x_at | NM_001406.3 | 1949 | Hs.26988 |
| EGF | 206254_at | NM_001963.2 | 1950 | Hs.419815 |
| EGFL4 | 214778_at | NM_001410.1 | 1954 | Hs.132483 |
| EGFL5 | 212830_at | W68084 | 1955 | Hs.494977 |
| EGFL5 | 212831_at | BF110421 | 1955 | Hs.494977 |
| EGFL6 | 219454_at | NM_015507.2 | 25975 | Hs.12844 |
| EGFL7 | 218825_at | NM_016215.3 | 51162 | Hs.91481 |
| EGFL8 | 208469_s_at | NM_030652.2 | 80864 | Hs.332138 |
| EGFL8 | 209826_at | NM_030652.2 | 80864 | Hs.332138 |
| EGFL9 | 220262_s_at | NM_206539.1 | 65989 | Hs.337251 |
| EGFLAM | 226911_at | NM_182798.1 | 133584 | Hs.20103 |
| EGFR | 1565483_at | NM_201282.1 | 1956 | Hs.488293 |
| EGFR | 1565484_x_at | NM_201282.1 | 1956 | Hs.488293 |
| EGFR | 201983_s_at | NM_005228.3 | 1956 | Hs.488293 |
| EGFR | 201984_s_at | NM_005228.3 | 1956 | Hs.488293 |
| EGFR | 210984_x_at | NM_005228.3 | 1956 | Hs.488293 |
| EGFR | 211550_at | NM_201284.1 | 1956 | Hs.488293 |
| EGFR | 211551_at | K03193.1 | 1956 | Hs.488293 |
| EGFR | 211607_x_at | NM_005228.3 | 1956 | Hs.488293 |
| EI24 | 216396_s_at | NM_001007277.1 | 9538 | Hs.343911 |
| EI24 | 208289_s_at | NM_004879 | 9538 | Hs.343911 |
| ELA1 | 224198_at | NM_001971.4 | 1990 | Hs.348395 |
| ELA2 | 206871_at | NM_001972.2 | 1991 | Hs.99863 |
| ELA2B | 207077_at | NM_015849.2 | 51032 | Hs.553535 |
| ELA3B | 206151_x_at | NM_007352.2 | 23436 | Hs.181289 |
| ELL | 1564568_at | AL050168 | 8178 | 515260 |
| ELL | 1565254_s_at | NM_006532.2 | 8178 | Hs.515260 |
| ELL | 1569474_at | BC017563 | 8178 | 515260 |
| ELL | 204095_s_at | NM_006532.2 | 8178 | Hs.515260 |
| ELL | 204096_s_at | NM_006532.2 | 8178 | Hs.515260 |
| ELMO1 | 204513_s_at | NM_001039459.1 | 9844 | Hs.304578 |
| ELN | 216269_s_at | NM_000501.1 | 2006 | Hs.252418 |
| ELN | 212670_at | NM_000501 | 2006 | Hs.252418 |
| ELSPBP1 | 220366_at | NM_022142.3 | 64100 | Hs.104894 |
| ELTD1 | 219134_at | NM_022159 | 64123 | Hs.132314 |
| EMB | 226789_at | U52054 |  | Hs.446408 |
| EMCN | 219436_s_at | NM_016242.2 | 51705 | Hs.152913 |
| EMCN | 222885_at | NM_016242.2 | 51705 | Hs.152913 |
| EMCN | 1564134_at | AL833154 | 51705 | 152913 |
| EMID1 | 213779_at | NM_133455.2 | 129080 | Hs.289106 |
| EMID1 | 1564251_at | AL831910.1 | 129080 | Hs.289106 |
| EMID2 | 233894_x_at | NM_133457.1 | 136227 | Hs.131603 |
| EMILIN1 | 204163_at | NM_007046.1 | 11117 | Hs.63348 |
| EMILIN2 | 221980_at | NM_032048 | 84034 | Hs.270143 |
| EMILIN2 | 224374_s_at | NM_032048.2 | 84034 | Hs.532815 |
| EMILIN2 | 242288_s_at | NM_032048.2 | 84034 | Hs.532815 |
| EMILIN3 | 228307_at | NM_052846.1 | 90187 | Hs.25897 |
| EMILIN3 | 236262_at | NM_024756 | 79812 | Hs.127216 |
| EMP1 | 213895_at | NM_001423 | 2012 | Hs.306692 |
| EMP1 | 229011_at | NM_001423 | 2012 | Hs.306692 |
| EMP1 | 234232_at | AK024577.1 | 2012 | Hs.436298 |
| EMP1 | 234233_s_at | AK024577.1 | 2012 | Hs.436298 |
| EMP1 | 1564796_at | NM_001423 | 2012 | Hs.436298 |
| EMP1 | 201324_at | NM_001423.1 | 2012 | Hs.436298 |
| EMP1 | 201325_s_at | NM_001423.1 | 2012 | Hs.436298 |
| EMP2 | 225078_at | NM_001424 | 2013 | Hs.531561 |
| EMP2 | 225079_at | NM_001424 | 2013 | Hs.531561 |
| EMP2 | 238500_at | NM_001424 | 2013 | Hs.356835 |
| EMP2 | 204975_at | NM_001424.3 | 2013 | Hs.531561 |
| EMP3 | 203729_at | NM_001425.1 | 2014 | Hs.9999 |
| EMR1 | 207111_at | NM_001974.3 | 2015 | Hs.2375 |
| EMR2 | 232009_at | NM_013447.2 | 30817 | Hs.466039 |
| EMR2 | 207610_s_at | NM_013447.2 | 30817 | Hs.466039 |
| EMR3 | 210724_at | NM_032571.2 | 84658 | Hs.295626 |
| ENAM | 223893_at | NM_031889.1 | 10117 | Hs.283949 |
| ENAM | 240586_at | NM_031889 | 10117 | Hs.283949 |
| ENG | 228586_at | NM_000118 | 2022 | Hs.76753 |
| ENG | 201808_s_at | NM_000118.1 | 2022 | Hs.76753 |
| ENG | 201809_s_at | NM_000118.1 | 2022 | Hs.76753 |
| ENOX1 | 219501_at | NM_017993.1 | 55068 | Hs.128258 |
| ENPEP | 204844_at | NM_001977.2 | 2028 | Hs.435765 |
| ENPEP | 204845_s_at | NM_001977.2 | 2028 | Hs.435765 |
| ENPP1 | 205065_at | NM_006208 | 5167 | Hs.213840 |
| ENPP1 | 205066_s_at | NM_006208.1 | 5167 | Hs.527295 |
| ENPP2 | 209392_at | NM_006209.3 | 5168 | Hs.190977 |
| ENPP2 | 210839_s_at | NM_001040092.1 | 5168 | Hs.190977 |
| ENPP3 | 219912_s_at | NM_005021 | 5169 | Hs.264750 |
| ENPP3 | 222938_x_at | NM_005021.2 | 5169 | Hs.486489 |
| ENPP3 | 232737_s_at | NM_005021 | 5169 | Hs.486489 |
| ENPP4 | 204160_s_at | NM_014936.3 | 22875 | Hs.54037 |
| ENPP4 | 204161_s_at | NM_014936.3 | 22875 | Hs.54037 |
| ENPP5 | 227803_at | NM_021572.4 | 59084 | Hs.35198 |
| ENPP5 | 237054_at | BX647968.1 | 59084 | Hs.35198 |
| ENPP6 | 229916_at | NM_153343.2 | 133121 | Hs.297814 |
| ENTPD1 | 207691_x_at | NM_001776.3 | 953 | Hs.567230 |
| ENTPD1 | 209473_at | NM_001776 | 953 | Hs.205353 |
| ENTPD1 | 209474_s_at | NM_001776.3 | 953 | Hs.567230 |
| ENTPD2 | 230430_at | NM_001246.2 | 954 | Hs.123036 |
| ENTPD2 | 207372_s_at | NM_203468.1 | 954 | Hs.123036 |
| ENTPD3 | 1555118_at | NM_001248 | 956 | Hs.441145 |
| ENTPD3 | 206191_at | NM_001248.1 | 956 | Hs.441145 |
| ENTPD7 | 220153_at | NM_020354.2 | 57089 | Hs.369424 |
| EPB41 | 214530_x_at | NM_004437 | 2035 | Hs.37427 |
| EPB41 | 225051_at | NM_004437.2 | 2035 | Hs.175437 |
| EPB41 | 1554481_a_at | NM_004437.2 | 2035 | Hs.175437 |
| EPB41 | 207793_s_at | NM_203343.1 | 2035 | Hs.175437 |
| EPB41L2 | 231174_s_at | NM_001431 | 2037 | Hs.440387 |
| EPB41L2 | 242092_at | NM_001431 | 2037 | Hs.440387 |
| EPB41L2 | 201718_s_at | NM_001431.1 | 2037 | Hs.486470 |
| EPB41L2 | 201719_s_at | NM_001431.1 | 2037 | Hs.486470 |
| EPB41L3 | 206710_s_at | NM_012307.2 | 23136 | Hs.213394 |
| EPB41L3 | 211776_s_at | NM_012307.2 | 23136 | Hs.213394 |
| EPB41L3 | 212681_at | NM_012307.2 | 23136 | Hs.213394 |
| EPB42 | 210746_s_at | NM_000119.1 | 2038 | Hs.368642 |
| EPDR1 | 223253_at | NM_017549.2 | 54749 | Hs.416007 |
| EPHA1 | 215804_at | NM_005232 | 2041 | Hs.89839 |
| EPHA1 | 205977_s_at | NM_005232.3 | 2041 | Hs.89839 |
| EPHA10 | 1553371_at | NM_173641.1 | 284656 | Hs.129435 |
| EPHA2 | 203499_at | NM_004431.2 | 1969 | Hs.171596 |
| EPHA3 | 206070_s_at | NM_005233.3 | 2042 | Hs.123642 |
| EPHA3 | 206071_s_at | NM_005233.3 | 2042 | Hs.123642 |
| EPHA3 | 211164_at | NM_182644.1 | 2042 | Hs.123642 |
| EPHA4 | 227449_at | NM_004438.3 | 2043 | Hs.371218 |
| EPHA4 | 228948_at | NM_004438.3 | 2043 | Hs.371218 |
| EPHA4 | 229374_at | NM_004438.3 | 2043 | Hs.371218 |
| EPHA4 | 206114_at | NM_004438.3 | 2043 | Hs.371218 |
| EPHA5 | 215664_s_at | NM_004439.4 | 2044 | Hs.479853 |
| EPHA5 | 237939_at | NM_004439 | 2044 | Hs.479853 |
| EPHA6 | 233184_at | XM_942926.1 | 285220 | Hs.292059 |
| EPHA6 | 1561396_at | XM_114973.5 | 285220 | Hs.292059 |
| EPHA7 | 229288_at | NM_004440 | 2045 | Hs.73962 |
| EPHA7 | 238533_at | NM_004440 | 2045 | Hs.73962 |
| EPHA7 | 1554629_at | NM_004440 | 2045 | Hs.73962 |
| EPHA7 | 206852_at | NM_004440.2 | 2045 | Hs.73962 |
| EPHA8 | 231796_at | NM_020526.3 | 2046 | Hs.283613 |
| EPHA8 | 1554069_at | NM_001006943.1 | 2046 | Hs.283613 |
| EPHB1 | 217324_at | NM_004441 | 2047 | Hs.272311 |
| EPHB1 | 230425_at | NM_004441.3 | 2047 | Hs.116092 |
| EPHB1 | 210753_s_at | NM_004441.3 | 2047 | Hs.116092 |
| EPHB1 | 211898_s_at | NM_004441.3 | 2047 | Hs.116092 |
| EPHB2 | 209588_at | NM_017449.2 | 2048 | Hs.523329 |
| EPHB2 | 209589_s_at | NM_017449.2 | 2048 | Hs.523329 |
| EPHB2 | 210651_s_at | NM_017449.2 | 2048 | Hs.523329 |
| EPHB2 | 211165_x_at | NM_017449.2 | 2048 | Hs.523329 |
| EPHB3 | 1438_at | NM_004443.3 | 2049 | Hs.2913 |
| EPHB3 | 204600_at | NM_004443.3 | 2049 | Hs.2913 |
| EPHB4 | 216680_s_at | NM_004444.4 | 2050 | Hs.437008 |
| EPHB4 | 202894_at | NM_004444.4 | 2050 | Hs.437008 |
| EPHB6 | 204718_at | NM_004445.2 | 2051 | Hs.380089 |
| EPIM | 207346_at | NM_001980 | 2054 | Hs.437585 |
| EPIM | 213434_at | NM_194356.1 | 2054 | Hs.437585 |
| EPO | 217254_s_at | NM_000799.2 | 2056 | Hs.2303 |
| EPO | 207257_at | NM_000799.2 | 2056 | Hs.2303 |
| EPOR | 215054_at | NM_000121 | 2057 | Hs.127826 |
| EPOR | 216999_at | NM_000121 | 2057 | Hs.127826 |
| EPOR | 37986_at | NM_000121 | 2057 | Hs.127826 |
| EPOR | 396_f_at | NM_000121.2 | 2057 | Hs.127826 |
| EPOR | 209962_at | NM_000121.2 | 2057 | Hs.127826 |
| EPOR | 209963_s_at | NM_000121.2 | 2057 | Hs.127826 |
| EPS15L2 | 207750_at | AF119858.1 | 55380 |  |
| ERBB2 | 216836_s_at | NM_001005862.1 | 2064 | Hs.446352 |
| ERBB2 | 234354_x_at |  |  |  |
| ERBB2 | 210930_s_at | NM_001005862.1 | 2064 | Hs.446352 |
| ERBB3 | 215638_at | NM_001982 | 2065 | Hs.306251 |
| ERBB3 | 226213_at | NM_001982 | 2065 | Hs.118681 |
| ERBB3 | 1563252_at | NM_001982 | 2065 | Hs.118681 |
| ERBB3 | 1563253_s_at | NM_001982.2 | 2065 | Hs.118681 |
| ERBB3 | 202454_s_at | NM_001982.2 | 2065 | Hs.118681 |
| ERBB4 | 214053_at | NM_005235.1 | 2066 | Hs.390729 |
| ERBB4 | 233498_at | NM_005235.1 | 2066 | Hs.390729 |
| ERBB4 | 206794_at | NM_005235.1 | 2066 | Hs.390729 |
| EREG | 1569583_at | NM_001432 | 2069 | Hs.115263 |
| EREG | 205767_at | NM_001432.1 | 2069 | Hs.115263 |
| ERMAP | 219905_at | NM_001017922.1 | 114625 | Hs.439437 |
| ERVWE1 | 1553352_x_at | NM_014590.3 | 30816 |  |
| ERVWE1 | 1555299_s_at | NM_014590.3 | 30816 |  |
| ESAM | 225369_at | NM_138961.1 | 90952 | Hs.173840 |
| ESM1 | 208394_x_at | NM_007036 | 11082 | Hs.129944 |
| EVA1 | 203779_s_at | NM_005797.2 | 10205 | Hs.116651 |
| EVA1 | 203780_at | NM_005797.2 | 10205 | Hs.116651 |
| EVA1 | 230518_at | NM_144765.1 | 10205 | Hs.116651 |
| EVC | 219432_at | NM_014556 | 2121 | Hs.558326 |
| EVC | 234421_s_at | NM_153717.1 | 2121 | Hs.558326 |
| EVC | 1557375_at | NM_014556 | 2121 | Hs.28051 |
| EVC | 210887_s_at | NM_153717.1 | 2121 | Hs.558326 |
| EVC2 | 229974_at | NM_147127.3 | 132884 | Hs.87306 |
| EVC2 | 1555256_at | NM_147127 | 132884 | Hs.87306 |
| EVI2A | 204774_at | NM_014210.2 | 2123 | Hs.553491 |
| EVI2B | 211742_s_at | NM_006495.2 | 2124 | Hs.5509 |
| EXOC1 | 222127_s_at | NM_001024924.1 | 55763 | Hs.269665 |
| EXOC2 | 219349_s_at | NM_018303.4 | 55770 | Hs.484412 |
| EXOC2 | 226270_at | NM_018303.4 | 55770 | Hs.484412 |
| EXOC3 | 228847_at | AK074086.1 | 11336 | Hs.481464 |
| EXOC3 | 212630_at | NM_007277.4 | 11336 | Hs.481464 |
| EXOC6 | 226259_at | NM_001013848.1 | 54536 | Hs.292097 |
| EXOC6 | 232599_at | AF220217.1 | 54536 | Hs.292097 |
| EXOC6 | 233924_s_at | NM_001013848.1 | 54536 | Hs.292097 |
| EXOC7 | 214802_at | AK022397.1 | 23265 | Hs.533985 |
| EXOC7 | 215413_at | NM_015219 | 23265 | Hs.533985 |
| EXOC7 | 212026_s_at | NM_001013839.1 | 23265 | Hs.533985 |
| EXOC7 | 212034_s_at | NM_001013839.1 | 23265 | Hs.533985 |
| EXOC7 | 212035_s_at | NM_001013839.1 | 23265 | Hs.533985 |
| F10 | 205620_at | NM_000504.3 | 2159 | Hs.361463 |
| F11 | 1563160_at | NM_000128 | 2160 | Hs.1430 |
| F11 | 1569591_at | NM_000128 | 2160 | Hs.1430 |
| F11 | 1569592_a_at | NM_000128 | 2160 | Hs.1430 |
| F11 | 206610_s_at | NM_000128.2 | 2160 | Hs.1430 |
| F11R | 221664_s_at | NM_016946.3 | 50848 | Hs.517293 |
| F11R | 222354_at | NM_016946 | 50848 | Hs.414880 |
| F11R | 223000_s_at | NM_144502.1 | 50848 | Hs.517293 |
| F11R | 224097_s_at | NM_144503.1 | 50848 | Hs.517293 |
| F11R | 226482_s_at | NM_016946 | 50848 | Hs.517293 |
| F12 | 215961_at | NM_000505 | 2161 | Hs.1321 |
| F12 | 205774_at | NM_000505.2 | 2161 | Hs.1321 |
| F13A1 | 203305_at | NM_000129.2 | 2162 | Hs.335513 |
| F13B | 207810_at | NM_001994.1 | 2165 | Hs.435782 |
| F2 | 205754_at | NM_000506.2 | 2147 | Hs.76530 |
| F2R | 1569642_at | NM_001992 | 2149 | Hs.482562 |
| F2R | 203989_x_at | NM_001992.2 | 2149 | Hs.482562 |
| F2RL1 | 213506_at | NM_005242.3 | 2150 | Hs.154299 |
| F2RL1 | 206429_at | NM_005242.3 | 2150 | Hs.154299 |
| F2RL2 | 230147_at | NM_004101.2 | 2151 | Hs.42502 |
| F2RL2 | 206795_at | NM_004101.2 | 2151 | Hs.42502 |
| F2RL3 | 207221_at | NM_003950 | 9002 | Hs.137574 |
| F3 | 204363_at | NM_001993.2 | 2152 | Hs.62192 |
| F5 | 204713_s_at | NM_000130 | 2153 | Hs.30054 |
| F5 | 204714_s_at | NM_000130 | 2153 | Hs.30054 |
| F7 | 237414_at | NM_000131 | 2155 | Hs.36989 |
| F7 | 207300_s_at | NM_000131.2 | 2155 | Hs.36989 |
| F8 | 205756_s_at | NM_019863.2 | 2157 | Hs.413083 |
| FAAH2 | 230792_at | NM_174912.2 | 158584 | Hs.496205 |
| FADS2 | 243953_at | NM_004265 | 9415 | Hs.502745 |
| FADS2 | 202218_s_at | NM_004265.2 | 9415 | Hs.502745 |
| FAIM2 | 203618_at | NM_012306.2 | 23017 | Hs.182859 |
| FAIM2 | 203619_s_at | NM_012306.2 | 23017 | Hs.182859 |
| FAIM3 | 221601_s_at | NM_005449.3 | 9214 | Hs.58831 |
| FAIM3 | 221602_s_at | NM_005449.3 | 9214 | Hs.58831 |
| FAM119A | 235177_at | NM_145280.3 | 151194 | Hs.516646 |
| FAM119B | 213861_s_at | NM_206914.1 | 25895 | Hs.528351 |
| FAM11A | 1554105_at | NM_032508.1 | 84548 | Hs.522172 |
| FAM11A | 231326_s_at | AI914124 | 84548 | 522172 |
| FAM11B | 219253_at | NR_000034 | 79134 | Hs.376722 |
| FAM12A | 217568_at | NM_006683.4 | 10876 | Hs.304757 |
| FAM12A | 208142_at | NM_006683.4 | 10876 | Hs.304757 |
| FAM12A | 208143_s_at | NM_006683.4 | 10876 | Hs.304757 |
| FAM12B | 220759_at | NM_022360.4 | 64184 | Hs.525202 |
| FAM131A | 221904_at | NM_144635.3 | 131408 | Hs.436847 |
| FAM131A | 232806_s_at | NM_144635.3 | 131408 | Hs.436847 |
| FAM131A | 232807_at | NM_144635 | 131408 | Hs.436847 |
| FAM134C | 212697_at | NM_178126.2 | 162427 | Hs.463079 |
| FAM14A | 223626_x_at | NM_032036.2 | 83982 | Hs.94695 |
| FAM14B | 230172_at | NM_145249 | 122509 | Hs.19414 |
| FAM18B | 218446_s_at | NM_016078.3 | 51030 | Hs.87295 |
| FAM18B2 | 1552377_s_at | NM_145301.1 | 201158 | Hs.528884 |
| FAM19A1 | 230923_at | NM_213609.2 | 407738 | Hs.7047 |
| FAM19A4 | 242348_at | NM_001005527.1 | 151647 | Hs.187873 |
| FAM19A5 | 229655_at | AK123443.1 | 25817 | Hs.436854 |
| FAM20A | 226804_at | NM_017565.2 | 54757 | Hs.268874 |
| FAM20A | 241981_at | NM_017565 | 54757 | Hs.268874 |
| FAM20A | 242945_at | NM_017565 | 54757 | Hs.268874 |
| FAM20A | 243221_at | AA579773 |  | Hs.115930 |
| FAM20B | 202915_s_at | NM_014864.2 | 9917 | Hs.5737 |
| FAM20B | 202916_s_at | NM_014864.2 | 9917 | Hs.5737 |
| FAM20C | 226722_at | NM_020223.1 | 56975 | Hs.134742 |
| FAM24A | 243143_at | NM_001029888.1 | 118670 | Hs.369829 |
| FAM24B | 231146_at | NM_152644.1 | 196792 | Hs.114648 |
| FAM26A | 1554711_at | NM_182494.1 | 119395 | Hs.364624 |
| FAM26B | 221565_s_at | NM_015916.3 | 51063 | Hs.241545 |
| FAM26B | 57715_at | NM_015916.3 | 51063 | Hs.241545 |
| FAM26C | 1554367_at | NM_001001412.1 | 255022 | Hs.530398 |
| FAM26F | 229390_at | NM_001010919.1 | 441168 | Hs.381220 |
| FAM26F | 229391_s_at | NM_001010919.1 | 441168 | Hs.381220 |
| FAM38B | 219602_s_at | NM_022068.1 | 63895 | Hs.293907 |
| FAM38B | 222908_at | AK026797.1 | 63895 | Hs.293907 |
| FAM3A | 38043_at | NM_021806.1 | 60343 | Hs.289108 |
| FAM3A | 209405_s_at | NM_021806.1 | 60343 | Hs.289108 |
| FAM3B | 227194_at | NM_058186.3 | 54097 | Hs.473877 |
| FAM3C | 236316_at | NM_014888 | 10447 | Hs.434053 |
| FAM3C | 240062_at | NM_014888 | 10447 | Hs.434053 |
| FAM3C | 201889_at | NM_014888.2 | 10447 | Hs.562055 |
| FAM3D | 227676_at | NM_138805.2 | 131177 | Hs.61265 |
| FAM55A | 1553828_at | NM_152315.1 | 120400 | Hs.374720 |
| FAM55A | 1561387_a_at | NM_152315.1 | 120400 | Hs.374720 |
| FAM55D | 220645_at | NM_017678.1 | 54827 | Hs.179100 |
| FAM57A | 218898_at | NM_024792.1 | 79850 | Hs.154396 |
| FAM5B | 214822_at | NM_021165.1 | 57795 | Hs.495918 |
| FAM5C | 217562_at | NM_199051.1 | 339479 | Hs.65765 |
| FAM62A | 208858_s_at | NM_015292.1 | 23344 | Hs.8309 |
| FAM62B | 224698_at | NM_020728.1 | 57488 | Hs.490795 |
| FAM62B | 224699_s_at | NM_020728.1 | 57488 | Hs.490795 |
| FAM62B | 1555829_at | BC013957.2 | 57488 | Hs.490795 |
| FAM62B | 1555830_s_at | NM_020728.1 | 57488 | Hs.490795 |
| FAM62B | 1558511_s_at | NM_020728.1 | 57488 | Hs.490795 |
| FAM62B | 1564424_at | BC035983 | 57488 | 490795 |
| FAM62C | 239770_at | AJ697972.1 | 83850 | Hs.477711 |
| FAM62C | 1554912_at | BC037292.1 | 83850 | Hs.477711 |
| FAM62C | 1557793_at | AA149621 | 83850 | Hs.318921 |
| FAM69A | 216044_x_at | AK027146.1 | 388650 | Hs.180946 |
| FAM69B | 227652_at | NM_152421.2 | 138311 | Hs.495480 |
| FAM69B | 229002_at | NM_152421.2 | 138311 | Hs.495480 |
| FAM70A | 219895_at | NM_017938.2 | 55026 | Hs.437563 |
| FAM73A | 243042_at | NM_198549.1 | 374986 | Hs.156625 |
| FAM73B | 226356_at | NM_032809.2 | 84895 | Hs.325309 |
| FAM77C | 219438_at | NM_024522.1 | 79570 | Hs.470259 |
| FAM77D | 1553241_at | NM_173688.1 | 286183 | Hs.491856 |
| FAM87B | 1559140_at | XM_927098.1 | 400728 | Hs.406174 |
| FAM87B | 1559141_s_at | XM_927098.1 | 400728 | Hs.406174 |
| FAM8A1 | 203420_at | NM_016255.1 | 51439 | Hs.95260 |
| FAP | 209955_s_at | NM_004460.2 | 2191 | Hs.516493 |
| FAS | 215719_x_at | NM_152874.1 | 355 | Hs.244139 |
| FAS | 216252_x_at | NM_152874.1 | 355 | Hs.244139 |
| FAS | 204780_s_at | NM_000043.3 | 355 | Hs.244139 |
| FAS | 204781_s_at | NM_000043.3 | 355 | Hs.244139 |
| FASLG | 210865_at | NM_000639.1 | 356 | Hs.2007 |
| FASLG | 211333_s_at | NM_000639.1 | 356 | Hs.2007 |
| FAT | 201579_at | NM_005245.3 | 2195 | Hs.481371 |
| FAT2 | 208153_s_at | NM_001447.1 | 2196 | Hs.132158 |
| FAT3 | 236029_at | XM_061871 | 120114 | Hs.258867 |
| FAT3 | 1558964_at | AA334950 | 120114 | Hs.258867 |
| FAT3 | 1560490_at | BC016722.1 | 120114 | Hs.258867 |
| FAT4 | 219427_at | AL713715.1 | 79633 | Hs.269121 |
| FBLN1 | 201787_at | NM_001996.2 | 2192 | Hs.24601 |
| FBLN1 | 202994_s_at | NM_006486.2 | 2192 | Hs.24601 |
| FBLN1 | 202995_s_at | NM_006486.2 | 2192 | Hs.24601 |
| FBLN1 | 207834_at | NM_006485.2 | 2192 | Hs.24601 |
| FBLN1 | 207835_at | NM_006487.2 | 2192 | Hs.24601 |
| FBLN2 | 203886_s_at | NM_001998.2 | 2199 | Hs.198862 |
| FBLN5 | 203088_at | NM_006329.2 | 10516 | Hs.332708 |
| FBN1 | 235318_at | NM_000138.3 | 2200 | Hs.146447 |
| FBN1 | 202765_s_at | NM_000138.3 | 2200 | Hs.146447 |
| FBN1 | 202766_s_at | NM_000138.3 | 2200 | Hs.146447 |
| FBN2 | 215717_s_at | NM_001999 | 2201 | Hs.519294 |
| FBN2 | 1554737_at | NM_001999 | 2201 | Hs.519294 |
| FBN2 | 203184_at | NM_001999.3 | 2201 | Hs.519294 |
| FBN3 | 240117_at | NM_032447.3 | 84467 | Hs.370362 |
| FCAMR | 231496_at | NM_032029.1 | 83953 | Hs.145519 |
| FCAR | 207674_at | NM_002000.2 | 2204 | Hs.193122 |
| FCAR | 211305_x_at | NM_002000 | 2204 | Hs.193122 |
| FCAR | 211306_s_at | NM_133280.1 | 2204 | Hs.193122 |
| FCAR | 211307_s_at | NM_002000.2 | 2204 | Hs.193122 |
| FCAR | 211816_x_at | NM_002000.2 | 2204 | Hs.193122 |
| FCER1A | 1562537_at | NM_002001 | 2205 | Hs.897 |
| FCER1A | 211734_s_at | NM_002001.2 | 2205 | Hs.897 |
| FCER1G | 1554899_s_at | NM_004106.1 | 2207 | Hs.433300 |
| FCER1G | 204232_at | NM_004106.1 | 2207 | Hs.433300 |
| FCER2 | 206759_at | NM_002002.3 | 2208 | Hs.465778 |
| FCER2 | 206760_s_at | NM_002002.3 | 2208 | Hs.465778 |
| FCGBP | 203240_at | NM_003890.1 | 8857 | Hs.111732 |
| FCGR1A | 214511_x_at | NM_000566.2 | 2209 | Hs.77424 |
| FCGR1A | 216950_s_at | NM_000566.2 | 2209 | Hs.77424 |
| FCGR1A | 216951_at | NM_000566 | 2209 | Hs.77424 |
| FCGR2A | 1565673_at | NM_021642 | 2212 | Hs.352642 |
| FCGR2A | 1565674_at | NM_021642 | 2212 | Hs.352642 |
| FCGR2A | 203561_at | NM_021642.2 | 2212 | Hs.352642 |
| FCGR2B | 210889_s_at | NM_001002273.1 | 2213 | Hs.492655 |
| FCGR2B | 211395_x_at | NM_001002273.1 | 2213 | Hs.492655 |
| FCGR2C | 210992_x_at | NM_201563.2 | 9103 | Hs.492655 |
| FCGR2C | 211396_at | NM_001005412.1 | 9103 | Hs.492655 |
| FCGR3A | 204006_s_at | NM_000569.6 | 2214 | Hs.372679 |
| FCGR3B | 204007_at | NM_000570.2 | 2215 | Hs.372679 |
| FCGRT | 218831_s_at | NM_004107.3 | 2217 | Hs.111903 |
| FCN1 | 1560034_a_at | NM_002003 | 2219 | Hs.440898 |
| FCN1 | 205237_at | NM_002003.2 | 2219 | Hs.440898 |
| FCN2 | 1559577_at | AK096112.1 | 2220 | Hs.54517 |
| FCN2 | 207804_s_at | NM_004108.2 | 2220 | Hs.54517 |
| FCN2 | 208439_s_at | NM_004108 | 2220 | Hs.54517 |
| FCN3 | 205866_at | NM_173452.1 | 8547 | Hs.333383 |
| FCRL1 | 235982_at | NM_052938.3 | 115350 | Hs.374126 |
| FCRL1 | 243968_x_at | NM_052938 | 115350 | Hs.374126 |
| FCRL2 | 221239_s_at | NM_030764.2 | 79368 | Hs.437393 |
| FCRL2 | 224192_at | AF319439.1 | 79368 | Hs.437393 |
| FCRL2 | 224193_s_at | NM_030764.2 | 79368 | Hs.437393 |
| FCRL2 | 224194_at | AF319440.1 | 79368 | Hs.437393 |
| FCRL2 | 1563674_at | NM_030764.2 | 79368 | Hs.437393 |
| FCRL3 | 1553196_a_at | NM_052939.3 | 115352 | Hs.292449 |
| FCRL4 | 224401_s_at | NM_031282.1 | 83417 | Hs.120260 |
| FCRL4 | 224402_s_at | NM_031282.1 | 83417 | Hs.120260 |
| FCRL4 | 224403_at | NM_031282.1 | 83417 | Hs.120260 |
| FCRL5 | 224404_s_at | AF343662.1 | 83416 | Hs.415950 |
| FCRL5 | 224405_at | AF343663.1 | 83416 | Hs.415950 |
| FCRL5 | 224406_s_at | NM_031281.1 | 83416 | Hs.415950 |
| FCRL5 | 231647_s_at | AF343663.1 | 83416 | Hs.415950 |
| FCRLM1 | 235372_at | NM_032738.3 | 84824 | Hs.266331 |
| FCRLM1 | 235400_at | NM_032738.3 | 84824 | Hs.266331 |
| FCRLM1 | 235401_s_at | NM_032738.3 | 84824 | Hs.266331 |
| FER1L3 | 201798_s_at | NM_013451.2 | 26509 | Hs.500572 |
| FER1L3 | 211864_s_at | NM_133337.1 | 26509 | Hs.500572 |
| FER1L3 | 217518_at | NM_013451 | 26509 | Hs.362731 |
| FERMT3 | 223303_at | NM_178443.1 | 83706 | Hs.180535 |
| FETUB | 210521_s_at | NM_014375.2 | 26998 | Hs.81073 |
| FGA | 231671_at | NM_000508 | 2243 | Hs.351593 |
| FGA | 205649_s_at | NM_000508.3 | 2243 | Hs.351593 |
| FGA | 205650_s_at | NM_021871.2 | 2243 | Hs.351593 |
| FGB | 216238_s_at | NM_005141.2 | 2244 | Hs.300774 |
| FGB | 204988_at | NM_005141.2 | 2244 | Hs.300774 |
| FGF1 | 1552721_a_at | NM_000800.2 | 2246 | Hs.483635 |
| FGF1 | 205117_at | NM_000800 | 2246 | 483635 |
| FGF1 | 208240_s_at | NM_033136.1 | 2246 | Hs.483635 |
| FGF10 | 231762_at | NM_004465.1 | 2255 | Hs.248049 |
| FGF11 | 227271_at | NM_004112.2 | 2256 | Hs.528468 |
| FGF11 | 231803_at | NM_004112.2 | 2256 | Hs.528468 |
| FGF16 | 221374_at | NM_003868.1 | 8823 | Hs.534376 |
| FGF17 | 221376_at | NM_003867.2 | 8822 | Hs.248192 |
| FGF18 | 231382_at | NM_003862 | 8817 | Hs.87191 |
| FGF18 | 206987_x_at | NM_003862.1 | 8817 | Hs.87191 |
| FGF18 | 211029_x_at | NM_003862.1 | 8817 | Hs.87191 |
| FGF18 | 211485_s_at | NM_003862.1 | 8817 | Hs.87191 |
| FGF19 | 223761_at | NM_005117.2 | 9965 | Hs.249200 |
| FGF2 | 204421_s_at | NM_002006.3 | 2247 | Hs.284244 |
| FGF2 | 204422_s_at | NM_002006.3 | 2247 | Hs.284244 |
| FGF20 | 220394_at | NM_019851.1 | 26281 | Hs.199905 |
| FGF21 | 221433_at | NM_019113.2 | 26291 | Hs.283015 |
| FGF22 | 221315_s_at | NM_020637.1 | 27006 | Hs.248087 |
| FGF22 | 242184_s_at | NM_020637 | 27006 | Hs.248087 |
| FGF22 | 242185_at | NM_020637 | 27006 | Hs.248087 |
| FGF22 | 1566814_at | NM_020637 | 27006 | Hs.248087 |
| FGF22 | 1566816_at | NM_020637 | 27006 | Hs.248087 |
| FGF23 | 221166_at | NM_020638.2 | 8074 | Hs.287370 |
| FGF3 | 214571_at | NM_005247.2 | 2248 | Hs.37092 |
| FGF4 | 1552982_a_at | NM_002007.1 | 2249 | Hs.1755 |
| FGF5 | 208378_x_at | NM_033143.2 | 2250 | Hs.37055 |
| FGF5 | 210310_s_at | NM_004464.3 | 2250 | Hs.37055 |
| FGF5 | 210311_at | NM_004464.3 | 2250 | Hs.37055 |
| FGF6 | 208417_at | NM_020996.1 | 2251 | Hs.166015 |
| FGF7 | 1555102_at | NM_002009 | 2252 | Hs.122006 |
| FGF7 | 1555103_s_at | NM_002009 | 2252 | Hs.122006 |
| FGF7 | 205782_at | NM_002009.2 | 2252 | Hs.122006 |
| FGF8 | 208449_s_at | NM_006119.2 | 2253 | Hs.57710 |
| FGF9 | 206404_at | NM_002010.1 | 2254 | Hs.111 |
| FGFBP1 | 205014_at | NM_005130.3 | 9982 | Hs.1690 |
| FGFBP2 | 223836_at | NM_031950.2 | 83888 | Hs.98785 |
| FGFR1 | 215404_x_at | NM_000604 | 2260 | Hs.264887 |
| FGFR1 | 222164_at | AK001052.1 | 2260 | Hs.264887 |
| FGFR1 | 226705_at | NM_000604 | 2260 | Hs.264887 |
| FGFR1 | 207822_at | NM_000604 | 2260 | Hs.264887 |
| FGFR1 | 207937_x_at | NM_000604 | 2260 | Hs.264887 |
| FGFR1 | 210973_s_at | NM_000604.2 | 2260 | Hs.264887 |
| FGFR1 | 211535_s_at | NM_023109.1 | 2260 | Hs.264887 |
| FGFR2 | 230842_at | NM_000141 | 2263 | Hs.404081 |
| FGFR2 | 240913_at | NM_000141 | 2263 | Hs.404081 |
| FGFR2 | 1560859_at | AF086021.1 | 2263 | Hs.533683 |
| FGFR2 | 203638_s_at | NM_022969.1 | 2263 | Hs.533683 |
| FGFR2 | 203639_s_at | NM_022972.1 | 2263 | Hs.533683 |
| FGFR2 | 208225_at | NM_022971.1 | 2263 | Hs.533683 |
| FGFR2 | 208228_s_at | NM_022969.1 | 2263 | Hs.533683 |
| FGFR2 | 208234_x_at | NM_022969.1 | 2263 | Hs.533683 |
| FGFR2 | 211398_at | NM_000141 | 2263 | Hs.533683 |
| FGFR2 | 211399_at | NM_000141 | 2263 | Hs.533683 |
| FGFR2 | 211400_at | NM_000141 | 2263 | Hs.533683 |
| FGFR2 | 211401_s_at | NM_022972.1 | 2263 | Hs.533683 |
| FGFR3 | 222006_at | NM_000142 | 2261 | Hs.1420 |
| FGFR3 | 204379_s_at | NM_000142.2 | 2261 | Hs.1420 |
| FGFR3 | 204380_s_at | NM_022965.1 | 2261 | Hs.1420 |
| FGFR4 | 1554961_at | NM_002011 | 2264 | Hs.165950 |
| FGFR4 | 1554962_a_at | NM_002011.3 | 2264 | Hs.165950 |
| FGFR4 | 204579_at | NM_002011.3 | 2264 | Hs.165950 |
| FGFR4 | 211237_s_at | NM_002011.3 | 2264 | Hs.165950 |
| FGFRL1 | 223321_s_at | NM_001004356.2 | 53834 | Hs.193326 |
| FGFRL1 | 223648_s_at | NM_001004356.2 | 53834 | Hs.193326 |
| FGG | 219612_s_at | NM_000509.4 | 2266 | Hs.546255 |
| FGG | 226621_at | NM_000509 | 2266 | Hs.75431 |
| FGL1 | 1570237_at | NM_004467 | 2267 | Hs.107 |
| FGL1 | 205305_at | NM_004467.3 | 2267 | Hs.491143 |
| FGL2 | 204834_at | NM_006682 | 10875 | Hs.520989 |
| FIBCD1 | 238943_at | NM_032843.3 | 84929 | Hs.133205 |
| FIBIN | 226769_at | NM_203371.1 | 387758 | Hs.32478 |
| FIBIN | 231001_at | NM_203371.1 | 387758 | Hs.32478 |
| FICD | 219910_at | NM_007076.2 | 11153 | Hs.234961 |
| FIGF | 206742_at | NM_004469.2 | 2277 | Hs.11392 |
| FJX1 | 219522_at | NM_014344.2 | 24147 | Hs.39384 |
| FKBP11 | 219117_s_at | NM_016594.1 | 51303 | Hs.438695 |
| FKBP11 | 219118_at | NM_016594.1 | 51303 | Hs.438695 |
| FKBP11 | 228308_at | NM_033124 | 51303 | Hs.438695 |
| FLJ00060 | 216428_x_at | AK024467.1 | 90011 | Hs.288520 |
| FLJ00060 | 216492_at | AK024467 | 90011 | Hs.288520 |
| FLJ10099 | 218008_at | NM_017994 | 55069 | Hs.488478 |
| FLJ10099 | 224688_at | NM_017994 | 55069 | Hs.287955 |
| FLJ10815 | 218727_at | NM_018231.1 | 55238 | Hs.10499 |
| FLJ10815 | 222215_at | NM_018231 | 55238 | Hs.10499 |
| FLJ10815 | 56821_at | NM_018231.1 | 55238 | Hs.10499 |
| FLJ12688 | 226467_at | AK095333.1 | 79613 | Hs.13526 |
| FLJ13096 | 220172_at | NM_025000 | 80067 | Hs.413518 |
| FLJ13096 | 231921_at | NM_025000 | 80067 | Hs.413518 |
| FLJ13236 | 220441_at | NM_024902.2 | 79962 | Hs.170298 |
| FLJ13236 | 244193_at | NM_024902 | 79962 | Hs.170298 |
| FLJ13576 | 218962_s_at | NM_022484 | 64418 | Hs.121847 |
| FLJ13576 | 234726_s_at | NM_022484 | 64418 | Hs.121847 |
| FLJ14627 | 221908_at | NM_032814 | 84900 | Hs.347310 |
| FLJ14627 | 221909_at | NM_032814 | 84900 | Hs.437195 |
| FLJ14681 | 225411_at | NM_032824 | 84910 | Hs.23317 |
| FLJ14681 | 225412_at | NM_032824 | 84910 | Hs.23317 |
| FLJ14712 | 239552_at | NM_182545 | 221806 | Hs.50802 |
| FLJ14803 | 222267_at | NM_032842 | 84928 | Hs.267245 |
| FLJ20160 | 219858_s_at | NM_017694.2 | 54842 | Hs.418581 |
| FLJ20160 | 225325_at | NM_017694.2 | 54842 | Hs.418581 |
| FLJ20254 | 217899_at | NM_017727 | 54867 | Hs.533934 |
| FLJ20255 | 220097_s_at | NM_017728 | 54868 | Hs.370262 |
| FLJ20513 | 220133_at | NM_017855 | 54959 | Hs.143811 |
| FLJ21749 | 1558712_at | NM_025124 | 80194 | Hs.288761 |
| FLJ21749 | 233018_at | NM_025124 | 80194 | Hs.288761 |
| FLJ22170 | 240875_at | NM_025099 | 80169 | Hs.156055 |
| FLJ22222 | 219254_at | NM_024648.1 | 79701 | Hs.187505 |
| FLJ22222 | 53071_s_at | NM_175902.3 | 79701 | Hs.187505 |
| FLJ22222 | 64438_at | NM_175902.3 | 79701 | Hs.187505 |
| FLJ22938 | 229998_x_at | NM_024676 | 79729 | Hs.87016 |
| FLJ23548 | 219973_at | NM_024590 | 79642 | Hs.22895 |
| FLJ25082 | 242062_at | NM_144660 | 142891 | Hs.282105 |
| FLJ30934 | 1554181_at | NM_152760.2 | 254122 | Hs.533600 |
| FLJ31810 | 232720_at | NM_152570 | 158038 | Hs.446325 |
| FLJ31842 | 234980_at | NM_152487 | 148534 | Hs.84522 |
| FLJ31842 | 237515_at | NM_152487 | 148534 | Hs.84522 |
| FLJ32784 | 1552853_at | NM_144623 | 127731 | Hs.205178 |
| FLJ32784 | 1552854_a_at | NM_144623 | 127731 | Hs.205178 |
| FLJ32894 | 1553484_at | NM_144667.1 | 144360 | Hs.350668 |
| FLJ33674 | 1556308_at | BC040508.1 | 285368 | Hs.517789 |
| FLJ34221 | 239883_s_at | NM_178826 | 121601 | Hs.58785 |
| FLJ35773 | 1552432_at | NM_152599.2 | 162387 | Hs.213603 |
| FLJ35880 | 1553834_at | NM_153264.2 | 256076 | Hs.205403 |
| FLJ35880 | 1553835_a_at | NM_153264.2 | 256076 | Hs.205403 |
| FLJ37034 | 229622_at | BC031951.1 | 151176 | Hs.556113 |
| FLJ37440 | 229247_at | NM_153214.1 | 129804 | Hs.437696 |
| FLJ38482 | 226589_at | NM_152681 | 201931 | Hs.369632 |
| FLJ39207 | 1569057_s_at | XM_371369.3 | 375056 | Hs.118474 |
| FLJ39207 | 212305_s_at | XM_371369.3 | 375056 | Hs.118474 |
| FLJ39207 | 212310_at | XM_371369.3 | 375056 | Hs.118474 |
| FLJ40941 | 1553657_at | XM_936977.1 | 146177 | Hs.10697 |
| FLJ90013 | 216373_at | NM_153365 | 202018 | Hs.479223 |
| FLJ90013 | 216507_at | NM_153365 | 202018 | Hs.434489 |
| FLJ90013 | 226735_at | NM_153365 | 202018 | Hs.479223 |
| FLJ90013 | 238798_at | NM_153365 | 202018 | Hs.434489 |
| FLJ90013 | 242318_at | NM_153365 | 202018 | Hs.434489 |
| FLJ90724 | 1553706_at | NM_153692 | 203100 | Hs.322452 |
| FLOT1 | 1562165_at | NM_005803 | 10211 | Hs.179986 |
| FLOT1 | 208748_s_at | NM_005803.2 | 10211 | Hs.179986 |
| FLOT1 | 208749_x_at | NM_005803.2 | 10211 | Hs.179986 |
| FLOT1 | 210142_x_at | NM_005803.2 | 10211 | Hs.179986 |
| FLOT2 | 201350_at | NM_004475.2 | 2319 | Hs.514038 |
| FLOT2 | 211299_s_at | NM_004475.2 | 2319 | Hs.514038 |
| FLRT1 | 210414_at | NM_013280.4 | 23769 | Hs.558469 |
| FLRT2 | 204358_s_at | NM_013231.4 | 23768 | Hs.533710 |
| FLRT2 | 204359_at | NM_013231.4 | 23768 | Hs.533710 |
| FLRT3 | 219250_s_at | NM_013281.2 | 23767 | Hs.41296 |
| FLRT3 | 222853_at | NM_198391.1 | 23767 | Hs.41296 |
| FLT1 | 222033_s_at | NM_002019 | 2321 | Hs.347713 |
| FLT1 | 226497_s_at | NM_002019 | 2321 | Hs.347713 |
| FLT1 | 226498_at | NM_002019 | 2321 | Hs.347713 |
| FLT1 | 210287_s_at | U01134.1 | 2321 | Hs.507621 |
| FLT3 | 206674_at | NM_004119.1 | 2322 | Hs.507590 |
| FLT3LG | 206980_s_at | NM_001459.2 | 2323 | Hs.428 |
| FLT3LG | 210607_at | NM_001459 | 2323 | Hs.428 |
| FLT4 | 234379_at | NM_002020 | 2324 | Hs.415048 |
| FLT4 | 210316_at | NM_002020 | 2324 | Hs.415048 |
| FLVCR | 222906_at | NM_014053 | 28982 | Hs.7055 |
| FMOD | 202709_at | NM_002023.3 | 2331 | Hs.519168 |
| FMR1NB | 1552906_at | NM_152578.1 | 158521 | Hs.128580 |
| FN1 | 214701_s_at | NM_002026.2 | 2335 | Hs.203717 |
| FN1 | 214702_at | NM_054034.2 | 2335 | Hs.203717 |
| FN1 | 216442_x_at | NM_002026.2 | 2335 | Hs.203717 |
| FN1 | 1558199_at | NM_002026 | 2335 | Hs.203717 |
| FN1 | 210495_x_at | NM_002026.2 | 2335 | Hs.203717 |
| FN1 | 211719_x_at | NM_002026.2 | 2335 | Hs.203717 |
| FN1 | 212464_s_at | NM_002026.2 | 2335 | Hs.203717 |
| FNDC1 | 226930_at | NM_032532.1 | 84624 | Hs.520525 |
| FNDC4 | 218843_at | NM_022823.1 | 64838 | Hs.27836 |
| FNDC6 | 228575_at | NM_144717.2 | 152028 | Hs.61232 |
| FNDC7 | 240837_at | NM_173532.1 | 163479 | Hs.258253 |
| FOLH1 | 217487_x_at | NM_001014986.1 | 2346 | Hs.523961 |
| FOLH1 | 205860_x_at | NM_001014986.1 | 2346 | Hs.380325 |
| FOLH1 | 211303_x_at | NM_004476.1 | 2346 | Hs.380325 |
| FOLR1 | 204437_s_at | NM_016729.1 | 2348 | Hs.73769 |
| FOLR1 | 211074_at | NM_000802 | 2348 | 73769 |
| FOLR2 | 229619_at | NM_000803 | 2350 | Hs.433159 |
| FOLR2 | 204829_s_at | NM_000803.2 | 2350 | Hs.433159 |
| FOLR3 | 206371_at | NM_000804.2 | 2352 | Hs.352 |
| FPR1 | 205118_at | NM_002029 | 2357 | Hs.753 |
| FPR1 | 205119_s_at | NM_002029.3 | 2357 | Hs.753 |
| FPRL1 | 210772_at | NM_001005738.1 | 2358 | Hs.99855 |
| FPRL1 | 210773_s_at | NM_001005738.1 | 2358 | Hs.99855 |
| FPRL2 | 214560_at | NM_002030.3 | 2359 | Hs.445466 |
| FPRL2 | 230422_at | NM_002030.3 | 2359 | Hs.445466 |
| FRAS1 | 220910_at | NM_206841.1 | 80144 | Hs.369448 |
| FRAS1 | 226145_s_at | NM_025074 | 80144 | Hs.369448 |
| FRAS1 | 231511_at | NM_025074 | 80144 | Hs.15420 |
| FRAS1 | 1552503_at | NM_032863.2 | 80144 | Hs.369448 |
| FRAS1 | 1560153_at | AK092082.1 | 80144 | Hs.369448 |
| FREM1 | 228233_at | NM_144966.3 | 158326 | Hs.31564 |
| FREM2 | 230964_at | XM_929884.1 | 341640 | Hs.253994 |
| FREQ | 218266_s_at | NM_014286.2 | 23413 | Hs.301760 |
| FREQ | 222570_at | NM_014286.2 | 23413 | Hs.301760 |
| FREQ | 230138_at | NM_014286 | 23413 | Hs.301760 |
| FREQ | 230146_s_at | NM_014286.2 | 23413 | Hs.301760 |
| FREQ | 238753_at | NM_014286 | 23413 | Hs.301760 |
| FRMD5 | 230831_at | AW294986 |  | Hs.444681 |
| FRMD5 | 1569470_a_at | NM_032892.3 | 84978 | Hs.368399 |
| FRMD6 | 225464_at | AK126521.1 | 122786 | Hs.434914 |
| FRMD6 | 225481_at | BX647863.1 | 122786 | Hs.434914 |
| FRS2 | 221308_at | NM_006654 | 10818 | Hs.334831 |
| FRS2 | 226045_at | NM_006654 | 10818 | Hs.334831 |
| FRS2 | 233318_at | NM_006654 | 10818 | Hs.202404 |
| FRS2 | 238486_at | NM_006654 | 10818 | Hs.202404 |
| FRY | 214318_s_at | NM_023037.1 | 10129 | Hs.507669 |
| FRY | 214319_at | NM_023037 | 10129 | Hs.507669 |
| FRY | 1555904_at | NM_023037 | 10129 | Hs.390874 |
| FRY | 204072_s_at | NM_023037.1 | 10129 | Hs.507669 |
| FRZB | 231273_x_at | NM_001463 | 2487 | Hs.128453 |
| FRZB | 244419_at | NM_001463 | 2487 | Hs.128453 |
| FRZB | 203697_at | NM_001463.2 | 2487 | Hs.128453 |
| FRZB | 203698_s_at | NM_001463.2 | 2487 | Hs.128453 |
| FSHB | 214489_at | NM_000510.2 | 2488 | Hs.36975 |
| FSHR | 211201_at | NM_000145.2 | 2492 | Hs.1428 |
| FST | 226847_at | NM_006350 | 10468 | Hs.9914 |
| FST | 204948_s_at | NM_006350.2 | 10468 | Hs.9914 |
| FST | 207345_at | NM_006350.2 | 10468 | Hs.9914 |
| FSTL1 | 240891_at | NM_007085 | 11167 | Hs.433622 |
| FSTL1 | 208782_at | NM_007085.3 | 11167 | Hs.269512 |
| FSTL3 | 241921_x_at | AW015517 | 10272 | Hs.529038 |
| FSTL3 | 203592_s_at | NM_005860.2 | 10272 | Hs.529038 |
| FSTL4 | 214859_at | NM_015082.1 | 23105 | Hs.483390 |
| FSTL4 | 1564511_a_at | NM_015082.1 | 23105 | Hs.483390 |
| FSTL4 | 1565910_at | AK093331 | 23105 | Hs.123420 |
| FSTL5 | 232010_at | NM_020116.2 | 56884 | Hs.32452 |
| FTHL17 | 224379_at | NM_031894.1 | 53940 | Hs.333125 |
| FUCA2 | 223120_at | NM_032020.3 | 2519 | Hs.17680 |
| FURIN | 201945_at | NM_002569.2 | 5045 | Hs.513153 |
| FXYD1 | 205384_at | NM_005031.3 | 5348 | Hs.442498 |
| FXYD2 | 1556294_at | NM_001680 | 486 | Hs.413137 |
| FXYD2 | 205674_x_at | NM_021603.2 | 486 | Hs.413137 |
| FXYD2 | 207434_s_at | NM_001680.3 | 486 | Hs.413137 |
| FXYD3 | 202488_s_at | NM_021910.1 | 5349 | Hs.301350 |
| FXYD3 | 202489_s_at | NM_005971.2 | 5349 | Hs.301350 |
| FXYD4 | 231058_at | NM_173160.2 | 53828 | Hs.130497 |
| FXYD5 | 218084_x_at | NM_144779.1 | 53827 | Hs.333418 |
| FXYD5 | 224252_s_at | NM_014164.4 | 53827 | Hs.333418 |
| FXYD6 | 217897_at | NM_022003.1 | 53826 | Hs.504031 |
| FXYD6 | 240323_at | NM_022003 | 53826 | Hs.410748 |
| FXYD7 | 220131_at | NM_022006.1 | 53822 | Hs.134729 |
| FY | 208335_s_at | NM_002036.2 | 2532 | Hs.153381 |
| FZD1 | 204451_at | NM_003505.1 | 8321 | Hs.94234 |
| FZD1 | 204452_s_at | NM_003505.1 | 8321 | Hs.94234 |
| FZD10 | 219764_at | NM_007197.2 | 11211 | Hs.31664 |
| FZD2 | 238129_s_at | NM_001466 | 2535 | Hs.142912 |
| FZD2 | 210220_at | NM_001466.2 | 2535 | Hs.142912 |
| FZD3 | 219683_at | NM_017412.2 | 7976 | Hs.40735 |
| FZD4 | 218665_at | NM_012193.2 | 8322 | Hs.19545 |
| FZD4 | 224337_s_at | NM_012193.2 | 8322 | Hs.19545 |
| FZD4 | 229441_at | NM_012193 | 8322 | Hs.19545 |
| FZD5 | 206136_at | NM_003468.2 | 7855 | Hs.17631 |
| FZD6 | 203987_at | NM_003506.2 | 8323 | Hs.292464 |
| FZD7 | 203705_s_at | NM_003507.1 | 8324 | Hs.173859 |
| FZD7 | 203706_s_at | NM_003507.1 | 8324 | Hs.173859 |
| FZD8 | 216587_s_at | NM_031866.1 | 8325 | Hs.302634 |
| FZD8 | 224325_at | NM_031866.1 | 8325 | Hs.302634 |
| FZD8 | 227405_s_at | NM_031866.1 | 8325 | Hs.302634 |
| FZD9 | 207639_at | NM_003508.2 | 8326 | Hs.534367 |
| G1P2 | 205483_s_at | NM_005101.1 | 9636 | Hs.458485 |
| GABARAP | 200645_at | NM_007278.1 | 11337 | Hs.84359 |
| GABBR1 | 238569_at | NM_001470 | 2550 | Hs.167017 |
| GABBR1 | 203146_s_at | NM_021903.1 | 2550 | Hs.167017 |
| GABRA1 | 244118_at | NM_000806.3 | 2554 | Hs.175934 |
| GABRA1 | 206678_at | NM_000806.3 | 2554 | Hs.175934 |
| GABRA2 | 1554308_s_at | NM_000807 | 2555 | Hs.116250 |
| GABRA2 | 207014_at | NM_000807.1 | 2555 | Hs.116250 |
| GABRA3 | 207210_at | NM_000808.2 | 2556 | Hs.123024 |
| GABRA4 | 233437_at | NM_000809.2 | 2557 | Hs.248112 |
| GABRA4 | 208463_at | NM_000809.2 | 2557 | Hs.248112 |
| GABRA5 | 215531_s_at | NM_000810.2 | 2558 | Hs.24969 |
| GABRA5 | 217280_x_at | NM_000810.2 | 2558 | Hs.24969 |
| GABRA5 | 206456_at | NM_000810.2 | 2558 | Hs.24969 |
| GABRA6 | 207182_at | NM_000811.1 | 2559 | Hs.90791 |
| GABRB1 | 207010_at | NM_000812.2 | 2560 | Hs.27283 |
| GABRB2 | 207352_s_at | NM_000813.1 | 2561 | Hs.87083 |
| GABRB3 | 227690_at | NM_000814.4 | 2562 | Hs.302352 |
| GABRB3 | 227830_at | NM_000814.4 | 2562 | Hs.302352 |
| GABRB3 | 229724_at | NM_000814.4 | 2562 | Hs.302352 |
| GABRB3 | 1561316_at | NM_000814 | 2562 | Hs.302352 |
| GABRB3 | 1569689_s_at | NM_000814 | 2562 | Hs.302352 |
| GABRB3 | 205850_s_at | NM_000814.4 | 2562 | Hs.302352 |
| GABRD | 230255_at | NM_000815.2 | 2563 | Hs.113882 |
| GABRD | 208457_at | NM_000815.2 | 2563 | Hs.113882 |
| GABRE | 204537_s_at | NM_021984.2 | 2564 | Hs.22785 |
| GABRG1 | 241805_at | NM_173536.3 | 2565 | Hs.375051 |
| GABRG1 | 1552943_at | NM_173536.3 | 2565 | Hs.375051 |
| GABRG2 | 1568612_at | NM_198904.1 | 2566 | Hs.7195 |
| GABRG2 | 206849_at | NM_000816.2 | 2566 | Hs.7195 |
| GABRG3 | 216895_at | NM_033223 | 2567 | Hs.549509 |
| GABRG3 | 1555517_at | BC045709 | 2567 | - |
| GABRP | 205044_at | NM_014211.1 | 2568 | Hs.26225 |
| GABRQ | 220886_at | NM_018558.1 | 55879 | Hs.283081 |
| GABRR1 | 206525_at | NM_002042.2 | 2569 | Hs.437745 |
| GABRR2 | 208217_at | NM_002043.1 | 2570 | Hs.99927 |
| GABRR3 | 234410_at | XM_116036.7 | 200959 | Hs.534578 |
| GAD2 | 216651_s_at | NM_000818.1 | 2572 | Hs.231829 |
| GAD2 | 243265_at | NM_000818 | 2572 | Hs.231829 |
| GAD2 | 1568601_at | NM_000818 | 2572 | Hs.231829 |
| GAD2 | 206780_at | NM_000818.1 | 2572 | Hs.231829 |
| GAD2 | 211264_at | NM_000818 | 2572 | Hs.231829 |
| GAL | 214240_at | NM_015973.3 | 51083 | Hs.278959 |
| GAL | 207466_at | NM_015973 | 51083 | Hs.278959 |
| GAL3ST1 | 205670_at | NM_004861.1 | 9514 | Hs.17958 |
| GALNT8 | 220929_at | NM_017417.1 | 26290 | Hs.511985 |
| GALR1 | 220821_at | NM_001480.2 | 2587 | Hs.272191 |
| GALR2 | 211226_at | NM_003857.2 | 8811 | Hs.158351 |
| GALR3 | 33579_i_at | NM_003614 | 8484 |  |
| GAPVD1 | 214869_x_at | NM_015635.2 | 26130 | Hs.495134 |
| GAPVD1 | 212802_s_at | NM_015635.2 | 26130 | Hs.495134 |
| GAPVD1 | 212804_s_at | NM_015635.2 | 26130 | Hs.495134 |
| GAS6 | 1598_g_at | NM_000820.1 | 2621 | Hs.369201 |
| GAS6 | 202177_at | NM_000820.1 | 2621 | Hs.369201 |
| GAST | 208138_at | NM_000805.3 | 2520 | Hs.2681 |
| GBA2 | 223921_s_at | NM_020944.2 | 57704 | Hs.443134 |
| GBA2 | 224627_at | NM_020944.2 | 57704 | Hs.443134 |
| GBA2 | 228292_at | NM_020944 | 57704 | Hs.443134 |
| GBAS | 201816_s_at | NM_001483.1 | 2631 | Hs.437367 |
| GC | 204965_at | NM_000583.2 | 2638 | Hs.418497 |
| GCG | 206422_at | NM_002054.2 | 2641 | Hs.516494 |
| GCGR | 210565_at | NM_000160.1 | 2642 | Hs.208 |
| GDF10 | 206159_at | NM_004962.2 | 2662 | Hs.2171 |
| GDF11 | 216860_s_at | NM_005811.2 | 10220 | Hs.567259 |
| GDF11 | 217498_at | NM_005811 | 10220 | Hs.432439 |
| GDF11 | 226232_at | NM_005811 | 10220 | Hs.432439 |
| GDF11 | 226234_at | NM_005811 | 10220 | Hs.432439 |
| GDF15 | 221577_x_at | NM_004864.1 | 9518 | Hs.515258 |
| GDF2 | 221136_at | NM_016204.1 | 2658 | Hs.279463 |
| GDF3 | 220053_at | NM_020634.1 | 9573 | Hs.86232 |
| GDF5 | 206614_at | NM_000557.2 | 8200 | Hs.1573 |
| GDF8 | 207145_at | NM_005259.1 | 2660 | Hs.41565 |
| GDF9 | 221314_at | NM_005260 | 2661 | Hs.25022 |
| GDNF | 221359_at | NM_000514.2 | 2668 | Hs.248114 |
| GDPD1 | 1555606_a_at | NM_182569.1 | 284161 | Hs.153485 |
| GDPD1 | 1560227_at | NM_182569.1 | 284161 | Hs.153485 |
| GDPD2 | 220291_at | NM_017711.2 | 54857 | Hs.438712 |
| GDPD3 | 219722_s_at | NM_001031718.1 | 79153 | Hs.289015 |
| GENX-3414 | 1557575_at | NM_003943 | 8987 | Hs.109590 |
| GENX-3414 | 1570398_at | NM_003943 | 8987 | Hs.109590 |
| GENX-3414 | 203986_at | NM_003943.1 | 8987 | Hs.109590 |
| GFOD1 | 219821_s_at | NM_018988.1 | 54438 | Hs.484686 |
| GFOD2 | 221028_s_at | NM_030819.2 | 81577 | Hs.461030 |
| GFRA1 | 230163_at | NM_005264 | 2674 | Hs.444372 |
| GFRA1 | 205696_s_at | NM_005264.3 | 2674 | Hs.109425 |
| GFRA2 | 205721_at | NM_001495.4 | 2675 | Hs.561846 |
| GFRA2 | 205722_s_at | NM_001495.4 | 2675 | Hs.561846 |
| GFRA3 | 214479_at | NM_001496.3 | 2676 | Hs.58042 |
| GFRA3 | 229936_at | NM_001496.3 | 2676 | Hs.58042 |
| GFRA4 | 221199_at | NM_022139 | 64096 | Hs.302025 |
| GFRA4 | 234868_s_at | NM_022139 | 64096 | Hs.302025 |
| GGT1 | 215603_x_at | NM_001032364.1 | 2678 | Hs.355394 |
| GGT1 | 209918_at | NM_005265 | 2678 | Hs.352119 |
| GGT2 | 244179_x_at | XM_932672.1 | 645367 | Hs.444164 |
| GGT6 | 236225_at | NM_153338.1 | 124975 | Hs.130749 |
| GGTL3 | 226469_s_at | NM_052830.3 | 2686 | Hs.433738 |
| GGTL3 | 226470_at | NM_052830.3 | 2686 | Hs.433738 |
| GGTL3 | 226471_at | NM_052830.3 | 2686 | Hs.433738 |
| GGTLA1 | 205582_s_at | NM_004121.1 | 2687 | Hs.437156 |
| GH1 | 205840_x_at | NM_022559.2 | 2688 | Hs.500468 |
| GH1 | 206885_x_at | NM_022560.2 | 2688 | Hs.500468 |
| GH1 | 206886_x_at | NM_000515.3 | 2688 | Hs.500468 |
| GH1 | 208068_x_at | NM_000515.3 | 2688 | Hs.500468 |
| GH1 | 208069_x_at | NM_000515.3 | 2688 | Hs.500468 |
| GH1 | 211151_x_at | NM_022559.2 | 2688 | Hs.500468 |
| GHR | 205498_at | NM_000163.2 | 2690 | Hs.125180 |
| GHRH | 214524_at | NM_021081.3 | 2691 | Hs.37023 |
| GHRHR | 207825_s_at | NM_000823.2 | 2692 | Hs.767 |
| GHRHR | 211544_s_at | NM_000823.2 | 2692 | Hs.767 |
| GHRHR | 211545_at | NM_000823 | 2692 | Hs.767 |
| GHRL | 223862_at | NM_016362.2 | 51738 | Hs.302131 |
| GHRL | 237647_at | NM_016362 | 51738 | Hs.202345 |
| GHSR | 221360_s_at | NM_004122.1 | 2693 | Hs.248115 |
| GHSR | 224554_at | NM_198407.1 | 2693 | Hs.248115 |
| GIF | 207033_at | NM_005142.2 | 2694 | Hs.110014 |
| GIMAP2 | 232024_at | NM_015660.2 | 26157 | Hs.255008 |
| GIMAP5 | 215352_at | BC033688.1 | 55340 | Hs.412331 |
| GIMAP5 | 218805_at | NM_018384.3 | 55340 | Hs.412331 |
| GIMAP5 | 64064_at | NM_018384.3 | 55340 | Hs.412331 |
| GIP | 207899_at | NM_004123.2 | 2695 | Hs.1454 |
| GIPR | 231291_at | NM_000164 | 2696 | Hs.251412 |
| GIPR | 208105_at | NM_000164.2 | 2696 | Hs.558331 |
| GJA1 | 201667_at | NM_000165.2 | 2697 | Hs.74471 |
| GJA10 | 221415_s_at | NM_030772.3 | 81025 | Hs.546740 |
| GJA12 | 207025_at | NM_020435.2 | 57165 | Hs.100072 |
| GJA3 | 208590_x_at | NM_021954.2 | 2700 | Hs.533967 |
| GJA4 | 40687_at | NM_002060.2 | 2701 | Hs.296310 |
| GJA4 | 204904_at | NM_002060.2 | 2701 | Hs.296310 |
| GJA5 | 214466_at | NM_181703.1 | 2702 | Hs.447968 |
| GJA8 | 208489_at | NM_005267.3 | 2703 | Hs.157433 |
| GJB1 | 204973_at | NM_000166.2 | 2705 | Hs.333303 |
| GJB2 | 223278_at | NM_004004.3 | 2706 | Hs.524894 |
| GJB3 | 215243_s_at | NM_001005752.1 | 2707 | Hs.522561 |
| GJB3 | 205490_x_at | NM_024009.2 | 2707 | Hs.522561 |
| GJB3 | 205491_s_at | NM_024009 | 2707 | Hs.522561 |
| GJB4 | 216579_at | AK057628.1 | 127534 | Hs.351203 |
| GJB5 | 206156_at | NM_005268.2 | 2709 | Hs.198249 |
| GJB6 | 231771_at | NM_006783.2 | 10804 | Hs.511757 |
| GJC1 | 230025_at | NM_152219.2 | 125111 | Hs.444663 |
| GJC1 | 230606_at | NM_152219 | 125111 | Hs.135211 |
| GJC1 | 1553511_at | NM_152219.2 | 125111 | Hs.444663 |
| GJD2 | 221407_at | NM_020660.1 | 57369 | Hs.283816 |
| GJE1 | 234955_at | BC022382.1 | 401393 | Hs.382827 |
| GKN1 | 220191_at | NM_019617.3 | 56287 | Hs.69319 |
| GKN2 | 238222_at | NM_182536.2 | 200504 | Hs.16757 |
| GLA | 214430_at | NM_000169.1 | 2717 | Hs.69089 |
| GLB1L | 206540_at | NM_024506.3 | 79411 | Hs.181173 |
| GLDN | 230360_at | BX538105.1 | 342035 | Hs.526441 |
| GLDN | 232649_at | AK023623.1 | 342035 | Hs.526441 |
| GLIPR1 | 214085_x_at | NM_006851 | 11010 | Hs.553516 |
| GLIPR1 | 233515_at | NM_006851 | 11010 | Hs.401813 |
| GLIPR1 | 235038_at | NM_006851 | 11010 | Hs.401813 |
| GLIPR1 | 204221_x_at | NM_006851 | 11010 | Hs.553516 |
| GLIPR1 | 204222_s_at | NM_006851.1 | 11010 | Hs.553516 |
| GLP1R | 208390_s_at | NM_002062.2 | 2740 | Hs.389103 |
| GLP1R | 208391_s_at | NM_002062.2 | 2740 | Hs.389103 |
| GLP1R | 208400_at | NM_002062.2 | 2740 | Hs.389103 |
| GLP1R | 208401_s_at | NM_002062.2 | 2740 | Hs.389103 |
| GLP1R | 211232_x_at | NM_002062.2 | 2740 | Hs.389103 |
| GLP2R | 221312_at | NM_004246.1 | 9340 | Hs.248202 |
| GLRA1 | 207972_at | NM_000171.1 | 2741 | Hs.121490 |
| GLRA2 | 207462_at | NM_002063.2 | 2742 | Hs.2700 |
| GLRA3 | 216021_s_at | NM_006529 | 8001 | Hs.413099 |
| GLRA3 | 207928_s_at | NM_006529 | 8001 | Hs.413099 |
| GLRA3 | 210661_at | NM_006529 | 8001 | Hs.413099 |
| GLRB | 244680_at | NM_000824 | 2743 | Hs.32973 |
| GLRB | 205279_s_at | NM_000824.2 | 2743 | Hs.32973 |
| GLRB | 205280_at | NM_000824.2 | 2743 | Hs.32973 |
| GLT1D1 | 229770_at | NM_144669.1 | 144423 | Hs.12381 |
| GLT8D2 | 221447_s_at | NM_031302.2 | 83468 | Hs.567305 |
| GLT8D2 | 227070_at | NM_031302.2 | 83468 | Hs.567305 |
| GML | 208000_at | NM_002066.1 | 2765 | Hs.86161 |
| GNAS | 214157_at | NM_000516 | 2778 | Hs.157307 |
| GNAS | 214548_x_at | NM_000516.3 | 2778 | Hs.125898 |
| GNAS | 217057_s_at | NM_016592.1 | 2778 | Hs.125898 |
| GNAS | 217058_at | NM_000516 | 2778 | 125898 |
| GNAS | 217673_x_at | NM_000516 | 2778 | Hs.157307 |
| GNAS | 228173_at | NM_000516 | 2778 | Hs.157307 |
| GNAS | 229274_at | NM_000516 | 2778 | Hs.157307 |
| GNAS | 235851_s_at | NM_000516 | 2778 | Hs.157307 |
| GNAS | 239037_at | NM_000516 | 2778 | Hs.157307 |
| GNAS | 242816_at | NM_000516 | 2778 | Hs.157307 |
| GNAS | 242975_s_at | NM_000516 | 2778 | Hs.157307 |
| GNAS | 200780_x_at | NM_000516.3 | 2778 | Hs.125898 |
| GNAS | 200981_x_at | NM_000516.3 | 2778 | Hs.125898 |
| GNAS | 211858_x_at | NM_000516.3 | 2778 | Hs.125898 |
| GNAS | 212273_x_at | NM_000516.3 | 2778 | Hs.125898 |
| GNG10 | 201921_at | NM_001017998.2 | 2790 | Hs.534196 |
| GNG8 | 234284_at | NM_033258.1 | 94235 | Hs.283961 |
| GNGT2 | 235139_at | NM_031498.1 | 2793 | Hs.181781 |
| GNLY | 37145_at | NM_012483.1 | 10578 | Hs.105806 |
| GNLY | 205495_s_at | NM_012483.1 | 10578 | Hs.105806 |
| GNPTG | 224887_at | NM_032520.3 | 84572 | Hs.241575 |
| GNRH1 | 235540_at | NM_000825 | 2796 | Hs.82963 |
| GNRH1 | 207987_s_at | NM_000825.2 | 2796 | Hs.82963 |
| GNRH2 | 208519_x_at | NM_178332.1 | 2797 | Hs.129715 |
| GNRHR | 216341_s_at | NM_000406.2 | 2798 | Hs.407587 |
| GNRHR | 211522_s_at | NM_001012763.1 | 2798 | Hs.407587 |
| GNRHR | 211523_at | NM_000406.2 | 2798 | Hs.407587 |
| GNRHR2 | 1553067_a_at | NM_057163 | 114814 | Hs.356873 |
| GOLGA5 | 218241_at | NM_005113.2 | 9950 | Hs.104320 |
| GOLPH2 | 1558248_at | NM_016548 | 51280 | Hs.352662 |
| GOLPH2 | 217771_at | NM_016548.2 | 51280 | Hs.494337 |
| GOPC | 225022_at | NM_001017408.1 | 57120 | Hs.567333 |
| GOPC | 225023_at | NM_001017408.1 | 57120 | Hs.567333 |
| GOPC | 227214_at | NM_001017408.1 | 57120 | Hs.191539 |
| GOPC | 227215_at | NM_001017408.1 | 57120 | Hs.191539 |
| GOSR1 | 1555199_at | NM_004871 | 9527 | Hs.462680 |
| GOSR1 | 204630_s_at | NM_001007024.1 | 9527 | Hs.462680 |
| GOSR1 | 213020_at | NM_001007024.1 | 9527 | Hs.462680 |
| GOSR1 | 213021_at | NM_001007024.1 | 9527 | Hs.462680 |
| GOT2 | 200708_at | NM_002080.2 | 2806 | Hs.460929 |
| GP1BA | 207389_at | NM_000173.3 | 2811 | Hs.1472 |
| GP1BB | 206655_s_at | NM_000407.4 | 2812 | Hs.517410 |
| GP1BB | 209769_s_at | L20860.1 | 2812 | Hs.517410 |
| GP2 | 214324_at | NM_001007240.1 | 2813 | Hs.53985 |
| GP2 | 214325_at | NM_001502 | 2813 | Hs.53985 |
| GP2 | 206681_x_at | NM_001007240.1 | 2813 | Hs.53985 |
| GP2 | 208473_s_at | NM_001007240.1 | 2813 | Hs.53985 |
| GP5 | 207926_at | NM_004488.1 | 2814 | Hs.73734 |
| GP5 | 211525_s_at | NM_004488.1 | 2814 | Hs.73734 |
| GP6 | 220336_s_at | NM_016363.3 | 51206 | Hs.272216 |
| GP9 | 206883_x_at | NM_000174.2 | 2815 | Hs.1144 |
| GPA33 | 205929_at | NM_005814.1 | 10223 | Hs.437229 |
| GPAA1 | 215690_x_at | NM_003801.3 | 8733 | Hs.4742 |
| GPAA1 | 201618_x_at | NM_003801.3 | 8733 | Hs.4742 |
| GPAA1 | 211060_x_at | NM_003801.3 | 8733 | Hs.4742 |
| GPB5 | 1553945_at | NM_145171 | 122876 | Hs.375028 |
| GPBAR1 | 1552501_a_at | NM_170699.1 | 151306 | Hs.160954 |
| GPC1 | 202755_s_at | NM_002081.1 | 2817 | Hs.328232 |
| GPC1 | 202756_s_at | NM_002081.1 | 2817 | Hs.328232 |
| GPC2 | 239422_at | NM_152742.1 | 221914 | Hs.211701 |
| GPC3 | 234194_at | AK025597 | 2719 | 644108 |
| GPC3 | 234646_at | AK025597 | 2719 | 644108 |
| GPC3 | 209220_at | NM_004484.2 | 2719 | Hs.356794 |
| GPC4 | 204983_s_at | NM_001448.2 | 2239 | Hs.58367 |
| GPC4 | 204984_at | NM_001448.2 | 2239 | Hs.58367 |
| GPC5 | 207174_at | NM_004466.3 | 2262 | Hs.508364 |
| GPC6 | 223730_at | NM_005708.2 | 10082 | Hs.444329 |
| GPHA2 | 237230_at | NM_130769.2 | 170589 | Hs.436119 |
| GPI | 232002_at | NM_000175 | 2821 | Hs.406458 |
| GPI | 208308_s_at | NM_000175.2 | 2821 | Hs.466471 |
| GPI | 210848_at | NM_000175 | 2821 | Hs.406458 |
| GPIHBP1 | 238062_at | NM_178172.2 | 338328 | Hs.426410 |
| GPLD1 | 215554_at | NM_177483.1 | 2822 | Hs.512001 |
| GPLD1 | 217405_x_at | NM_001503 | 2822 | 591810 |
| GPLD1 | 206264_at | NM_001503.2 | 2822 | Hs.512001 |
| GPLD1 | 206265_s_at | NM_001503.2 | 2822 | Hs.512001 |
| GPLD1 | 206266_at | NM_001503 | 2822 | Hs.512001 |
| GPM6A | 236024_at | NM_201591.1 | 2823 | Hs.75819 |
| GPM6A | 209469_at | NM_005277.3 | 2823 | Hs.75819 |
| GPM6A | 209470_s_at | NM_005277.3 | 2823 | Hs.75819 |
| GPM6B | 240286_at | NM_005278 | 2824 | Hs.5422 |
| GPM6B | 209167_at | NM_005278 | 2824 | Hs.5422 |
| GPM6B | 209168_at | NM_001001995.1 | 2824 | Hs.495710 |
| GPM6B | 209169_at | NM_001001995.1 | 2824 | Hs.495710 |
| GPM6B | 209170_s_at | NM_001001994.1 | 2824 | Hs.495710 |
| GPNMB | 1554018_at | NM_002510 | 10457 | Hs.190495 |
| GPNMB | 201141_at | NM_001005340.1 | 10457 | Hs.190495 |
| GPR1 | 214605_x_at | NM_005279.2 | 2825 | Hs.184907 |
| GPR10 | 231805_at | NM_004248 | 2834 | Hs.248119 |
| GPR101 | 1553544_at | NM_054021.1 | 83550 | Hs.350569 |
| GPR103 | 1555533_at | NM_198179.1 | 84109 | Hs.368977 |
| GPR107 | 220264_s_at | NM_020960 | 57720 | Hs.512461 |
| GPR107 | 220265_at | NM_020960 | 57720 | Hs.512461 |
| GPR107 | 232657_at | NM_020960 | 57720 | Hs.442329 |
| GPR107 | 211977_at | NM_020960.3 | 57720 | Hs.512461 |
| GPR107 | 211979_at | NM_020960.3 | 57720 | Hs.555968 |
| GPR108 | 225058_at | AL365404 | 56927 | Hs.167641 |
| GPR109B | 205220_at | NM_006018.1 | 8843 | Hs.458425 |
| GPR110 | 220907_at | NM_025048.2 | 266977 | Hs.256897 |
| GPR110 | 235988_at | NM_153840 | 266977 | Hs.256897 |
| GPR110 | 238689_at | NM_153840.2 | 266977 | Hs.256897 |
| GPR111 | 1553036_at | NM_153839.2 | 222611 | Hs.381370 |
| GPR112 | 1553006_at | NM_153834.3 | 139378 | Hs.381354 |
| GPR113 | 236608_at | NM_153835 | 165082 | Hs.558674 |
| GPR113 | 1553016_at | NM_153835.1 | 165082 | Hs.558674 |
| GPR114 | 229971_at | NM_153837.1 | 221188 | Hs.187884 |
| GPR114 | 1555447_at | NM_153837 | 221188 | Hs.187884 |
| GPR115 | 237690_at | NM_153838 | 221393 | Hs.150131 |
| GPR115 | 1553031_at | NM_153838.2 | 221393 | Hs.150131 |
| GPR116 | 212950_at | NM_015234.3 | 221395 | Hs.362806 |
| GPR116 | 212951_at | NM_015234.3 | 221395 | Hs.362806 |
| GPR12 | 214558_at | NM_005288 | 2835 | Hs.123034 |
| GPR123 | 239221_at | NM_032422 | 84435 | Hs.435183 |
| GPR124 | 221814_at | NM_032777.6 | 25960 | Hs.17270 |
| GPR124 | 65718_at | NM_032777 | 25960 | Hs.17270 |
| GPR125 | 1555122_at | BC026009 | 166647 | Hs.99195 |
| GPR125 | 210473_s_at | NM_145290.2 | 166647 | Hs.99195 |
| GPR126 | 233887_at | NM_020455 | 57211 | Hs.419170 |
| GPR126 | 213094_at | NM_001032395.1 | 57211 | Hs.318894 |
| GPR128 | 1553296_at | NM_032787.1 | 84873 | Hs.334511 |
| GPR132 | 221140_s_at | NM_013345.2 | 29933 | Hs.532504 |
| GPR132 | 223887_at | NM_013345.2 | 29933 | Hs.532504 |
| GPR133 | 232267_at | AK131538.1 | 283383 | Hs.435168 |
| GPR135 | 214503_x_at | M76676.1 | 64582 | Hs.512691 |
| GPR135 | 217641_at | BC032831.2 | 64582 | Hs.512691 |
| GPR135 | 211659_at | AY288418.1 | 64582 | Hs.512691 |
| GPR137 | 219430_at | NM_020155.2 | 56834 | Hs.523763 |
| GPR137 | 221966_at | NM_020155.2 | 56834 | Hs.523763 |
| GPR137 | 43934_at | NM_020155.2 | 56834 | Hs.523763 |
| GPR143 | 206696_at | NM_000273.1 | 4935 | Hs.74124 |
| GPR144 | 216289_at | NM_182611 | 347088 | Hs.454099 |
| GPR145 | 224408_at | NM_001040179.1 | 84539 | Hs.558571 |
| GPR146 | 228770_at | NM_138445.2 | 115330 | Hs.553555 |
| GPR147 | 221387_at | NM_022146.1 | 64106 | Hs.302026 |
| GPR15 | 208524_at | NM_005290.1 | 2838 | Hs.506506 |
| GPR153 | 221902_at | CR627473.1 | 387509 | Hs.531581 |
| GPR153 | 64942_at | CR627473.1 | 387509 | Hs.531581 |
| GPR155 | 231166_at | NM_001033045.2 | 151556 | Hs.516604 |
| GPR155 | 239533_at | NM_001033045.2 | 151556 | Hs.516604 |
| GPR155 | 244509_at | NM_001033045.2 | 151556 | Hs.516604 |
| GPR156 | 239607_at | AB209362.1 | 165829 | Hs.333358 |
| GPR156 | 1553028_at | NM_153002.1 | 165829 | Hs.333358 |
| GPR157 | 220901_at | BC018691.1 | 80045 | Hs.287490 |
| GPR157 | 227970_at | AK026883.1 | 80045 | Hs.287490 |
| GPR158 | 232195_at | NM_020752.2 | 57512 | Hs.499108 |
| GPR160 | 223423_at | NM_014373.1 | 26996 | Hs.231320 |
| GPR161 | 214104_at | NM_153832.1 | 23432 | Hs.271809 |
| GPR161 | 230369_at | AK091271.1 | 23432 | Hs.271809 |
| GPR161 | 235961_at | NM_153832.1 | 23432 | Hs.271809 |
| GPR161 | 206971_at | NM_007369.1 | 23432 | Hs.271809 |
| GPR161 | 206972_s_at | NM_007369.1 | 23432 | Hs.271809 |
| GPR162 | 205056_s_at | NM_019858.1 | 27239 | Hs.418105 |
| GPR17 | 215225_s_at | NM_005291.1 | 2840 | Hs.46453 |
| GPR17 | 206190_at | NM_005291.1 | 2840 | Hs.46453 |
| GPR171 | 207651_at | NM_013308.2 | 29909 | Hs.549152 |
| GPR172A | 218151_x_at | NM_024531.3 | 79581 | Hs.6459 |
| GPR172A | 222155_s_at | NM_024531.3 | 79581 | Hs.6459 |
| GPR172B | 220756_s_at | NM_017986.2 | 55065 | Hs.110128 |
| GPR173 | 221299_at | NM_018969.3 | 54328 | Hs.283023 |
| GPR174 | 224285_at | NM_032553.1 | 84636 | Hs.326713 |
| GPR175 | 218855_at | NM_016372.1 | 131601 | Hs.6418 |
| GPR176 | 227846_at | BC067106.1 | 11245 | Hs.37196 |
| GPR176 | 206673_at | NM_007223.1 | 11245 | Hs.37196 |
| GPR18 | 210279_at | NM_005292.2 | 2841 | Hs.558333 |
| GPR180 | 231871_at | NM_180989.4 | 160897 | Hs.159422 |
| GPR19 | 207183_at | NM_006143.1 | 2842 | Hs.92458 |
| GPR20 | 214510_at | NM_005293.2 | 2843 | Hs.188859 |
| GPR21 | 221294_at | NM_005294.1 | 2844 | Hs.368372 |
| GPR22 | 221288_at | NM_005295.2 | 2845 | Hs.567237 |
| GPR22 | 244493_at | BC045823.1 | 2845 | Hs.567237 |
| GPR23 | 206960_at | NM_005296.1 | 2846 | Hs.522701 |
| GPR24 | 221354_s_at | NM_005297.3 | 2847 | Hs.248122 |
| GPR24 | 223855_s_at | NM_005297.3 | 2847 | Hs.248122 |
| GPR24 | 230498_at | NM_005297.3 | 2847 | Hs.248122 |
| GPR25 | 221363_x_at | NM_005298.2 | 2848 | Hs.534316 |
| GPR26 | 244617_at | NM_153442.1 | 2849 | Hs.12751 |
| GPR27 | 221306_at | NM_018971.1 | 2850 | Hs.553493 |
| GPR27 | 227769_at | NM_018971 | 2850 | Hs.356084 |
| GPR3 | 214613_at | NM_005281.2 | 2827 | Hs.66542 |
| GPR30 | 210640_s_at | NM_001039966.1 | 2852 | Hs.20961 |
| GPR30 | 211829_s_at | NM_001039966.1 | 2852 | Hs.20961 |
| GPR31 | 208556_at | NM_005299.1 | 2853 | Hs.248124 |
| GPR32 | 221469_at | NM_001506.1 | 2854 | Hs.515555 |
| GPR34 | 223620_at | NM_001033514.1 | 2857 | Hs.495989 |
| GPR35 | 210264_at | NM_005301.2 | 2859 | Hs.239891 |
| GPR37 | 214586_at | NM_005302 | 2861 | Hs.406094 |
| GPR37 | 209631_s_at | NM_005302.2 | 2861 | Hs.406094 |
| GPR37L1 | 207055_at | NM_004767.2 | 9283 | Hs.132049 |
| GPR39 | 229104_s_at | AK122643.1 | 2863 | Hs.432395 |
| GPR39 | 229105_at | AK122643.1 | 2863 | Hs.432395 |
| GPR39 | 208600_s_at | NM_001508.1 | 2863 | Hs.432395 |
| GPR4 | 206236_at | NM_005282.1 | 2828 | Hs.17170 |
| GPR4 | 211266_s_at | NM_005282.1 | 2828 | Hs.17170 |
| GPR40 | 1561507_at | NM_005303 | 2864 | Hs.248127 |
| GPR40 | 231761_at | NM_005303.1 | 2864 | Hs.248127 |
| GPR41 | 221385_s_at | NM_005304.2 | 2865 | Hs.248055 |
| GPR43 | 221345_at | NM_005306.1 | 2867 | Hs.248056 |
| GPR44 | 216464_x_at | NM_004778.1 | 11251 | Hs.299567 |
| GPR44 | 206361_at | NM_004778.1 | 11251 | Hs.299567 |
| GPR45 | 211175_at | NM_007227.3 | 11250 | Hs.129701 |
| GPR50 | 208311_at | NM_004224.1 | 9248 | Hs.158329 |
| GPR51 | 209990_s_at | NM_005458.5 | 9568 | Hs.198612 |
| GPR51 | 209991_x_at | NM_005458.5 | 9568 | Hs.198612 |
| GPR51 | 211679_x_at | NM_005458.5 | 9568 | Hs.198612 |
| GPR51 | 217077_s_at | NM_005458.5 | 9568 | Hs.198612 |
| GPR52 | 221313_at | NM_005684.2 | 9293 | Hs.567256 |
| GPR54 | 242517_at | NM_032551.3 | 84634 | Hs.208229 |
| GPR55 | 231745_at | NM_005683.2 | 9290 | Hs.114545 |
| GPR55 | 237559_at | NM_005683.2 | 9290 | Hs.114545 |
| GPR56 | 206582_s_at | NM_201524.1 | 9289 | Hs.513633 |
| GPR56 | 212070_at | NM_005682.4 | 9289 | Hs.513633 |
| GPR6 | 214655_at | NM_005284.3 | 2830 | Hs.46332 |
| GPR6 | 1553507_a_at | NM_005284.3 | 2830 | Hs.46332 |
| GPR61 | 224531_at | NM_031936.3 | 83873 | Hs.514690 |
| GPR61 | 244618_at | NM_031936 | 83873 | Hs.56276 |
| GPR62 | 1554559_at | NM_080865.2 | 118442 | Hs.232213 |
| GPR63 | 220993_s_at | NM_030784.1 | 81491 | Hs.485990 |
| GPR64 | 206002_at | NM_005756.1 | 10149 | Hs.146978 |
| GPR65 | 214467_at | NM_003608.2 | 8477 | Hs.513440 |
| GPR68 | 229055_at | NM_003485.3 | 8111 | Hs.8882 |
| GPR68 | 211249_at | NM_003485.3 | 8111 | Hs.8882 |
| GPR7 | 231752_at | NM_005285 | 2831 | Hs.248117 |
| GPR73L1 | 234450_at | NM_144773.2 | 128674 | Hs.375029 |
| GPR74 | 224169_at | NM_004885.1 | 10886 | Hs.99231 |
| GPR75 | 220481_at | NM_006794.1 | 10936 | Hs.21691 |
| GPR77 | 221149_at | NM_018485.1 | 27202 | Hs.534412 |
| GPR78 | 1553063_at | NM_080819.2 | 27201 | Hs.350588 |
| GPR8 | 221358_at | NM_005286.2 | 2832 | Hs.248118 |
| GPR81 | 224131_at | NM_032554.3 | 27198 | Hs.524811 |
| GPR82 | 1553316_at | NM_080817.2 | 27197 | Hs.567268 |
| GPR82 | 1553317_s_at | NM_080817 | 27197 | Hs.567268 |
| GPR83 | 222953_at | NM_016540.2 | 10888 | Hs.272385 |
| GPR84 | 223767_at | NM_020370.1 | 53831 | Hs.306199 |
| GPR85 | 219898_at | NM_018970.3 | 54329 | Hs.152009 |
| GPR85 | 234303_s_at | NM_018970.3 | 54329 | Hs.152009 |
| GPR87 | 219936_s_at | NM_023915.2 | 53836 | Hs.58561 |
| GPR88 | 220313_at | NM_022049.1 | 54112 | Hs.170053 |
| GPR92 | 230252_at | NM_020400.4 | 57121 | Hs.155538 |
| GPR92 | 231788_at | NM_020400.4 | 57121 | Hs.155538 |
| GPR97 | 220404_at | NM_170776.3 | 222487 | Hs.383403 |
| GPR97 | 1553723_at | NM_170776.3 | 222487 | Hs.383403 |
| GPRC5A | 203108_at | NM_003979.3 | 9052 | Hs.194691 |
| GPRC5A | 212444_at | NM_003979 | 9052 | Hs.194691 |
| GPRC5B | 225511_at | NM_153208 | 124152 | Hs.306382 |
| GPRC5B | 228313_at | NM_016235 | 51704 | Hs.242407 |
| GPRC5B | 203631_s_at | NM_016235.1 | 51704 | Hs.148685 |
| GPRC5B | 203632_s_at | NM_016235.1 | 51704 | Hs.148685 |
| GPRC5C | 219327_s_at | NM_022036.2 | 55890 | Hs.446438 |
| GPRC5C | 230907_at | NM_018653 | 55890 | Hs.446438 |
| GPRC5D | 221297_at | NM_018654.1 | 55507 | Hs.127718 |
| GPRC6A | 1552775_at | NM_148963.1 | 222545 | Hs.266745 |
| GPX3 | 214091_s_at | NM_002084.3 | 2878 | Hs.386793 |
| GPX3 | 201348_at | NM_002084.3 | 2878 | Hs.386793 |
| GPX5 | 208028_s_at | NM_003996.2 | 2880 | Hs.248129 |
| GPX7 | 213170_at | NM_015696.2 | 2882 | Hs.43728 |
| GRAMD1A | 224807_at | NM_020895.2 | 57655 | Hs.515351 |
| GRAMD1B | 212906_at | BC011969.1 | 57476 | Hs.144725 |
| GRAMD1C | 219313_at | NM_017577.2 | 54762 | Hs.24583 |
| GRAMD2 | 229616_s_at | NM_001012642.1 | 196996 | Hs.412093 |
| GREM1 | 218468_s_at | NM_013372.5 | 26585 | Hs.40098 |
| GREM1 | 218469_at | NM_013372.5 | 26585 | Hs.40098 |
| GREM2 | 220794_at | NM_022469.3 | 64388 | Hs.98206 |
| GREM2 | 235504_at | NM_022469.3 | 64388 | Hs.98206 |
| GREM2 | 240509_s_at | NM_022469.3 | 64388 | Hs.98206 |
| GRIA1 | 209793_at | NM_000827 | 2890 | Hs.519693 |
| GRIA1 | 211520_s_at | NM_000827.2 | 2890 | Hs.519693 |
| GRIA2 | 236538_at | NM_000826.2 | 2891 | Hs.32763 |
| GRIA2 | 241172_at | NM_000826 | 2891 | Hs.32763 |
| GRIA2 | 205358_at | NM_000826.2 | 2891 | Hs.32763 |
| GRIA3 | 217565_at | NM_000828 | 2892 | Hs.377070 |
| GRIA3 | 1569290_s_at | NM_000828.2 | 2892 | Hs.377070 |
| GRIA3 | 206730_at | NM_007325.2 | 2892 | Hs.377070 |
| GRIA3 | 208032_s_at | NM_007325.2 | 2892 | Hs.377070 |
| GRIA4 | 238663_x_at | NM_000829 | 2893 | Hs.503743 |
| GRIA4 | 1569178_at | NM_000829 | 2893 | Hs.113943 |
| GRIA4 | 208464_at | NM_000829.1 | 2893 | Hs.503743 |
| GRID1 | 231977_at | NM_017551.1 | 2894 | Hs.530653 |
| GRID1 | 1555267_at | NM_017551.1 | 2894 | Hs.530653 |
| GRID1 | 1555268_a_at | NM_017551.1 | 2894 | Hs.530653 |
| GRID2 | 221364_at | NM_001510.1 | 2895 | Hs.480281 |
| GRIK1 | 1560378_at | NM_000830 | 2897 | Hs.222405 |
| GRIK1 | 207242_s_at | NM_000830.3 | 2897 | Hs.473554 |
| GRIK2 | 213845_at | NM_021956 | 2898 | 98262 |
| GRIK2 | 215655_at | NM_021956 | 2898 | Hs.307494 |
| GRIK2 | 1555375_at | NM_021956 | 2898 | Hs.98262 |
| GRIK2 | 1560142_at | NM_175768.1 | 2898 | Hs.98262 |
| GRIK2 | 1560265_at | NM_021956 | 2898 | Hs.307494 |
| GRIK2 | 1563754_at | NM_021956 | 2898 | Hs.98262 |
| GRIK3 | 207454_at | NM_000831.2 | 2899 | Hs.2389 |
| GRIK4 | 242215_at | NM_014619 | 2900 | Hs.222901 |
| GRIK4 | 208552_at | NM_014619.2 | 2900 | Hs.533740 |
| GRIK5 | 214966_at | NM_002088.3 | 2901 | Hs.367799 |
| GRIK5 | 216691_at | NM_002088 | 2901 | Hs.367799 |
| GRIK5 | 217509_x_at | NM_002088.3 | 2901 | Hs.367799 |
| GRIK5 | 1553168_at | NM_002088 | 2901 | Hs.367799 |
| GRIN1 | 205914_s_at | NM_007327.1 | 2902 | Hs.558334 |
| GRIN1 | 205915_x_at | NM_007327.1 | 2902 | Hs.558334 |
| GRIN1 | 210781_x_at | NM_007327.1 | 2902 | Hs.558334 |
| GRIN1 | 210782_x_at | NM_000832.4 | 2902 | Hs.558334 |
| GRIN1 | 211125_x_at | NM_000832.4 | 2902 | Hs.558334 |
| GRIN2A | 206534_at | NM_000833.2 | 2903 | Hs.411472 |
| GRIN2B | 210411_s_at | NM_000834.2 | 2904 | Hs.445066 |
| GRIN2B | 210412_at | NM_000834.2 | 2904 | Hs.445066 |
| GRIN2C | 217573_at | NM_000835 | 2905 | Hs.436980 |
| GRIN2C | 1569268_at | NM_000835 | 2905 | Hs.436980 |
| GRIN2C | 210400_at | NM_000835.3 | 2905 | Hs.436980 |
| GRIN2D | 229883_at | AB209292.1 | 2906 | Hs.445015 |
| GRIN2D | 207036_x_at | NM_000836.1 | 2906 | Hs.445015 |
| GRIN3A | 233171_at | NM_133445.1 | 116443 | Hs.556011 |
| GRIN3A | 233220_at | NM_133445.1 | 116443 | Hs.556011 |
| GRIN3B | 233892_at | NM_138690.1 | 116444 | Hs.558600 |
| GRINA | 212090_at | NM_000837.1 | 2907 | Hs.339697 |
| GRM1 | 207299_s_at | NM_000838.2 | 2911 | Hs.32945 |
| GRM1 | 210939_s_at | NM_000838.2 | 2911 | Hs.32945 |
| GRM1 | 210940_s_at | NM_000838.2 | 2911 | Hs.32945 |
| GRM2 | 241802_x_at | NM_000839.2 | 2912 | Hs.121510 |
| GRM2 | 208465_at | NM_000839.2 | 2912 | Hs.121510 |
| GRM3 | 205814_at | NM_000840.2 | 2913 | Hs.112621 |
| GRM4 | 210234_at | NM_000841.1 | 2914 | Hs.429018 |
| GRM5 | 1565389_s_at | NM_000842.1 | 2915 | Hs.147361 |
| GRM5 | 207235_s_at | NM_000842.1 | 2915 | Hs.147361 |
| GRM6 | 208035_at | NM_000843.2 | 2916 | Hs.248131 |
| GRM7 | 217008_s_at | NM_181874.1 | 2917 | Hs.475336 |
| GRM7 | 241049_at | NM_000844 | 2917 | Hs.83407 |
| GRM7 | 207548_at | NM_000844.2 | 2917 | Hs.475336 |
| GRM8 | 216255_s_at | NM_000845.1 | 2918 | Hs.449625 |
| GRM8 | 216256_at | NM_000845 | 2918 | Hs.449625 |
| GRM8 | 216992_s_at | NM_000845.1 | 2918 | Hs.449625 |
| GRP | 231432_at | NM_002091 | 2922 | Hs.153444 |
| GRP | 206326_at | NM_001012513.1 | 2922 | Hs.153444 |
| GRPR | 207929_at | NM_005314.2 | 2925 | Hs.567238 |
| GSN | 214040_s_at | NM_000177.4 | 2934 | Hs.522373 |
| GSN | 227957_at | NM_000177 | 2934 | Hs.446537 |
| GSN | 227958_s_at | NM_000177 | 2934 | Hs.446537 |
| GSN | 1564525_at | NM_000177 | 2934 | Hs.446537 |
| GSN | 200696_s_at | NM_000177.4 | 2934 | Hs.522373 |
| GUCA2A | 207003_at | NM_033553.2 | 2980 | Hs.778 |
| GUCA2B | 207502_at | NM_007102.1 | 2981 | Hs.32966 |
| GUCY2C | 206312_at | NM_004963.1 | 2984 | Hs.524278 |
| GUCY2D | 207884_at | NM_000180.1 | 3000 | Hs.309958 |
| GUCY2F | 208053_at | NM_001522.1 | 2986 | Hs.123074 |
| GYG2 | 215695_s_at | NM_003918.1 | 8908 | Hs.541209 |
| GYG2 | 210963_s_at | NM_003918.1 | 8908 | Hs.541209 |
| GYG2 | 210964_s_at | NM_003918.1 | 8908 | Hs.541209 |
| GYPA | 1559520_at | NM_002099 | 2993 | Hs.34287 |
| GYPA | 205837_s_at | NM_002099.3 | 2993 | Hs.371903 |
| GYPA | 205838_at | NM_002099.3 | 2993 | Hs.371903 |
| GYPA | 211820_x_at | NM_002099.3 | 2993 | Hs.371903 |
| GYPA | 211821_x_at | NM_002099.3 | 2993 | Hs.371903 |
| GYPB | 214407_x_at | NM_002100.3 | 2994 | Hs.434973 |
| GYPB | 216833_x_at | U05255.1 | 2994 | Hs.434973 |
| GYPB | 207459_x_at | NM_002100 | 2994 | Hs.434973 |
| GYPC | 202947_s_at | NM_002101.3 | 2995 | Hs.59138 |
| GYPE | 216398_at | NM_002102 | 2996 | Hs.395535 |
| GYPE | 1561136_at | AF085899.1 | 2996 | Hs.371903 |
| GYPE | 1561137_s_at | AF085899.1 | 2996 | Hs.371903 |
| GYPE | 207854_at | NM_002102.2 | 2996 | Hs.371903 |
| GZMA | 205488_at | NM_006144.2 | 3001 | Hs.90708 |
| GZMK | 206666_at | NM_002104.2 | 3003 | Hs.277937 |
| GZMM | 207460_at | NM_005317.2 | 3004 | Hs.465511 |
| HABP2 | 206010_at | NM_004132.2 | 3026 | Hs.422542 |
| HADH2 | 202282_at | NM_004493.2 | 3028 | Hs.171280 |
| HAMP | 220491_at | NM_021175.2 | 57817 | Hs.8821 |
| HAPLN1 | 205523_at | NM_001884.2 | 1404 | Hs.2799 |
| HAPLN1 | 205524_s_at | NM_001884.2 | 1404 | Hs.2799 |
| HAPLN2 | 220142_at | NM_021817.2 | 60484 | Hs.410719 |
| HAPLN3 | 227262_at | NM_178232.2 | 145864 | Hs.447530 |
| HAPLN4 | 235420_at | NM_023002.1 | 404037 | Hs.367829 |
| HAS1 | 207316_at | NM_001523.1 | 3036 | Hs.57697 |
| HAS2 | 230372_at | NM_005328 | 3037 | Hs.159226 |
| HAS2 | 206432_at | NM_005328.1 | 3037 | Hs.159226 |
| HAS3 | 223541_at | NM_005329.2 | 3038 | Hs.85962 |
| HAS3 | 1552980_at | NM_138612.1 | 3038 | Hs.85962 |
| HAVCR1 | 207052_at | NM_012206.1 | 26762 | Hs.129711 |
| HAVCR2 | 235458_at | NM_032782.3 | 84868 | Hs.155111 |
| HAVCR2 | 1554285_at | NM_032782.3 | 84868 | Hs.155111 |
| HAVCR2 | 1555628_a_at | NM_032782.3 | 84868 | Hs.155111 |
| HAVCR2 | 1555629_at | NM_032782 | 84868 | Hs.155111 |
| HBEGF | 244857_at | NM_001945 | 1839 | Hs.799 |
| HBEGF | 38037_at | NM_001945.1 | 1839 | Hs.799 |
| HBEGF | 203821_at | NM_001945.1 | 1839 | Hs.799 |
| HCFC2 | 219484_at | NM_013320.1 | 29915 | Hs.506558 |
| HCN1 | 1562563_at | NM_021072 | 348980 | Hs.353176 |
| HCN2 | 214893_x_at | NM_001194.2 | 610 | Hs.124161 |
| HCN2 | 207592_s_at | NM_001194.2 | 610 | Hs.124161 |
| HCN3 | 222078_at | BF110802 | 57657 | Hs.284171 |
| HCN3 | 228741_s_at | NM_020897.1 | 57657 | Hs.284171 |
| HCN4 | 206946_at | NM_005477.1 | 10021 | Hs.160264 |
| HCRTR1 | 207619_at | NM_001525.1 | 3061 | Hs.388226 |
| HCRTR2 | 207393_at | NM_001526.2 | 3062 | Hs.151624 |
| HCST | 223640_at | NM_014266.3 | 10870 | Hs.117339 |
| HDGF | 216484_x_at | NM_004494.1 | 3068 | Hs.506748 |
| HDGF | 200896_x_at | NM_004494.1 | 3068 | Hs.506748 |
| HDLBP | 219674_s_at | AF116718.1 | 55498 | Hs.471851 |
| HDLBP | 221767_x_at | NM_005336 | 3069 | Hs.427152 |
| HDLBP | 222916_s_at | NM_005336 | 3069 | Hs.471851 |
| HDLBP | 225012_at | NM_005336 | 3069 | Hs.471851 |
| HDLBP | 235624_at | NM_005336 | 3069 | Hs.427152 |
| HDLBP | 200643_at | NM_005336.2 | 3069 | Hs.471851 |
| HEG1 | 212822_at | XM_087386.8 | 57493 | Hs.477420 |
| HEG1 | 213069_at | XM_087386.8 | 57493 | Hs.477420 |
| HEPH | 203902_at | NM_014799 | 9843 | Hs.31720 |
| HEPH | 203903_s_at | NM_138737.1 | 9843 | Hs.31720 |
| HERPUD2 | 222751_at | NM_022373.3 | 64224 | Hs.424711 |
| HERPUD2 | 1552628_a_at | NM_022373.3 | 64224 | Hs.424711 |
| HERPUD2 | 1558699_a_at | NM_022373.3 | 64224 | Hs.424711 |
| HFE | 214647_s_at | NM_000410.3 | 3077 | Hs.233325 |
| HFE | 235754_at | NM_000410 | 3077 | Hs.233325 |
| HFE | 1553402_a_at | NM_000410.3 | 3077 | Hs.233325 |
| HFE | 206086_x_at | NM_139008.2 | 3077 | Hs.233325 |
| HFE | 206087_x_at | NM_000410 | 3077 | Hs.233325 |
| HFE | 210864_x_at | NM_139005.2 | 3077 | Hs.233325 |
| HFE | 211326_x_at | NM_000410 | 3077 | Hs.233325 |
| HFE | 211327_x_at | NM_000410.3 | 3077 | Hs.233325 |
| HFE | 211328_x_at | NM_139005.2 | 3077 | Hs.233325 |
| HFE | 211329_x_at | NM_000410.3 | 3077 | Hs.233325 |
| HFE | 211330_s_at | NM_000410.3 | 3077 | Hs.233325 |
| HFE | 211331_x_at | NM_139005.2 | 3077 | Hs.233325 |
| HFE | 211332_x_at | NM_139005.2 | 3077 | Hs.233325 |
| HFE | 211863_x_at | NM_139008.2 | 3077 | Hs.233325 |
| HFE | 211866_x_at | NM_139006.2 | 3077 | Hs.233325 |
| HFE2 | 228621_at | NM_145277.3 | 148738 | Hs.511850 |
| HGF | 209961_s_at | NM_000601.4 | 3082 | Hs.396530 |
| HGF | 210755_at | NM_001010934.1 | 3082 | Hs.396530 |
| HGF | 210997_at | NM_001010931.1 | 3082 | Hs.396530 |
| HGF | 210998_s_at | NM_000601.4 | 3082 | Hs.396530 |
| HGFAC | 207027_at | NM_001528.2 | 3083 | Hs.104 |
| HHIP | 223775_at | NM_022475.1 | 64399 | Hs.507991 |
| HHIP | 237466_s_at | NM_022475.1 | 64399 | Hs.507991 |
| HHIP | 1556037_s_at | NM_022475.1 | 64399 | Hs.507991 |
| HHLA2 | 220812_s_at | NM_007072.2 | 11148 | Hs.252351 |
| HHLA2 | 234624_at | AK026893 | 11148 | 252351 |
| HHLA2 | 234673_at | NM_007072 | 11148 | Hs.252351 |
| HIAT1 | 225222_at | NM_033055.2 | 64645 | Hs.124156 |
| HIAT1 | 231895_at | NM_033055 | 64645 | Hs.21015 |
| HIATL1 | 223073_at | NM_032558.2 | 84641 | Hs.555996 |
| HIATL1 | 224078_at | NM_032558.2 | 84641 | Hs.555996 |
| HIF1A | 238869_at |  | 3091 |  |
| HIF1A | 200989_at | NM_001530.2 | 3091 | Hs.509554 |
| HIGD1A | 217845_x_at | NM_014056.1 | 25994 | Hs.562430 |
| HIGD1A | 221896_s_at | NM_014056.1 | 25994 | Hs.7917 |
| HIGD1B | 219719_at | NM_016438.2 | 51751 | Hs.287963 |
| HIGD2A | 209329_x_at | NM_138820.2 | 192286 | Hs.534575 |
| HLA-A | 213932_x_at | XM_945079.1 | 649853 | Hs.181244 |
| HLA-A | 215313_x_at | NM_002116.5 | 3105 | Hs.181244 |
| HLA-B | 208729_x_at | NM_005514.5 | 3106 | Hs.534125 |
| HLA-B | 209140_x_at | NM_005514.5 | 3106 | Hs.534125 |
| HLA-C | 214459_x_at | NM_002117 | 3107 | Hs.534125 |
| HLA-C | 216526_x_at | NM_002117 | 3107 | Hs.534125 |
| HLA-C | 208812_x_at | NM_002117 | 3107 | Hs.534125 |
| HLA-C | 211799_x_at | NM_002117 | 3107 | Hs.534125 |
| HLA-C | 211911_x_at | AY732487.1 | 3107 | Hs.534125 |
| HLA-DMA | 217478_s_at | NM_006120.2 | 3108 | Hs.351279 |
| HLA-DMB | 203932_at | NM_002118.3 | 3109 | Hs.1162 |
| HLA-DOA | 216946_at | NM_002119 | 3111 | Hs.351874 |
| HLA-DOA | 217001_x_at | NM_002119 | 3111 | Hs.351874 |
| HLA-DOA | 226878_at | NM_002119.3 | 3111 | Hs.351874 |
| HLA-DOA | 206313_at | NM_002119.3 | 3111 | Hs.351874 |
| HLA-DOA | 211142_x_at | NM_002119 | 3111 | Hs.351874 |
| HLA-DOB | 1554984_a_at | NM_002120 | 3112 | Hs.1802 |
| HLA-DOB | 205671_s_at | NM_002120.2 | 3112 | Hs.1802 |
| HLA-DPA1 | 213537_at | NM_033554 | 3113 | Hs.914 |
| HLA-DPA1 | 211990_at | NM_033554.2 | 3113 | Hs.347270 |
| HLA-DPA1 | 211991_s_at | NM_033554.2 | 3113 | Hs.347270 |
| HLA-DPB1 | 244485_at | NM_002121 | 3115 | Hs.368409 |
| HLA-DPB1 | 201137_s_at | NM_002121 | 3115 | Hs.485130 |
| HLA-DQA1 | 213831_at | NM_002122.3 | 3117 | Hs.387679 |
| HLA-DQA1 | 236203_at | AB209628.1 | 3117 | Hs.387679 |
| HLA-DQA1 | 203290_at | NM_002122 | 3117 | Hs.387679 |
| HLA-DQA1 | 212671_s_at | NM_002122.3 | 3117 | Hs.387679 |
| HLA-DQB1 | 209480_at | XM_942240.1 | 650557 | Hs.409934 |
| HLA-DQB1 | 209823_x_at | NM_002123.2 | 3119 | Hs.409934 |
| HLA-DQB1 | 210747_at | NM_002123 | 3119 | Hs.409934 |
| HLA-DQB1 | 211654_x_at | XM_942240.1 | 650557 | Hs.409934 |
| HLA-DQB1 | 211656_x_at | NM_002123 | 3119 | Hs.409934 |
| HLA-DQB1 | 212998_x_at | NM_002123.2 | 3119 | Hs.409934 |
| HLA-DQB1 | 212999_x_at | XM_945818.1 | 650557 | Hs.409934 |
| HLA-DRA | 208894_at | NM_019111.3 | 3122 | Hs.520048 |
| HLA-DRA | 210982_s_at | NM_019111.3 | 3122 | Hs.520048 |
| HLA-DRB1 | 209312_x_at | NM_002124.1 | 3123 | Hs.520049 |
| HLA-DRB3 | 221491_x_at | NM_022555 | 3125 | Hs.308026 |
| HLA-DRB3 | 238900_at | BE669692 |  |  |
| HLA-DRB4 | 215666_at | NM_021983 | 3126 | Hs.520049 |
| HLA-DRB4 | 215669_at | NM_021983 | 3126 | Hs.449633 |
| HLA-DRB4 | 208306_x_at | NM_021983 | 3126 | Hs.449633 |
| HLA-DRB4 | 209728_at | NM_021983.4 | 3126 | Hs.520049 |
| HLA-DRB5 | 215193_x_at | NM_002125.3 | 3127 | Hs.534322 |
| HLA-DRB5 | 204670_x_at | X99895.1 | 3127 | Hs.520049 |
| HLA-E | 217456_x_at | NM_005516 | 3133 | Hs.381008 |
| HLA-E | 200904_at | NM_005516 | 3133 | Hs.381008 |
| HLA-E | 200905_x_at | NM_005516.4 | 3133 | Hs.381008 |
| HLA-F | 221875_x_at | NM_018950.1 | 3134 | Hs.519972 |
| HLA-F | 221978_at | NM_018950 | 3134 | Hs.411958 |
| HLA-F | 222279_at | AK092748.1 | 285830 | Hs.309247 |
| HLA-F | 204806_x_at | NM_018950.1 | 3134 | Hs.519972 |
| HLA-G | 210514_x_at | NM_002127.3 | 3135 | Hs.512152 |
| HLA-G | 211528_x_at | NM_002127.3 | 3135 | Hs.512152 |
| HLA-G | 211529_x_at | NM_002127.3 | 3135 | Hs.512152 |
| HLA-G | 211530_x_at | NM_002127.3 | 3135 | Hs.512152 |
| HMCN1 | 235944_at | NM_031935.1 | 83872 | Hs.58877 |
| HMOX1 | 203665_at | NM_002133.1 | 3162 | Hs.517581 |
| HOMER2 | 217080_s_at | NM_004839.2 | 9455 | Hs.93564 |
| HOMER3 | 215489_x_at | NM_004838.2 | 9454 | Hs.410683 |
| HOMER3 | 222222_s_at | NM_004838.2 | 9454 | Hs.410683 |
| HOMER3 | 204647_at | NM_004838.2 | 9454 | Hs.410683 |
| HP | 206697_s_at | NM_005143.2 | 3240 | Hs.513711 |
| HP | 208470_s_at | NM_005143.2 | 3240 | Hs.513711 |
| HPN | 204934_s_at | NM_002151.1 | 3249 | Hs.182385 |
| HPR | 208471_at | NM_020995.3 | 3250 | Hs.512155 |
| HPX | 39763_at | NM_000613.1 | 3263 | Hs.426485 |
| HPX | 210013_at | NM_000613.1 | 3263 | Hs.426485 |
| HRASLS3 | 209581_at | NM_007069.1 | 11145 | Hs.502775 |
| HRASLS3 | 235110_at | NM_007069 | 11145 | Hs.417630 |
| HRG | 31835_at | NM_000412.2 | 3273 | Hs.1498 |
| HRG | 206226_at | NM_000412.2 | 3273 | Hs.1498 |
| HRH1 | 216735_x_at | AK024553 | 3269 | 1570 |
| HRH1 | 216738_at | AK024553 | 3269 | 1570 |
| HRH1 | 205579_at | NM_000861.2 | 3269 | Hs.1570 |
| HRH1 | 205580_s_at | NM_000861.2 | 3269 | Hs.1570 |
| HRH2 | 220805_at | NM_022304.1 | 3274 | Hs.247885 |
| HRH3 | 220447_at | NM_007232.1 | 11255 | Hs.251399 |
| HRH3 | 221663_x_at | NM_007232.1 | 11255 | Hs.251399 |
| HRH3 | 1555215_a_at | NM_007232.1 | 11255 | Hs.251399 |
| HRH3 | 1555715_a_at | NM_007232.1 | 11255 | Hs.251399 |
| HRH4 | 221169_s_at | NM_021624.2 | 59340 | Hs.287388 |
| HRH4 | 221170_at | NM_021624.2 | 59340 | Hs.287388 |
| HS3ST3B1 | 221062_at | NM_006041.1 | 9953 | Hs.48384 |
| HS3ST3B1 | 227361_at | NM_006041 | 9953 | Hs.48384 |
| HS3ST3B1 | 1561908_a_at | NM_006041 | 9953 | Hs.48384 |
| HS6ST1 | 225263_at | NM_004807 | 9394 | Hs.512841 |
| HS6ST1 | 206997_s_at | NM_004807.1 | 9394 | Hs.528498 |
| HS6ST2 | 230030_at | NM_147175.2 | 90161 | Hs.385956 |
| HS6ST2 | 1552766_at | NM_147175.2 | 90161 | Hs.385956 |
| HS6ST2 | 1552767_a_at | NM_147175.2 | 90161 | Hs.385956 |
| HS6ST3 | 229714_at | NM_153456.2 | 266722 | Hs.171001 |
| HS6ST3 | 232275_s_at | NM_153456.2 | 266722 | Hs.171001 |
| HS6ST3 | 232276_at | NM_153456.2 | 266722 | Hs.171001 |
| HS6ST3 | 1552842_at | NM_153456.2 | 266722 | Hs.171001 |
| HSD11B1L | 228757_at | NM_198704.1 | 374875 | Hs.512319 |
| HSD11B1L | 1569905_at | NM_198533.1 | 374875 | Hs.512319 |
| HSPA2 | 211538_s_at | NM_021979.2 | 3306 | Hs.432648 |
| HSPA9B | 200690_at | NM_004134 | 3313 | Hs.184233 |
| HSPA9B | 200691_s_at | NM_004134.4 | 3313 | Hs.560739 |
| HSPA9B | 200692_s_at | NM_004134.4 | 3313 | Hs.560739 |
| HSPA9B | 232200_at | NM_004134 | 3313 | Hs.184233 |
| HSPC105 | 229522_at | NM_145168 | 93517 | Hs.87779 |
| HSPD1 | 241716_at | NM_002156 | 3329 | Hs.79037 |
| HSPD1 | 243372_at | NM_002156 | 3329 | Hs.79037 |
| HSPD1 | 200806_s_at | NM_002156.4 | 3329 | Hs.113684 |
| HSPD1 | 200807_s_at | NM_002156.4 | 3329 | Hs.113684 |
| HSPG2 | 201654_s_at | NM_005529.3 | 3339 | Hs.555874 |
| HSPG2 | 201655_s_at | NM_005529.3 | 3339 | Hs.555874 |
| HTN1 | 206639_x_at | NM_002159.2 | 3346 | Hs.250959 |
| HTN3 | 206786_at | NM_000200.1 | 3347 | Hs.177888 |
| HTR1A | 221351_at | NM_000524.2 | 3350 | Hs.247940 |
| HTR1B | 210799_at | NM_000863.1 | 3351 | Hs.123016 |
| HTR1D | 207368_at | NM_000864.3 | 3352 | Hs.121482 |
| HTR1E | 207404_s_at | NM_000865.1 | 3354 | Hs.1611 |
| HTR1F | 221458_at | NM_000866 | 3355 | Hs.248136 |
| HTR2A | 207135_at | NM_000621.2 | 3356 | Hs.424980 |
| HTR2A | 211616_s_at | NM_000621.2 | 3356 | Hs.424980 |
| HTR2B | 206638_at | NM_000867.2 | 3357 | Hs.421649 |
| HTR2C | 207307_at | NM_000868.1 | 3358 | Hs.149037 |
| HTR2C | 211479_s_at | NM_000868.1 | 3358 | Hs.149037 |
| HTR3A | 216615_s_at | NM_000869.2 | 3359 | Hs.413899 |
| HTR3A | 217002_s_at | NM_000869.2 | 3359 | Hs.413899 |
| HTR3B | 221084_at | NM_006028.3 | 9177 | Hs.241377 |
| HTR3C | 1553041_at | NM_130770 | 170572 | Hs.352185 |
| HTR4 | 216939_s_at | NM_001040169.1 | 3360 | Hs.483773 |
| HTR4 | 207577_at | NM_001040172.1 | 3360 | Hs.483773 |
| HTR4 | 207578_s_at | NM_001040173.1 | 3360 | Hs.483773 |
| HTR5A | 221362_at | NM_024012.2 | 3361 | Hs.65791 |
| HTR6 | 1552857_a_at | NM_000871.1 | 3362 | Hs.22180 |
| HTR6 | 206944_at | NM_000871 | 3362 | Hs.22180 |
| HTR7 | 216098_s_at | NM_000872.3 | 3363 | Hs.73739 |
| HTR7 | 236281_x_at | NM_000872.3 | 3363 | Hs.73739 |
| HTR7 | 207818_s_at | NM_000872.3 | 3363 | Hs.73739 |
| HTR7 | 207927_at | NM_019859.2 | 3363 | Hs.73739 |
| HTRA1 | 201185_at | NM_002775.3 | 5654 | Hs.501280 |
| HTRA3 | 226944_at | NM_053044.2 | 94031 | Hs.479119 |
| HTRA3 | 228580_at | NM_053044 | 94031 | Hs.479119 |
| HVCN1 | 226879_at | NM_032369.2 | 84329 | Hs.334637 |
| HYAL2 | 206855_s_at | NM_033158.2 | 8692 | Hs.76873 |
| HYAL4 | 220249_at | NM_012269.1 | 23553 | Hs.28673 |
| IAPP | 207062_at | NM_000415.1 | 3375 | Hs.142255 |
| IBRDC1 | 235492_at | NM_152553 | 154214 | Hs.368639 |
| IBRDC3 | 213038_at | NM_153341.1 | 127544 | Hs.546478 |
| IBRDC3 | 36564_at | NM_153341.1 | 127544 | Hs.546478 |
| IBSP | 236028_at | NM_004967 | 3381 | Hs.49215 |
| IBSP | 207370_at | NM_004967.2 | 3381 | Hs.518726 |
| ICAM1 | 215485_s_at | NM_000201.1 | 3383 | Hs.515126 |
| ICAM1 | 1570090_at | NM_000201 | 3383 | Hs.168383 |
| ICAM1 | 202637_s_at | NM_000201.1 | 3383 | Hs.515126 |
| ICAM1 | 202638_s_at | NM_000201.1 | 3383 | Hs.515126 |
| ICAM2 | 213620_s_at | NM_000873.2 | 3384 | Hs.431460 |
| ICAM2 | 204683_at | NM_000873.2 | 3384 | Hs.431460 |
| ICAM3 | 204949_at | NM_002162.2 | 3385 | Hs.353214 |
| ICAM4 | 207194_s_at | NM_001039132.1 | 3386 | Hs.386467 |
| ICAM5 | 206906_at | NM_003259.2 | 7087 | Hs.465862 |
| ICOS | 210439_at | NM_012092.2 | 29851 | Hs.56247 |
| ICOSLG | 213450_s_at | NM_015259.4 | 23308 | Hs.14155 |
| ICOSLG | 211197_s_at | NM_015259.4 | 23308 | Hs.14155 |
| ICOSLG | 211198_s_at | NM_015259.4 | 23308 | Hs.14155 |
| ICOSLG | 211199_s_at | NM_015259.4 | 23308 | Hs.14155 |
| IER3 | 201631_s_at | NM_003897.2 | 8870 | Hs.76095 |
| IF | 1555564_a_at | NM_000204.1 | 3426 | Hs.312485 |
| IF | 203854_at | NM_000204.1 | 3426 | Hs.312485 |
| IFI27 | 202411_at | NM_005532.3 | 3429 | Hs.532634 |
| IFI6 | 216510_x_at | NM_000584 | 152098 | 543209 |
| IFI6 | 216557_x_at | NM_002038 | 2537 | 511731 |
| IFI6 | 211868_x_at | NM_002038 | 2537 | 511731 |
| IFITM1 | 214022_s_at | NM_003641.2 | 8519 | Hs.458414 |
| IFITM1 | 201601_x_at | NM_003641.2 | 8519 | Hs.458414 |
| IFITM2 | 201315_x_at | NM_006435.1 | 10581 | Hs.174195 |
| IFITM3 | 212203_x_at | NM_021034.1 | 10410 | Hs.374650 |
| IFNA1 | 208344_x_at | NM_024013.1 | 3439 | Hs.37026 |
| IFNA1 | 208375_at | NM_024013.1 | 3439 | Hs.37026 |
| IFNA10 | 208261_x_at | NM_002171.1 | 3446 | Hs.282275 |
| IFNA10 | 211405_x_at | NM_002171.1 | 3446 | Hs.282275 |
| IFNA14 | 208182_x_at | NM_002172.1 | 3448 | Hs.93907 |
| IFNA16 | 208448_x_at | NM_002173.1 | 3449 | Hs.56303 |
| IFNA2 | 211338_at | NM_000605.2 | 3440 | Hs.211575 |
| IFNA21 | 211145_x_at | NM_002175.1 | 3452 | Hs.113211 |
| IFNA4 | 207964_x_at | NM_021068.1 | 3441 | Hs.1510 |
| IFNA5 | 214569_at | NM_002169 | 3442 | Hs.37113 |
| IFNA6 | 208548_at | NM_021002.1 | 3443 | Hs.533470 |
| IFNA7 | 208259_x_at | NM_021057.2 | 3444 | Hs.282274 |
| IFNA8 | 207932_at | NM_002170.2 | 3445 | Hs.73890 |
| IFNAR1 | 225661_at | NM_000629.2 | 3454 | Hs.529400 |
| IFNAR1 | 225669_at | NM_000629.2 | 3454 | Hs.529400 |
| IFNAR1 | 204191_at | NM_000629.2 | 3454 | Hs.529400 |
| IFNAR2 | 227125_at | NM_000874 | 3455 | Hs.86958 |
| IFNAR2 | 204785_x_at | NM_000874.3 | 3455 | Hs.549042 |
| IFNAR2 | 204786_s_at | NM_207585.1 | 3455 | Hs.549042 |
| IFNB1 | 208173_at | NM_002176.2 | 3456 | Hs.93177 |
| IFNE1 | 1553574_at | NM_176891.3 | 338376 | Hs.441972 |
| IFNG | 210354_at | NM_000619.2 | 3458 | Hs.856 |
| IFNGR1 | 202727_s_at | NM_000416.1 | 3459 | Hs.520414 |
| IFNGR1 | 211676_s_at | NM_000416.1 | 3459 | Hs.520414 |
| IFNGR2 | 231696_x_at | NM_005534 | 3460 | Hs.409200 |
| IFNGR2 | 201642_at | NM_005534.2 | 3460 | Hs.517240 |
| IFNK | 224093_at | NM_020124.1 | 56832 | Hs.567291 |
| IFNW1 | 207817_at | NM_002177.1 | 3467 | Hs.73010 |
| IGF1 | 209540_at | NM_000618.2 | 3479 | Hs.160562 |
| IGF1 | 209541_at | NM_000618.2 | 3479 | Hs.160562 |
| IGF1 | 209542_x_at | NM_000618.2 | 3479 | Hs.160562 |
| IGF1 | 211577_s_at | NM_000618.2 | 3479 | Hs.160562 |
| IGF1R | 203627_at | NM_000875 | 3480 | Hs.239176 |
| IGF1R | 203628_at | NM_000875 | 3480 | Hs.239176 |
| IGF1R | 208441_at | NM_000875 | 3480 | Hs.20573 |
| IGF2 | 1564836_at | NM_000612 | 3481 | 373908 |
| IGF2 | 202409_at | BC042127.1 | 3481 | Hs.373908 |
| IGF2 | 202410_x_at | NM_000612 | 3481 | Hs.373908 |
| IGF2 | 210881_s_at | NM_000612 | 3481 | Hs.373908 |
| IGF2R | 201392_s_at | NM_000876.1 | 3482 | Hs.487062 |
| IGF2R | 201393_s_at | NM_000876.1 | 3482 | Hs.487062 |
| IGFALS | 215712_s_at | NM_004970.1 | 3483 | Hs.839 |
| IGFBP1 | 237989_at | NM_000596 | 3484 | Hs.401316 |
| IGFBP1 | 205302_at | NM_000596.2 | 3484 | Hs.401316 |
| IGFBP2 | 202718_at | NM_000597.2 | 3485 | Hs.438102 |
| IGFBP3 | 210095_s_at | NM_000598.4 | 3486 | Hs.450230 |
| IGFBP3 | 212143_s_at | NM_000598.4 | 3486 | Hs.450230 |
| IGFBP4 | 201508_at | NM_001552.2 | 3487 | Hs.462998 |
| IGFBP5 | 1555997_s_at | NM_000599.2 | 3488 | Hs.148567 |
| IGFBP5 | 203424_s_at | NM_000599.2 | 3488 | Hs.148567 |
| IGFBP5 | 203425_s_at | NM_000599.2 | 3488 | Hs.148567 |
| IGFBP5 | 203426_s_at | NM_000599.2 | 3488 | Hs.148567 |
| IGFBP5 | 211958_at | NM_000599.2 | 3488 | Hs.148567 |
| IGFBP5 | 211959_at | NM_000599.2 | 3488 | Hs.148567 |
| IGFBP6 | 203851_at | NM_002178.2 | 3489 | Hs.274313 |
| IGFBP7 | 213910_at | NM_001553 | 3490 | Hs.435795 |
| IGFBP7 | 236764_at | NM_001553 | 3490 | Hs.435795 |
| IGFBP7 | 201162_at | NM_001553.1 | 3490 | Hs.479808 |
| IGFBP7 | 201163_s_at | NM_001553.1 | 3490 | Hs.479808 |
| IGFL1 | 239430_at | NM_198541.1 | 374918 | Hs.546554 |
| IGFL2 | 231148_at | NM_001002915.1 | 147920 | Hs.99376 |
| IGHA1 | 214973_x_at | AK128565.1 | 3493 | Hs.497723 |
| IGHA1 | 216541_x_at | AJ275397 | 28461 | 648398 |
| IGHA1 | 216542_x_at | AJ275355 | 3493 | 648398 |
| IGHA1 | 216706_x_at | M21388 | 152098 | 543209 |
| IGHA1 | 217022_s_at | XM_942723.1 | 3493 | Hs.497723 |
| IGHA1 | 217169_at | M31949 | 3493 | 648398 |
| IGHA1 | 217360_x_at | AJ275408 | 3493 | 648398 |
| IGHA1 | 234477_at | AJ243643 | 3493 | 648398 |
| IGHA1 | 234792_x_at | AJ275439 | 3493 | 648398 |
| IGHD | 213674_x_at | AK090461.1 | 3495 | Hs.558340 |
| IGHD | 215621_s_at | AK090461.1 | 3495 | Hs.558340 |
| IGHD | 222285_at | AK090461.1 | 3495 | Hs.558340 |
| IGHD | 230877_at | AK126280.1 |  |  |
| IGHG1 | 217369_at | AJ275383 | 3500 | 510635 |
| IGHG1 | 228518_at | AK128301.1 | 3500 | Hs.558341 |
| IGHG1 | 211430_s_at |  | 649897 | Hs.510635 |
| IGHG4 | 231668_x_at | BG222989 | 3503 | Hs.497712 |
| IGHM | 209374_s_at | BC001872 | 3507 | Hs.510635 |
| IGHM | 212827_at | X17115 | 3507 | Hs.510635 |
| IGJ | 212592_at | NM_144646 | 3512 | Hs.381568 |
| IGKC | 211645_x_at | BC095489.1 | 3514 | Hs.449621 |
| IGKV1-5 | 214768_x_at | BC034142.1 | 28299 | Hs.449621 |
| IGLL1 | 206660_at | NM_020070.2 | 3543 | Hs.348935 |
| IGSF1 | 223807_at | NM_001555 | 3547 | Hs.22111 |
| IGSF1 | 207695_s_at | NM_001555.2 | 3547 | Hs.22111 |
| IGSF10 | 230670_at | NM_178822.3 | 285313 | Hs.477965 |
| IGSF11 | 228375_at | NM_001015887.1 | 152404 | Hs.112873 |
| IGSF2 | 244652_at | NM_004258 | 9398 | Hs.74115 |
| IGSF2 | 207167_at | NM_004258.1 | 9398 | Hs.74115 |
| IGSF21 | 227154_at | NM_032880.2 | 84966 | Hs.212511 |
| IGSF3 | 1552672_a_at | NM_001007237.1 | 3321 | Hs.171057 |
| IGSF3 | 1556162_at | NM_001542 | 3321 | Hs.171057 |
| IGSF3 | 1556163_a_at | NM_001542.2 | 3321 | Hs.171057 |
| IGSF3 | 202421_at | NM_001007237.1 | 3321 | Hs.171057 |
| IGSF4 | 209030_s_at | NM_014333.2 | 23705 | Hs.370510 |
| IGSF4 | 209031_at | NM_014333 | 23705 | Hs.156682 |
| IGSF4 | 209032_s_at | NM_014333.2 | 23705 | Hs.370510 |
| IGSF4 | 244345_at | NM_014333 | 23705 | Hs.370510 |
| IGSF4B | 211677_x_at | NM_021189.2 | 57863 | Hs.365689 |
| IGSF4B | 213948_x_at | NM_021189.2 | 57863 | Hs.365689 |
| IGSF4B | 221921_s_at | NM_021189.2 | 57863 | Hs.365689 |
| IGSF4C | 215258_at | NM_145296.1 | 199731 | Hs.370984 |
| IGSF4C | 229335_at | NM_145296.1 | 199731 | Hs.370984 |
| IGSF4D | 1552752_a_at | NM_153184.2 | 253559 | Hs.333991 |
| IGSF4D | 1552754_a_at | NM_153184.2 | 253559 | Hs.333991 |
| IGSF4D | 1555544_a_at | NM_153184.2 | 253559 | Hs.333991 |
| IGSF6 | 206420_at | NM_005849.2 | 10261 | Hs.530902 |
| IGSF8 | 225025_at | NM_052868.1 | 93185 | Hs.332012 |
| IGSF9 | 229276_at | NM_020789.2 | 57549 | Hs.492155 |
| IHH | 215420_at | BE869172 | 3549 | Hs.369782 |
| IHH | 229358_at | AA628967 | 3549 | Hs.369782 |
| IL10 | 207433_at | NM_000572.2 | 3586 | Hs.193717 |
| IL10RA | 204912_at | NM_001558.2 | 3587 | Hs.504035 |
| IL10RB | 209575_at | NM_000628.3 | 3588 | Hs.418291 |
| IL11 | 206924_at | NM_000641.2 | 3589 | Hs.467304 |
| IL11 | 206926_s_at | NM_000641.2 | 3589 | Hs.467304 |
| IL11RA | 1552646_at | NM_147162.1 | 3590 | Hs.558346 |
| IL11RA | 204773_at | NM_004512.3 | 3590 | Hs.558346 |
| IL12A | 207160_at | NM_000882.2 | 3592 | Hs.673 |
| IL12B | 207901_at | NM_002187.2 | 3593 | Hs.674 |
| IL12RB1 | 239522_at | NM_005535 | 3594 | Hs.223894 |
| IL12RB1 | 1552584_at | NM_153701.1 | 3594 | Hs.223894 |
| IL12RB1 | 206890_at | NM_005535.1 | 3594 | Hs.223894 |
| IL12RB2 | 206999_at | NM_001559.2 | 3595 | Hs.479347 |
| IL13 | 207844_at | NM_002188.2 | 3596 | Hs.845 |
| IL13RA1 | 201887_at | NM_001560.2 | 3597 | Hs.496646 |
| IL13RA1 | 201888_s_at | NM_001560.2 | 3597 | Hs.496646 |
| IL13RA1 | 210904_s_at | NM_001560.2 | 3597 | Hs.496646 |
| IL13RA1 | 211612_s_at | NM_001560.2 | 3597 | Hs.496646 |
| IL13RA2 | 206172_at | NM_000640.2 | 3598 | Hs.336046 |
| IL15 | 217371_s_at | NM_000585.2 | 3600 | Hs.311958 |
| IL15 | 205992_s_at | NM_000585.2 | 3600 | Hs.311958 |
| IL15RA | 207375_s_at | NM_002189.2 | 3601 | Hs.524117 |
| IL16 | 1555016_at | NM_004513 | 3603 | Hs.459095 |
| IL16 | 209827_s_at | NM_004513.3 | 3603 | Hs.459095 |
| IL16 | 209828_s_at | NM_172217.1 | 3603 | Hs.459095 |
| IL17 | 208402_at | NM_002190.2 | 3605 | Hs.41724 |
| IL17 | 216876_s_at | NM_002190.2 | 3605 | Hs.41724 |
| IL17B | 220273_at | NM_014443.2 | 27190 | Hs.409558 |
| IL17C | 224079_at | NM_013278.3 | 27189 | Hs.278911 |
| IL17D | 227401_at | NM_138284.1 | 53342 | Hs.130652 |
| IL17D | 228977_at | NM_138284 | 53342 | Hs.434103 |
| IL17E | 220971_at | NM_172314.1 | 64806 | Hs.302036 |
| IL17F | 234408_at | NM_052872.3 | 112744 | Hs.272295 |
| IL17R | 205707_at | NM_014339.4 | 23765 | Hs.129751 |
| IL17RB | 219255_x_at | NM_018725.2 | 55540 | Hs.558512 |
| IL17RB | 224156_x_at | NM_018725.2 | 55540 | Hs.558512 |
| IL17RB | 224361_s_at | NM_172234.1 | 55540 | Hs.558512 |
| IL17RB | 235531_at | NM_018725 | 55540 | Hs.5470 |
| IL17RB | 236897_at | NM_018725 | 55540 | Hs.5470 |
| IL17RC | 221926_s_at | NM_032732.3 | 84818 | Hs.129959 |
| IL17RC | 224514_x_at | NM_032732.3 | 84818 | Hs.129959 |
| IL17RC | 64440_at | NM_032732.3 | 84818 | Hs.129959 |
| IL17RD | 229263_at | NM_017563.1 | 54756 | Hs.558503 |
| IL17RE | 229401_at | NM_144640 | 132014 | Hs.390823 |
| IL17RE | 236186_x_at | NM_144640.2 | 132014 | Hs.390823 |
| IL18 | 206295_at | NM_001562.2 | 3606 | Hs.83077 |
| IL18BP | 219323_s_at | NM_005699 | 10068 | Hs.325978 |
| IL18BP | 222868_s_at | NM_001039660.1 | 10068 | Hs.325978 |
| IL18BP | 224283_x_at | NM_005699 | 10068 | Hs.325978 |
| IL18R1 | 206618_at | NM_003855.2 | 8809 | Hs.469521 |
| IL18RAP | 207072_at | NM_003853.2 | 8807 | Hs.158315 |
| IL19 | 220745_at | NM_153758.1 | 29949 | Hs.128395 |
| IL1A | 208200_at | NM_000575 | 3552 | Hs.1722 |
| IL1A | 210118_s_at | NM_000575.3 | 3552 | Hs.1722 |
| IL1B | 39402_at | NM_000576.2 | 3553 | Hs.126256 |
| IL1B | 205067_at | NM_000576.2 | 3553 | Hs.126256 |
| IL1F10 | 224262_at | NM_173161.1 | 84639 | Hs.306974 |
| IL1F5 | 222223_s_at | NM_012275.2 | 26525 | Hs.516301 |
| IL1F6 | 221404_at | NM_014440.1 | 27179 | Hs.278910 |
| IL1F7 | 221470_s_at | NM_014439.3 | 27178 | Hs.166371 |
| IL1F7 | 224555_x_at | NM_173202.1 | 27178 | Hs.166371 |
| IL1F8 | 224230_at | NM_014438.3 | 27177 | Hs.278909 |
| IL1F8 | 231755_at | NM_173178.1 | 27177 | Hs.278909 |
| IL1F9 | 220322_at | NM_019618.2 | 56300 | Hs.211238 |
| IL1R1 | 215561_s_at | NM_000877.2 | 3554 | Hs.557403 |
| IL1R1 | 202948_at | NM_000877.2 | 3554 | Hs.557403 |
| IL1R2 | 205403_at | NM_004633.3 | 7850 | Hs.25333 |
| IL1R2 | 211372_s_at | NM_004633.3 | 7850 | Hs.25333 |
| IL1RAP | 205227_at | NM_002182.2 | 3556 | Hs.478673 |
| IL1RAP | 210233_at | NM_134470.2 | 3556 | Hs.478673 |
| IL1RAPL1 | 220663_at | NM_014271.2 | 11141 | Hs.495893 |
| IL1RAPL1 | 222963_s_at | NM_014271.2 | 11141 | Hs.495893 |
| IL1RAPL1 | 234417_at | AF181286 | 11141 | 495893 |
| IL1RAPL1 | 234746_at | AF181286 | 11141 | 495893 |
| IL1RAPL2 | 221112_at | NM_017416.1 | 26280 | Hs.567266 |
| IL1RL1 | 234066_at | NM_003856 | 9173 | Hs.66 |
| IL1RL1 | 242809_at | NM_003856 | 9173 | Hs.66 |
| IL1RL1 | 207526_s_at | NM_003856.2 | 9173 | Hs.66 |
| IL1RL1 | 210442_at | NM_016232.4 | 9173 | Hs.66 |
| IL1RL2 | 208038_at | NM_003854.2 | 8808 | Hs.469520 |
| IL1RN | 216243_s_at | NM_173843.1 | 3557 | Hs.81134 |
| IL1RN | 216244_at | NM_173843.1 | 3557 | Hs.81134 |
| IL1RN | 212657_s_at | NM_173843.1 | 3557 | Hs.81134 |
| IL1RN | 212659_s_at | NM_000577.3 | 3557 | Hs.81134 |
| IL2 | 207849_at | NM_000586.2 | 3558 | Hs.89679 |
| IL20 | 224071_at | NM_018724.3 | 50604 | Hs.272373 |
| IL20RA | 219115_s_at | NM_014432.2 | 53832 | Hs.445868 |
| IL20RA | 222828_at | NM_014432 | 53832 | Hs.288240 |
| IL20RA | 222829_s_at | NM_014432.2 | 53832 | Hs.445868 |
| IL20RA | 240396_at | NM_014432 | 53832 | Hs.288240 |
| IL21 | 221271_at | NM_021803.1 | 59067 | Hs.439487 |
| IL21R | 219971_at | NM_021798.2 | 50615 | Hs.210546 |
| IL21R | 221658_s_at | NM_181078.1 | 50615 | Hs.210546 |
| IL22 | 221165_s_at | NM_020525.4 | 50616 | Hs.287369 |
| IL22 | 222974_at | NM_020525.4 | 50616 | Hs.287369 |
| IL22RA1 | 220056_at | NM_021258.2 | 58985 | Hs.110915 |
| IL22RA2 | 237493_at | NM_181310.1 | 116379 | Hs.126891 |
| IL23A | 220054_at | NM_016584.2 | 51561 | Hs.98309 |
| IL23R | 1552912_a_at | NM_144701.2 | 149233 | Hs.200929 |
| IL23R | 1561853_a_at | NM_144701.2 | 149233 | Hs.200929 |
| IL24 | 239122_at | NM_006850 | 11009 | Hs.411311 |
| IL24 | 206569_at | NM_006850.2 | 11009 | Hs.411311 |
| IL26 | 221111_at | NM_018402.1 | 55801 | Hs.272350 |
| IL27 | 1552995_at | NM_145659.3 | 246778 | Hs.528111 |
| IL27RA | 217702_at | NM_004843.2 | 9466 | Hs.132781 |
| IL27RA | 222062_at | NM_004843.2 | 9466 | Hs.132781 |
| IL27RA | 205926_at | NM_004843.2 | 9466 | Hs.132781 |
| IL28A | 1552915_at | NM_172138.1 | 282616 | Hs.406744 |
| IL28B | 1552609_s_at | NM_172139.2 | 282617 | Hs.406744 |
| IL28RA | 244261_at | NM_170743.2 | 163702 | Hs.221375 |
| IL28RA | 1555499_a_at | NM_170743.2 | 163702 | Hs.221375 |
| IL29 | 1552917_at | NM_172140.1 | 282618 | Hs.406745 |
| IL2RA | 206341_at | NM_000417.1 | 3559 | Hs.231367 |
| IL2RA | 211269_s_at | NM_000417.1 | 3559 | Hs.231367 |
| IL2RB | 205291_at | NM_000878.2 | 3560 | Hs.474787 |
| IL2RG | 204116_at | NM_000206.1 | 3561 | Hs.84 |
| IL3 | 207906_at | NM_000588.3 | 3562 | Hs.694 |
| IL31RA | 243541_at | AY499339.1 | 133396 | Hs.55378 |
| IL31RA | 1553032_at | NM_139017.3 | 133396 | Hs.55378 |
| IL31RA | 1555431_a_at | NM_139017.3 | 133396 | Hs.55378 |
| IL32 | 203828_s_at | NM_001012635.1 | 9235 | Hs.943 |
| IL3RA | 206148_at | NM_002183.2 | 3563 | Hs.196472 |
| IL4 | 207538_at | NM_000589.2 | 3565 | Hs.73917 |
| IL4 | 207539_s_at | NM_172348.1 | 3565 | Hs.73917 |
| IL4R | 203233_at | NM_000418.2 | 3566 | Hs.513457 |
| IL5 | 207952_at | NM_000879.2 | 3567 | Hs.2247 |
| IL5RA | 207902_at | NM_000564.2 | 3568 | Hs.68876 |
| IL5RA | 210744_s_at | NM_000564.2 | 3568 | Hs.68876 |
| IL5RA | 211516_at | NM_175724.1 | 3568 | Hs.68876 |
| IL5RA | 211517_s_at | NM_000564.2 | 3568 | Hs.68876 |
| IL6 | 243977_at | BC027978.1 | 541472 | Hs.512234 |
| IL6 | 205207_at | NM_000600.1 | 3569 | Hs.512234 |
| IL6R | 217489_s_at | NM_000565.2 | 3570 | Hs.135087 |
| IL6R | 226333_at | NM_000565 | 3570 | Hs.193400 |
| IL6R | 205945_at | NM_000565.2 | 3570 | Hs.135087 |
| IL6ST | 234474_x_at | NM_002184 | 3572 | Hs.71968 |
| IL6ST | 234967_at | NM_002184 | 3572 | Hs.454699 |
| IL6ST | 204863_s_at | NM_002184.2 | 3572 | Hs.454699 |
| IL6ST | 204864_s_at | NM_002184.2 | 3572 | Hs.454699 |
| IL6ST | 211000_s_at | NM_002184.2 | 3572 | Hs.454699 |
| IL6ST | 212195_at | NM_002184 | 3572 | Hs.71968 |
| IL6ST | 212196_at | NM_002184 | 3572 | Hs.71968 |
| IL7 | 241808_at | NM_000880 | 3574 | Hs.72927 |
| IL7 | 206693_at | NM_000880.2 | 3574 | Hs.536926 |
| IL7R | 226218_at | NM_002185 | 3575 | Hs.362807 |
| IL7R | 205798_at | NM_002185.2 | 3575 | Hs.362807 |
| IL8 | 217384_x_at | AJ275374 | 3576 | 551925 /// 648510 /// 650507 |
| IL8 | 202859_x_at | NM_000584.2 | 3576 | Hs.624 |
| IL8 | 211506_s_at | NM_000584.2 | 3576 | Hs.624 |
| IL8RA | 207094_at | NM_000634.2 | 3577 | Hs.194778 |
| IL8RB | 207008_at | NM_001557.2 | 3579 | Hs.846 |
| IL9 | 208193_at | NM_000590.1 | 3578 | Hs.960 |
| IL9R | 217212_s_at | NM_002186.2 | 3581 | Hs.406228 |
| IL9R | 208164_s_at | NM_002186.2 | 3581 | Hs.406228 |
| ILDR1 | 235583_at | NM_175924.2 | 286676 | Hs.98484 |
| ILDR1 | 1553545_at | NM_175924.2 | 286676 | Hs.98484 |
| ILVBL | 202993_at | NM_006844.3 | 10994 | Hs.78880 |
| ILVBL | 210624_s_at | NM_176826.1 | 10994 | Hs.78880 |
| IMAGE:4837709 | 1558493_at | BC053680.1 | 164380 | Hs.400007 |
| IMPG1 | 207054_at | NM_001563.2 | 3617 | Hs.129882 |
| IMPG2 | 220830_at | NM_016247.2 | 50939 | Hs.272380 |
| INHBA | 204926_at | NM_002192 | 3624 | Hs.28792 |
| INHBA | 210511_s_at | NM_002192.2 | 3624 | Hs.28792 |
| INHBB | 205258_at | NM_002193.1 | 3625 | Hs.1735 |
| INHBC | 207687_at | NM_005538.2 | 3626 | Hs.374664 |
| INHBC | 207688_s_at | NM_005538 | 3626 | Hs.374664 |
| INHBE | 210587_at | NM_031479.3 | 83729 | Hs.279497 |
| INS | 206598_at | NM_000207 | 3630 | Hs.89832 |
| INSL3 | 214400_at | NM_005543.2 | 3640 | Hs.37062 |
| INSL3 | 214572_s_at | NM_005543.2 | 3640 | Hs.37062 |
| INSL3 | 1553594_a_at | NM_005543.2 | 3640 | Hs.37062 |
| INSL4 | 206549_at | NM_002195.1 | 3641 | Hs.418506 |
| INSL5 | 221091_at | NM_005478.3 | 10022 | Hs.251380 |
| INSL6 | 221403_s_at | NM_007179.2 | 11172 | Hs.216373 |
| INSR | 213792_s_at | NM_000208 | 3643 | Hs.465744 |
| INSR | 226212_s_at | NM_000208 | 3643 | Hs.438669 |
| INSR | 226216_at | NM_000208 | 3643 | Hs.438669 |
| INSR | 226450_at | NM_000208 | 3643 | Hs.438669 |
| INSR | 227432_s_at | NM_000208 | 3643 | Hs.438669 |
| INSR | 207851_s_at | NM_000208.1 | 3643 | Hs.465744 |
| INSRR | 215776_at | NM_014215.1 | 3645 | Hs.248138 |
| ISLR | 207191_s_at | NM_005545.3 | 3671 | Hs.513022 |
| ISLR2 | 232208_at | NM_020851.1 | 57611 | Hs.408161 |
| ITGA1 | 214660_at | NM_181501 | 3672 | Hs.439320 |
| ITGA1 | 226731_at | NM_181501 | 3672 | Hs.439320 |
| ITGA1 | 1560359_at | NM_181501 | 3672 | Hs.439320 |
| ITGA10 | 206766_at | NM_003637.3 | 8515 | Hs.158237 |
| ITGA11 | 222899_at | NM_001004439.1 | 22801 | Hs.436416 |
| ITGA11 | 1554819_a_at | NM_001004439.1 | 22801 | Hs.436416 |
| ITGA2 | 227314_at | NM_002203 | 3673 | Hs.387725 |
| ITGA2 | 237728_at | NM_002203 | 3673 | Hs.387725 |
| ITGA2 | 205032_at | NM_002203.2 | 3673 | Hs.482077 |
| ITGA2B | 216956_s_at | NM_000419.3 | 3674 | Hs.411312 |
| ITGA2B | 216966_at | J02963 | 3674 | 411312 |
| ITGA2B | 206493_at | NM_000419.3 | 3674 | Hs.411312 |
| ITGA2B | 206494_s_at | NM_000419.3 | 3674 | Hs.411312 |
| ITGA3 | 201474_s_at | NM_005501.1 | 3675 | Hs.265829 |
| ITGA4 | 213416_at | NM_000885.4 | 3676 | Hs.555880 |
| ITGA4 | 243366_s_at | NM_000885 | 3676 | Hs.145140 |
| ITGA4 | 244599_at | NM_000885 | 3676 | Hs.145140 |
| ITGA4 | 205884_at | NM_000885.4 | 3676 | Hs.555880 |
| ITGA4 | 205885_s_at | NM_000885.4 | 3676 | Hs.555880 |
| ITGA5 | 201389_at | NM_002205.2 | 3678 | Hs.505654 |
| ITGA6 | 215177_s_at | NM_000210.1 | 3655 | Hs.133397 |
| ITGA6 | 229643_at | NM_000210 | 3655 | Hs.212296 |
| ITGA6 | 201656_at | NM_000210.1 | 3655 | Hs.133397 |
| ITGA7 | 216331_at | NM_002206.1 | 3679 | Hs.524484 |
| ITGA7 | 209663_s_at | NM_002206.1 | 3679 | Hs.524484 |
| ITGA8 | 214265_at | NM_003638.1 | 8516 | Hs.171311 |
| ITGA8 | 239092_at | BF939224 | 8516 | Hs.171025 |
| ITGA8 | 242071_x_at | BF446919 | 8516 | Hs.171025 |
| ITGA9 | 227297_at | BX647350.1 | 3680 | Hs.113157 |
| ITGA9 | 1555335_at | NM_002207 | 3680 | Hs.113157 |
| ITGA9 | 1555336_a_at | NM_002207.2 | 3680 | Hs.113157 |
| ITGA9 | 206009_at | NM_002207.2 | 3680 | Hs.113157 |
| ITGAD | 1560686_at | NM_005353.2 | 3681 | Hs.513528 |
| ITGAE | 205055_at | NM_002208.3 | 3682 | Hs.513867 |
| ITGAL | 213475_s_at | NM_002209.1 | 3683 | Hs.174103 |
| ITGAL | 1554240_a_at | NM_002209.1 | 3683 | Hs.174103 |
| ITGAM | 205786_s_at | NM_000632.3 | 3684 | Hs.172631 |
| ITGAV | 202351_at | NM_002210.2 | 3685 | Hs.436873 |
| ITGAX | 1563003_at | NM_000887 | 3687 | Hs.248472 |
| ITGAX | 210184_at | NM_000887.3 | 3687 | Hs.248472 |
| ITGB1 | 215878_at | NM_002211 | 3688 | Hs.287797 |
| ITGB1 | 216178_x_at | NM_033668.1 | 3688 | Hs.429052 |
| ITGB1 | 216190_x_at | NM_002211 | 3688 | Hs.287797 |
| ITGB1 | 1553530_a_at | NM_033668.1 | 3688 | Hs.429052 |
| ITGB1 | 1553678_a_at | NM_002211.2 | 3688 | Hs.429052 |
| ITGB1 | 1561042_at | NM_002211 | 3688 | Hs.429052 |
| ITGB1 | 211945_s_at | NM_002211.2 | 3688 | Hs.429052 |
| ITGB2 | 236988_x_at | NM_000211 | 3689 | Hs.375957 |
| ITGB2 | 1555349_a_at | NM_000211.2 | 3689 | Hs.375957 |
| ITGB2 | 202803_s_at | NM_000211.2 | 3689 | Hs.375957 |
| ITGB3 | 215240_at | NM_000212 | 3690 | Hs.87149 |
| ITGB3 | 216261_at | NM_000212 | 3690 | Hs.218040 |
| ITGB3 | 204625_s_at | NM_000212.2 | 3690 | Hs.218040 |
| ITGB3 | 204626_s_at | NM_000212.2 | 3690 | Hs.218040 |
| ITGB3 | 204627_s_at | NM_000212.2 | 3690 | Hs.218040 |
| ITGB3 | 204628_s_at | NM_000212.2 | 3690 | Hs.218040 |
| ITGB3 | 211579_at | NM_000212 | 3690 | Hs.218040 |
| ITGB4 | 214292_at | NM_001005619.1 | 3691 | Hs.370255 |
| ITGB4 | 230704_s_at | NM_000213 | 3691 | Hs.85266 |
| ITGB4 | 204989_s_at | NM_000213.3 | 3691 | Hs.370255 |
| ITGB4 | 204990_s_at | NM_000213.3 | 3691 | Hs.370255 |
| ITGB4 | 211905_s_at | NM_000213.3 | 3691 | Hs.370255 |
| ITGB5 | 214020_x_at | NM_002213 | 3693 | Hs.149846 |
| ITGB5 | 201124_at | NM_002213 | 3693 | Hs.149846 |
| ITGB5 | 201125_s_at | NM_002213.3 | 3693 | Hs.13155 |
| ITGB6 | 226535_at | NM_000888 | 3694 | Hs.470399 |
| ITGB6 | 208083_s_at | NM_000888.3 | 3694 | Hs.470399 |
| ITGB6 | 208084_at | NM_000888.3 | 3694 | Hs.470399 |
| ITGB7 | 236810_at | NM_000889 | 3695 | Hs.1741 |
| ITGB7 | 205718_at | NM_000889.1 | 3695 | Hs.1741 |
| ITGB8 | 226189_at | NM_002214.2 | 3696 | Hs.547130 |
| ITGB8 | 242982_x_at | NM_002214 | 3696 | Hs.547130 |
| ITGB8 | 205816_at | NM_002214.2 | 3696 | Hs.547130 |
| ITGB8 | 211488_s_at | NM_002214.2 | 3696 | Hs.547130 |
| ITGBL1 | 214927_at | NM_004791 | 9358 | Hs.311054 |
| ITGBL1 | 1557079_at | NM_004791 | 9358 | Hs.311054 |
| ITGBL1 | 1557080_s_at | NM_004791 | 9358 | Hs.311054 |
| ITGBL1 | 205422_s_at | NM_004791.1 | 9358 | Hs.508597 |
| ITIH1 | 210888_s_at | NM_002215.1 | 3697 | Hs.420257 |
| ITIH2 | 204987_at | NM_002216.2 | 3698 | Hs.75285 |
| ITIH3 | 205755_at | NM_002217.2 | 3699 | Hs.76716 |
| ITIH4 | 242720_at | NM_002218 | 3700 | Hs.76415 |
| ITIH4 | 37201_at | NM_002218.3 | 3700 | Hs.518000 |
| ITIH4 | 206287_s_at | NM_002218.3 | 3700 | Hs.518000 |
| ITIH5 | 219064_at | NM_030569.3 | 80760 | Hs.498586 |
| ITIH5 | 1553243_at | NM_032817.2 | 80760 | Hs.498586 |
| ITIH5L | 234894_at | NM_198510.1 | 347365 | Hs.454272 |
| ITLN1 | 223597_at | NM_017625.2 | 55600 | Hs.50813 |
| ITM2A | 202746_at | NM_004867.3 | 9452 | Hs.17109 |
| ITM2A | 202747_s_at | NM_004867.3 | 9452 | Hs.17109 |
| ITM2B | 217731_s_at | NM_021999.2 | 9445 | Hs.446450 |
| ITM2B | 217732_s_at | NM_021999.2 | 9445 | Hs.446450 |
| ITPR1 | 216944_s_at | NM_002222.1 | 3708 | Hs.443239 |
| ITPR1 | 240052_at | NM_002222 | 3708 | Hs.149900 |
| ITPR1 | 1562373_at | NM_002222 | 3708 | Hs.149900 |
| ITPR1 | 203710_at | NM_002222.1 | 3708 | Hs.443239 |
| ITPR1 | 211323_s_at | NM_002222.1 | 3708 | Hs.443239 |
| ITPR2 | 216487_at | AL049988 | 3709 | 512235 |
| ITPR2 | 216614_at | AL049988 | 3709 | 512235 |
| ITPR2 | 237876_at | AI732946 |  | Hs.150401 |
| ITPR2 | 240458_at | AI242023 |  | Hs.137003 |
| ITPR2 | 243342_at | AI611635 |  | Hs.370354 |
| ITPR2 | 244456_at | AA700218 |  | Hs.189047 |
| ITPR2 | 244457_at | BF724206 |  | Hs.221024 |
| ITPR2 | 202662_s_at | NM_002223 | 3709 | Hs.512235 |
| ITPR2 | 211360_s_at | NM_002223 | 3709 | Hs.512235 |
| ITPR3 | 228192_at | NM_002224 | 3710 | Hs.77515 |
| ITPR3 | 229443_at | NM_002224 | 3710 | Hs.77515 |
| ITPR3 | 239542_at | NM_002224 | 3710 | Hs.77515 |
| ITPR3 | 201187_s_at | NM_002224.1 | 3710 | Hs.65758 |
| ITPR3 | 201188_s_at | NM_002224.1 | 3710 | Hs.65758 |
| ITPR3 | 201189_s_at | NM_002224.1 | 3710 | Hs.65758 |
| IZUMO1 | 1554196_at | NM_182575.1 | 284359 | Hs.400688 |
| JAG1 | 216268_s_at | NM_000214.1 | 182 | Hs.224012 |
| JAG1 | 229924_s_at | NM_000214 | 182 | Hs.409202 |
| JAG1 | 231183_s_at | NM_000214 | 182 | Hs.409202 |
| JAG1 | 209097_s_at | NM_000214.1 | 182 | Hs.224012 |
| JAG1 | 209098_s_at | NM_000214.1 | 182 | Hs.224012 |
| JAG1 | 209099_x_at | NM_000214.1 | 182 | Hs.224012 |
| JAG2 | 32137_at | NM_002226.3 | 3714 | Hs.433445 |
| JAG2 | 209784_s_at | NM_002226.3 | 3714 | Hs.433445 |
| JAM2 | 219213_at | NM_021219.2 | 58494 | Hs.517227 |
| JAM2 | 229127_at | NM_021219 | 58494 | Hs.436494 |
| JAM3 | 231720_s_at | NM_032801.3 | 83700 | Hs.150718 |
| JAM3 | 231721_at | NM_032801.3 | 83700 | Hs.150718 |
| JAM3 | 212813_at | NM_032801.3 | 83700 | Hs.150718 |
| JPH1 | 229139_at | NM_020647.2 | 56704 | Hs.160574 |
| JPH1 | 1553533_at | NM_020647.2 | 56704 | Hs.160574 |
| JPH2 | 220385_at | NM_175913.3 | 57158 | Hs.441737 |
| JPH2 | 229578_at | NM_020433.4 | 57158 | Hs.441737 |
| JPH2 | 236647_at | BF437470 |  | Hs.255277 |
| JPH2 | 1553174_at | NM_020433.4 | 57158 | Hs.441737 |
| JPH3 | 220188_at | NM_020655.2 | 57338 | Hs.123450 |
| JPH3 | 229294_at | NM_020655.2 | 57338 | Hs.123450 |
| JPH3 | 234736_at | NM_020655 | 57338 | Hs.4775 |
| JPH3 | 243679_at | NM_020655 | 57338 | Hs.123450 |
| JTB | 244049_at | NM_006694 | 10899 | Hs.6396 |
| JTB | 200048_s_at | NM_006694.2 | 10899 | Hs.6396 |
| JTB | 210434_x_at | NM_006694.2 | 10899 | Hs.6396 |
| JTB | 210927_x_at | NM_006694 | 10899 | Hs.6396 |
| KAL1 | 229853_at | NM_000216 | 3730 | Hs.380850 |
| KAL1 | 205206_at | NM_000216.1 | 3730 | Hs.559559 |
| KAZALD1 | 217511_at | NM_030929.3 | 81621 | Hs.534859 |
| KAZALD1 | 221000_s_at | AF333487.1 | 81621 | Hs.534859 |
| KCNA1 | 208479_at | NM_000217.1 | 3736 | Hs.416139 |
| KCNA10 | 208560_at | NM_005549.2 | 3744 | Hs.248140 |
| KCNA2 | 239118_at | NM_004974 | 3737 | Hs.248139 |
| KCNA2 | 208564_at | NM_004974.2 | 3737 | Hs.248139 |
| KCNA3 | 207237_at | NM_002232.3 | 3738 | Hs.169948 |
| KCNA4 | 207248_at | NM_002233.2 | 3739 | Hs.458267 |
| KCNA5 | 206762_at | NM_002234.2 | 3741 | Hs.150208 |
| KCNA6 | 1553347_s_at | NM_002235.2 | 3742 | Hs.306190 |
| KCNA7 | 232936_at | NM_031886.2 | 3743 | Hs.306973 |
| KCNAB1 | 230605_at | NM_003471 | 7881 | Hs.157818 |
| KCNAB1 | 231524_at | NM_003471 | 7881 | Hs.157818 |
| KCNAB1 | 208213_s_at | NM_003471.2 | 7881 | Hs.157818 |
| KCNAB1 | 210078_s_at | NM_003471.2 | 7881 | Hs.157818 |
| KCNAB1 | 210079_x_at | NM_172160.1 | 7881 | Hs.157818 |
| KCNAB1 | 210471_s_at | NM_003471.2 | 7881 | Hs.157818 |
| KCNAB2 | 203402_at | NM_003636 | 8514 | Hs.440497 |
| KCNAB2 | 211791_s_at | NM_003636.2 | 8514 | Hs.440497 |
| KCNAB3 | 221413_at | NM_004732.2 | 9196 | Hs.435074 |
| KCNB1 | 211006_s_at | NM_004975.2 | 3745 | Hs.84244 |
| KCNB2 | 208123_at | NM_004770.2 | 9312 | Hs.6702 |
| KCNB2 | 208172_s_at | NM_004770.2 | 9312 | Hs.6702 |
| KCNC1 | 208477_at | NM_004976.2 | 3746 | Hs.303870 |
| KCNC2 | 240614_at | NM_153748.1 | 3747 | Hs.27214 |
| KCNC3 | 230531_at | AB208930.1 | 3748 | Hs.467146 |
| KCNC3 | 243893_at | AB208930.1 | 3748 | Hs.467146 |
| KCNC3 | 207600_at | NM_004977.2 | 3748 | Hs.467146 |
| KCNC4 | 228436_at | NM_001039574.1 | 3749 | Hs.153521 |
| KCNC4 | 235467_s_at | NM_001039574.1 | 3749 | Hs.153521 |
| KCNC4 | 208251_at | NM_001039574.1 | 3749 | Hs.153521 |
| KCND1 | 206842_at | NM_004979.4 | 3750 | Hs.55276 |
| KCND2 | 207103_at | NM_012281.2 | 3751 | Hs.21703 |
| KCND3 | 213832_at | AA530995 | 3752 | Hs.23729 |
| KCND3 | 211301_at | NM_004980.3 | 3752 | Hs.535274 |
| KCND3 | 211827_s_at | NM_172198.1 | 3752 | Hs.535274 |
| KCNE1 | 236407_at | NM_000219.2 | 3753 | Hs.121495 |
| KCNE1 | 208514_at | NM_000219.2 | 3753 | Hs.553470 |
| KCNE1L | 220010_at | NM_012282.2 | 23630 | Hs.522753 |
| KCNE2 | 221095_s_at | NM_172201.1 | 9992 | Hs.551521 |
| KCNE3 | 222922_at | NM_005472.3 | 10008 | Hs.523899 |
| KCNE3 | 222923_s_at | NM_005472.3 | 10008 | Hs.523899 |
| KCNE3 | 227647_at | NM_005472.3 | 10008 | Hs.523899 |
| KCNE4 | 222379_at | NM_080671 | 23704 | Hs.348522 |
| KCNE4 | 1552507_at | NM_080671.1 | 23704 | Hs.348522 |
| KCNE4 | 1552508_at | NM_080671 | 23704 | Hs.348522 |
| KCNF1 | 210263_at | NM_002236.4 | 3754 | Hs.23735 |
| KCNG1 | 214595_at | NM_002237.2 | 3755 | Hs.118695 |
| KCNG1 | 211053_at | NM_172318.1 | 3755 | Hs.118695 |
| KCNG2 | 208550_x_at | NM_012283.1 | 26251 | Hs.247905 |
| KCNG3 | 1552897_a_at | NM_133329.4 | 170850 | Hs.352633 |
| KCNG3 | 1552898_a_at | NM_133329.4 | 170850 | Hs.352633 |
| KCNG4 | 1552598_at | NM_133490.2 | 93107 | Hs.335877 |
| KCNH1 | 207635_s_at | NM_172362.1 | 3756 | Hs.527656 |
| KCNH2 | 205262_at | NM_172057.1 | 3757 | Hs.188021 |
| KCNH2 | 210036_s_at | NM_172056.1 | 3757 | Hs.188021 |
| KCNH3 | 223726_at | NM_012284.1 | 23416 | Hs.64064 |
| KCNH4 | 220802_at | NM_012285.1 | 23415 | Hs.304081 |
| KCNH5 | 242502_at | NM_139318.3 | 27133 | Hs.27043 |
| KCNH5 | 1555074_a_at | NM_139318.3 | 27133 | Hs.27043 |
| KCNH5 | 1555304_a_at | NM_139318.3 | 27133 | Hs.27043 |
| KCNH6 | 221023_s_at | NM_173092.1 | 81033 | Hs.436680 |
| KCNH6 | 211045_s_at | NM_030779.2 | 81033 | Hs.436680 |
| KCNH6 | 211046_at | NM_030779 | 81033 | Hs.436680 |
| KCNH7 | 224099_at | NM_033272.2 | 90134 | Hs.567312 |
| KCNH7 | 1555316_a_at | NM_033272.2 | 90134 | Hs.567312 |
| KCNH8 | 1552742_at | NM_144633.2 | 131096 | Hs.475656 |
| KCNIP1 | 221307_at | NM_001034837.1 | 30820 | Hs.484111 |
| KCNIP2 | 221321_s_at | NM_014591.4 | 30819 | Hs.97044 |
| KCNIP2 | 223727_at | NM_173191.2 | 30819 | Hs.97044 |
| KCNIP2 | 224528_s_at | NM_014591 | 30819 | Hs.97044 |
| KCNIP2 | 237266_at | NM_014591 | 30819 | Hs.97044 |
| KCNIP2 | 1555230_a_at | NM_014591.4 | 30819 | Hs.97044 |
| KCNIP4 | 216643_at | NM_001035003 | 80333 | 543693 |
| KCNIP4 | 217438_at | NM_001035003 | 80333 | 543693 |
| KCNIP4 | 224530_s_at | NM_001035003.1 | 80333 | Hs.558552 |
| KCNIP4 | 236783_at | NM_001035003.1 | 80333 | Hs.558552 |
| KCNJ1 | 210402_at | NM_000220.2 | 3758 | Hs.527830 |
| KCNJ1 | 210403_s_at | NM_000220.2 | 3758 | Hs.527830 |
| KCNJ10 | 228581_at | NM_002241.3 | 3766 | Hs.408960 |
| KCNJ10 | 206692_at | NM_002241.3 | 3766 | Hs.408960 |
| KCNJ11 | 231740_at | NM_000525.3 | 3767 | Hs.248141 |
| KCNJ12 | 207110_at | NM_021012.3 | 3768 | Hs.2363 |
| KCNJ12 | 208566_at | XM_939575.1 | 650488 | Hs.2363 |
| KCNJ12 | 208567_s_at | NM_021012.3 | 3768 | Hs.2363 |
| KCNJ13 | 244582_at | NM_002242 | 3769 | Hs.296361 |
| KCNJ13 | 210179_at | NM_002242.2 | 3769 | Hs.467338 |
| KCNJ13 | 211427_s_at | NM_002242.2 | 3769 | Hs.467338 |
| KCNJ14 | 220776_at | NM_013348.2 | 3770 | Hs.144011 |
| KCNJ15 | 238428_at | NM_002243 | 3772 | Hs.17287 |
| KCNJ15 | 210119_at | NM_002243.3 | 3772 | Hs.411299 |
| KCNJ15 | 211806_s_at | NM_002243.3 | 3772 | Hs.411299 |
| KCNJ16 | 219564_at | NM_018658.1 | 3773 | Hs.463985 |
| KCNJ16 | 222901_s_at | NM_170742.1 | 3773 | Hs.463985 |
| KCNJ2 | 231513_at | NM_000891 | 3759 | Hs.1547 |
| KCNJ2 | 206765_at | NM_000891.2 | 3759 | Hs.1547 |
| KCNJ3 | 233059_at | NM_002239 | 3760 | Hs.199776 |
| KCNJ3 | 207141_s_at | NM_002239.2 | 3760 | Hs.199776 |
| KCNJ3 | 207142_at | NM_002239.2 | 3760 | Hs.199776 |
| KCNJ4 | 208359_s_at | NM_004981.1 | 3761 | Hs.32505 |
| KCNJ4 | 211451_s_at | NM_004981.1 | 3761 | Hs.32505 |
| KCNJ5 | 1570531_at | BC018531 | 3762 | 632109 |
| KCNJ5 | 208397_x_at | NM_000890.3 | 3762 | Hs.558350 |
| KCNJ5 | 208404_x_at | NM_000890 | 3762 | Hs.193044 |
| KCNJ5 | 211304_x_at | NM_000890 | 3762 | Hs.193044 |
| KCNJ5 | 211817_s_at | NM_000890.3 | 3762 | Hs.558350 |
| KCNJ6 | 210454_s_at | NM_002240.2 | 3763 | Hs.50927 |
| KCNJ8 | 205303_at | NM_004982.2 | 3764 | Hs.102308 |
| KCNJ8 | 205304_s_at | NM_004982.2 | 3764 | Hs.102308 |
| KCNJ9 | 207527_at | NM_004983.2 | 3765 | Hs.66726 |
| KCNK1 | 1566464_at | NM_002245 | 3775 | Hs.376874 |
| KCNK1 | 1566465_at | NM_002245 | 3775 | Hs.376874 |
| KCNK1 | 204678_s_at | NM_002245.2 | 3775 | Hs.208544 |
| KCNK1 | 204679_at | NM_002245.2 | 3775 | Hs.208544 |
| KCNK10 | 220727_at | NM_138317.1 | 54207 | Hs.365690 |
| KCNK10 | 233967_at | NM_021161 | 54207 | Hs.365690 |
| KCNK10 | 1564143_at | NM_021161 | 54207 | 592299 |
| KCNK12 | 220448_at | NM_022055.1 | 56660 | Hs.243991 |
| KCNK13 | 221325_at | NM_022054.2 | 56659 | Hs.510191 |
| KCNK15 | 220540_at | NM_022358 | 60598 | Hs.528664 |
| KCNK16 | 234554_at | NM_032115.2 | 83795 | Hs.287765 |
| KCNK17 | 224049_at | NM_031460.2 | 89822 | Hs.162282 |
| KCNK2 | 210261_at | NM_001017424.1 | 3776 | Hs.497745 |
| KCNK3 | 228127_at | NM_002246 | 3777 | Hs.24040 |
| KCNK3 | 205952_at | NM_002246.1 | 3777 | Hs.24040 |
| KCNK4 | 219883_at | XM_942456.1 | 652796 | Hs.97174 |
| KCNK4 | 224552_s_at | NM_033310.2 | 50801 | Hs.97174 |
| KCNK5 | 219615_s_at | NM_003740.3 | 8645 | Hs.444448 |
| KCNK5 | 240175_at | NM_003740 | 8645 | Hs.444448 |
| KCNK6 | 223658_at | NM_004823.1 | 9424 | Hs.240395 |
| KCNK7 | 220412_x_at | NM_033455.1 | 10089 | Hs.175218 |
| KCNK7 | 224008_s_at | NM_033348.1 | 10089 | Hs.175218 |
| KCNK7 | 224055_x_at | NM_005714.1 | 10089 | Hs.175218 |
| KCNK9 | 224072_s_at | NM_016601.2 | 51305 | Hs.493037 |
| KCNK9 | 238870_at | NM_016601 | 51305 | Hs.117010 |
| KCNMA1 | 214921_at | NM_002247 | 3778 | Hs.144795 |
| KCNMA1 | 221583_s_at | NM_001014797.1 | 3778 | Hs.144795 |
| KCNMA1 | 221584_s_at | NM_002247.2 | 3778 | Hs.144795 |
| KCNMA1 | 228414_at | NM_001014797.1 | 3778 | Hs.144795 |
| KCNMA1 | 1561962_at | NM_002247 | 3778 | Hs.354740 |
| KCNMB1 | 1554710_at | NM_004137 | 3779 | Hs.484099 |
| KCNMB1 | 209948_at | NM_004137.2 | 3779 | Hs.484099 |
| KCNMB2 | 221097_s_at | NM_181361.1 | 10242 | Hs.478368 |
| KCNMB2 | 223823_at | NM_005832.3 | 10242 | Hs.478368 |
| KCNMB3 | 221125_s_at | NM_171828.1 | 27094 | Hs.85701 |
| KCNMB3 | 231854_at | NM_014407 | 27094 | Hs.120905 |
| KCNMB3 | 235980_at | NM_014407 | 27094 | Hs.120905 |
| KCNMB4 | 222857_s_at | NM_014505.4 | 27345 | Hs.525529 |
| KCNMB4 | 235930_at | NM_014505 | 27345 | Hs.348361 |
| KCNN1 | 206231_at | NM_002248.3 | 3780 | Hs.158173 |
| KCNN2 | 220116_at | NM_021614.2 | 3781 | Hs.98280 |
| KCNN3 | 244040_at | NM_002249 | 3782 | Hs.89230 |
| KCNN3 | 205902_at | NM_002249.3 | 3782 | Hs.490765 |
| KCNN3 | 205903_s_at | NM_002249.3 | 3782 | Hs.490765 |
| KCNN4 | 204401_at | NM_002250.2 | 3783 | Hs.10082 |
| KCNQ1 | 234590_x_at | NM_000218 | 3784 | Hs.367809 |
| KCNQ1 | 204487_s_at | NM_000218.2 | 3784 | Hs.95162 |
| KCNQ1 | 211217_s_at | NM_000218.2 | 3784 | Hs.95162 |
| KCNQ2 | 205737_at | NM_004518 | 3785 | Hs.161851 |
| KCNQ2 | 210508_s_at | NM_172107.1 | 3785 | Hs.161851 |
| KCNQ2 | 211486_s_at | NM_172106.1 | 3785 | Hs.161851 |
| KCNQ3 | 228579_at | NM_004519 | 3786 | Hs.388289 |
| KCNQ3 | 1557042_at | NM_004519 | 3786 | Hs.388289 |
| KCNQ3 | 206573_at | NM_004519.2 | 3786 | Hs.374023 |
| KCNQ4 | 221083_at | NM_004700.2 | 9132 | Hs.473058 |
| KCNQ4 | 243209_at | NM_004700 | 9132 | Hs.473058 |
| KCNQ5 | 223891_at | NM_019842.2 | 56479 | Hs.98129 |
| KCNQ5 | 244106_at | NM_019842 | 56479 | Hs.371688 |
| KCNQ5 | 244623_at | BF513800 | 56479 | Hs.675919 |
| KCNS1 | 207366_at | NM_002251.3 | 3787 | Hs.117780 |
| KCNS2 | 232401_at | R16167 | 3788 | Hs.388045 |
| KCNS2 | 1554212_s_at | NM_020697.2 | 3788 | Hs.388045 |
| KCNS2 | 1554680_s_at | NM_020697.2 | 3788 | Hs.388045 |
| KCNS3 | 205968_at | NM_002252.3 | 3790 | Hs.414489 |
| KCNT1 | 1563608_a_at | NM_020822.1 | 57582 | Hs.104950 |
| KCNT1 | 1569461_at | BC035730 | 57582 | Hs.379381 |
| KCNT1 | 1569462_x_at | BC035730 | 57582 | Hs.379381 |
| KCNT2 | 244455_at | NM_198503.2 | 343450 | Hs.420016 |
| KCNU1 | 237273_at | BC028701.2 | 157855 | Hs.13861 |
| KCNV1 | 220294_at | NM_014379.2 | 27012 | Hs.13285 |
| KCNV2 | 217522_at | NM_133497 | 169522 | Hs.441357 |
| KCTD1 | 226245_at | NM_198991.1 | 284252 | Hs.526630 |
| KCTD1 | 226246_at | NM_198991.1 | 284252 | Hs.526630 |
| KCTD11 | 235857_at | NM_001002914.1 | 147040 | Hs.553609 |
| KCTD12 | 212188_at | NM_138444.2 | 115207 | Hs.109438 |
| KCTD12 | 212192_at | NM_138444.2 | 115207 | Hs.109438 |
| KCTD14 | 219545_at | NM_023930.2 | 65987 | Hs.17296 |
| KCTD14 | 58916_at | NM_023930.2 | 65987 | Hs.17296 |
| KCTD15 | 218553_s_at | NM_024076.1 | 79047 | Hs.221873 |
| KCTD15 | 222664_at | AK025590.1 | 79047 | Hs.221873 |
| KCTD15 | 228683_s_at | NM_024076.1 | 79047 | Hs.221873 |
| KCTD16 | 233234_at | NM_020768.1 | 57528 | Hs.161256 |
| KCTD17 | 1554566_at | BC009961.2 | 79734 | Hs.517597 |
| KCTD17 | 205561_at | NM_024681.1 | 79734 | Hs.517597 |
| KCTD18 | 226493_at | NM_152387.2 | 130535 | Hs.379185 |
| KCTD18 | 1553136_at | NM_175916 | 130535 | Hs.379185 |
| KCTD19 | 244538_at | BC070103.1 | 146212 | Hs.299127 |
| KCTD2 | 34858_at | NM_015353.1 | 23510 | Hs.514468 |
| KCTD2 | 212564_at | NM_015353.1 | 23510 | Hs.514468 |
| KCTD21 | 229873_at | NM_001029859.1 | 283219 | Hs.567330 |
| KCTD3 | 217894_at | NM_016121.3 | 51133 | Hs.335139 |
| KCTD4 | 239787_at | NM_198404.1 | 386618 | Hs.23406 |
| KCTD4 | 240512_x_at | NM_198404.1 | 386618 | Hs.23406 |
| KCTD5 | 218474_s_at | NM_018992.1 | 54442 | Hs.61960 |
| KCTD5 | 222645_s_at | NM_018992.1 | 54442 | Hs.61960 |
| KCTD6 | 238001_at | NM_153331.2 | 200845 | Hs.13982 |
| KCTD6 | 238077_at | NM_153331.2 | 200845 | Hs.13982 |
| KCTD7 | 1553717_at | AK055201.1 | 154881 | Hs.520914 |
| KCTD7 | 1555569_a_at | NM_153033.1 | 154881 | Hs.520914 |
| KCTD9 | 218823_s_at | NM_017634.2 | 54793 | Hs.224505 |
| KCTD9 | 224316_at | AF130091.1 | 54793 | Hs.224505 |
| KDR | 203934_at | NM_002253.1 | 3791 | Hs.479756 |
| KEL | 206077_at | NM_000420.2 | 3792 | Hs.368588 |
| KERA | 220504_at | NM_007035.3 | 11081 | Hs.125750 |
| KIAA0090 | 215991_s_at | NM_015047 | 23065 | Hs.439200 |
| KIAA0090 | 1556186_s_at | NM_015047 | 23065 | Hs.439200 |
| KIAA0090 | 212394_at | NM_015047 | 23065 | Hs.439200 |
| KIAA0090 | 212395_s_at | NM_015047.1 | 23065 | Hs.439200 |
| KIAA0090 | 212396_s_at | NM_015047.1 | 23065 | Hs.439200 |
| KIAA0195 | 202650_s_at | NM_014738.2 | 9772 | Hs.514474 |
| KIAA0241 | 1555055_at | BC027724.2 | 23080 | Hs.128056 |
| KIAA0241 | 1563452_at | AL833560 | 23080 | Hs.134792 |
| KIAA0241 | 212471_at | NM_015060.1 | 23080 | Hs.128056 |
| KIAA0241 | 212474_at | D87682.1 | 23080 | Hs.128056 |
| KIAA0241 | 212475_at | D87682.1 | 23080 | Hs.128056 |
| KIAA0241 | 244355_at | AI090310 | 23080 | Hs.134792 |
| KIAA0247 | 202181_at | NM_014734.2 | 9766 | Hs.440025 |
| KIAA0286 | 212619_at | NM_015257.1 | 23306 | Hs.533787 |
| KIAA0286 | 212621_at | NM_015257.1 | 23306 | Hs.533787 |
| KIAA0317 | 244137_at | NM_014821 | 9870 | Hs.434432 |
| KIAA0317 | 1554989_at | NM_014821 | 9870 | Hs.497417 |
| KIAA0317 | 1566978_at | AF085823 | 9870 | 497417 |
| KIAA0317 | 1566979_at | AF085823 | 9870 | 497417 |
| KIAA0317 | 202128_at | NM_001039479.1 | 9870 | Hs.497417 |
| KIAA0319 | 206017_at | NM_014809.2 | 9856 | Hs.26441 |
| KIAA0319L | 217929_s_at | NM_024874.3 | 79932 | Hs.456507 |
| KIAA0319L | 222468_at | NM_024874.3 | 79932 | Hs.456507 |
| KIAA0527 | 214954_at | XM_171054.4 | 26032 | Hs.196647 |
| KIAA0830 | 212570_at | XM_290546.4 | 23052 | Hs.167115 |
| KIAA0830 | 212573_at | XM_290546.4 | 23052 | Hs.167115 |
| KIAA0892 | 216926_s_at | NM_015329.2 | 23383 | Hs.112751 |
| KIAA0892 | 217652_at | AW157731 | 23383 | Hs.112751 |
| KIAA0892 | 227465_at | NM_015329.2 | 23383 | Hs.112751 |
| KIAA0892 | 212505_s_at | NM_015329.2 | 23383 | Hs.112751 |
| KIAA0922 | 209760_at | NM_015196.2 | 23240 | Hs.205572 |
| KIAA1024 | 215081_at | NM_015206.1 | 23251 | Hs.301654 |
| KIAA1109 | 214929_s_at | NM_032202 | 84162 | Hs.408142 |
| KIAA1109 | 216294_s_at | NM_032202 | 84162 | Hs.408142 |
| KIAA1109 | 233096_at | AB037792.1 | 84162 | Hs.408142 |
| KIAA1109 | 1553792_at | NM_032202 | 84162 | Hs.408142 |
| KIAA1109 | 1553793_a_at | NM_032202 | 84162 | Hs.408142 |
| KIAA1109 | 212779_at | NM_032202 | 84162 | Hs.408142 |
| KIAA1161 | 231185_at | NM_020702.1 | 57462 | Hs.522083 |
| KIAA1161 | 232244_at | AB032987.2 | 57462 | Hs.522083 |
| KIAA1212 | 219387_at | AF112218.1 | 55704 | Hs.292925 |
| KIAA1212 | 221078_s_at | NM_018084.3 | 55704 | Hs.292925 |
| KIAA1212 | 225045_at | NM_018084.3 | 55704 | Hs.292925 |
| KIAA1212 | 238759_at | NM_018084 | 55704 | Hs.292925 |
| KIAA1212 | 239233_at | NM_018084 | 55704 | Hs.292925 |
| KIAA1244 | 231856_at | NM_020340.2 | 57221 | Hs.194408 |
| KIAA1305 | 220911_s_at | NM_025081 | 57523 | Hs.288348 |
| KIAA1324 | 221874_at | NM_020775 | 57535 | Hs.262811 |
| KIAA1324 | 226248_s_at | NM_020775.2 | 57535 | Hs.262811 |
| KIAA1324 | 243349_at | NM_020775.2 | 57535 | Hs.262811 |
| KIAA1324L | 235301_at | NM_152748.2 | 222223 | Hs.208093 |
| KIAA1324L | 244317_at | NM_152748.2 | 222223 | Hs.208093 |
| KIAA1409 | 229550_at | NM_020818.2 | 57578 | Hs.126561 |
| KIAA1423 | 225127_at | BF217531 | 57583 | Hs.99145 |
| KIAA1432 | 223825_at | AL136875 | 57589 | Hs.211520 |
| KIAA1432 | 223826_s_at | NM_020829.1 | 57589 | Hs.211520 |
| KIAA1432 | 226221_at | AL138104 | 57589 | Hs.211520 |
| KIAA1432 | 226222_at | AB037853 | 57589 | Hs.211520 |
| KIAA1529 | 229627_at | NM_020893.1 | 57653 | Hs.435629 |
| KIAA1529 | 229628_s_at | NM_020893.1 | 57653 | Hs.435629 |
| KIAA1549 | 223575_at | XM_935389.1 | 57670 | Hs.490294 |
| KIAA1644 | 221901_at | XM_936510.1 | 85352 | Hs.6829 |
| KIAA1644 | 52837_at | XM_936510.1 | 85352 | Hs.6829 |
| KIAA1679 | 232327_at | XM_937523.1 | 80731 | Hs.68533 |
| KIAA1715 | 225717_at | NM_030650.1 | 80856 | Hs.209561 |
| KIAA1715 | 225718_at | NM_030650.1 | 80856 | Hs.209561 |
| KIAA1715 | 229173_at | CR936742.1 | 80856 | Hs.209561 |
| KIAA1754 | 225582_at | NM_033397.2 | 85450 | Hs.523252 |
| KIAA1754L | 240037_at | NM_001008949.1 | 150771 | Hs.65009 |
| KIAA1772 | 220340_at | NM_024935.2 | 80000 | Hs.54838 |
| KIAA1772 | 233977_at | AB051559.1 | 80000 | Hs.54838 |
| KIAA1797 | 218503_at | NM_017794.2 | 54914 | Hs.408652 |
| KIAA1822 | 1556564_at | AK095603 | 84439 | Hs.288522 |
| KIAA1822L | 220283_at | NM_024746.2 | 79802 | Hs.123515 |
| KIAA1913 | 234994_at | NM_052913 | 114801 | Hs.172870 |
| KIAA1919 | 232139_s_at | AB067506.1 | 91749 | Hs.400572 |
| KIAA1919 | 238828_at | AB067506.1 | 91749 | Hs.400572 |
| KIAA1919 | 242851_at | AB067506.1 | 91749 | Hs.400572 |
| KIAA2013 | 224706_at | NM_138346.1 | 90231 | Hs.520094 |
| KIAA2013 | 224708_at | BC004501.2 | 90231 | Hs.520094 |
| KIAA2013 | 1555933_at | AB095933.1 | 90231 | Hs.520094 |
| KIAA2024 | 244121_at | NM_172070 | 130507 | Hs.379548 |
| KIDINS220 | 214932_at | AL133620.1 | 57498 | Hs.9873 |
| KIDINS220 | 1557246_at | AA992480 | 57498 | Hs.9873 |
| KIDINS220 | 212162_at | NM_020738.1 | 57498 | Hs.9873 |
| KIDINS220 | 212163_at | NM_020738.1 | 57498 | Hs.9873 |
| KIR2DL1 | 210890_x_at | NM_014218.1 | 3802 | Hs.258612 |
| KIR2DL1 | 211397_x_at | NM_014218.1 | 3802 | Hs.258612 |
| KIR2DL3 | 208179_x_at | NM_015868.2 | 3804 | Hs.258612 |
| KIR2DL4 | 208426_x_at | NM_002255.3 | 3805 | Hs.166085 |
| KIR2DL4 | 211242_x_at | NM_002255.3 | 3805 | Hs.166085 |
| KIR2DL4 | 211245_x_at | NM_002255.3 | 3805 | Hs.166085 |
| KIR2DL5B | 211410_x_at | NM_001018081.1 | 553128 | Hs.375019 |
| KIR2DS1 | 208198_x_at | NM_014512.1 | 3806 | Hs.512574 |
| KIR2DS4 | 216552_x_at | NM_178228.2 | 3809 | Hs.258612 |
| KIR2DS4 | 211532_x_at | NM_012314.2 | 3809 | Hs.258612 |
| KIR2DS5 | 208122_x_at | NM_014513.1 | 3810 | Hs.375019 |
| KIR2DS5 | 208203_x_at | NM_014513.1 | 3810 | Hs.375019 |
| KIR3DL1 | 211389_x_at | NM_013289.1 | 3811 | Hs.278457 |
| KIR3DL1 | 211687_x_at | NM_013289.1 | 3811 | Hs.278457 |
| KIR3DL2 | 216907_x_at | NM_006737.1 | 3812 | Hs.380156 |
| KIR3DL2 | 217318_x_at | NM_001015070 | 115653 | 645224 |
| KIR3DL2 | 207313_x_at | NM_006737.1 | 3812 | Hs.380156 |
| KIR3DL2 | 207314_x_at | NM_006737.1 | 3812 | Hs.380156 |
| KIR3DL2 | 211688_x_at | NM_006737.1 | 3812 | Hs.380156 |
| KIR3DL3 | 216676_x_at | NM_153443.2 | 115653 | Hs.278457 |
| KIRREL | 220825_s_at | NM_018240.3 | 55243 | Hs.272234 |
| KIRREL | 225303_at | AK090554.1 | 55243 | Hs.272234 |
| KIRREL | 232467_at | NM_018240 | 55243 | Hs.272234 |
| KIRREL2 | 223755_at | NM_199180.1 | 84063 | Hs.145729 |
| KIRREL3 | 240402_at | NM_032531 | 84623 | Hs.302350 |
| KISS1 | 205563_at | NM_002256.2 | 3814 | Hs.95008 |
| KIT | 205051_s_at | NM_000222.1 | 3815 | Hs.479754 |
| KITLG | 226534_at | NM_000899.3 | 4254 | Hs.1048 |
| KITLG | 207029_at | NM_000899.3 | 4254 | Hs.1048 |
| KITLG | 211124_s_at | NM_000899.3 | 4254 | Hs.1048 |
| KL | 205978_at | NM_004795.2 | 9365 | Hs.524953 |
| KLB | 235708_at | NM_175737 | 152831 | Hs.90756 |
| KLB | 244276_at | BC033021.1 | 152831 | Hs.90756 |
| KLHL11 | 220657_at | NM_018143.1 | 55175 | Hs.558507 |
| KLK10 | 215808_at | NM_002776 | 5655 | Hs.275464 |
| KLK10 | 209792_s_at | NM_002776.3 | 5655 | Hs.275464 |
| KLK11 | 205470_s_at | NM_006853.2 | 11012 | Hs.57771 |
| KLK12 | 220782_x_at | NM_019598.2 | 43849 | Hs.411572 |
| KLK12 | 233586_s_at | NM_019598.2 | 43849 | Hs.411572 |
| KLK12 | 234316_x_at | NM_145894.1 | 43849 | Hs.411572 |
| KLK13 | 217315_s_at | NM_015596.1 | 26085 | Hs.165296 |
| KLK13 | 205783_at | NM_015596.1 | 26085 | Hs.165296 |
| KLK14 | 220573_at | NM_022046.4 | 43847 | Hs.283925 |
| KLK15 | 221462_x_at | NM_017509.2 | 55554 | Hs.164469 |
| KLK15 | 233477_at | NM_017509.2 | 55554 | Hs.164469 |
| KLK15 | 234495_at | NM_017509 | 55554 | Hs.164469 |
| KLK15 | 234966_at | NM_017509 | 55554 | Hs.250770 |
| KLK3 | 231629_x_at | NM_001648 | 354 | Hs.171995 |
| KLK3 | 204582_s_at | NM_001030047.1 | 354 | Hs.171995 |
| KLK3 | 204583_x_at | NM_001030049.1 | 354 | Hs.171995 |
| KLK4 | 224062_x_at | NM_004917.3 | 9622 | Hs.218366 |
| KLK4 | 231782_s_at | NM_004917 | 9622 | Hs.218366 |
| KLK4 | 233854_x_at | NM_004917 | 9622 | Hs.218366 |
| KLK4 | 1555697_at | NM_004917 | 9622 | Hs.218366 |
| KLK4 | 1555737_a_at | NM_004917.3 | 9622 | Hs.218366 |
| KLK5 | 222242_s_at | NM_012427.3 | 25818 | Hs.50915 |
| KLK7 | 239381_at | NM_139277.1 | 5650 | Hs.151254 |
| KLK7 | 205778_at | NM_005046.2 | 5650 | Hs.151254 |
| KLK8 | 1552319_a_at | NM_007196.2 | 11202 | Hs.104570 |
| KLK8 | 206125_s_at | NM_007196.2 | 11202 | Hs.104570 |
| KLK9 | 233687_s_at | NM_012315.1 | 284366 | Hs.448942 |
| KLKB1 | 206541_at | NM_000892.3 | 3818 |  |
| KLRB1 | 214470_at | NM_002258.2 | 3820 | Hs.169824 |
| KLRB1 | 242628_at | NM_002258 | 3820 | Hs.169824 |
| KLRC1 | 206785_s_at | NM_002259.3 | 3821 | Hs.512576 |
| KLRC3 | 207723_s_at | NM_002261.2 | 3823 | Hs.74082 |
| KLRC4 | 210690_at | NM_013431 | 8302 | Hs.268510 |
| KLRD1 | 207795_s_at | NM_002262.2 | 3824 | Hs.524251 |
| KLRD1 | 207796_x_at | NM_002262.2 | 3824 | Hs.524251 |
| KLRD1 | 210606_x_at | NM_002262.2 | 3824 | Hs.524251 |
| KLRF1 | 220646_s_at | NM_016523.1 | 51348 | Hs.183125 |
| KLRG1 | 210288_at | NM_005810.3 | 10219 | Hs.558446 |
| KLRK1 | 1555691_a_at | NM_007360.1 | 22914 | Hs.387787 |
| KLRK1 | 205821_at | NM_007360.1 | 22914 | Hs.387787 |
| KNCN | 1564044_at | NM_182516.1 | 148930 | Hs.350764 |
| KNG1 | 206054_at | NM_000893.2 | 3827 | Hs.77741 |
| KREMEN1 | 224534_at | NM_001039571.1 | 83999 | Hs.229335 |
| KREMEN1 | 227250_at | NM_001039570.1 | 83999 | Hs.229335 |
| KREMEN1 | 234843_s_at | NM_001039570.1 | 83999 | Hs.229335 |
| KREMEN1 | 235370_at | NM_001039570.1 | 83999 | Hs.229335 |
| KREMEN1 | 243029_at | NM_001039570.1 | 83999 | Hs.229335 |
| KREMEN2 | 219692_at | NM_172229.1 | 79412 | Hs.534484 |
| KRTAP1-1 | 220976_s_at | NM_030967.2 | 81851 | Hs.247934 |
| KRTAP1-3 | 220978_at | NM_030966.1 | 81850 | Hs.551371 |
| KRTAP1-3 | 234880_x_at | NM_030966.1 | 81850 | Hs.551371 |
| KRTAP5-8 | 208532_x_at | NM_021046.1 | 57830 | Hs.445245 |
| KRTCAP3 | 235148_at | NM_173853.2 | 200634 | Hs.59509 |
| KRTDAP | 230835_at | NM_207392.1 | 388533 | Hs.112457 |
| KTN1 | 214709_s_at | NM_182926.1 | 3895 | Hs.509414 |
| KTN1 | 200914_x_at | NM_182926.1 | 3895 | Hs.509414 |
| KTN1 | 200915_x_at | NM_182926.1 | 3895 | Hs.509414 |
| L1CAM | 204584_at | NM_000425 | 3897 | Hs.522818 |
| L1CAM | 204585_s_at | NM_024003.1 | 3897 | Hs.522818 |
| LACRT | 224540_at | NM_033277.1 | 90070 | Hs.307096 |
| LAD1 | 216641_s_at | NM_005558.3 | 3898 | Hs.519035 |
| LAD1 | 203287_at | NM_005558.3 | 3898 | Hs.519035 |
| LAG3 | 206486_at | NM_002286.4 | 3902 | Hs.409523 |
| LAIR1 | 208071_s_at | NM_002287.3 | 3903 | Hs.467288 |
| LAIR1 | 210644_s_at | NM_002287.3 | 3903 | Hs.467288 |
| LAIR2 | 207509_s_at | NM_002288.3 | 3904 | Hs.43803 |
| LALBA | 207816_at | NM_002289.2 | 3906 | Hs.72938 |
| LAMA1 | 222346_at | NM_005559 | 284217 | Hs.270364 |
| LAMA1 | 227048_at | NM_005559.2 | 284217 | Hs.270364 |
| LAMA2 | 213519_s_at | NM_000426.2 | 3908 | Hs.200841 |
| LAMA2 | 216839_at | NM_000426 | 3908 | Hs.200841 |
| LAMA2 | 216840_s_at | NM_000426.2 | 3908 | Hs.200841 |
| LAMA2 | 205116_at | NM_000426.2 | 3908 | Hs.200841 |
| LAMA3 | 234608_at | AK024889.1 | 3909 | Hs.436367 |
| LAMA3 | 234719_at | NM_000227 | 3909 | 436367 |
| LAMA3 | 1560078_at | NM_198129.1 | 3909 | Hs.436367 |
| LAMA3 | 1563772_a_at | NM_198129.1 | 3909 | Hs.436367 |
| LAMA3 | 1568879_a_at | NM_198129.1 | 3909 | Hs.436367 |
| LAMA3 | 203726_s_at | NM_000227.2 | 3909 | Hs.436367 |
| LAMA4 | 216081_at | NM_002290 | 3910 | Hs.213861 |
| LAMA4 | 202202_s_at | NM_002290.2 | 3910 | Hs.213861 |
| LAMA4 | 210089_s_at | NM_002290.2 | 3910 | Hs.213861 |
| LAMA4 | 210989_at | NM_002290 | 3910 | Hs.437536 |
| LAMA4 | 210990_s_at | NM_002290.2 | 3910 | Hs.213861 |
| LAMA5 | 210150_s_at | NM_005560.3 | 3911 | Hs.90107 |
| LAMB2 | 216264_s_at | NM_002292.2 | 3913 | Hs.439726 |
| LAMB3 | 209270_at | NM_000228 | 3914 | Hs.497636 |
| LAMB4 | 215516_at | NM_007356.1 | 22798 | Hs.62022 |
| LAMB4 | 216622_at | AF029325 | 22798 | Hs.62022 |
| LAMB4 | 234334_s_at | NM_007356.1 | 22798 | Hs.62022 |
| LAMC1 | 200770_s_at | NM_002293.2 | 3915 | Hs.497039 |
| LAMC1 | 200771_at | NM_002293.2 | 3915 | Hs.497039 |
| LAMC2 | 202267_at | NM_005562.1 | 3918 | Hs.530509 |
| LAMC2 | 207517_at | NM_018891.1 | 3918 | Hs.530509 |
| LAMC3 | 219407_s_at | NM_006059.2 | 10319 | Hs.201805 |
| LAMC3 | 232558_at | NM_006059 | 10319 | Hs.201805 |
| LAMP1 | 213728_at | NM_005561 | 3916 | Hs.150101 |
| LAMP1 | 201551_s_at | NM_005561.2 | 3916 | Hs.494419 |
| LAMP1 | 201552_at | NM_005561.2 | 3916 | Hs.494419 |
| LAMP1 | 201553_s_at | NM_005561.2 | 3916 | Hs.494419 |
| LAMP2 | 200821_at | NM_013995.1 | 3920 | Hs.496684 |
| LAMP2 | 203041_s_at | NM_002294.1 | 3920 | Hs.496684 |
| LAMP2 | 203042_at | NM_002294.1 | 3920 | Hs.496684 |
| LANCL1 | 202019_s_at | NM_006055.1 | 10314 | Hs.13351 |
| LANCL1 | 202020_s_at | NM_006055.1 | 10314 | Hs.13351 |
| LAPTM4A | 200673_at | NM_014713.3 | 9741 | Hs.467807 |
| LAPTM4B | 214039_s_at | NM_018407.4 | 55353 | Hs.492314 |
| LAPTM4B | 1554679_a_at | NM_018407.4 | 55353 | Hs.492314 |
| LAPTM4B | 208029_s_at | NM_018407.4 | 55353 | Hs.492314 |
| LAPTM4B | 208767_s_at | NM_018407.4 | 55353 | Hs.492314 |
| LAPTM5 | 201720_s_at | NM_006762.1 | 7805 | Hs.371021 |
| LAPTM5 | 201721_s_at | NM_006762.1 | 7805 | Hs.371021 |
| LAT | 209881_s_at | NM_001014987.1 | 27040 | Hs.498997 |
| LAT | 211005_at | NM_001014987.1 | 27040 | Hs.498997 |
| LAT2 | 221581_s_at | NM_014146.3 | 7462 | Hs.56607 |
| LAT2 | 211768_at | BC006080.1 | 7462 | Hs.56607 |
| LAX1 | 207734_at | NM_017773.2 | 54900 | Hs.272794 |
| LAYN | 228080_at | NM_178834.2 | 143903 | Hs.503831 |
| LBP | 214461_at | NM_004139.2 | 3929 | Hs.154078 |
| LBP | 211652_s_at | NM_004139.2 | 3929 | Hs.154078 |
| LCAT | 204428_s_at | NM_000229.1 | 3931 | Hs.387239 |
| LCK | 204890_s_at | NM_005356.2 | 3932 | Hs.470627 |
| LCK | 204891_s_at | NM_005356.2 | 3932 | Hs.470627 |
| LCN1 | 207930_at | NM_002297.2 | 3933 | Hs.530311 |
| LCN10 | 238071_at | AK091788.1 | 414332 | Hs.98132 |
| LCN12 | 230717_at | BC041168.1 | 286256 | Hs.440519 |
| LCN2 | 212531_at | NM_005564 | 3934 | Hs.204238 |
| LCN6 | 1559864_at | BC040937.1 | 158062 | Hs.98132 |
| LCN8 | 239369_at | NM_178469 | 138307 | Hs.323991 |
| LCN8 | 1569614_s_at | AK126902.1 | 138307 | Hs.323991 |
| LCT | 206945_at | NM_002299.2 | 3938 | Hs.551506 |
| LDLR | 217173_s_at | NM_000527.2 | 3949 | Hs.213289 |
| LDLR | 202067_s_at | NM_000527.2 | 3949 | Hs.213289 |
| LDLR | 202068_s_at | NM_000527.2 | 3949 | Hs.213289 |
| LDLR | 211411_at | NM_000527 | 3949 | Hs.213289 |
| LDLRAD3 | 234985_at | BC042754.1 | 143458 | Hs.205865 |
| LEAP-2 | 1552362_a_at | NM_052971 | 116842 | Hs.337588 |
| LECT1 | 206309_at | NM_001011705.1 | 11061 | Hs.421391 |
| LECT2 | 207409_at | NM_002302.2 | 3950 | Hs.512580 |
| LEFTY1 | 206268_at | NM_020997.2 | 10637 | Hs.278239 |
| LEFTY2 | 206012_at | NM_003240.2 | 7044 | Hs.520187 |
| LENG4 | 209179_s_at | NM_024298.2 | 79143 | Hs.467279 |
| LENG4 | 211037_s_at | NM_024298 | 79143 | Hs.467279 |
| LEP | 207092_at | NM_000230.1 | 3952 | Hs.194236 |
| LEPR | 227095_at | NM_002303 | 3953 | Hs.23581 |
| LEPR | 1556919_at | NM_002303 | 3953 | Hs.23581 |
| LEPR | 202377_at | NM_002303 | 3953 | Hs.23581 |
| LEPR | 207255_at | NM_002303.3 | 3953 | Hs.23581 |
| LEPR | 209894_at | NM_001003679.1 | 3953 | Hs.23581 |
| LEPR | 211354_s_at | NM_001003679.1 | 3953 | Hs.23581 |
| LEPR | 211355_x_at | NM_001003680.1 | 3953 | Hs.23581 |
| LEPR | 211356_x_at | NM_001003680.1 | 3953 | Hs.23581 |
| LEPRE1 | 220750_s_at | NM_022356.2 | 64175 | Hs.437656 |
| LEPROT | 202378_s_at | NM_017526.2 | 54741 | Hs.23581 |
| LEPROTL1 | 202594_at | NM_015344.1 | 23484 | Hs.146585 |
| LEPROTL1 | 202595_s_at | NM_015344.1 | 23484 | Hs.146585 |
| LETM1 | 218939_at | NM_012318.1 | 3954 | Hs.120165 |
| LETM1 | 233439_at | NM_012318 | 3954 | Hs.120165 |
| LETMD1 | 207170_s_at | NM_001024668.1 | 25875 | Hs.370457 |
| LGALS3 | 1557197_a_at | AB209391.1 | 81625 | Hs.531081 |
| LGALS3 | 208949_s_at | NM_002306.1 | 3958 | Hs.531081 |
| LGALS3BP | 200923_at | NM_005567.2 | 3959 | Hs.514535 |
| LGALS4 | 204272_at | NM_006149.2 | 3960 | Hs.5302 |
| LGALS8 | 234611_at | XM_928731.1 | 645721 | Hs.4082 |
| LGALS8 | 244381_at | NM_006499 | 3964 | Hs.4082 |
| LGALS8 | 208934_s_at | NM_006499.3 | 3964 | Hs.4082 |
| LGALS8 | 208935_s_at | NM_006499 | 3964 | Hs.4082 |
| LGALS8 | 208936_x_at | NM_006499.3 | 3964 | Hs.4082 |
| LGALS8 | 210731_s_at | NM_006499 | 3964 | Hs.4082 |
| LGALS8 | 210732_s_at | NM_006499.3 | 3964 | Hs.4082 |
| LGALS9 | 203236_s_at | NM_002308 | 3965 | Hs.81337 |
| LGI1 | 206349_at | NM_005097.1 | 9211 | Hs.533670 |
| LGI2 | 219699_at | NM_018176.2 | 55203 | Hs.12488 |
| LGI3 | 238061_at | NM_139278.2 | 203190 | Hs.33470 |
| LGI4 | 227821_at | NM_139284.1 | 163175 | Hs.65256 |
| LGI4 | 242670_at | NM_139284 | 163175 | Hs.65256 |
| LGICZ1 | 1555550_at | NM_180990.2 | 353174 | Hs.514496 |
| LGR4 | 218326_s_at | NM_018490.1 | 55366 | Hs.502176 |
| LGR4 | 222596_s_at | NM_018490.1 | 55366 | Hs.502176 |
| LGR5 | 213880_at | AK075399.1 | 8549 | Hs.172176 |
| LGR5 | 210393_at | NM_003667.2 | 8549 | Hs.172176 |
| LGR6 | 227819_at | NM_021636.2 | 59352 | Hs.497402 |
| LGR8 | 1553326_at | NM_130806.2 | 122042 | Hs.531002 |
| LHB | 214471_x_at | NM_000894.2 | 3972 | Hs.154704 |
| LHCGR | 207240_s_at | NM_000233 | 3973 | Hs.468490 |
| LHFP | 218656_s_at | NM_005780 | 10186 | Hs.507798 |
| LHFP | 231411_at | NM_005780 | 10186 | Hs.93765 |
| LHFPL1 | 236048_at | NM_178175 | 340596 | Hs.297420 |
| LHFPL2 | 212658_at | NM_005779 | 10184 | Hs.79299 |
| LHFPL3 | 236761_at | AK124251.1 | 375612 | Hs.124316 |
| LHFPL5 | 1555043_at | AK126841.1 | 222662 | Hs.367947 |
| LIF | 1554305_at | BC014233.2 | 91370 | Hs.2250 |
| LIF | 205266_at | NM_002309.2 | 3976 | Hs.2250 |
| LIFR | 225571_at | NM_002310.3 | 3977 | Hs.133421 |
| LIFR | 225575_at | NM_002310.3 | 3977 | Hs.133421 |
| LIFR | 227771_at | NM_002310.3 | 3977 | Hs.133421 |
| LIFR | 205876_at | NM_002310.3 | 3977 | Hs.133421 |
| LILRA1 | 207872_s_at | NM_006863.1 | 11024 | Hs.534393 |
| LILRA1 | 210660_at | AF025529.1 | 11024 | Hs.534393 |
| LILRA2 | 207857_at | NM_006866.1 | 11027 | Hs.534394 |
| LILRA2 | 211100_x_at | NM_006866.1 | 11027 | Hs.534394 |
| LILRA2 | 211101_x_at | NM_006866.1 | 11027 | Hs.534394 |
| LILRA2 | 211102_s_at | NM_006866.1 | 11027 | Hs.534394 |
| LILRA3 | 206881_s_at | NM_006865.2 | 11026 | Hs.113277 |
| LILRA4 | 210313_at | NM_012276.3 | 23547 | Hs.406708 |
| LILRA5 | 215838_at | NM_021250.2 | 353514 | Hs.512233 |
| LILRA5 | 1555634_a_at | NM_181986.1 | 353514 | Hs.512233 |
| LILRA5 | 1555643_s_at | NM_021250.2 | 353514 | Hs.512233 |
| LILRA6 | 208594_x_at | NM_024318.1 | 79168 | Hs.554799 |
| LILRB1 | 229937_x_at | NM_006669 | 10859 | Hs.149924 |
| LILRB1 | 207104_x_at | NM_006669.2 | 10859 | Hs.149924 |
| LILRB1 | 211336_x_at | NM_006669.2 | 10859 | Hs.149924 |
| LILRB2 | 207697_x_at | NM_005874.1 | 10288 | Hs.554799 |
| LILRB2 | 210146_x_at | AF004231.1 | 10288 | Hs.534386 |
| LILRB3 | 210225_x_at | NM_006864.1 | 11025 | Hs.149924 |
| LILRB3 | 210784_x_at | NM_006864.1 | 11025 | Hs.149924 |
| LILRB3 | 211133_x_at | NM_006864.1 | 11025 | Hs.149924 |
| LILRB3 | 211135_x_at | NM_006864.1 | 11025 | Hs.149924 |
| LILRB4 | 210152_at | NM_006847.2 | 11006 | Hs.67846 |
| LILRB5 | 206856_at | NM_006840.2 | 10990 | Hs.306230 |
| LIM2 | 220519_s_at | NM_030657.2 | 3982 | Hs.162754 |
| LIME1 | 219541_at | NM_017806.1 | 54923 | Hs.233220 |
| LIMS2 | 220765_s_at | NM_017980.2 | 55679 | Hs.469881 |
| LIN7A | 240027_at | NM_004664 | 8825 | Hs.144333 |
| LIN7A | 241652_x_at | NM_004664 | 8825 | Hs.144333 |
| LIN7A | 206440_at | NM_004664.2 | 8825 | Hs.144333 |
| LIPC | 1562038_at | AK091917 | 3990 | 188630 |
| LIPC | 206606_at | NM_000236.1 | 3990 | Hs.188630 |
| LIPG | 219181_at | NM_006033.2 | 9388 | Hs.465102 |
| LIPH | 235871_at | NM_139248.2 | 200879 | Hs.68864 |
| LIPI | 242178_at | NM_198996.2 | 149998 | Hs.139907 |
| LMAN1L | 220420_at | NM_021819.2 | 79748 | Hs.187694 |
| LMAN2 | 215656_at | NM_006816 | 10960 | Hs.75864 |
| LMAN2 | 200805_at | NM_006816.1 | 10960 | Hs.75864 |
| LMAN2L | 221274_s_at | NM_030805.1 | 81562 | Hs.158852 |
| LMBR1 | 222505_at | AL833934.1 | 64327 | Hs.209989 |
| LMBR1 | 224036_s_at | AF063592.1 | 64327 | Hs.209989 |
| LMBR1 | 224410_s_at | NM_022458.2 | 64327 | Hs.209989 |
| LMBR1L | 220036_s_at | NM_018113.1 | 55716 | Hs.272838 |
| LMBRD1 | 218191_s_at | NM_018368.2 | 55788 | Hs.271643 |
| LMBRD2 | 232893_at | NM_001007527.1 | 92255 | Hs.294103 |
| LMLN | 229611_at | NM_033029 | 89782 | Hs.432613 |
| LMLN | 238037_at | NM_033029 | 89782 | Hs.432613 |
| LMLN | 244881_at | NM_033029 | 89782 | Hs.518540 |
| LMLN | 1553284_s_at | NM_033029.1 | 89782 | Hs.518540 |
| LMTK2 | 206223_at | NM_014916.2 | 22853 | Hs.444179 |
| LMTK3 | 1557103_a_at | XM_055866.7 | 114783 | Hs.207426 |
| LNPEP | 231866_at | AB208883.1 | 4012 | Hs.527199 |
| LNPEP | 236728_at | AK096804.1 | 4012 | Hs.527199 |
| LNPEP | 207904_s_at | NM_005575.2 | 4012 | Hs.527199 |
| LOC113444 | 239510_at | NM_138428 | 113444 | Hs.27160 |
| LOC118430 | 1553602_at | NM_058173 | 118430 | Hs.348419 |
| LOC124446 | 228513_at | AA496243 | 124446 | Hs.291623 |
| LOC124491 | 227586_at | NM_145254 | 124491 | Hs.487510 |
| LOC124491 | 230414_s_at | NM_145254 | 124491 | Hs.236456 |
| LOC124512 | 225808_at | XM_497558.2 | 124512 | Hs.74655 |
| LOC126147 | 1553890_s_at | NM_145807.1 | 126147 | Hs.326217 |
| LOC126147 | 1558247_s_at | NM_145807 | 126147 | Hs.326217 |
| LOC129642 | 213288_at | NM_138799 | 129642 | Hs.90797 |
| LOC130576 | 228360_at | NM_177964.3 | 130576 | Hs.357567 |
| LOC134145 | 225668_at | AK000674 | 134145 | Hs.409027 |
| LOC134145 | 225670_at | NM_199133.1 | 134145 | Hs.481569 |
| LOC143891 | 1557180_at | AB096240.1 | 399947 | Hs.172982 |
| LOC143891 | 1557181_s_at | AB096240.1 | 399947 | Hs.172982 |
| LOC145053 | 241672_at | BG413606 | 145053 | Hs.422375 |
| LOC146177 | 238864_at | NM_175059 | 146177 | Hs.10697 |
| LOC148710 | 214903_at | AF070580 | 148710 | Hs.25422 |
| LOC150084 | 243027_at | XM_945811.1 | 150084 | Hs.422120 |
| LOC153470 | 1554195_a_at | BC021680.1 | 389336 | Hs.173059 |
| LOC162073 | 1568619_s_at | XM_928728.1 | 162073 | Hs.530899 |
| LOC162073 | 227514_at | XM_928728.1 | 162073 | Hs.530899 |
| LOC162073 | 227954_at | XM_928728.1 | 162073 | Hs.530899 |
| LOC162073 | 227956_at | AI458417 | 162073 | Hs.28890 |
| LOC196264 | 1570584_at | BG037101 | 196264 | Hs.15396 |
| LOC196264 | 1570585_at | BC031223.1 | 196264 | Hs.15396 |
| LOC196264 | 227747_at | AK095399.1 | 196264 | Hs.15396 |
| LOC199675 | 235568_at | NM_174918 | 199675 | Hs.178703 |
| LOC201164 | 227037_at | NM_178836.2 | 201164 | Hs.31652 |
| LOC201164 | 244430_at | NM_178836 | 201164 | Hs.31652 |
| LOC205251 | 228614_at | NM_174925 | 205251 | Hs.128499 |
| LOC219854 | 226073_at | XM_933890.1 | 219854 | Hs.7626 |
| LOC221091 | 1556427_s_at | NM_203422.1 | 221091 | Hs.427449 |
| LOC221981 | 213894_at | XM_371877.4 | 221981 | Hs.120855 |
| LOC221981 | 214920_at | XM_371877.4 | 221981 | Hs.120855 |
| LOC221981 | 229159_at | XM_371877.4 | 221981 | Hs.120855 |
| LOC221981 | 230008_at | XM_371877.4 | 221981 | Hs.120855 |
| LOC253012 | 242601_at | NM_198151.1 | 253012 | Hs.443169 |
| LOC253981 | 1554714_at | BC039540 | 253981 | Hs.283378 |
| LOC253981 | 226430_at | AK025431.1 | 253981 | Hs.283378 |
| LOC255798 | 1559429_a_at | XM_942639.1 | 255798 | Hs.159017 |
| LOC257407 | 1556464_a_at | AF086098.1 | 257407 | Hs.526596 |
| LOC257407 | 213143_at | XM_939330.1 | 401034 | Hs.526596 |
| LOC257407 | 213148_at | XM_939330.1 | 401034 | Hs.526596 |
| LOC283241 | 224893_at | AK094227.1 | 25923 | Hs.356719 |
| LOC283464 | 226868_at | XM_290597.6 | 283464 | Hs.259347 |
| LOC283464 | 232021_at | XM_290597.6 | 283464 | Hs.259347 |
| LOC283537 | 214719_at | NM_181785 | 283537 | Hs.117167 |
| LOC284021 | 217513_at | XM_942446.1 | 284021 | Hs.406258 |
| LOC284274 | 1556288_at | BC037871 | 284274 | Hs.434164 |
| LOC284274 | 1564400_at | XM_927473.1 | 284274 | Hs.129564 |
| LOC285002 | 1568640_at | BC012788 | 285002 | Hs.131314 |
| LOC285002 | 235909_at | BF515082 | 285002 | Hs.131314 |
| LOC285002 | 240017_at | AW182817 | 285002 | Hs.131314 |
| LOC285016 | 238018_at | BC033490.1 | 285016 | Hs.355207 |
| LOC285878 | 230117_at | BC045679.1 | 285878 | Hs.335933 |
| LOC285878 | 236308_at | BC045679.1 | 285878 | Hs.335933 |
| LOC285929 | 239921_at | XM_209824.5 | 285929 | Hs.491104 |
| LOC285931 | 1562226_at | XM_371878.4 | 221806 | Hs.50802 |
| LOC286144 | 1562066_at | AK096398.1 | 286144 | Hs.558752 |
| LOC286144 | 224158_s_at | AF130048 | 286144 | Hs.440643 |
| LOC286144 | 225599_s_at | XM_926262.1 | 642649 | Hs.440643 |
| LOC286144 | 225600_at | XM_926262.1 | 642649 | Hs.440643 |
| LOC286144 | 225603_s_at | XM_926262.1 | 642649 | Hs.440643 |
| LOC339210 | 236863_at | XM_378687.3 | 339210 | Hs.158851 |
| LOC339366 | 242200_at | AA505848 | 339366 | Hs.372358 |
| LOC339903 | 228721_at | XM_934355.1 | 26172 | Hs.112822 |
| LOC339977 | 1568634_a_at | NM_001024611.1 | 339977 | Hs.87500 |
| LOC342551 | 232887_at | AU144437 | 342551 | Hs.283120 |
| LOC343700 | 234007_at | AI476463 | 343700 | Hs.272294 |
| LOC346356 | 229581_at | AI694118 | 346356 | Hs.42896 |
| LOC349151 | 231406_at | AW205664 | 349151 | Hs.440667 |
| LOC387601 | 1561093_at | AY517500.1 | 387601 | Hs.332119 |
| LOC387601 | 1561094_a_at | AK091990.1 | 387601 | Hs.332119 |
| LOC388335 | 229693_at | NM_001004313.1 | 388335 | Hs.462230 |
| LOC388397 | 221740_x_at | XM_373737.3 | 388397 | Hs.528473 |
| LOC388397 | 224686_x_at | XM_373737.3 | 388397 | Hs.528473 |
| LOC400258 | 1558420_at | NM_001008404.1 | 400258 | Hs.153827 |
| LOC400258 | 1558421_a_at | NM_001008404.1 | 400258 | Hs.153827 |
| LOC400451 | 221880_s_at | NM_207446.1 | 400451 | Hs.27373 |
| LOC400451 | 51158_at | NM_207446.1 | 400451 | Hs.27373 |
| LOC493869 | 227628_at | NM_001008397.1 | 493869 | Hs.559431 |
| LOC51136 | 221194_s_at | NM_016125 | 51136 | Hs.531701 |
| LOC51136 | 221195_at | NM_016125 | 51136 | Hs.531701 |
| LOC51136 | 227268_at | NM_016125 | 51136 | Hs.531701 |
| LOC55580 | 1562648_at | NM_017571 | 55580 | Hs.254122 |
| LOC613212 | 231391_at | XM_938437.1 | 613212 | Hs.66194 |
| LOC613212 | 236374_at | XM_928386.1 | 613212 | Hs.66194 |
| LOC89944 | 213713_s_at | NM_138342.2 | 89944 | Hs.436178 |
| LOC90342 | 233353_at | XM_937145.1 | 90342 | Hs.534025 |
| LOX | 213640_s_at | NM_002317.3 | 4015 | Hs.102267 |
| LOX | 215446_s_at | L16895 | 4015 | 102267 |
| LOX | 204298_s_at | NM_002317.3 | 4015 | Hs.102267 |
| LOXL1 | 203570_at | NM_005576.2 | 4016 | Hs.65436 |
| LOXL2 | 228808_s_at | NM_002318 | 4017 | Hs.83354 |
| LOXL2 | 1562263_at | NM_002318 | 4017 | Hs.83354 |
| LOXL2 | 202997_s_at | NM_002318.2 | 4017 | Hs.116479 |
| LOXL2 | 202998_s_at | NM_002318.2 | 4017 | Hs.116479 |
| LOXL2 | 202999_s_at | NM_002318 | 4017 | Hs.116479 |
| LOXL3 | 228253_at | NM_032603.2 | 84695 | Hs.469045 |
| LOXL4 | 227145_at | NM_032211.6 | 84171 | Hs.306814 |
| LOXL4 | 1570219_at | NM_032211 | 84171 | Hs.306814 |
| LPA | 207584_at | NM_005577.1 | 4018 | Hs.520120 |
| LPAL2 | 207762_at | NM_024492.2 | 80350 | Hs.439074 |
| LPAL2 | 210909_x_at | NM_145727.1 | 80350 | Hs.439074 |
| LPHN1 | 219145_at | NM_001008701.1 | 22859 | Hs.94229 |
| LPHN1 | 47560_at | NM_001008701.1 | 22859 | Hs.94229 |
| LPHN1 | 203488_at | NM_001008701.1 | 22859 | Hs.94229 |
| LPHN2 | 206953_s_at | NM_012302.2 | 23266 | Hs.24212 |
| LPHN3 | 236264_at | AK094703.1 | 23284 | Hs.28391 |
| LPHN3 | 242186_x_at | AK094703.1 | 23284 | Hs.28391 |
| LPHN3 | 209866_s_at | NM_015236.3 | 23284 | Hs.28391 |
| LPHN3 | 209867_s_at | NM_015236.3 | 23284 | Hs.28391 |
| LPL | 203548_s_at | NM_000237.1 | 4023 | Hs.180878 |
| LPL | 203549_s_at | NM_000237.1 | 4023 | Hs.180878 |
| LPO | 210682_at | NM_006151.1 | 4025 | Hs.234742 |
| LRAT | 220317_at | NM_004744.3 | 9227 | Hs.481028 |
| LRCH3 | 228119_at | AL137527.1 | 84859 | Hs.518414 |
| LRCH3 | 1553668_at | NM_032773.2 | 84859 | Hs.518414 |
| LRCH3 | 1559490_at | NM_032773 | 84859 | Hs.289038 |
| LRFN1 | 232486_at | XM_940573.1 | 57622 | Hs.97860 |
| LRFN2 | 232697_at | NM_020737.1 | 57497 | Hs.250015 |
| LRFN3 | 219346_at | NM_024509.1 | 79414 | Hs.143792 |
| LRFN4 | 219491_at | NM_024036.3 | 78999 | Hs.209979 |
| LRG1 | 228648_at | NM_052972.2 | 116844 | Hs.515079 |
| LRIG1 | 238339_x_at | NM_015541.2 | 26018 | Hs.518055 |
| LRIG1 | 240140_s_at | NM_015541 | 26018 | Hs.518055 |
| LRIG1 | 211596_s_at | NM_015541.2 | 26018 | Hs.518055 |
| LRIG2 | 242164_s_at | NM_014813.1 | 9860 | Hs.448972 |
| LRIG2 | 242165_at | NM_014813 | 9860 | Hs.309305 |
| LRIG2 | 205953_at | NM_014813.1 | 9860 | Hs.448972 |
| LRIG3 | 226908_at | NM_153377.3 | 121227 | Hs.253736 |
| LRP1 | 1555353_at | NM_002332 | 4035 | Hs.162757 |
| LRP1 | 1569042_at | NM_002332 | 4035 | Hs.162757 |
| LRP1 | 200784_s_at | NM_002332.1 | 4035 | Hs.162757 |
| LRP1 | 200785_s_at | NM_002332.1 | 4035 | Hs.162757 |
| LRP10 | 227252_at | NM_014045 | 26020 | Hs.28368 |
| LRP10 | 231861_at | NM_014045 | 26020 | Hs.525232 |
| LRP10 | 201412_at | NM_014045.3 | 26020 | Hs.525232 |
| LRP11 | 225060_at | NM_032832.3 | 84918 | Hs.408355 |
| LRP12 | 219631_at | NM_013437.2 | 29967 | Hs.301974 |
| LRP12 | 220253_s_at | NM_013437.2 | 29967 | Hs.301974 |
| LRP12 | 220254_at | NM_013437.2 | 29967 | Hs.301974 |
| LRP1B | 219643_at | NM_018557.2 | 53353 | Hs.470117 |
| LRP1B | 234184_at | AK025148 | 53353 | 470117 |
| LRP1B | 234209_at | AK025148 | 53353 | 470117 |
| LRP2 | 230863_at | NM_004525 | 4036 | Hs.252938 |
| LRP2 | 205710_at | NM_004525.1 | 4036 | Hs.470538 |
| LRP3 | 228525_at | NM_002333 | 4037 | Hs.143641 |
| LRP3 | 204381_at | NM_002333.1 | 4037 | Hs.515340 |
| LRP4 | 212850_s_at | NM_002334.1 | 4038 | Hs.4930 |
| LRP5 | 229591_at | NM_002335 | 4041 | Hs.6347 |
| LRP5 | 209468_at | NM_002335.1 | 4041 | Hs.6347 |
| LRP6 | 225745_at | NM_002336 | 4040 | Hs.549194 |
| LRP6 | 34697_at | NM_002336.1 | 4040 | Hs.549194 |
| LRP6 | 205606_at | NM_002336.1 | 4040 | Hs.549194 |
| LRP8 | 1566902_at | NM_001018054 | 7804 | 576154 |
| LRP8 | 1566903_at | NM_001018054 | 7804 | 576154 |
| LRP8 | 205282_at | NM_017522.3 | 7804 | Hs.444637 |
| LRP8 | 208433_s_at | NM_001018054.1 | 7804 | Hs.444637 |
| LRPAP1 | 201186_at | NM_002337.1 | 4043 | Hs.533136 |
| LRRC15 | 213909_at | NM_130830.2 | 131578 | Hs.288467 |
| LRRC15 | 1552960_at | NM_130830.2 | 131578 | Hs.288467 |
| LRRC19 | 220376_at | NM_022901.1 | 64922 | Hs.128071 |
| LRRC25 | 1559502_s_at | NM_145256.2 | 126364 | Hs.332156 |
| LRRC3 | 221017_s_at | NM_030891.3 | 81543 | Hs.326579 |
| LRRC32 | 203835_at | NM_005512.1 | 2615 | Hs.151641 |
| LRRC33 | 235359_at | NM_198565.1 | 375387 | Hs.478815 |
| LRRC37A3 | 220219_s_at | NM_199340.2 | 374819 | Hs.559258 |
| LRRC37A3 | 229962_at | NM_199340.2 | 374819 | Hs.559258 |
| LRRC37A3 | 243899_at | NM_199340.2 | 374819 | Hs.559258 |
| LRRC37B | 229231_at | NM_052888.2 | 114659 | Hs.514071 |
| LRRC38 | 1558375_at | XM_059074.4 | 126755 | Hs.459542 |
| LRRC3B | 229085_at | NM_052953.2 | 116135 | Hs.517868 |
| LRRC4 | 223552_at | NM_022143.3 | 64101 | Hs.108681 |
| LRRC4B | 239320_at | XM_292778.4 | 94030 | Hs.120873 |
| LRRC4C | 232226_at | NM_020929.1 | 57689 | Hs.135736 |
| LRRC4C | 241585_at | AI939418 | 57689 | 135736 |
| LRRC52 | 1556484_at | NM_001005214.1 | 440699 | Hs.120487 |
| LRRC54 | 218245_at | NM_015516.3 | 25987 | Hs.8361 |
| LRRC7 | 233499_at | CR749629.1 | 57554 | Hs.479658 |
| LRRC7 | 237602_at | H07100 |  | Hs.13471 |
| LRRC7 | 1552666_a_at | NM_020794.1 | 57554 | Hs.479658 |
| LRRC8A | 224624_at | NM_019594.2 | 56262 | Hs.173484 |
| LRRC8A | 233487_s_at | NM_019594.2 | 56262 | Hs.173484 |
| LRRC8B | 242038_at | BC053565.1 | 23507 | Hs.482017 |
| LRRC8C | 223533_at | NM_032270.2 | 84230 | Hs.412836 |
| LRRC8D | 218684_at | NM_018103.3 | 55144 | Hs.482087 |
| LRRC8E | 220174_at | NM_025061.3 | 80131 | Hs.501511 |
| LRRC8E | 239433_at | NM_025061.3 | 80131 | Hs.501511 |
| LRRN1 | 226884_at | NM_020873.3 | 57633 | Hs.163244 |
| LRRN3 | 209840_s_at | NM_018334.3 | 54674 | Hs.3781 |
| LRRN3 | 209841_s_at | NM_018334.3 | 54674 | Hs.3781 |
| LRRN5 | 205154_at | NM_006338.2 | 10446 | Hs.26312 |
| LRRN5 | 216167_at | AK024867.1 | 10446 | Hs.26312 |
| LRRTM2 | 206408_at | NM_015564.1 | 26045 | Hs.445981 |
| LRRTM4 | 220345_at | NM_024993.3 | 80059 | Hs.285782 |
| LRSAM1 | 227675_at | NM_001005373.1 | 90678 | Hs.495188 |
| LRSAM1 | 235449_at | NM_001005373.1 | 90678 | Hs.495188 |
| LRTM1 | 221612_at | NM_020678.2 | 57408 | Hs.558524 |
| LRTM1 | 91580_at | NM_020678.2 | 57408 | Hs.558524 |
| LSAMP | 214460_at | NM_002338.2 | 4045 | Hs.26479 |
| LSR | 208190_s_at | NM_205834.2 | 51599 | Hs.466507 |
| LST1 | 214181_x_at | NM_007161.2 | 7940 | Hs.436066 |
| LST1 | 214574_x_at | NM_205837.1 | 7940 | Hs.436066 |
| LST1 | 215633_x_at | NM_007161.2 | 7940 | Hs.436066 |
| LST1 | 210629_x_at | NM_205839.1 | 7940 | Hs.436066 |
| LST1 | 211581_x_at | NM_205837.1 | 7940 | Hs.436066 |
| LST1 | 211582_x_at | NM_205837.1 | 7940 | Hs.436066 |
| LTA | 206975_at | NM_000595.2 | 4049 | Hs.36 |
| LTB | 1559754_at | NM_002341 | 4050 | Hs.376208 |
| LTB | 207339_s_at | NM_009588.1 | 4050 | Hs.376208 |
| LTB4R | 216388_s_at | NM_181657.1 | 1241 | Hs.525256 |
| LTB4R | 236172_at | NM_000752 | 1241 | Hs.445013 |
| LTB4R | 210128_s_at | NM_181657.1 | 1241 | Hs.525256 |
| LTB4R2 | 220130_x_at | NM_019839.1 | 56413 | Hs.130685 |
| LTBP1 | 202728_s_at | NM_206943.1 | 4052 | Hs.49787 |
| LTBP1 | 202729_s_at | NM_000627.2 | 4052 | Hs.49787 |
| LTBP2 | 223690_at | NM_000428.2 | 4053 | Hs.512776 |
| LTBP2 | 204682_at | NM_000428.2 | 4053 | Hs.512776 |
| LTBP3 | 219922_s_at | NM_021070.2 | 4054 | Hs.289019 |
| LTBP3 | 227308_x_at | NM_021070 | 4054 | Hs.289019 |
| LTBP4 | 227989_at | NM_003573 | 8425 | Hs.85087 |
| LTBP4 | 204442_x_at | NM_003573 | 8425 | Hs.466766 |
| LTBP4 | 210628_x_at | NM_003573 | 8425 | Hs.466766 |
| LTBP4 | 213176_s_at | NM_003573 | 8425 | Hs.466766 |
| LTBR | 232819_s_at | NM_002342 | 4055 | Hs.1116 |
| LTBR | 243400_x_at | NM_002342 | 4055 | Hs.1116 |
| LTBR | 1559458_at | NM_002342 | 4055 | Hs.1116 |
| LTBR | 203005_at | NM_002342.1 | 4055 | Hs.1116 |
| LTF | 202018_s_at | NM_002343.2 | 4057 | Hs.518419 |
| LTK | 217184_s_at | NM_002344.3 | 4058 | Hs.434481 |
| LTK | 207106_s_at | NM_002344.3 | 4058 | Hs.434481 |
| LU | 203009_at | NM_005581.3 | 4059 | Hs.155048 |
| LU | 40093_at | NM_005581.3 | 4059 | Hs.155048 |
| LUZP2 | 215323_at | NM_001009909.2 | 338645 | Hs.144138 |
| LUZP2 | 216443_at | AK024615 | 338645 | 144138 |
| LUZP2 | 216744_at | AK024615 | 338645 | 144138 |
| LY6D | 206276_at | NM_003695.2 | 8581 | Hs.415762 |
| LY6E | 202145_at | NM_002346.1 | 4061 | Hs.521903 |
| LY6G5C | 219860_at | NM_001002848.1 | 80741 | Hs.25738 |
| LY6G6C | 207114_at | NM_025261.1 | 80740 | Hs.241586 |
| LY6G6D | 207457_s_at | NM_021246.2 | 58530 | Hs.408316 |
| LY6H | 206773_at | NM_002347.2 | 4062 | Hs.159590 |
| LY6K | 223687_s_at | NM_017527.2 | 54742 | Hs.69517 |
| LY6K | 223688_s_at | NM_017527.2 | 54742 | Hs.69517 |
| LY75 | 205668_at | NM_002349.1 | 4065 | Hs.153563 |
| LY86 | 205859_at | NM_004271.3 | 9450 | Hs.170081 |
| LY9 | 215967_s_at | NM_002348.2 | 4063 | Hs.403857 |
| LY9 | 231124_x_at | NM_001033667.1 | 4063 | Hs.403857 |
| LY9 | 210370_s_at | NM_002348 | 4063 | Hs.403857 |
| LY96 | 206584_at | NM_015364.2 | 23643 | Hs.69328 |
| LYG2 | 1554398_at | NM_175735.3 | 254773 | Hs.436468 |
| LYN | 202625_at | NM_002350 | 4067 | Hs.491767 |
| LYN | 202626_s_at | NM_002350.1 | 4067 | Hs.491767 |
| LYN | 210754_s_at | NM_002350.1 | 4067 | Hs.491767 |
| LYNX1 | 226305_at | NM_177457.3 | 66004 | Hs.158665 |
| LYNX1 | 244224_x_at | NM_023946 | 66004 | Hs.158665 |
| LYNX1 | 1554179_s_at | NM_023946.1 | 66004 | Hs.158665 |
| LYNX1 | 1559388_a_at | NM_177457.3 | 66004 | Hs.158665 |
| LYPD1 | 212909_at | NM_144586.3 | 116372 | Hs.432395 |
| LYPD3 | 204952_at | NM_014400.2 | 27076 | Hs.377028 |
| LYPD4 | 231266_at | NM_173506.3 | 147719 | Hs.98660 |
| LYPD5 | 236039_at | NM_182573.1 | 284348 | Hs.44289 |
| LYPD6 | 227764_at | NM_194317.2 | 130574 | Hs.21929 |
| LYPD6 | 239028_at | NM_194317.2 | 130574 | Hs.21929 |
| LYSMD3 | 226321_at | NM_198273.1 | 116068 | Hs.136235 |
| LYSMD4 | 228954_at | NM_152449.2 | 145748 | Hs.268675 |
| LYZ | 213975_s_at | NM_000239.1 | 4069 | Hs.524579 |
| LYZ | 1555745_a_at | NM_000239.1 | 4069 | Hs.524579 |
| LYZL1 | 230514_s_at | NM_032517.4 | 84569 | Hs.558572 |
| LYZL4 | 231306_at | NM_144634.2 | 131375 | Hs.234895 |
| LYZL6 | 207343_at | NM_020426.1 | 57151 | Hs.97477 |
| LZTS1 | 219042_at | NM_021020.1 | 11178 | Hs.521432 |
| LZTS1 | 221719_s_at | NM_021020.1 | 11178 | Hs.521432 |
| LZTS1 | 221721_s_at | NM_021020.1 | 11178 | Hs.521432 |
| LZTS1 | 221722_x_at | NM_021020.1 | 11178 | Hs.521432 |
| LZTS1 | 222107_x_at | NM_021020.1 | 11178 | Hs.521432 |
| LZTS1 | 47550_at | NM_021020.1 | 11178 | Hs.521432 |
| LZTS1 | 1569159_at | NM_021020 | 11178 | Hs.93605 |
| M6PR | 200900_s_at | NM_002355.2 | 4074 | Hs.134084 |
| M6PR | 200901_s_at | NM_002355.2 | 4074 | Hs.134084 |
| M6PRBP1 | 202122_s_at | NM_005817.3 | 10226 | Hs.140452 |
| MAC30 | 212279_at | NM_014573 | 27346 | Hs.199695 |
| MAC30 | 212281_s_at | NM_014573 | 27346 | Hs.199695 |
| MAC30 | 212282_at | NM_014573 | 27346 | Hs.199695 |
| MADCAM1 | 208037_s_at | NM_007164 | 8174 | Hs.102598 |
| MADD | 38398_at | NM_003682.2 | 8567 | Hs.82548 |
| MADD | 210252_s_at | NM_003682.2 | 8567 | Hs.82548 |
| MAEA | 207922_s_at | NM_001017405.1 | 10296 | Hs.139896 |
| MAG | 216617_s_at | NM_002361.2 | 4099 | Hs.515354 |
| MAG | 217102_at | AF041410.1 | 27307 | Hs.348346 |
| MAG | 217105_at | AF041410 | 27307 | Hs.348346 |
| MAGEA1 | 207325_x_at | NM_004988.3 | 4100 | Hs.72879 |
| MAGED1 | 209014_at | NM_001005332.1 | 9500 | Hs.5258 |
| MAL | 236973_at | NM_002371 | 4118 | Hs.80395 |
| MAL | 204777_s_at | NM_022439.1 | 4118 | Hs.80395 |
| MAL2 | 224650_at | NM_052886 | 114569 | Hs.201083 |
| MALL | 209373_at | NM_005434.3 | 7851 | Hs.185055 |
| MALT1 | 208309_s_at | NM_006785.2 | 10892 | Hs.180566 |
| MALT1 | 210017_at | NM_006785.2 | 10892 | Hs.180566 |
| MALT1 | 210018_x_at | NM_006785.2 | 10892 | Hs.180566 |
| MAMDC1 | 1570114_at | BC028193.1 | 161357 | Hs.436380 |
| MAMDC1 | 239935_at | NM_182830.2 | 161357 | Hs.436380 |
| MAMDC2 | 228885_at | NM_153267.3 | 256691 | Hs.127386 |
| MAMDC4 | 229473_at | NM_206920.1 | 158056 | Hs.376780 |
| MAN2B2 | 214703_s_at | NM_015274.1 | 23324 | Hs.188464 |
| MANBAL | 224689_at | NM_001003897.1 | 63905 | Hs.6126 |
| MAP2K2 | 213490_s_at | NM_030662.2 | 5605 | Hs.465627 |
| MAP2K2 | 237878_at | NM_030662 | 5605 | Hs.366546 |
| MAP2K2 | 202424_at | NM_030662.2 | 5605 | Hs.465627 |
| MARCO | 205819_at | NM_006770.3 | 8685 | Hs.67726 |
| MARVELD1 | 223095_at | NM_031484.2 | 83742 | Hs.209614 |
| MARVELD1 | 234917_at | AK027093.1 | 83742 | Hs.209614 |
| MARVELD2 | 235955_at | NM_001038603.1 | 153562 | Hs.444195 |
| MARVELD3 | 233634_at | BC005052.1 | 91862 | Hs.513706 |
| MARVELD3 | 239350_at | NM_052858.3 | 91862 | Hs.513706 |
| MAS1 | 208210_at | NM_002377.2 | 4142 | Hs.99900 |
| MAS1L | 234820_at | NM_052967.1 | 116511 | Hs.533297 |
| MASP1 | 213749_at | NM_001879 | 5648 | Hs.89983 |
| MASP1 | 232224_at | NM_139125.2 | 5648 | Hs.89983 |
| MASP1 | 235770_at | NM_001879 | 5648 | Hs.89983 |
| MASP1 | 206449_s_at | NM_001879.4 | 5648 | Hs.89983 |
| MASP1 | 210680_s_at | NM_001879.4 | 5648 | Hs.89983 |
| MASP2 | 216968_at | NM_006610 | 10747 | Hs.119983 |
| MASP2 | 229111_at | NM_006610 | 10747 | Hs.119983 |
| MASP2 | 207041_at | NM_006610.2 | 10747 | Hs.119983 |
| MASP2 | 210798_x_at | NM_139208.1 | 10747 | Hs.119983 |
| MASS1 | 215396_at | NM_032119 | 84059 | Hs.153692 |
| MASS1 | 223582_at | NM_032119.1 | 84059 | Hs.482811 |
| MASS1 | 224275_at | NM_032119 | 84059 | Hs.482811 |
| MASS1 | 234871_at | NM_032119.1 | 84059 | Hs.482811 |
| MASS1 | 240631_at | NM_032119 | 84059 | Hs.153692 |
| MATN1 | 206904_at | NM_002379 | 4146 | Hs.150366 |
| MATN1 | 206905_s_at | NM_002379.2 | 4146 | Hs.150366 |
| MATN2 | 202350_s_at | NM_002380.3 | 4147 | Hs.189445 |
| MATN3 | 206091_at | NM_002381.3 | 4148 | Hs.6985 |
| MATN4 | 207123_s_at | NM_030590.1 | 8785 | Hs.278489 |
| MBL2 | 207256_at | NM_000242.1 | 4153 | Hs.499674 |
| MBP | 225407_at | NM_001025100.1 | 4155 | Hs.551713 |
| MBP | 225408_at | NM_001025100.1 | 4155 | Hs.551713 |
| MBP | 228938_at | NM_002385 | 4155 | Hs.408543 |
| MBP | 236324_at | NM_002385 | 4155 | Hs.551713 |
| MBP | 1554544_a_at | NM_001025100.1 | 4155 | Hs.551713 |
| MBP | 207323_s_at | NM_001025081.1 | 4155 | Hs.551713 |
| MBP | 209072_at | NM_001025081.1 | 4155 | Hs.551713 |
| MBP | 210136_at | NM_001025100.1 | 4155 | Hs.551713 |
| MBTPS2 | 226760_at | AK056736.1 | 203411 | Hs.443490 |
| MBTPS2 | 1554604_at | NM_015884 | 51360 | Hs.443490 |
| MBTPS2 | 206473_at | NM_015884.1 | 51360 | Hs.443490 |
| MC1R | 205458_at | NM_002386.2 | 4157 | Hs.513829 |
| MC2R | 217425_at | NM_000529 | 4158 | Hs.248144 |
| MC2R | 217434_at | NM_000529.2 | 4158 | Hs.248144 |
| MC2R | 208568_at | NM_000529.2 | 4158 | Hs.248144 |
| MC3R | 221442_at | NM_019888.2 | 4159 | Hs.248018 |
| MC4R | 221467_at | NM_005912.1 | 4160 | Hs.532833 |
| MC5R | 208565_at | NM_005913.1 | 4161 | Hs.248145 |
| MCAM | 1568191_at | NM_006500 | 4162 | Hs.211579 |
| MCAM | 209086_x_at | NM_006500.2 | 4162 | Hs.511397 |
| MCAM | 209087_x_at | NM_006500.2 | 4162 | Hs.511397 |
| MCAM | 210869_s_at | NM_006500.2 | 4162 | Hs.511397 |
| MCAM | 211042_x_at | BC006329 | 4162 | 599039 |
| MCAM | 211340_s_at | NM_006500.2 | 4162 | Hs.511397 |
| MCOLN1 | 219952_s_at | NM_020533.1 | 57192 | Hs.546413 |
| MCOLN2 | 230110_at | NM_153259.2 | 255231 | Hs.459526 |
| MCOLN2 | 1555465_at | NM_153259.2 | 255231 | Hs.459526 |
| MCOLN3 | 220484_at | NM_018298.9 | 55283 | Hs.535239 |
| MCOLN3 | 229797_at | NM_018298.9 | 55283 | Hs.535239 |
| MCOLN3 | 242308_at | NM_018298 | 55283 | Hs.49344 |
| MCOLN3 | 1557292_a_at | NM_018298 | 55283 | Hs.535239 |
| MCP | 207549_x_at | NM_172350.1 | 4179 | Hs.510402 |
| MCP | 208783_s_at | NM_002389.3 | 4179 | Hs.510402 |
| MCP | 211574_s_at | NM_172354.1 | 4179 | Hs.510402 |
| MCP | 237126_at | NM_002389 | 4179 | Hs.83532 |
| MCTP1 | 220122_at | AK025997.1 | 79772 | Hs.127412 |
| MCTP1 | 1554730_at | BC030005.2 | 79772 | Hs.127412 |
| MCTP2 | 220603_s_at | AK002037.1 | 55784 | Hs.33368 |
| MCTP2 | 1554833_at | BC111024.1 | 55784 | Hs.33368 |
| MDAC1 | 1552594_at | NM_139172.1 | 147744 | Hs.160436 |
| MDGA1 | 232237_at | NM_153487.2 | 266727 | Hs.437993 |
| MDGA1 | 238543_x_at | NM_153487.2 | 266727 | Hs.437993 |
| MDGA1 | 242757_at | NM_153487.2 | 266727 | Hs.437993 |
| MDK | 242203_at | NM_002391 | 4192 | Hs.82045 |
| MDK | 209035_at | NM_001012333.1 | 4192 | Hs.82045 |
| MEGF10 | 1555343_at | NM_032446 | 84466 | Hs.438709 |
| MEGF11 | 1552439_s_at | NM_032445.1 | 84465 | Hs.438250 |
| MEGF11 | 1560334_at | NM_032445 | 84465 | Hs.438250 |
| MEGF11 | 1569879_a_at | NM_032445.1 | 84465 | Hs.438250 |
| MELL1 | 1552930_at | NM_033467.2 | 79258 | Hs.462033 |
| MEP1A | 206000_at | NM_005588.1 | 4224 | Hs.179704 |
| MEP1B | 207251_at | NM_005925.1 | 4225 | Hs.194777 |
| MEPE | 221150_at | NM_020203.1 | 56955 | Hs.189587 |
| MERTK | 233079_at | AK026802.1 | 10461 | Hs.306178 |
| MERTK | 206028_s_at | NM_006343.2 | 10461 | Hs.306178 |
| MERTK | 211913_s_at | NM_006343.2 | 10461 | Hs.306178 |
| MET | 213807_x_at | NM_000245 | 4233 | Hs.419124 |
| MET | 213816_s_at | NM_000245.2 | 4233 | Hs.132966 |
| MET | 203510_at | NM_000245.2 | 4233 | Hs.132966 |
| MET | 211599_x_at | NM_000245.2 | 4233 | Hs.132966 |
| METRN | 219051_x_at | NM_024042.2 | 79006 | Hs.533772 |
| METRN | 232269_x_at | NM_024042.2 | 79006 | Hs.533772 |
| METRNL | 225955_at | NM_001004431.1 | 284207 | Hs.514615 |
| MFAP1 | 203406_at | NM_005926.2 | 4236 | Hs.61418 |
| MFAP2 | 203417_at | NM_002403.2 | 4237 | Hs.389137 |
| MFAP3 | 214588_s_at | NM_005927 | 4238 | Hs.432818 |
| MFAP3 | 1552312_a_at | NM_005927.3 | 4238 | Hs.432818 |
| MFAP3 | 213123_at | NM_005927 | 4238 | Hs.432818 |
| MFAP3L | 205442_at | AB014526.1 | 9848 | Hs.178121 |
| MFAP3L | 210492_at | BC001279.1 | 9848 | Hs.178121 |
| MFAP3L | 210493_s_at | NM_021647.5 | 9848 | Hs.178121 |
| MFAP3L | 210843_s_at | NM_001009554.1 | 9848 | Hs.178121 |
| MFAP4 | 212713_at | NM_002404.1 | 4239 | Hs.296049 |
| MFAP5 | 213764_s_at | NM_003480.2 | 8076 | Hs.512842 |
| MFAP5 | 213765_at | NM_003480.2 | 8076 | Hs.512842 |
| MFAP5 | 209758_s_at | NM_003480.2 | 8076 | Hs.512842 |
| MFGE8 | 210605_s_at | NM_005928.1 | 4240 | Hs.3745 |
| MFI2 | 220043_s_at | NM_005929.3 | 4241 | Hs.184727 |
| MFI2 | 223723_at | NM_033316.2 | 4241 | Hs.184727 |
| MFI2 | 239521_at | NM_005929 | 4241 | Hs.252855 |
| MFI2 | 1556538_at | NM_005929 | 4241 | Hs.252855 |
| MFNG | 204152_s_at | NM_002405.2 | 4242 | Hs.517603 |
| MFNG | 204153_s_at | NM_002405.2 | 4242 | Hs.517603 |
| MFRP | 224286_at | NM_031433.1 | 83552 | Hs.512769 |
| MFSD1 | 218109_s_at | NM_022736.1 | 64747 | Hs.58663 |
| MFSD11 | 221192_x_at | NM_024311.2 | 79157 | Hs.464166 |
| MFSD11 | 223242_s_at | NM_024311.2 | 79157 | Hs.464166 |
| MFSD2 | 225316_at | NM_032793.2 | 84879 | Hs.75668 |
| MFSD2 | 1553386_at | AK027396.1 | 84879 | Hs.75668 |
| MFSD3 | 227296_at | NM_138431.1 | 113655 | Hs.7678 |
| MFSD4 | 238862_at | NM_181644.2 | 148808 | Hs.497518 |
| MFSD4 | 242372_s_at | NM_181644.2 | 148808 | Hs.497518 |
| MFSD5 | 212861_at | NM_032889.2 | 84975 | Hs.19210 |
| MFSD7 | 214269_at | NM_032219.2 | 84179 | Hs.410970 |
| MFSD9 | 224483_s_at | NM_032718.2 | 84804 | Hs.98798 |
| MGAM | 206522_at | NM_004668.1 | 8972 | Hs.122785 |
| MGC10731 | 244834_at | NM_030907 | 79363 | Hs.27017 |
| MGC10744 | 235490_at | NM_032354 | 84314 | Hs.413219 |
| MGC10946 | 229778_at | NM_030572 | 80763 | Hs.130692 |
| MGC13379 | 223305_at | NM_016499.2 | 51259 | Hs.26745 |
| MGC16824 | 1558557_at | NM_020314 | 57020 | Hs.546412 |
| MGC16824 | 203173_s_at | NM_020314.3 | 57020 | Hs.546412 |
| MGC20579 | 238226_at | NM_182614 | 348013 | Hs.208470 |
| MGC23909 | 224702_at | NM_174909 | 153339 | Hs.355606 |
| MGC24039 | 215058_at | NM_144973.2 | 160518 | Hs.118166 |
| MGC24039 | 228551_at | NM_144973 | 160518 | Hs.118166 |
| MGC24039 | 236415_at | NM_144973 | 160518 | Hs.118166 |
| MGC24039 | 238917_s_at | NM_144973 | 160518 | Hs.118166 |
| MGC24039 | 243613_at | NM_144973.2 | 160518 | Hs.118166 |
| MGC26963 | 227038_at | NM_152621 | 166929 | Hs.48343 |
| MGC26963 | 243141_at | NM_152621 | 166929 | Hs.48343 |
| MGC3035 | 222129_at | NM_024293 | 79137 | Hs.22412 |
| MGC33214 | 227861_at | NM_153354 | 153396 | Hs.379972 |
| MGC33214 | 236227_at | NM_153354 | 153396 | Hs.379972 |
| MGC33214 | 238783_at | NM_153354 | 153396 | Hs.379972 |
| MGC34821 | 1553923_at | NM_173586.1 | 283238 | Hs.375139 |
| MGC35295 | 1564194_a_at | NM_152717.1 | 219995 | Hs.207465 |
| MGC40499 | 217700_at | NM_152755 | 245812 | Hs.369867 |
| MGC40499 | 227313_at | NM_152755.1 | 245812 | Hs.369867 |
| MGC4172 | 218756_s_at | NM_024308 | 79154 | Hs.462859 |
| MGC52498 | 1555379_at | NM_182621.1 | 348378 | Hs.424589 |
| MGC52498 | 237033_at | NM_182621 | 348378 | Hs.424589 |
| MGP | 238481_at | NM_000900 | 4256 | Hs.365706 |
| MGP | 202291_s_at | NM_000900.2 | 4256 | Hs.365706 |
| MIA | 206560_s_at | NM_006533.1 | 8190 | Hs.279651 |
| MIA2 | 221177_at | NM_054024.3 | 117153 | Hs.287694 |
| MIA2 | 1553944_at | NM_054024.3 | 117153 | Hs.287694 |
| MICA | 205904_at | NM_000247.1 | 4276 | Hs.549053 |
| MICA | 205905_s_at | NM_000247.1 | 4276 | Hs.549053 |
| MICB | 206247_at | NM_005931.2 | 4277 | Hs.211580 |
| MIF | 217871_s_at | NM_002415.1 | 4282 | Hs.407995 |
| MIP | 220863_at | NM_012064.2 | 4284 | Hs.558356 |
| MIR16 | 202593_s_at | NM_016641.3 | 51573 | Hs.512607 |
| MIR16 | 226214_at | NM_016641.3 | 51573 | Hs.512607 |
| MLANA | 206426_at | NM_005511.1 | 2315 | Hs.154069 |
| MLANA | 206427_s_at | NM_005511.1 | 2315 | Hs.154069 |
| MLC1 | 213395_at | NM_015166.3 | 23209 | Hs.517729 |
| MLN | 207473_at | NM_001040109.1 | 4295 | Hs.2813 |
| MLNR | 221365_at | NM_001507.1 | 2862 | Hs.248126 |
| MMD | 244523_at | NM_012329 | 23531 | Hs.79889 |
| MMD | 203414_at | NM_012329.2 | 23531 | Hs.463483 |
| MMD2 | 230826_at | NM_198403.2 | 221938 | Hs.558694 |
| MMD2 | 1569998_at | BC024012.1 | 221938 | Hs.558694 |
| MME | 203434_s_at | NM_007288.1 | 4311 | Hs.307734 |
| MME | 203435_s_at | NM_000902.2 | 4311 | Hs.307734 |
| MMP1 | 204475_at | NM_002421.2 | 4312 | Hs.83169 |
| MMP10 | 205680_at | NM_002425.1 | 4319 | Hs.2258 |
| MMP11 | 235908_at | NM_005940 | 4320 | Hs.143751 |
| MMP11 | 203876_s_at | NM_005940.3 | 4320 | Hs.143751 |
| MMP11 | 203878_s_at | NM_005940.3 | 4320 | Hs.143751 |
| MMP12 | 204580_at | NM_002426.2 | 4321 | Hs.1695 |
| MMP13 | 205959_at | NM_002427.2 | 4322 | Hs.2936 |
| MMP14 | 217279_x_at | NM_004995 | 4323 | Hs.2399 |
| MMP14 | 160020_at | NM_004995.2 | 4323 | Hs.2399 |
| MMP14 | 202827_s_at | NM_004995.2 | 4323 | Hs.2399 |
| MMP14 | 202828_s_at | NM_004995.2 | 4323 | Hs.2399 |
| MMP15 | 243883_at | NM_002428 | 4324 | Hs.80343 |
| MMP15 | 203365_s_at | NM_002428.2 | 4324 | Hs.80343 |
| MMP16 | 207012_at | NM_005941.3 | 4325 | Hs.546267 |
| MMP16 | 207013_s_at | NM_005941.3 | 4325 | Hs.546267 |
| MMP16 | 208166_at | NM_022564.2 | 4325 | Hs.546267 |
| MMP16 | 208167_s_at | NM_005941.3 | 4325 | Hs.546267 |
| MMP17 | 206234_s_at | NM_016155.3 | 4326 | Hs.159581 |
| MMP19 | 204574_s_at | NM_002429.4 | 4327 | Hs.154057 |
| MMP19 | 204575_s_at | NM_001032360.1 | 4327 | Hs.154057 |
| MMP20 | 207599_at | NM_004771.3 | 9313 | Hs.302383 |
| MMP21 | 1552592_at | NM_147191.1 | 118856 | Hs.314141 |
| MMP23B | 207118_s_at | NM_006983.1 | 8510 | Hs.555018 |
| MMP24 | 221953_s_at | NM_006690 | 10893 | Hs.212581 |
| MMP24 | 225860_at | NM_006690 | 10893 | Hs.212581 |
| MMP24 | 228016_s_at | NM_006690 | 10893 | Hs.212581 |
| MMP24 | 49679_s_at | NM_006690 | 10893 | Hs.212581 |
| MMP24 | 78047_s_at | NM_006690 | 10893 | Hs.212581 |
| MMP24 | 208387_s_at | NM_006690.3 | 10893 | Hs.555914 |
| MMP24 | 213171_s_at | NM_006690.3 | 10893 | Hs.555914 |
| MMP25 | 207890_s_at | NM_022718.2 | 64386 | Hs.312579 |
| MMP26 | 220541_at | NM_021801.3 | 56547 | Hs.204732 |
| MMP27 | 220783_at | NM_022122.2 | 64066 | Hs.534479 |
| MMP28 | 219909_at | NM_024302.3 | 79148 | Hs.380710 |
| MMP28 | 222937_s_at | NM_024302.3 | 79148 | Hs.380710 |
| MMP28 | 224207_x_at | NM_024302.3 | 79148 | Hs.380710 |
| MMP28 | 239272_at | NM_001032278.1 | 79148 | Hs.380710 |
| MMP28 | 239273_s_at | NM_001032278.1 | 79148 | Hs.380710 |
| MMP3 | 205828_at | NM_002422.3 | 4314 | Hs.375129 |
| MMP7 | 204259_at | NM_002423.3 | 4316 | Hs.2256 |
| MMP8 | 207329_at | NM_002424.1 | 4317 | Hs.161839 |
| MMP9 | 203936_s_at | NM_004994.2 | 4318 | Hs.297413 |
| MMRN1 | 205612_at | NM_007351.2 | 22915 | Hs.268107 |
| MMRN2 | 219091_s_at | NM_024756.1 | 79812 | Hs.524479 |
| MMRN2 | 222822_s_at | NM_024756.1 | 79812 | Hs.524479 |
| MOG | 214650_x_at | NM_001008228.1 | 4340 | Hs.141308 |
| MOG | 1555807_a_at | NM_206813.2 | 4340 | Hs.141308 |
| MOG | 205989_s_at | NM_001008228.1 | 4340 | Hs.141308 |
| MOG | 211836_s_at | NM_002433.3 | 4340 | Hs.141308 |
| MOSPD1 | 218853_s_at | NM_019556.1 | 56180 | Hs.378505 |
| MOSPD1 | 1557455_s_at | NM_019556.1 | 56180 | Hs.378505 |
| MOSPD2 | 221895_at | NM_152581.1 | 158747 | Hs.190043 |
| MOSPD2 | 64883_at | NM_152581 | 158747 | Hs.190043 |
| MOSPD3 | 219070_s_at | NM_001040097.1 | 64598 | Hs.521086 |
| MPEG1 | 226818_at | XM_166227.6 | 219972 | Hs.523696 |
| MPEG1 | 226841_at | XM_166227.6 | 219972 | Hs.523696 |
| MPL | 216825_s_at | NM_005373.1 | 4352 | Hs.82906 |
| MPL | 207550_at | NM_005373.1 | 4352 | Hs.82906 |
| MPL | 211903_s_at | NM_005373.1 | 4352 | Hs.82906 |
| MPP2 | 213270_at | NM_005374.3 | 4355 | Hs.514208 |
| MPP2 | 207984_s_at | NM_005374.3 | 4355 | Hs.514208 |
| MPP3 | 206186_at | NM_001932.2 | 4356 | Hs.396566 |
| MPP5 | 219321_at | NM_022474.2 | 64398 | Hs.509699 |
| MPP5 | 226092_at | NM_022474.2 | 64398 | Hs.509699 |
| MPP5 | 235864_at | NM_022474 | 64398 | Hs.109122 |
| MPP5 | 242779_at | NM_022474 | 64398 | Hs.109122 |
| MPPE1 | 213727_x_at | NM_138608.2 | 65258 | Hs.514713 |
| MPPE1 | 213924_at | NM_023075 | 65258 | Hs.154145 |
| MPPE1 | 206764_x_at | NM_138608.2 | 65258 | Hs.514713 |
| MPPE1 | 209858_x_at | NM_138608.2 | 65258 | Hs.514713 |
| MPZ | 210280_at | NM_000530.4 | 4359 | Hs.93883 |
| MPZL1 | 231621_at | NM_003953 | 9019 | Hs.14891 |
| MPZL1 | 201874_at | NM_003953.4 | 9019 | Hs.493919 |
| MPZL1 | 201875_s_at | NM_003953.4 | 9019 | Hs.493919 |
| MPZL1 | 210087_s_at | NM_003953.4 | 9019 | Hs.493919 |
| MPZL1 | 210210_at | NM_003953 | 9019 | Hs.493919 |
| MPZL1 | 210594_x_at | NM_024569.3 | 9019 | Hs.493919 |
| MR1 | 207565_s_at | NM_001531.1 | 3140 | Hs.101840 |
| MR1 | 207566_at | NM_001531.1 | 3140 | Hs.101840 |
| MR1 | 210223_s_at | NM_001531.1 | 3140 | Hs.101840 |
| MR1 | 210224_at | NM_001531 | 3140 | Hs.101840 |
| MR1 | 210528_at | NM_001531 | 3140 | Hs.101840 |
| MRAP | 1554044_a_at | NM_178817.3 | 56246 | Hs.473611 |
| MRAP | 1555740_a_at | NM_178817.3 | 56246 | Hs.473611 |
| MRAP | 1555741_at | NM_206898.1 | 56246 | Hs.473611 |
| MRC1 | 204438_at | NM_002438.1 | 4360 | Hs.75182 |
| MRC2 | 37408_at | NM_006039 | 9902 | Hs.7835 |
| MRC2 | 209280_at | NM_006039 | 9902 | Hs.7835 |
| MRGPRF | 227727_at | NM_145015.2 | 219928 | Hs.118513 |
| MRGPRX1 | 1553401_at | NM_147199.2 | 259249 | Hs.350565 |
| MRGPRX2 | 1553889_at | NM_054030.2 | 117194 | Hs.350566 |
| MRGPRX3 | 1553293_at | NM_054031.2 | 117195 | Hs.380177 |
| MRGPRX4 | 1553504_at | NM_054032.2 | 117196 | Hs.356858 |
| MS4A1 | 217418_x_at | NM_021950.3 | 931 | Hs.438040 |
| MS4A1 | 228592_at | NM_021950.3 | 931 | Hs.438040 |
| MS4A1 | 228599_at | NM_021950.3 | 931 | Hs.438040 |
| MS4A1 | 231418_at | NM_021950 | 931 | Hs.438040 |
| MS4A1 | 210356_x_at | NM_021950.3 | 931 | Hs.438040 |
| MS4A12 | 220834_at | NM_017716.1 | 54860 | Hs.272789 |
| MS4A2 | 207496_at | NM_000139.2 | 2206 | Hs.386748 |
| MS4A2 | 207497_s_at | NM_000139.2 | 2206 | Hs.386748 |
| MS4A3 | 1554892_a_at | NM_001031666.1 | 932 | Hs.99960 |
| MS4A3 | 210254_at | NM_001031666.1 | 932 | Hs.99960 |
| MS4A4A | 219607_s_at | NM_024021.2 | 51338 | Hs.325960 |
| MS4A4A | 224357_s_at | NM_148975.1 | 51338 | Hs.325960 |
| MS4A4A | 1555728_a_at | NM_024021.2 | 51338 | Hs.325960 |
| MS4A5 | 220790_s_at | NM_023945.2 | 64232 | Hs.178066 |
| MS4A6A | 219666_at | NM_022349.2 | 64231 | Hs.523702 |
| MS4A6A | 223280_x_at | NM_152852.1 | 64231 | Hs.523702 |
| MS4A6A | 223922_x_at | NM_152852.1 | 64231 | Hs.523702 |
| MS4A6A | 224356_x_at | NM_022349.2 | 64231 | Hs.523702 |
| MS4A6A | 230550_at | NM_152851.1 | 64231 | Hs.523702 |
| MS4A6A | 232724_at | NM_022349 | 64231 | Hs.371612 |
| MS4A6A | 232725_s_at | NM_022349 | 64231 | Hs.371612 |
| MS4A6E | 231335_at | NM_139249.2 | 245802 | Hs.126580 |
| MS4A7 | 223343_at | NM_021201.4 | 58475 | Hs.530735 |
| MS4A7 | 223344_s_at | NM_206940.1 | 58475 | Hs.530735 |
| MS4A7 | 224358_s_at | NM_206938.1 | 58475 | Hs.530735 |
| MS4A8B | 224355_s_at | NM_031457.1 | 83661 | Hs.150878 |
| MSLN | 204885_s_at | NM_013404.3 | 10232 | Hs.408488 |
| MSMB | 207430_s_at | NM_002443.2 | 4477 | Hs.255462 |
| MSMB | 210297_s_at | NM_002443.2 | 4477 | Hs.255462 |
| MSR1 | 214770_at | NM_002445 | 4481 | Hs.446291 |
| MSR1 | 208422_at | NM_002445 | 4481 | Hs.446291 |
| MSR1 | 208423_s_at | NM_002445 | 4481 | Hs.446291 |
| MSR1 | 211887_x_at | NM_002445 | 4481 | Hs.446291 |
| MST1 | 213382_at | NM_020998 | 11223 | 126678 |
| MST1 | 216320_x_at | NM_020998.2 | 4485 | Hs.349110 |
| MST1 | 242015_x_at | NM_020998 | 4485 | Hs.349110 |
| MST1 | 205614_x_at | NM_020998.2 | 4485 | Hs.349110 |
| MST1R | 205455_at | NM_002447.1 | 4486 | Hs.517973 |
| MSTP9 | 213380_x_at | NM_020998 | 11223 | Hs.349110 |
| MSTP9 | 215563_s_at | NM_020998 | 11223 | Hs.349110 |
| MTFR1 | 203207_s_at | NM_014637.2 | 9650 | Hs.521608 |
| MTFR1 | 203208_s_at | NM_014637.2 | 9650 | Hs.521608 |
| MTNR1A | 221369_at | NM_005958.3 | 4543 | Hs.243467 |
| MTNR1B | 208516_at | NM_005959.3 | 4544 | Hs.147853 |
| MTUS1 | 212093_s_at | NM_001001924.1 | 57509 | Hs.7946 |
| MTUS1 | 212095_s_at | NM_001001924.1 | 57509 | Hs.7946 |
| MTUS1 | 212096_s_at | NM_001001924.1 | 57509 | Hs.7946 |
| MUC1 | 213693_s_at | NM_001018016.1 | 4582 | Hs.89603 |
| MUC1 | 207847_s_at | NM_001018016.1 | 4582 | Hs.89603 |
| MUC1 | 211695_x_at | NM_002456.4 | 4582 | Hs.89603 |
| MUC12 | 226654_at | AF147790 | 10071 | Hs.489355 |
| MUC12 | 231814_at | AF147791.1 | 10071 | Hs.489355 |
| MUC13 | 218687_s_at | NM_033049.2 | 56667 | Hs.5940 |
| MUC13 | 222712_s_at | NM_033049.2 | 56667 | Hs.5940 |
| MUC15 | 227238_at | NM_145650 | 143662 | Hs.407152 |
| MUC15 | 227241_at | NM_145650.2 | 143662 | Hs.407152 |
| MUC16 | 220196_at | NM_024690.2 | 94025 | Hs.432676 |
| MUC17 | 232321_at | NM_001040105.1 | 140453 | Hs.271819 |
| MUC17 | 232407_at | AK026404 | 140453 | Hs.271819 |
| MUC19 | 1553436_at | AK093065.1 | 283463 | Hs.244017 |
| MUC2 | 204673_at | NM_002457.1 | 4583 | Hs.315 |
| MUC20 | 226622_at | NM_152673.1 | 200958 | Hs.69321 |
| MUC20 | 231941_s_at | NM_152673.1 | 200958 | Hs.69321 |
| MUC3A | 217117_x_at | AF007194.1 | 4584 | Hs.489354 |
| MUC3B | 214676_x_at | AF113616 | 57876 | Hs.489354 |
| MUC3B | 214898_x_at | AB038783 | 57876 | Hs.489354 |
| MUC3B | 217054_at | AF007194 | 57876 | Hs.129782 |
| MUC3B | 1561421_a_at | AK057259 | 57876 | Hs.129782 |
| MUC4 | 217109_at | NM_004532.2 | 4585 | Hs.369646 |
| MUC4 | 217110_s_at | NM_004532.2 | 4585 | Hs.369646 |
| MUC4 | 1561785_at | NM_004532 | 4585 | Hs.198267 |
| MUC4 | 204895_x_at | NM_004532.2 | 4585 | Hs.369646 |
| MUC5AC | 214303_x_at | AW192795 | 4586 | Hs.558950 |
| MUC5AC | 214385_s_at | AI521646 | 4586 | Hs.534332 |
| MUC5AC | 217182_at | XM_001134429 | 4586 | - |
| MUC5AC | 217187_at | Z34278.1 | 4586 | Hs.534332 |
| MUC5B | 213432_at | AI697108 | 4587 | Hs.523395 |
| MUC5B | 222268_x_at | XM_938837.1 | 649768 | Hs.102482 |
| MUC6 | 214133_at | AI611214 | 4588 | Hs.528432 |
| MUC6 | 1565662_at | BF476613 | 4588 | Hs.398100 |
| MUC6 | 1565666_s_at | XM_927562.1 | 642724 | Hs.398100 |
| MUC7 | 217059_at | NM_152291.1 | 4589 | Hs.103944 |
| MUCDHL | 219796_s_at | NM_021924.2 | 53841 | Hs.165619 |
| MUCDHL | 220074_at | NM_017717.3 | 53841 | Hs.165619 |
| MUCDHL | 220075_s_at | NM_017717.3 | 53841 | Hs.165619 |
| MUSK | 241122_s_at | NM_005592.1 | 4593 | Hs.521653 |
| MUSK | 207632_at | NM_005592.1 | 4593 | Hs.521653 |
| MUSK | 207633_s_at | NM_005592.1 | 4593 | Hs.521653 |
| MXRA5 | 209596_at | NM_015419.1 | 25878 | Hs.369422 |
| MXRA7 | 227326_at | NM_001008528.1 | 439921 | Hs.369125 |
| MXRA7 | 212509_s_at | NM_001008529.1 | 439921 | Hs.250723 |
| MXRA8 | 213422_s_at | NM_032348.2 | 54587 | Hs.515687 |
| MYADM | 224920_x_at | NM_001020818.1 | 91663 | Hs.380906 |
| MYADM | 225673_at | NM_001020818.1 | 91663 | Hs.380906 |
| MYEF2 | 232672_x_at | NM_016132 | 50804 | Hs.44268 |
| MYOC | 210155_at | NM_000261.1 | 4653 | Hs.436037 |
| MYT2 | 207588_at | NM_003871 | 8827 | Hs.123048 |
| na | 1563638_at | AL833564 | 283816 | Hs.388854 |
| na | 214777_at | BG482805 | 339726 | Hs.377975 |
| na | 215503_at | AW118166 | 350918 | Hs.370724 |
| na | 217034_at | AF103529 | 339726 | Hs.377975 |
| na | 224604_at | AK025703.1 | 401152 | Hs.173705 |
| na | 226683_at | AU146771 | 345638 | Hs.432755 |
| na | 227610_at | BE858239 | 93259 | Hs.437875 |
| na | 228618_at | XM_938090.1 | 375033 | Hs.142003 |
| na | 230244_at | BC035093.2 | 389084 | Hs.567357 |
| na | 230445_at | XM_371078.2 | 388419 | Hs.211870 |
| na | 231462_at | BE467208 | 283953 | Hs.445302 |
| na | 232299_at | BC049848.1 | 389084 | Hs.567357 |
| na | 235265_at | BF446017 | 344287 | Hs.259495 |
| na | 235371_at | AI452595 | 351391 | Hs.97600 |
| na | 235544_x_at | XM_937866.1 | 284069 | Hs.154001 |
| na | 236667_at | BE218186 | 144404 | Hs.303277 |
| na | 236981_at | AI242058 | 147137 | Hs.14706 |
| na | 238014_at | AW291339 | 200448 | Hs.342585 |
| na | 240514_at | AA682371 | 283198 | Hs.378194 |
| na | 240878_at | BC033882.1 | 374768 | Hs.499707 |
| na | 243337_at | BE223071 | 166752 | Hs.388278 |
| NAALADL1 | 228424_at | NM_005468.2 | 10004 | Hs.13967 |
| NAALADL1 | 207895_at | NM_005468.2 | 10004 | Hs.13967 |
| NAALADL2 | 1557998_at | NM_207015.1 | 254827 | Hs.86024 |
| NAT8 | 206963_s_at | NM_003960.2 | 9027 | Hs.14637 |
| NAT8 | 210289_at | NM_003960.2 | 9027 | Hs.14637 |
| NAT8B | 206964_at | NM_016347.1 | 51471 | Hs.458287 |
| NAT8L | 235316_at | NM_178557.2 | 339983 | Hs.318529 |
| NBEA | 221207_s_at | NM_015678.3 | 26960 | Hs.491172 |
| NBEA | 226439_s_at | NM_015678.3 | 26960 | Hs.491172 |
| NBL1 | 37005_at | NM_005380.3 | 4681 | Hs.439671 |
| NBL1 | 201621_at | NM_005380.3 | 4681 | Hs.439671 |
| NCAM1 | 214952_at | NM_000615 | 4684 | Hs.555028 |
| NCAM1 | 217359_s_at | NM_000615 | 4684 | Hs.555028 |
| NCAM1 | 229799_s_at | NM_000615 | 4684 | Hs.555028 |
| NCAM1 | 231532_at | NM_000615 | 4684 | Hs.78792 |
| NCAM1 | 209968_s_at | NM_000615.3 | 4684 | Hs.555028 |
| NCAM1 | 212843_at | NM_000615 | 4684 | Hs.555028 |
| NCAM2 | 232390_at | NM_004540 | 4685 | Hs.135892 |
| NCAM2 | 205669_at | NM_004540 | 4685 | Hs.473450 |
| NCKAP1 | 207738_s_at | NM_205842.1 | 10787 | Hs.516633 |
| NCKAP1L | 209734_at | NM_005337.3 | 3071 | Hs.182014 |
| NCR1 | 217088_s_at | NM_004829.4 | 9437 | Hs.97084 |
| NCR1 | 217095_x_at | NM_004829.4 | 9437 | Hs.97084 |
| NCR1 | 207860_at | NM_004829 | 9437 | Hs.97084 |
| NCR2 | 217045_x_at | NM_004828 | 9436 | Hs.194721 |
| NCR2 | 217493_x_at | NM_004828 | 9436 | Hs.194721 |
| NCR2 | 221075_s_at | NM_004828.2 | 9436 | Hs.194721 |
| NCR3 | 210763_x_at | NM_147130.1 | 259197 | Hs.509513 |
| NCR3 | 211010_s_at | NM_147130.1 | 259197 | Hs.509513 |
| NCR3 | 211583_x_at | NM_147130.1 | 259197 | Hs.509513 |
| NCSTN | 237076_at | NM_015331 | 23385 | Hs.4788 |
| NCSTN | 208759_at | NM_015331.2 | 23385 | Hs.517249 |
| NDP | 206022_at | NM_000266.1 | 4693 | Hs.522615 |
| NECL1 | 216535_at | NM_021189 | 57863 | Hs.365689 |
| NECL1 | 226953_at | NM_021189 | 57863 | Hs.365689 |
| NEGR1 | 229461_x_at | NM_173808.2 | 257194 | Hs.146542 |
| NEGR1 | 239548_at | NM_173808.2 | 257194 | Hs.146542 |
| NEGR1 | 243357_at | NM_173808.2 | 257194 | Hs.146542 |
| NEGR1 | 1553194_at | NM_173808.2 | 257194 | Hs.146542 |
| NELL1 | 1554394_at | AB085898.1 | 4745 | Hs.502145 |
| NELL1 | 206089_at | NM_006157.2 | 4745 | Hs.502145 |
| NELL2 | 203413_at | NM_006159.1 | 4753 | Hs.505326 |
| NENF | 214075_at | NM_013349.3 | 29937 | Hs.461787 |
| NENF | 218407_x_at | NM_013349.3 | 29937 | Hs.461787 |
| NEO1 | 225270_at | NM_002499 | 4756 | Hs.388613 |
| NEO1 | 229877_at | NM_002499 | 4756 | Hs.388613 |
| NEO1 | 204321_at | NM_002499.1 | 4756 | Hs.388613 |
| NETO1 | 1552736_a_at | NM_138966.2 | 81832 | Hs.558557 |
| NETO1 | 1552904_at | NM_138999.1 | 81832 | Hs.558557 |
| NETO1 | 1562713_a_at | NM_138966.2 | 81832 | Hs.558557 |
| NETO2 | 218888_s_at | NM_018092.3 | 81831 | Hs.444046 |
| NETO2 | 222774_s_at | NM_018092.3 | 81831 | Hs.444046 |
| NEU3 | 216083_s_at | NM_006656.4 | 10825 | Hs.191074 |
| NEU3 | 206948_at | NM_006656.4 | 10825 | Hs.191074 |
| NF2 | 217150_s_at | NM_181825.1 | 4771 | Hs.187898 |
| NF2 | 218915_at | NM_000268.2 | 4771 | Hs.187898 |
| NF2 | 238618_at | NM_000268.2 | 4771 | Hs.187898 |
| NF2 | 204991_s_at | NM_000268.2 | 4771 | Hs.187898 |
| NF2 | 210767_at | NM_181825.1 | 4771 | Hs.187898 |
| NF2 | 211017_s_at | NM_000268.2 | 4771 | Hs.187898 |
| NF2 | 211091_s_at | NM_000268.2 | 4771 | Hs.187898 |
| NF2 | 211092_s_at | NM_181827.1 | 4771 | Hs.187898 |
| NFAM1 | 230322_at | NM_145912.4 | 150372 | Hs.436677 |
| NFAM1 | 243099_at | NM_145912 | 150372 | Hs.436677 |
| NFASC | 213438_at | NM_015090.2 | 23114 | Hs.13349 |
| NFASC | 230242_at | NM_015090.2 | 23114 | Hs.13349 |
| NFASC | 243645_at | NM_015090.2 | 23114 | Hs.13349 |
| NGFB | 206814_at | NM_002506.2 | 4803 | Hs.2561 |
| NGFR | 205858_at | NM_002507.1 | 4804 | Hs.415768 |
| NHEDC1 | 1553633_s_at | NM_139173.2 | 150159 | Hs.534678 |
| NHEDC1 | 1555141_a_at | NM_139173.2 | 150159 | Hs.534678 |
| NHEDC1 | 1555142_at | NM_139173 | 150159 | Hs.534678 |
| NHLRC3 | 227040_at | NM_001012754.2 | 387921 | Hs.507783 |
| NHLRC3 | 239314_at | NM_001012754.2 | 387921 | Hs.507783 |
| NID1 | 1561082_at | BC012501.1 | 4811 | Hs.356624 |
| NID1 | 202007_at | BC045606.1 | 4811 | Hs.356624 |
| NID1 | 202008_s_at | NM_002508.1 | 4811 | Hs.356624 |
| NID2 | 204114_at | NM_007361.2 | 22795 | Hs.369840 |
| NIFIE14 | 202475_at | NM_006326 | 10430 | Hs.9234 |
| NINJ1 | 203045_at | NM_004148.2 | 4814 | Hs.494457 |
| NINJ2 | 219594_at | NM_016533.4 | 4815 | Hs.504422 |
| NIPA1 | 225752_at | NM_144599.3 | 123606 | Hs.511797 |
| NIPA1 | 1552696_at | NM_144599.3 | 123606 | Hs.511797 |
| NIPA2 | 212129_at | NM_001008894.1 | 81614 | Hs.370367 |
| NIPA2 | 212133_at | NM_030922 | 81614 | Hs.83724 |
| NKG7 | 213915_at | NM_005601.3 | 4818 | Hs.10306 |
| NKPD1 | 1560430_at | NM_198478.1 | 284353 | Hs.299256 |
| NKTR | 215338_s_at | NM_001012651.1 | 4820 | Hs.529509 |
| NKTR | 215339_at | NM_005385 | 4820 | Hs.529509 |
| NKTR | 231235_at | NM_005385 | 4820 | Hs.369815 |
| NKTR | 239013_at | NM_005385 | 4820 | Hs.369815 |
| NKTR | 1557736_at | NM_005385 | 4820 | Hs.529509 |
| NKTR | 1557737_s_at | NM_005385 | 4820 | Hs.529509 |
| NKTR | 1570342_at | NM_005385 | 4820 | Hs.529509 |
| NKTR | 202379_s_at | NM_001012651.1 | 4820 | Hs.529509 |
| NKTR | 202380_s_at | NM_001012651.1 | 4820 | Hs.529509 |
| NLGN1 | 231361_at | NM_014932 | 22871 | Hs.71132 |
| NLGN1 | 205893_at | NM_014932.2 | 22871 | Hs.478289 |
| NLGN2 | 226288_s_at | NM_020795.2 | 57555 | Hs.26229 |
| NLGN2 | 235838_at | NM_020795 | 57555 | Hs.26229 |
| NLGN2 | 1554428_s_at | NM_020795.2 | 57555 | Hs.26229 |
| NLGN3 | 219726_at | NM_018977.2 | 54413 | Hs.438877 |
| NLGN3 | 234751_s_at | NM_018977.2 | 54413 | Hs.438877 |
| NLGN3 | 239632_at | NM_018977 | 54413 | Hs.438877 |
| NLGN4X | 221933_at | NM_020742.2 | 57502 | Hs.21107 |
| NLGN4X | 1554689_a_at | NM_020742.2 | 57502 | Hs.21107 |
| NLGN4Y | 1554125_a_at | NM_014893 | 22829 | Hs.439199 |
| NLGN4Y | 207703_at | NM_014893 | 22829 | Hs.439199 |
| NMB | 205204_at | NM_205858.1 | 4828 | Hs.386470 |
| NMBR | 207333_at | NM_002511.1 | 4829 | Hs.533948 |
| NMU | 206023_at | NM_006681.1 | 10874 | Hs.418367 |
| NMUR1 | 221383_at | NM_006056.2 | 10316 | Hs.471619 |
| NMUR2 | 224088_at | NM_020167.3 | 56923 | Hs.283093 |
| NNAT | 204239_s_at | NM_005386.2 | 4826 | Hs.504703 |
| NODAL | 220689_at | NM_018055 | 4838 | Hs.370414 |
| NODAL | 230916_at | NM_018055.3 | 4838 | Hs.370414 |
| NODAL | 237896_at | NM_018055 | 4838 | Hs.370414 |
| NOG | 231798_at | NM_005450 | 9241 | Hs.248201 |
| NOS3 | 229093_at | NM_000603 |  |  |
| NOS3 | 205581_s_at | NM_000603.3 | 4846 | Hs.511603 |
| NOTCH1 | 218902_at | NM_017617.2 | 4851 | Hs.495473 |
| NOTCH1 | 223508_at | NM_017617 | 4851 | Hs.495473 |
| NOTCH1 | 231660_at | BF508721 | 4851 | Hs.495473 |
| NOTCH2 | 227067_x_at | NM_024408.2 | 4853 | Hs.487360 |
| NOTCH2 | 202443_x_at | NM_024408.2 | 4853 | Hs.487360 |
| NOTCH2 | 202445_s_at | NM_024408.2 | 4853 | Hs.502564 |
| NOTCH2 | 210756_s_at | NM_024408.2 | 4853 | Hs.487360 |
| NOTCH2 | 212377_s_at | NM_024408.2 | 4853 | Hs.487360 |
| NOTCH2NL | 214722_at | AL833369.1 | 388677 | Hs.487360 |
| NOTCH3 | 203237_s_at | NM_000435.1 | 4854 | Hs.8546 |
| NOTCH3 | 203238_s_at | NM_000435.1 | 4854 | Hs.8546 |
| NOTCH4 | 240786_at | NM_004557 | 4855 | Hs.436100 |
| NOTCH4 | 205247_at | NM_004557.3 | 4855 | Hs.436100 |
| NOTUM | 228649_at | NM_178493.3 | 147111 | Hs.106137 |
| NOV | 214321_at | NM_002514 | 4856 | Hs.235935 |
| NOV | 204501_at | NM_002514.2 | 4856 | Hs.235935 |
| NOX4 | 219773_at | NM_016931.2 | 50507 | Hs.448667 |
| NPAL1 | 232158_x_at | CR749484.1 | 152519 | Hs.134190 |
| NPAL2 | 220128_s_at | NM_024759.1 | 79815 | Hs.309489 |
| NPAL3 | 214579_at | NM_020448.2 | 57185 | Hs.523442 |
| NPAL3 | 225875_s_at | NM_020448.2 | 57185 | Hs.523442 |
| NPAL3 | 225876_at | NM_020448.2 | 57185 | Hs.523442 |
| NPAL3 | 210267_at | BC001265.1 | 57185 | Hs.523442 |
| NPB | 230044_at | NM_148896 | 256933 | Hs.435721 |
| NPB | 1553663_a_at | NM_148896.2 | 256933 | Hs.558709 |
| NPC1 | 217584_at | NM_000271 | 4864 | Hs.404930 |
| NPC1 | 238568_s_at | NM_000271 | 4864 | Hs.404930 |
| NPC1 | 238873_at | NM_000271 | 4864 | Hs.404930 |
| NPC1 | 202679_at | NM_000271.2 | 4864 | Hs.464779 |
| NPC1L1 | 220106_at | NM_013389.1 | 29881 | Hs.408111 |
| NPC1L1 | 224305_s_at | NM_013389.1 | 29881 | Hs.408111 |
| NPC1L1 | 224306_at | NM_013389 | 29881 | Hs.408111 |
| NPDC1 | 218086_at | NM_015392.2 | 56654 | Hs.105547 |
| NPFF | 214184_at | NM_003717 | 8620 | Hs.104555 |
| NPFF | 206402_s_at | NM_003717.1 | 8620 | Hs.104555 |
| NPHS1 | 207673_at | NM_004646.1 | 4868 | Hs.122186 |
| NPHS2 | 220424_at | NM_014625.1 | 7827 | Hs.412710 |
| NPNT | 225911_at | NM_001033047.1 | 255743 | Hs.518921 |
| NPPA | 209957_s_at | NM_006172.1 | 4878 | Hs.75640 |
| NPPB | 206801_at | NM_002521.2 | 4879 | Hs.219140 |
| NPPC | 221348_at | NM_024409.1 | 4880 | Hs.247916 |
| NPR1 | 32625_at | NM_000906.2 | 4881 | Hs.490330 |
| NPR1 | 204648_at | NM_000906.2 | 4881 | Hs.490330 |
| NPR2 | 214066_x_at | NM_003995.3 | 4882 | Hs.78518 |
| NPR2 | 204310_s_at | NM_003995.3 | 4882 | Hs.78518 |
| NPR3 | 219789_at | NM_000908.2 | 4883 | Hs.237028 |
| NPR3 | 219790_s_at | NM_000908.2 | 4883 | Hs.237028 |
| NPTX2 | 213479_at | NM_002523.1 | 4885 | Hs.3281 |
| NPTXR | 213040_s_at | NM_058178.1 | 23467 | Hs.91622 |
| NPW | 243110_x_at | XM_496111.2 | 283869 | Hs.233533 |
| NPY | 206001_at | NM_000905.2 | 4852 | Hs.1832 |
| NPY1R | 205440_s_at | NM_000909 | 4886 | Hs.519057 |
| NPY2R | 208379_x_at | NM_000910 | 4887 | Hs.37125 |
| NPY2R | 210729_at | NM_000910.2 | 4887 | Hs.37125 |
| NPY2R | 210730_s_at | NM_000910.2 | 4887 | Hs.37125 |
| NPY5R | 207400_at | NM_006174.2 | 4889 | Hs.519058 |
| NPY6R | 236860_at | XR_000577.1 | 4888 | Hs.529445 |
| NPY6R | 210444_at | XR_000088 | 4888 | Hs.529445 |
| NRCAM | 216959_x_at | NM_001037132.1 | 4897 | Hs.21422 |
| NRCAM | 204105_s_at | NM_001037132.1 | 4897 | Hs.21422 |
| NRD1 | 229422_at | NM_002525 | 4898 | Hs.4099 |
| NRD1 | 242235_x_at | NM_002525 | 4898 | Hs.4099 |
| NRD1 | 242994_at | NM_002525 | 4898 | Hs.4099 |
| NRD1 | 208709_s_at | NM_002525.1 | 4898 | Hs.554766 |
| NRG1 | 206237_s_at | NM_013956.1 | 3084 | Hs.453951 |
| NRG1 | 206343_s_at | NM_013958.1 | 3084 | Hs.453951 |
| NRG1 | 208230_s_at | NM_013956.1 | 3084 | Hs.453951 |
| NRG1 | 208231_at | NM_013960.1 | 3084 | Hs.453951 |
| NRG1 | 208241_at | NM_004495.1 | 3084 | Hs.453951 |
| NRG2 | 206879_s_at | NM_004883.1 | 9542 | Hs.408515 |
| NRG2 | 208062_s_at | NM_004883.1 | 9542 | Hs.408515 |
| NRG3 | 229233_at | H05240 | 10718 | Hs.444783 |
| NRG3 | 234505_at | AL049357 | 10718 | 125119 |
| NRG3 | 234510_at | AL049357 | 10718 | 125119 |
| NRG3 | 1563494_at | AL161982 | 10718 | 125119 |
| NRG4 | 242426_at | NM_138573 | 145957 | Hs.238914 |
| NRN1 | 218625_at | NM_016588.2 | 51299 | Hs.103291 |
| NRP1 | 233626_at | NM_003873 | 8829 | Hs.173548 |
| NRP1 | 233701_at | NM_003873 | 8829 | Hs.173548 |
| NRP1 | 242677_at | NM_003873 | 8829 | Hs.173548 |
| NRP1 | 1561365_at | NM_003873 | 8829 | Hs.173548 |
| NRP1 | 210510_s_at | NM_003873.3 | 8829 | Hs.131704 |
| NRP1 | 210615_at | NM_003873 | 8829 | Hs.131704 |
| NRP1 | 212298_at | NM_003873.3 | 8829 | Hs.131704 |
| NRP2 | 214632_at | NM_003872.2 | 8828 | Hs.471200 |
| NRP2 | 219367_s_at | NM_003872 | 8828 | Hs.368746 |
| NRP2 | 222877_at | NM_003872 | 8828 | Hs.368746 |
| NRP2 | 223510_at | NM_018534.3 | 8828 | Hs.471200 |
| NRP2 | 225566_at | NM_003872.2 | 8828 | Hs.471200 |
| NRP2 | 228102_at | NM_003872 | 8828 | Hs.368746 |
| NRP2 | 228103_s_at | NM_003872 | 8828 | Hs.368746 |
| NRP2 | 228699_at | NM_003872 | 8828 | Hs.368746 |
| NRP2 | 229225_at | NM_003872.2 | 8828 | Hs.471200 |
| NRP2 | 230410_at | NM_003872 | 8828 | Hs.368746 |
| NRP2 | 232701_at | AU159344 |  | Hs.287606 |
| NRP2 | 1555468_at | NM_003872 | 8828 | Hs.471200 |
| NRP2 | 210841_s_at | NM_201267.1 | 8828 | Hs.471200 |
| NRP2 | 210842_at | NM_201264.1 | 8828 | Hs.471200 |
| NRP2 | 211844_s_at | NM_003872.2 | 8828 | Hs.471200 |
| NRTN | 210683_at | NM_004558.2 | 4902 | Hs.234775 |
| NRXN1 | 216096_s_at | NM_004801.2 | 9378 | Hs.468505 |
| NRXN1 | 228547_at | NM_004801 | 9378 | Hs.468505 |
| NRXN1 | 237535_x_at | NM_004801 | 9378 | Hs.22998 |
| NRXN1 | 1558708_at | NM_004801 | 9378 | Hs.468505 |
| NRXN1 | 209914_s_at | NM_004801.2 | 9378 | Hs.468505 |
| NRXN1 | 209915_s_at | NM_004801.2 | 9378 | Hs.468505 |
| NRXN2 | 209982_s_at | NM_015080.2 | 9379 | Hs.372938 |
| NRXN2 | 209983_s_at | NM_138734.1 | 9379 | Hs.372938 |
| NRXN3 | 215020_at | NM_004796 | 9369 | Hs.368307 |
| NRXN3 | 215021_s_at | NM_004796.3 | 9369 | Hs.368307 |
| NRXN3 | 229649_at | NM_004796.3 | 9369 | Hs.368307 |
| NRXN3 | 205795_at | NM_004796.3 | 9369 | Hs.368307 |
| NT5E | 227486_at | NM_002526 | 4907 | Hs.153952 |
| NT5E | 1553994_at | NM_002526 | 4907 | Hs.153952 |
| NT5E | 1553995_a_at | NM_002526.1 | 4907 | Hs.153952 |
| NT5E | 203939_at | NM_002526.1 | 4907 | Hs.153952 |
| NTF3 | 206706_at | NM_002527.3 | 4908 | Hs.99171 |
| NTF5 | 231785_at | NM_006179 | 4909 | Hs.266902 |
| NTN1 | 208005_at | NM_004822.1 | 9423 | Hs.128002 |
| NTN2L | 207640_x_at | NM_006181.1 | 4917 | Hs.158336 |
| NTN4 | 223315_at | NM_021229.3 | 59277 | Hs.201034 |
| NTN4 | 234202_at | AK025670 | 59277 | 201034 |
| NTNG1 | 236088_at | NM_014917.2 | 22854 | Hs.211236 |
| NTNG1 | 1561767_at | S50182 | 22854 | 143707 |
| NTNG1 | 206713_at | AB023193.1 | 22854 | Hs.211236 |
| NTNG2 | 233072_at | NM_032536.1 | 84628 | Hs.163642 |
| NTRK1 | 208605_s_at | NM_001007792.1 | 4914 | Hs.406293 |
| NTRK2 | 214680_at | NM_001007097.1 | 4915 | Hs.132293 |
| NTRK2 | 221795_at | NM_001007097.1 | 4915 | Hs.132293 |
| NTRK2 | 221796_at | NM_001007097.1 | 4915 | Hs.132293 |
| NTRK2 | 229463_at | BX649001.1 | 4915 | Hs.132293 |
| NTRK2 | 236095_at | NM_001018065.1 | 4915 | Hs.132293 |
| NTRK2 | 207152_at | NM_001018064.1 | 4915 | Hs.132293 |
| NTRK3 | 215025_at | NM_001007156.1 | 4916 | Hs.410969 |
| NTRK3 | 215115_x_at | NM_001012338.1 | 4916 | Hs.410969 |
| NTRK3 | 217033_x_at | NM_002530.2 | 4916 | Hs.410969 |
| NTRK3 | 217377_x_at | NM_002530.2 | 4916 | Hs.410969 |
| NTRK3 | 228849_at | NM_001007156.1 | 4916 | Hs.410969 |
| NTRK3 | 1557795_s_at | NM_001007156.1 | 4916 | Hs.410969 |
| NTRK3 | 206462_s_at | NM_001012338.1 | 4916 | Hs.410969 |
| NTS | 206291_at | NM_006183.3 | 4922 | Hs.80962 |
| NTSR1 | 207360_s_at | NM_002531.1 | 4923 | Hs.110642 |
| NTSR2 | 206899_at | NM_012344.2 | 23620 | Hs.131138 |
| NUCB1 | 200646_s_at | NM_006184.3 | 4924 | Hs.515524 |
| NUCB1 | 200649_at | NM_006184.3 | 4924 | Hs.515524 |
| NUCB2 | 229838_at | NM_005013 | 4925 | Hs.128686 |
| NUCB2 | 203675_at | NM_005013.1 | 4925 | Hs.128686 |
| NUMB | 230462_at | NM_003744 | 8650 | Hs.509909 |
| NUMB | 236930_at | NM_003744 | 8650 | Hs.445301 |
| NUMB | 207545_s_at | NM_001005743.1 | 8650 | Hs.509909 |
| NUMB | 209073_s_at | NM_001005744.1 | 8650 | Hs.509909 |
| NUP210L | 232598_at | NM_207308.1 | 91181 | Hs.67639 |
| NXPH1 | 232377_at | NM_152745.2 | 30010 | Hs.487564 |
| NXPH2 | 230883_at | AI765540 | 11249 | Hs.435019 |
| NXPH2 | 232334_at | AI984820 | 11249 | Hs.435019 |
| NXPH2 | 1559355_at | NM_007226.1 | 11249 | Hs.435019 |
| NXPH3 | 221991_at | NM_007225.1 | 11248 | Hs.55069 |
| NXPH3 | 228210_at | NM_007225 | 11248 | Hs.55069 |
| NXPH4 | 221967_at | NM_007224.1 | 11247 | Hs.534396 |
| NYX | 221684_s_at | NM_022567.1 | 60506 | Hs.302019 |
| NYX | 234496_x_at | NM_022567.1 | 60506 | Hs.302019 |
| OACT1 | 227379_at | AI734993 | 154141 | Hs.377830 |
| OACT2 | 226726_at | NM_138799.2 | 129642 | Hs.467634 |
| OAS1 | 202869_at | NM_016816.2 | 4938 | Hs.524760 |
| OAS1 | 205552_s_at | NM_001032409.1 | 4938 | Hs.524760 |
| OBP2A | 220848_x_at | NM_014582 | 29991 | Hs.558492 |
| OBP2A | 234436_x_at | NM_014582 | 29991 | Hs.558492 |
| OBP2A | 234841_x_at | NM_014582 | 29991 | Hs.558492 |
| OBP2B | 220959_s_at | NM_014581.2 | 29989 | Hs.449629 |
| OBP2B | 233860_s_at | NM_014581.2 | 29989 | Hs.449629 |
| OBP2B | 234722_x_at | AJ251027.1 | 29989 | Hs.449629 |
| OCA2 | 206498_at | NM_000275.1 | 4948 | Hs.130937 |
| OCLN | 209925_at | NM_002538.2 | 4950 | Hs.482439 |
| ODF1 | 214485_at | NM_024410.2 | 4956 | Hs.159274 |
| ODF4 | 1552408_at | NM_153007.3 | 146852 | Hs.186045 |
| ODF4 | 1552409_a_at | NM_153007.3 | 146852 | Hs.186045 |
| ODZ1 | 1553007_a_at | NM_014253 | 10178 | Hs.23796 |
| ODZ2 | 231867_at | AB032953 | 57451 | Hs.155915 |
| ODZ3 | 219523_s_at | NM_018104 | 55714 | Hs.130438 |
| ODZ3 | 227050_at | AI928518 | 55714 | Hs.41793 |
| ODZ3 | 1559661_at | AF195420 | 55714 | Hs.130438 |
| ODZ4 | 213273_at | BF112171 | 26011 | Hs.213087 |
| OGFR | 202841_x_at | NM_007346.2 | 11054 | Hs.67896 |
| OGFR | 210443_x_at | NM_007346.2 | 11054 | Hs.67896 |
| OGFR | 211512_s_at | NM_007346.2 | 11054 | Hs.67896 |
| OGFR | 211513_s_at | NM_007346.2 | 11054 | Hs.67896 |
| OGN | 218730_s_at | NM_024416.2 | 4969 | Hs.109439 |
| OGN | 222722_at | NM_024416.2 | 4969 | Hs.109439 |
| OKL38 | 219475_at | NM_013370 | 29948 | Hs.528383 |
| OLFM2 | 223601_at | NM_058164.1 | 93145 | Hs.169743 |
| OLFM3 | 1554524_a_at | NM_058170.1 | 118427 | Hs.484475 |
| OLFM3 | 1554526_at | NM_058170.1 | 118427 | Hs.484475 |
| OLFM4 | 212768_s_at | NM_006418.3 | 10562 | Hs.508113 |
| OLFML1 | 217525_at | NM_198474.2 | 283298 | Hs.503500 |
| OLFML2A | 213075_at | NM_182487.1 | 169611 | Hs.357004 |
| OLFML2B | 213125_at | NM_015441.1 | 25903 | Hs.507515 |
| OLFML3 | 218162_at | NM_020190.2 | 56944 | Hs.9315 |
| OLR1 | 210004_at | NM_002543.2 | 4973 | Hs.412484 |
| OMD | 205907_s_at | NM_005014.1 | 4958 | Hs.94070 |
| OMD | 205908_s_at | NM_005014.1 | 4958 | Hs.94070 |
| OMG | 238720_at | NM_002544 | 4974 | Hs.445237 |
| OMG | 207093_s_at | NM_002544.3 | 4974 | Hs.558363 |
| OPCML | 214111_at | NM_002545.3 | 4978 | Hs.4817 |
| OPCML | 206215_at | NM_002545.3 | 4978 | Hs.4817 |
| OPN1MW | 221327_s_at | NM_000513.1 | 2652 | Hs.247787 |
| OPN1SW | 208036_at | NM_001708.1 | 611 | Hs.550465 |
| OPN3 | 219032_x_at | NM_001030011.1 | 23596 | Hs.409081 |
| OPN3 | 224392_s_at | NM_001030011.1 | 23596 | Hs.409081 |
| OPN3 | 1565947_a_at | NM_014322 | 23596 | Hs.170129 |
| OPN3 | 1565949_x_at | NM_014322 | 23596 | Hs.170129 |
| OPN3 | 1566337_x_at | NM_014322 | 23596 | Hs.170129 |
| OPN4 | 234226_at | NM_001030015.1 | 94233 | Hs.283922 |
| OPN5 | 233657_at | NM_001030051.1 | 221391 | Hs.213717 |
| OPN5 | 1568914_at | NM_001030051.1 | 221391 | Hs.213717 |
| OPRD1 | 207792_at | NM_000911.3 | 4985 | Hs.372 |
| OPRK1 | 229944_at | NM_000912.3 | 4986 | Hs.106795 |
| OPRK1 | 207553_at | NM_000912.3 | 4986 | Hs.106795 |
| OPRL1 | 206563_s_at | NM_000913.3 | 4987 | Hs.2859 |
| OPRL1 | 206564_at | NM_000913.3 | 4987 | Hs.2859 |
| OPRM1 | 207989_at | NM_000914 | 4988 | Hs.2353 |
| OPRM1 | 207994_s_at | NM_000914.2 | 4988 | Hs.2353 |
| OPRM1 | 211359_s_at | NM_000914.2 | 4988 | Hs.2353 |
| OPRS1 | 201692_at | NM_005866.2 | 10280 | Hs.522087 |
| OPRS1 | 214484_s_at | NM_005866.2 | 10280 | Hs.522087 |
| OPTC | 223884_at | NM_014359.3 | 26254 | Hs.467538 |
| OR10A3 | 1567250_at | X64986 | 26496 | Hs.381322 |
| OR10A3 | 1567251_at | NM_001003745.1 | 26496 | Hs.381322 |
| OR10A4 | 1555655_at | NM_207186.1 | 283297 | Hs.448685 |
| OR10A5 | 1555651_at | NM_178168.1 | 144124 | Hs.447478 |
| OR10C1 | 221339_at | NM_013941.2 | 442194 | Hs.553838 |
| OR10D3P | 1567252_at | X64983 | 26497 | 538726 |
| OR10D3P | 1567253_at | X64983.1 | 26497 | Hs.538726 |
| OR10H1 | 208558_at | NM_013940.1 | 26539 | Hs.533925 |
| OR10H2 | 208543_at | NM_013939.1 | 26538 | Hs.247694 |
| OR10H3 | 208520_at | NM_013938.1 | 26532 | Hs.553524 |
| OR10J1 | 221346_at | NM_012351.1 | 26476 | Hs.532661 |
| OR11A1 | 221343_at | NM_013937.2 | 26531 | Hs.247861 |
| OR12D2 | 221344_at | NM_013936.2 | 26529 | Hs.247862 |
| OR12D3 | 221431_s_at | NM_030959.2 | 81797 | Hs.272280 |
| OR13C4 | 1567297_at | X64991 | 138804 | 553601 |
| OR1A1 | 221388_at | NM_014565.1 | 8383 | Hs.532688 |
| OR1A2 | 221445_at | NM_012352.1 | 26189 | Hs.532660 |
| OR1C1 | 1567054_at | X89674 | 26188 | Hs.381306 |
| OR1C1 | 1567055_at | NM_012353.1 | 26188 | Hs.381306 |
| OR1D2 | 221464_at | NM_002548.1 | 4991 | Hs.532771 |
| OR1D5 | 221341_s_at | NM_014566.1 | 8386 | Hs.553508 |
| OR1E1 | 214515_at | NM_003553 | 8387 | Hs.278485 |
| OR1E2 | 208587_s_at | NM_003554.1 | 8388 | Hs.533931 |
| OR1F1 | 221402_at | NM_012360.1 | 4992 | Hs.278464 |
| OR1F2 | 217308_at | AJ003145 | 26184 | Hs.247693 |
| OR1G1 | 221375_at | NM_003555.1 | 8390 | Hs.248183 |
| OR1I1 | 234403_at | NM_001004713.1 | 126370 | Hs.553578 |
| OR1J2 | 1567257_at | NM_054107.1 | 26740 | Hs.460145 |
| OR1J4 | 1567282_at | X64979 | 26219 | Hs.381307 |
| OR1J4 | 1567284_at | NM_001004452.1 | 26219 | Hs.528355 |
| OR1Q1 | 1567064_at | X89667 | 26729 | Hs.381330 |
| OR1Q1 | 1567065_at | NM_012364.1 | 158131 | Hs.534571 |
| OR2A4 | 233493_at | NM_030908.1 | 79541 | Hs.486488 |
| OR2A7 | 233492_s_at | NM_001005328.1 | 401427 | Hs.529671 |
| OR2B2 | 216408_at | NM_033057.1 | 81697 | Hs.553551 |
| OR2B3 | 234903_at | NM_001005226.1 | 442184 | Hs.553835 |
| OR2B6 | 216522_at | NM_012367.1 | 26212 | Hs.532145 |
| OR2C1 | 221460_at | NM_012368.1 | 4993 | Hs.258574 |
| OR2C3 | 1555018_at | NM_198074.3 | 81472 | Hs.332905 |
| OR2F1 | 208526_at | NM_012369.2 | 26211 | Hs.553522 |
| OR2F2 | 217302_at | NM_001004685.1 | 135948 | Hs.553596 |
| OR2F2 | 208525_s_at | NM_001004685.1 | 135948 | Hs.553596 |
| OR2H1 | 216817_s_at | NM_030883.3 | 26716 | Hs.434715 |
| OR2H1 | 1556986_at | NM_030883.3 | 26716 | Hs.434715 |
| OR2H1 | 1556987_s_at | NM_030883.3 | 26716 | Hs.434715 |
| OR2H1 | 1567656_at | NM_030883 | 26716 | Hs.434715 |
| OR2H1 | 1567657_at | NM_030883.3 | 26716 | Hs.434715 |
| OR2H2 | 217081_at | NM_007160.3 | 7932 | Hs.529493 |
| OR2H2 | 208573_s_at | NM_007160.3 | 7932 | Hs.529493 |
| OR2J2 | 216818_s_at | NM_030905.1 | 26707 | Hs.163518 |
| OR2J2 | 208508_s_at | NM_030905.1 | 26707 | Hs.163518 |
| OR2J3 | 217334_at | NM_001005216.1 | 442186 | Hs.553836 |
| OR2K2 | 1567272_at | X64977 | 26248 | Hs.381312 |
| OR2K2 | 1567273_at | NM_205859.1 | 26248 | Hs.381312 |
| OR2L13 | 1554706_at | NM_175911.2 | 284521 | Hs.546529 |
| OR2L2 | 1567237_at | X64978 | 26246 | 372936 |
| OR2L2 | 1567238_at | NM_001004686.2 | 26246 | Hs.372936 |
| OR2L2 | 1567240_x_at | NM_001004686.2 | 26246 | Hs.372936 |
| OR2M4 | 1566288_at | X89666 | 26245 | Hs.381309 |
| OR2M4 | 1566289_at | NM_017504.1 | 26245 | Hs.381309 |
| OR2S2 | 221409_at | NM_019897.1 | 56656 | Hs.553540 |
| OR2W1 | 221451_s_at | NM_030903.2 | 26692 | Hs.553526 |
| OR3A1 | 221353_at | NM_002550.1 | 4994 | Hs.555884 |
| OR3A2 | 221386_at | NM_002551.3 | 4995 | Hs.532772 |
| OR3A3 | 221426_s_at | NM_012373.1 | 8392 | Hs.532689 |
| OR4D1 | 1567068_at | X89670 | 26689 | Hs.381329 |
| OR4D1 | 1567069_at | NM_012374.1 | 26689 | Hs.531188 |
| OR4D2 | 1555377_at | NM_001004707.2 | 124538 | Hs.446879 |
| OR4F3 | 234369_at | NM_001005224.1 | 26683 | Hs.487233 |
| OR4N4 | 1560734_at | AK097598.1 | 283694 | Hs.525666 |
| OR4N4 | 1560735_s_at | NM_001005241.1 | 283694 | Hs.525666 |
| OR4N4 | 1564855_at | AK058056 | 283694 | Hs.386026 |
| OR4N4 | 1564856_s_at | AK097598.1 | 283694 | Hs.525666 |
| OR51B2 | 234486_at | NM_033180 | 79345 | Hs.283879 |
| OR51B4 | 234469_at | NM_033179.1 | 79339 | Hs.283880 |
| OR51B5 | 234775_at | NM_001005567.1 | 282763 | Hs.382831 |
| OR51B5 | 1570516_s_at | NM_001005567.1 | 282763 | Hs.382831 |
| OR51B6 | 234526_at | NM_001004750.1 | 390058 | Hs.553730 |
| OR51E1 | 229768_at | AL833127.1 | 143503 | Hs.470038 |
| OR51E2 | 221424_s_at | NM_030774.2 | 81285 | Hs.501758 |
| OR51E2 | 232482_at | NM_030774.2 | 81285 | Hs.501758 |
| OR51E2 | 236121_at | NM_030774.2 | 81285 | Hs.501758 |
| OR51I1 | 234626_at | NM_001005288.1 | 390063 | Hs.553732 |
| OR51I2 | 234521_at | NM_001004754.1 | 390064 | Hs.553733 |
| OR51M1 | 234777_at | NM_001004756.1 | 390059 | Hs.553731 |
| OR52A1 | 221329_at | NM_012375.1 | 23538 | Hs.553520 |
| OR52D1 | 234617_at | NM_001005163.1 | 390066 | Hs.553734 |
| OR5H1 | 1567246_at | X64988 | 26341 | Hs.381318 |
| OR5H1 | 1567247_at | NM_001005338.1 | 26341 | Hs.537383 |
| OR5I1 | 208521_at | NM_006637.1 | 10798 | Hs.533706 |
| OR5J2 | 1567244_at | X64974 | 282775 | 537145 |
| OR5J2 | 1567245_at | NM_001005492.1 | 282775 | Hs.537145 |
| OR5K1 | 1567287_at | X64984 | 26339 | 621533 |
| OR5K1 | 1567288_at | NM_001004736.1 | 26339 | Hs.531371 |
| OR5L2 | 1567285_at | X64990 | 26338 | Hs.381315 |
| OR5L2 | 1567286_at | NM_001004739.1 | 26338 | Hs.528356 |
| OR5P2 | 1552991_at | NM_153444.1 | 120065 | Hs.351824 |
| OR5P3 | 1553078_at | NM_153445.1 | 120066 | Hs.351825 |
| OR5U1 | 234545_at | NM_030946.1 | 442191 | Hs.553837 |
| OR5V1 | 234840_s_at | NM_030876.4 | 81696 | Hs.274460 |
| OR5V1 | 208098_at | NM_030876 | 81696 | Hs.274460 |
| OR6A1 | 221465_at | NM_003696 | 8590 | Hs.248186 |
| OR6B1 | 234363_at | NM_001005281.1 | 135946 | Hs.553595 |
| OR6C4 | 224343_x_at | L42788 | 341418 | 495021 |
| OR7A10 | 217316_at | NM_001005190.1 | 390892 | Hs.553776 |
| OR7A17 | 208509_s_at | NM_030901.1 | 26333 | Hs.247717 |
| OR7A5 | 208285_at | NM_017506.1 | 26659 | Hs.137573 |
| OR7C1 | 216690_at | NM_198944.1 | 26664 | Hs.466053 |
| OR7C2 | 208507_at | NM_012377.1 | 26658 | Hs.553525 |
| OR7D2 | 1562337_at | NM_175883.1 | 162998 | Hs.531755 |
| OR7E24P | 215463_at | XR_000082 | 26648 | Hs.129832 |
| OR8B8 | 1555212_at | NM_012378.1 | 26493 | Hs.351822 |
| OR8D1 | 1555661_at | NM_001002917.1 | 283159 | Hs.504211 |
| OR8D2 | 234766_at | NM_001002918.1 | 283160 | Hs.504212 |
| OR8G1 | 1567059_at | X89672 | 26494 | Hs.381320 |
| OR8G1 | 1567060_at | NM_001002905.1 | 26494 | Hs.448172 |
| OR8G2 | 1567056_at | X89669 | 26492 | Hs.381319 |
| OR8G2 | 1567058_at | NM_001007249.1 | 26492 | Hs.381319 |
| OR9A1P | 1567248_at | X64982 | 26495 | 544835 |
| OR9A1P | 1567249_at | X64982.1 | 26495 | Hs.544835 |
| ORAI3 | 221864_at | NM_152288.1 | 93129 | Hs.460617 |
| ORM1 | 205040_at | NM_000607.1 | 5004 | Hs.522356 |
| ORM1 | 205041_s_at | NM_000607.1 | 5004 | Hs.522356 |
| OSAP | 223734_at | NM_032623.2 | 84709 | Hs.154140 |
| OSBPL10 | 216755_at | AK024945.1 | 114884 | Hs.150122 |
| OSBPL10 | 217017_at | AK024945 | 114884 | 150122 |
| OSBPL10 | 219073_s_at | NM_017784.3 | 114884 | Hs.150122 |
| OSBPL10 | 222818_at | NM_017784 | 114884 | Hs.368238 |
| OSBPL10 | 231656_x_at | NM_017784 | 114884 | Hs.368238 |
| OSCAR | 1554503_a_at | NM_130771.2 | 126014 | Hs.347655 |
| OSM | 214637_at | NM_020530 | 5008 | Hs.248156 |
| OSM | 230170_at | NM_020530.3 | 5008 | Hs.248156 |
| OSMR | 1554008_at | NM_003999 | 9180 | Hs.120658 |
| OSMR | 205729_at | NM_003999.1 | 9180 | Hs.120658 |
| OSTM1 | 218196_at | NM_014028.3 | 28962 | Hs.226780 |
| OSTM1 | 235197_s_at | NM_014028.3 | 28962 | Hs.226780 |
| OSTM1 | 235198_at | NM_014028.3 | 28962 | Hs.226780 |
| OTOA | 1553432_s_at | NM_170664.1 | 146183 | Hs.408336 |
| OTOF | 220492_s_at | NM_004802.2 | 9381 | Hs.91608 |
| OTOF | 1555251_a_at | NM_004802.2 | 9381 | Hs.91608 |
| OTOP2 | 234271_at | NM_178160 | 92736 | Hs.352515 |
| OTOP2 | 234551_at | NM_178160 | 92736 | Hs.352515 |
| OTOR | 221209_s_at | NM_020157.2 | 56914 | Hs.41119 |
| OTOS | 242883_at | NM_148961.3 | 150677 | Hs.148586 |
| OXER1 | 1553222_at | NM_148962.3 | 165140 | Hs.168439 |
| OXGR1 | 1553319_at | NM_080818.3 | 27199 | Hs.352218 |
| OXT | 207576_x_at | NM_000915.2 | 5020 | Hs.113216 |
| OXTR | 206825_at | NM_000916.3 | 5021 | Hs.2820 |
| P2RX1 | 210401_at | NM_002558.2 | 5023 | Hs.41735 |
| P2RX2 | 221356_x_at | NM_012226.3 | 22953 | Hs.258580 |
| P2RX2 | 221372_s_at | NM_012226.3 | 22953 | Hs.258580 |
| P2RX2 | 224069_x_at | NM_012226.3 | 22953 | Hs.258580 |
| P2RX2 | 224557_x_at | NM_012226.3 | 22953 | Hs.258580 |
| P2RX3 | 208338_at | NM_002559.2 | 5024 | Hs.146738 |
| P2RX4 | 204088_at | NM_002560.2 | 5025 | Hs.321709 |
| P2RX5 | 210448_s_at | NM_002561.2 | 5026 | Hs.408615 |
| P2RX7 | 207091_at | NM_002562.4 | 5027 | Hs.507102 |
| P2RXL1 | 206880_at | NM_005446.2 | 9127 | Hs.113275 |
| P2RXL1 | 211147_s_at | NM_005446.2 | 9127 | Hs.113275 |
| P2RY1 | 207455_at | NM_002563.2 | 5028 | Hs.2411 |
| P2RY10 | 214615_at | NM_014499.2 | 27334 | Hs.296433 |
| P2RY10 | 1553856_s_at | NM_014499.2 | 27334 | Hs.296433 |
| P2RY11 | 214546_s_at | NM_002566.4 | 5032 | Hs.166168 |
| P2RY12 | 224102_at | NM_176876.1 | 64805 | Hs.558534 |
| P2RY12 | 235885_at | NM_022788 | 64805 | Hs.444983 |
| P2RY13 | 220005_at | NM_176894.1 | 53829 | Hs.546396 |
| P2RY14 | 206637_at | NM_014879.2 | 9934 | Hs.2465 |
| P2RY2 | 206277_at | NM_002564.2 | 5029 | Hs.339 |
| P2RY4 | 221466_at | NM_002565.3 | 5030 | Hs.533929 |
| P2RY5 | 218589_at | NM_005767.3 | 10161 | Hs.123464 |
| P2RY5 | 1557763_at | NM_005767 | 10161 | Hs.123464 |
| P2RY6 | 208373_s_at | NM_176796.1 | 5031 | Hs.16362 |
| P2RY8 | 229686_at | NM_178129.3 | 286530 | Hs.111377 |
| P4HB | 1564494_s_at | NM_000918.2 | 5034 | Hs.464336 |
| P4HB | 200654_at | NM_000918 | 5034 | Hs.464336 |
| P4HB | 200656_s_at | NM_000918.2 | 5034 | Hs.464336 |
| PAEP | 229410_at | NM_024881 | 5047 | Hs.134074 |
| PAEP | 206859_s_at | NM_001018049.1 | 5047 | Hs.134074 |
| PAG1 | 225622_at | NM_018440.3 | 55824 | Hs.266175 |
| PAG1 | 225626_at | NM_018440.3 | 55824 | Hs.266175 |
| PAG1 | 227354_at | NM_018440.3 | 55824 | Hs.266175 |
| PALM | 203859_s_at | NM_002579.2 | 5064 | Hs.78482 |
| PAM | 214620_x_at | NM_000919 | 5066 | Hs.352733 |
| PAM | 202336_s_at | NM_000919.2 | 5066 | Hs.369430 |
| PAM | 212958_x_at | NM_000919 | 5066 | Hs.352733 |
| PANX1 | 227107_at | NM_015368 | 24145 | Hs.32163 |
| PANX1 | 235295_at | NM_015368 | 24145 | Hs.32163 |
| PANX1 | 236372_at | NM_015368 | 24145 | Hs.32163 |
| PANX1 | 204715_at | NM_015368.3 | 24145 | Hs.503584 |
| PANX2 | 239067_s_at | NM_052839.2 | 56666 | Hs.440092 |
| PANX2 | 244471_x_at | NM_052839 | 56666 | Hs.440092 |
| PANX2 | 1552944_a_at | NM_052839.2 | 56666 | Hs.440092 |
| PANX3 | 240955_at | NM_052959.2 | 116337 | Hs.99235 |
| PAPLN | 226435_at | NM_173462.2 | 89932 | Hs.458428 |
| PAPLN | 233896_s_at | NM_173462.2 | 89932 | Hs.458428 |
| PAPLN | 236572_at | NM_173462 | 89932 | Hs.301152 |
| PAPPA | 224940_s_at | NM_002581.3 | 5069 | Hs.494928 |
| PAPPA | 224941_at | NM_002581.3 | 5069 | Hs.494928 |
| PAPPA | 224942_at | NM_002581.3 | 5069 | Hs.494928 |
| PAPPA | 228128_x_at | NM_002581.3 | 5069 | Hs.494928 |
| PAPPA | 232748_at | AK024330.1 | 5069 | Hs.494928 |
| PAPPA | 1558607_at | AF085834.1 | 5069 | Hs.494928 |
| PAPPA | 1558608_a_at | AF085834.1 | 5069 | Hs.494928 |
| PAPPA | 1559400_s_at | NM_002581.3 | 5069 | Hs.494928 |
| PAPPA | 1559928_at | AF085855.1 | 5069 | Hs.494928 |
| PAPPA | 201981_at | NM_002581.3 | 5069 | Hs.494928 |
| PAPPA | 201982_s_at | NM_002581.3 | 5069 | Hs.494928 |
| PAPPA2 | 228237_at | NM_020318.1 | 60676 | Hs.187284 |
| PAPPA2 | 211918_x_at | NM_020318.1 | 60676 | Hs.187284 |
| PAQR3 | 213372_at | NM_001040202.1 | 152559 | Hs.558657 |
| PAQR3 | 1557576_at | BC031256.1 | 152559 | Hs.558657 |
| PAQR4 | 212858_at | NM_152341.2 | 124222 | Hs.351474 |
| PAQR5 | 220333_at | NM_017705.2 | 54852 | Hs.147229 |
| PAQR6 | 219236_at | NM_024897.2 | 79957 | Hs.235873 |
| PAQR7 | 242123_at | NM_178422.4 | 164091 | Hs.523652 |
| PAQR8 | 226423_at | NM_133367.2 | 85315 | Hs.239388 |
| PAQR8 | 227626_at | NM_133367.2 | 85315 | Hs.239388 |
| PAQR9 | 1558322_a_at | NM_198504.1 | 344838 | Hs.563105 |
| PAQR9 | 1560467_at | AK093381.1 | 344838 | Hs.563105 |
| PATE | 231279_at | NM_138294.2 | 160065 | Hs.148565 |
| PATE | 243468_at | NM_138294.2 | 160065 | Hs.148565 |
| PCDH1 | 215277_at | NM_032420.2 | 5097 | Hs.79769 |
| PCDH1 | 203918_at | NM_002587.3 | 5097 | Hs.79769 |
| PCDH10 | 228635_at | NM_032961.1 | 57575 | Hs.192859 |
| PCDH10 | 1552925_at | NM_020815.1 | 57575 | Hs.192859 |
| PCDH11X | 217049_x_at | NM_032967.1 | 27328 | Hs.496456 |
| PCDH11X | 1557880_at | NM_014522.1 | 27328 | Hs.496456 |
| PCDH11X | 208366_at | NM_014522.1 | 27328 | Hs.496456 |
| PCDH11X | 210292_s_at | NM_032968.2 | 27328 | Hs.496456 |
| PCDH11Y | 241772_at | NM_032971 | 83259 | 567884 |
| PCDH11Y | 211227_s_at | NM_032971.1 | 83259 | Hs.496456 |
| PCDH12 | 219656_at | NM_016580.2 | 51294 | Hs.439474 |
| PCDH15 | 1553344_at | NM_033056.2 | 65217 | Hs.232819 |
| PCDH15 | 1560330_at | NM_033056 | 65217 | Hs.232819 |
| PCDH17 | 205656_at | NM_001040429.1 | 27253 | Hs.106511 |
| PCDH18 | 225975_at | NM_019035.2 | 54510 | Hs.518989 |
| PCDH18 | 225977_at | NM_019035.2 | 54510 | Hs.518989 |
| PCDH19 | 227282_at | NM_020766.1 | 57526 | Hs.4993 |
| PCDH20 | 232054_at | NM_022843.2 | 64881 | Hs.391781 |
| PCDH20 | 1560566_at | NM_022843 | 64881 | Hs.391781 |
| PCDH21 | 213369_at | NM_033100.1 | 92211 | Hs.137556 |
| PCDH21 | 1555019_at | BC038799.1 | 92211 | Hs.137556 |
| PCDH7 | 228640_at | NM_002589 | 5099 | Hs.443020 |
| PCDH7 | 205534_at | NM_002589.2 | 5099 | Hs.479439 |
| PCDH7 | 205535_s_at | NM_002589.2 | 5099 | Hs.479439 |
| PCDH7 | 210273_at | NM_032457.1 | 5099 | Hs.479439 |
| PCDH7 | 210941_at | NM_032456.1 | 5099 | Hs.479439 |
| PCDH8 | 206935_at | NM_032949.1 | 5100 | Hs.19492 |
| PCDH9 | 216456_at | NM_020403 | 5101 | Hs.407643 |
| PCDH9 | 216707_at | NM_020403 | 5101 | Hs.407643 |
| PCDH9 | 219737_s_at | NM_203487.1 | 5101 | Hs.407643 |
| PCDH9 | 219738_s_at | NM_020403.3 | 5101 | Hs.407643 |
| PCDH9 | 1563611_at | NM_020403 | 5101 | Hs.407643 |
| PCDH9 | 1565601_at | NM_020403 | 5101 | Hs.407643 |
| PCDHA1 | 223435_s_at | NM_018900.2 | 56147 | Hs.199343 |
| PCDHA10 | 211867_s_at | NM_018901.2 | 56139 | Hs.199343 |
| PCDHA2 | 210572_at | NM_031496.1 | 56146 | Hs.199343 |
| PCDHA2 | 211365_s_at | NM_018905.2 | 56146 | Hs.199343 |
| PCDHA3 | 211870_s_at | NM_018906.2 | 56145 | Hs.199343 |
| PCDHA5 | 211838_x_at | NM_018908.2 | 56143 | Hs.199343 |
| PCDHA6 | 211811_s_at | NM_018909.2 | 56142 | Hs.199343 |
| PCDHA9 | 208205_at | NM_014005.3 | 9752 | Hs.199343 |
| PCDHAC1 | 1553235_at | NM_031882.2 | 56135 | Hs.199343 |
| PCDHAC1 | 1553237_x_at | NM_031882.2 | 56135 | Hs.199343 |
| PCDHAC1 | 210674_s_at | NM_018898.3 | 56135 | Hs.199343 |
| PCDHAC2 | 224212_s_at | NM_018899.4 | 56134 | Hs.199343 |
| PCDHAC2 | 224539_s_at | NM_018899.4 | 56134 | Hs.199343 |
| PCDHB1 | 221303_at | NM_013340.2 | 29930 | Hs.278950 |
| PCDHB10 | 223854_at | NM_018930.3 | 56126 | Hs.558517 |
| PCDHB11 | 208504_x_at | NM_018931.2 | 56125 | Hs.283084 |
| PCDHB12 | 221408_x_at | NM_018932.3 | 56124 | Hs.429820 |
| PCDHB13 | 221450_x_at | NM_018933.2 | 56123 | Hs.283803 |
| PCDHB13 | 232415_at | NM_018933.2 | 56123 | Hs.283803 |
| PCDHB14 | 231726_at | NM_018934.2 | 56122 | Hs.283678 |
| PCDHB15 | 231789_at | NM_018935 | 56121 | Hs.25748 |
| PCDHB15 | 240445_at | NM_018935 | 56121 | Hs.25748 |
| PCDHB16 | 232099_at | NM_020957 | 57717 | Hs.147674 |
| PCDHB18 | 234479_at | NR_001281 | 54660 | Hs.284306 |
| PCDHB18 | 234724_x_at | NR_001281 | 54660 | Hs.284306 |
| PCDHB2 | 221410_x_at | NM_018936.2 | 56133 | Hs.533023 |
| PCDHB2 | 231725_at | NM_018936.2 | 56133 | Hs.533023 |
| PCDHB3 | 244183_x_at | NM_018937.2 | 56132 | Hs.508987 |
| PCDHB4 | 231750_at | NM_018938.2 | 56131 | Hs.283661 |
| PCDHB4 | 240317_at | NM_018938.2 | 56131 | Hs.283661 |
| PCDHB5 | 223629_at | NM_015669.2 | 26167 | Hs.119693 |
| PCDHB6 | 221317_x_at | NM_018939.2 | 56130 | Hs.283085 |
| PCDHB6 | 239443_at | NM_018939.2 | 56130 | Hs.283085 |
| PCDHB7 | 231738_at | NM_018940.2 | 56129 | Hs.203830 |
| PCDHB8 | 221319_at | NM_019120.2 | 56128 | Hs.283803 |
| PCDHB9 | 223927_at | NM_019119.3 | 56127 | Hs.231119 |
| PCDHGA1 | 211880_x_at | NM_018912.2 | 56114 | Hs.368160 |
| PCDHGA10 | 211875_x_at | NM_018913.2 | 56106 | Hs.368160 |
| PCDHGA11 | 211877_s_at | NM_018914.2 | 56105 | Hs.368160 |
| PCDHGA12 | 211876_x_at | NM_003735.2 | 26025 | Hs.368160 |
| PCDHGA2 | 216352_x_at | NM_018915.2 | 56113 | Hs.368160 |
| PCDHGA3 | 211879_x_at | NM_018916.3 | 56112 | Hs.368160 |
| PCDHGA8 | 210368_at | AB002325.1 | 9708 | Hs.368160 |
| PCDHGA9 | 211873_s_at | NM_018921.2 | 56107 | Hs.368160 |
| PCDHGB5 | 211807_x_at | NM_032099.1 | 56101 | Hs.368160 |
| PCDHGB6 | 221682_s_at | NM_018926.2 | 56100 | Hs.368160 |
| PCDHGB7 | 1552661_at | NM_032101.1 | 56099 | Hs.368160 |
| PCDHGB7 | 1552662_a_at | NM_018927.2 | 56099 | Hs.368160 |
| PCDHGC3 | 214563_at | NM_032402.1 | 5098 | Hs.368160 |
| PCDHGC3 | 214564_s_at | NM_002588.2 | 5098 | Hs.368160 |
| PCDHGC3 | 215836_s_at | NM_002588.2 | 5098 | Hs.368160 |
| PCDHGC3 | 234029_at | NM_002588 | 5098 | Hs.283794 |
| PCDHGC3 | 1552735_at | NM_002588 | 5098 | Hs.283794 |
| PCDHGC3 | 205717_x_at | NM_002588.2 | 5098 | Hs.368160 |
| PCDHGC3 | 209079_x_at | NM_002588.2 | 5098 | Hs.368160 |
| PCDHGC3 | 211066_x_at | NM_002588.2 | 5098 | Hs.368160 |
| PCDHGC4 | 231753_s_at | NM_018928.2 | 56098 | Hs.368160 |
| PCDHGC4 | 231754_at | NM_032406.1 | 56098 | Hs.368160 |
| PCDHGC5 | 224536_s_at | NM_032407.1 | 56097 | Hs.368160 |
| PCDHGC5 | 224537_at | NM_032407.1 | 56097 | Hs.368160 |
| PCLKC | 220186_s_at | NM_017675.3 | 54825 | Hs.4205 |
| PCLO | 213558_at | AB011131 | 27445 | Hs.12376 |
| PCLO | 210650_s_at | BC001304 | 27445 | Hs.12376 |
| PCM1 | 214118_x_at | NM_006197 | 5108 | Hs.348501 |
| PCM1 | 214937_x_at | NM_006197.2 | 5108 | Hs.491148 |
| PCM1 | 228905_at | NM_006197 | 5108 | Hs.348501 |
| PCM1 | 202174_s_at | NM_006197.2 | 5108 | Hs.491148 |
| PCM1 | 209996_x_at | NM_006197.2 | 5108 | Hs.491148 |
| PCM1 | 209997_x_at | NM_006197.2 | 5108 | Hs.491148 |
| PCNX | 215175_at | NM_014982 | 22990 | Hs.158722 |
| PCNX | 238792_at | NM_014982 | 22990 | Hs.55947 |
| PCNX | 239100_x_at | NM_014982 | 22990 | Hs.55947 |
| PCNX | 213159_at | NM_014982.1 | 22990 | Hs.158722 |
| PCNX | 213173_at | NM_014982 | 22990 | Hs.55947 |
| PCNXL2 | 220461_at | AK021445.1 | 80003 | Hs.370605 |
| PCNXL2 | 39650_s_at | NM_014801.2 | 80003 | Hs.370605 |
| PCNXL2 | 1554256_a_at | NM_014801.2 | 80003 | Hs.370605 |
| PCNXL2 | 205689_at | AB007895.1 | 80003 | Hs.370605 |
| PCNXL3 | 227858_at | XM_945229.1 | 399909 | Hs.380801 |
| PCOLCE | 202465_at | NM_002593.2 | 5118 | Hs.202097 |
| PCOLCE2 | 219295_s_at | NM_013363.2 | 26577 | Hs.8944 |
| PCOLN3 | 201933_at | NM_002768 | 5119 | Hs.461777 |
| PCSK1N | 218952_at | NM_013271.2 | 27344 | Hs.522640 |
| PCSK4 | 230901_x_at | NM_017573.3 | 54760 | Hs.46884 |
| PCSK5 | 213652_at | NM_006200 | 5125 | Hs.288931 |
| PCSK5 | 205559_s_at | NM_006200.2 | 5125 | Hs.368542 |
| PCSK5 | 205560_at | NM_006200.2 | 5125 | Hs.368542 |
| PCSK6 | 1553531_at | NM_138322.2 | 5046 | Hs.498494 |
| PCSK6 | 207414_s_at | NM_002570.3 | 5046 | Hs.498494 |
| PCSK6 | 210553_x_at | NM_138325.2 | 5046 | Hs.498494 |
| PCSK6 | 211262_at | NM_138323.1 | 5046 | Hs.498494 |
| PCSK6 | 211263_s_at | NM_138321.1 | 5046 | Hs.498494 |
| PCSK9 | 227759_at | NM_174936.2 | 255738 | Hs.18844 |
| PCYOX1L | 218953_s_at | NM_024028.2 | 78991 | Hs.483796 |
| PDCD1 | 207634_at | NM_005018.1 | 5133 | Hs.158297 |
| PDCD1LG2 | 220049_s_at | NM_025239.2 | 80380 | Hs.532279 |
| PDCD1LG2 | 224399_at | NM_025239.2 | 80380 | Hs.532279 |
| PDDC1 | 227968_at | NM_182612.2 | 347862 | Hs.218362 |
| PDE3B | 214582_at | NM_000753 | 5140 | Hs.337616 |
| PDE3B | 222317_at | NM_000753 | 5140 | Hs.337616 |
| PDE3B | 208591_s_at | NM_000922.2 | 5140 | Hs.445711 |
| PDGFA | 216867_s_at | NM_002607.4 | 5154 | Hs.376032 |
| PDGFA | 229830_at | NM_002607 | 5154 | Hs.376032 |
| PDGFA | 1564472_at | NM_002607 | 5154 | Hs.376032 |
| PDGFA | 205463_s_at | NM_002607.4 | 5154 | Hs.376032 |
| PDGFB | 216061_x_at | NM_033016.1 | 5155 | Hs.1976 |
| PDGFB | 217112_at | M12783.1 | 5155 | Hs.1976 |
| PDGFB | 204200_s_at | NM_002608.1 | 5155 | Hs.1976 |
| PDGFC | 218718_at | NM_016205.1 | 56034 | Hs.148162 |
| PDGFC | 222719_s_at | NM_016205.1 | 56034 | Hs.148162 |
| PDGFD | 219304_s_at | NM_033135.3 | 80310 | Hs.352298 |
| PDGFD | 222860_s_at | NM_033135.3 | 80310 | Hs.352298 |
| PDGFRA | 215305_at | NM_006206 | 5156 | Hs.74615 |
| PDGFRA | 1554828_at | NM_006206 | 5156 | Hs.74615 |
| PDGFRA | 203131_at | NM_006206.3 | 5156 | Hs.74615 |
| PDGFRA | 211533_at | NM_006206 | 5156 | Hs.74615 |
| PDGFRB | 202273_at | NM_002609.3 | 5159 | Hs.509067 |
| PDGFRL | 205226_at | NM_006207.1 | 5157 | Hs.458573 |
| PDPN | 221898_at | NM_001006624.1 | 10630 | Hs.468675 |
| PDPN | 226658_at | NM_001006624.1 | 10630 | Hs.468675 |
| PDPN | 204879_at | AJ225022.1 | 10630 | Hs.468675 |
| PDPN | 208233_at | AF030427.1 | 10630 | Hs.468675 |
| PDYN | 206803_at | NM_024411.2 | 5173 | Hs.22584 |
| PDZK11 | 223037_at | NM_016484.3 | 51248 | Hs.11042 |
| PDZK1IP1 | 219630_at | NM_005764.3 | 10158 | Hs.431099 |
| PDZK1IP1 | 1553589_a_at | NM_005764.3 | 10158 | Hs.431099 |
| PDZK3 | 209493_at | NM_178140.2 | 23037 | Hs.481819 |
| PDZK3 | 233025_at | NM_015022 | 23037 | Hs.481819 |
| PDZK3 | 233026_s_at | NM_178140.2 | 23037 | Hs.481819 |
| PECAM1 | 1559921_at | NM_000442 | 5175 | Hs.78146 |
| PECAM1 | 208981_at | NM_000442 | 5175 | Hs.514412 |
| PECAM1 | 208982_at | NM_000442 | 5175 | Hs.514412 |
| PECAM1 | 208983_s_at | NM_000442 | 5175 | Hs.514412 |
| PENK | 213791_at | NM_006211.2 | 5179 | Hs.339831 |
| PENK | 1560826_at | NM_006211 | 5179 | Hs.339831 |
| PERP | 217744_s_at | NM_022121.2 | 64065 | Hs.520421 |
| PERP | 222392_x_at | NM_022121.2 | 64065 | Hs.520421 |
| PERP | 236009_at | AI767250 | 64065 | Hs.201446 |
| PF4 | 206390_x_at | NM_002619.1 | 5196 | Hs.81564 |
| PF4V1 | 207815_at | NM_002620.2 | 5197 | Hs.72933 |
| PFC | 206380_s_at | NM_002621.1 | 5199 | Hs.53155 |
| PGA5 | 213265_at | NM_014224.1 | 5222 | Hs.432854 |
| PGBD5 | 219225_at | NM_024554.2 | 79605 | Hs.520463 |
| PGC | 1555236_a_at | NM_002630.1 | 5225 | Hs.1867 |
| PGC | 205261_at | NM_002630.1 | 5225 | Hs.1867 |
| PGF | 215179_x_at | NM_002632 | 5228 | Hs.252820 |
| PGF | 209652_s_at | NM_002632.4 | 5228 | Hs.252820 |
| PGLYRP1 | 207384_at | NM_005091.1 | 8993 | Hs.137583 |
| PGLYRP2 | 242817_at | NM_052890.2 | 114770 | Hs.282244 |
| PGRMC1 | 201120_s_at | NM_006667.2 | 10857 | Hs.90061 |
| PGRMC1 | 201121_s_at | NM_006667.2 | 10857 | Hs.90061 |
| PGRMC2 | 213227_at | NM_006320 | 10424 | Hs.507910 |
| PGRMC2 | 201701_s_at | NM_006320.1 | 10424 | Hs.507910 |
| PHB | 200658_s_at | NM_002634.2 | 5245 | Hs.514303 |
| PHB | 200659_s_at | NM_002634.2 | 5245 | Hs.514303 |
| PHEX | 210617_at | NM_000444.3 | 5251 | Hs.495834 |
| PHGDHL1 | 224298_s_at | NM_177967.2 | 337867 | Hs.567334 |
| PI15 | 229947_at | NM_015886.3 | 51050 | Hs.98558 |
| PI15 | 207938_at | NM_015886.3 | 51050 | Hs.98558 |
| PI16 | 228312_at | NM_153370.2 | 221476 | Hs.25391 |
| PI3 | 41469_at | NM_002638.2 | 5266 | Hs.112341 |
| PI3 | 203691_at | NM_002638.2 | 5266 | Hs.112341 |
| PI4K2A | 215134_at | NM_018425.2 | 55361 | Hs.25300 |
| PIGK | 227639_at | NM_005482 | 10026 | Hs.293653 |
| PIGK | 1555394_at | BC026186.1 | 10026 | Hs.293653 |
| PIGK | 209707_at | NM_005482 | 10026 | Hs.293653 |
| PIGP | 221689_s_at | NM_153681.2 | 51227 | Hs.408790 |
| PIGQ | 1555359_at | NM_004204 | 9091 | Hs.18079 |
| PIGQ | 204144_s_at | NM_004204.2 | 9091 | Hs.18079 |
| PIGR | 226147_s_at | NM_002644.2 | 5284 | Hs.497589 |
| PIGR | 229659_s_at | NM_002644 | 5284 | Hs.205126 |
| PIGR | 204213_at | NM_002644.2 | 5284 | Hs.497589 |
| PIK3C2B | 204484_at | NM_002646.2 | 5287 | Hs.497487 |
| PIK3IP1 | 221756_at | NM_052880.3 | 113791 | Hs.26670 |
| PIK3IP1 | 221757_at | NM_052880.3 | 113791 | Hs.26670 |
| PIK3IP1 | 1555632_at | AF528079.1 | 113791 | Hs.26670 |
| PILRA | 219788_at | NM_013439.2 | 29992 | Hs.444407 |
| PILRA | 222218_s_at | NM_013439.2 | 29992 | Hs.444407 |
| PILRB | 220954_s_at | NM_175047.2 | 29990 | Hs.530084 |
| PILRB | 225321_s_at | NM_013440.3 | 29990 | Hs.530084 |
| PIP | 206509_at | NM_002652.2 | 5304 | Hs.99949 |
| PITPNM2 | 220719_at | AK023831.1 | 80079 | Hs.512749 |
| PITPNM2 | 232950_s_at | NM_020845.2 | 57605 | Hs.272759 |
| PITPNM2 | 1552923_a_at | NM_020845.2 | 57605 | Hs.272759 |
| PITPNM2 | 1552924_a_at | NM_020845.2 | 57605 | Hs.272759 |
| PJA2 | 201133_s_at | NM_014819.2 | 9867 | Hs.483036 |
| PKD1 | 214682_at | XM_932304.1 | 339047 | Hs.513391 |
| PKD1 | 216949_s_at | NM_000296.2 | 5310 | Hs.527862 |
| PKD1 | 241090_at | NM_000296 | 5310 | Hs.75813 |
| PKD1 | 202327_s_at | NM_001009944.1 | 5310 | Hs.527862 |
| PKD1 | 202328_s_at | NM_001009944.1 | 5310 | Hs.527862 |
| PKD1L1 | 1553004_at | NM_138295.2 | 168507 | Hs.195979 |
| PKD1L1 | 1563465_at | NM_138295 | 168507 | Hs.408195 |
| PKD1L2 | 244444_at | NM_052892.2 | 114780 | Hs.413525 |
| PKD1L2 | 1554371_at | NM_052892 | 114780 | Hs.413525 |
| PKD1L2 | 1559261_a_at | NM_052892.2 | 114780 | Hs.413525 |
| PKD1L2 | 1561849_at | NM_052892 | 114780 | Hs.413525 |
| PKD2 | 203688_at | NM_000297.2 | 5311 | Hs.181272 |
| PKD2L1 | 221061_at | NM_016112.2 | 9033 | Hs.159241 |
| PKD2L2 | 221118_at | NM_014386.1 | 27039 | Hs.310431 |
| PKD2L2 | 224226_at | NM_014386 | 27039 | Hs.310431 |
| PKDREJ | 220548_at | NM_006071.1 | 10343 | Hs.241383 |
| PKHD1 | 241694_at | NM_138694.2 | 5314 | Hs.446118 |
| PKHD1 | 244410_at | NM_138694.2 | 5314 | Hs.446118 |
| PKHD1 | 1553003_at | NM_170724.1 | 5314 | Hs.446118 |
| PKHD1L1 | 230673_at | NM_177531.3 | 93035 | Hs.170128 |
| PKP2 | 214154_s_at | NM_004572.2 | 5318 | Hs.164384 |
| PKP2 | 207717_s_at | NM_001005242.1 | 5318 | Hs.164384 |
| PKP4 | 214874_at | NM_003628 | 8502 | Hs.277132 |
| PKP4 | 201927_s_at | NM_001005476.1 | 8502 | Hs.407580 |
| PKP4 | 201928_at | NM_001005476.1 | 8502 | Hs.407580 |
| PKP4 | 201929_s_at | NM_001005476.1 | 8502 | Hs.407580 |
| PLA1A | 219584_at | NM_015900.1 | 51365 | Hs.437451 |
| PLA2G10 | 207222_at | NM_003561.1 | 8399 | Hs.144442 |
| PLA2G12A | 221027_s_at | NM_030821.3 | 81579 | Hs.480519 |
| PLA2G12A | 223373_s_at | NM_030821.3 | 81579 | Hs.480519 |
| PLA2G12B | 224411_at | NM_032562.2 | 84647 | Hs.333175 |
| PLA2G12B | 231009_at | NM_032562.2 | 84647 | Hs.333175 |
| PLA2G1B | 206311_s_at | NM_000928.2 | 5319 | Hs.992 |
| PLA2G2D | 220423_at | NM_012400.2 | 26279 | Hs.189507 |
| PLA2G2E | 221389_at | NM_014589.1 | 30814 | Hs.272372 |
| PLA2G2F | 221416_at | NM_022819.2 | 64600 | Hs.302034 |
| PLA2G3 | 220780_at | NM_015715.2 | 50487 | Hs.149623 |
| PLA2G4A | 210145_at | NM_024420.1 | 5321 | Hs.497200 |
| PLA2G5 | 215870_s_at | NM_000929.1 | 5322 | Hs.319438 |
| PLA2G5 | 215871_at | NM_000929 | 5322 | Hs.319438 |
| PLA2G5 | 206178_at | NM_000929.1 | 5322 | Hs.319438 |
| PLA2G7 | 206214_at | NM_005084.2 | 7941 | Hs.554780 |
| PLA2R1 | 235746_s_at | NM_007366 | 22925 | Hs.410477 |
| PLA2R1 | 240039_at | NM_007366 | 22925 | Hs.410477 |
| PLA2R1 | 207415_at | NM_001007267.1 | 22925 | Hs.410477 |
| PLA2R1 | 210194_at | NM_007366 | 22925 | Hs.410477 |
| PLAC1 | 219702_at | NM_021796.3 | 10761 | Hs.496811 |
| PLAC1L | 1557085_at | NM_173801.3 | 219990 | Hs.132310 |
| PLAC2 | 229385_s_at | NM_153375 | 257000 | Hs.515575 |
| PLAC2 | 235994_s_at | NM_153375 | 257000 | Hs.107203 |
| PLAC2 | 244374_at | NM_153375.1 | 257000 | Hs.515575 |
| PLAC9 | 227419_x_at | NM_001012973.1 | 219348 | Hs.204947 |
| PLAC9 | 230246_at | AI275020 | 219348 | Hs.361171 |
| PLAT | 201860_s_at | NM_000931.2 | 5327 | Hs.491582 |
| PLAU | 205479_s_at | NM_002658.2 | 5328 | Hs.77274 |
| PLAU | 211668_s_at | NM_002658.2 | 5328 | Hs.77274 |
| PLAUR | 214866_at | NM_001005376.1 | 5329 | Hs.466871 |
| PLAUR | 210845_s_at | NM_001005377.1 | 5329 | Hs.466871 |
| PLAUR | 211924_s_at | NM_001005377.1 | 5329 | Hs.466871 |
| PLB1 | 235703_at | NM_153021.3 | 151056 | Hs.387498 |
| PLCG1 | 216551_x_at | NM_182811.1 | 5335 | Hs.268177 |
| PLCG1 | 202789_at | NM_002660.2 | 5335 | Hs.268177 |
| PLCG2 | 1563263_at | NM_002661 | 5336 | Hs.271620 |
| PLCG2 | 204613_at | NM_002661.1 | 5336 | Hs.413111 |
| PLD4 | 235802_at | NM_138790.2 | 122618 | Hs.407101 |
| PLD5 | 1563933_a_at | AK091691.1 | 200150 | Hs.498252 |
| PLEKHB1 | 229046_s_at | NM_021200 | 58473 | Hs.445489 |
| PLEKHB1 | 229047_at | NM_021200 | 58473 | Hs.445489 |
| PLEKHB1 | 209504_s_at | NM_021200.1 | 58473 | Hs.445489 |
| PLG | 230931_at | NM_000301 | 5340 | Hs.143436 |
| PLG | 240033_at | NM_000301 | 5340 | Hs.143436 |
| PLG | 209977_at | NM_000301.1 | 5340 | Hs.558366 |
| PLG | 209978_s_at | NM_000301.1 | 5340 | Hs.558366 |
| PLGLA1 | 230120_s_at | XM_934130.1 | 285189 | Hs.449164 |
| PLGLB1 | 222249_at | AB051438 | 5343 | 424184 |
| PLGLB1 | 205871_at | NM_001032392.1 | 5343 | Hs.450026 |
| PLLP | 217265_at | AL020989 | 51090 | 632215 |
| PLLP | 204519_s_at | NM_015993.1 | 51090 | Hs.200821 |
| PLP1 | 210198_s_at | NM_000533.3 | 5354 | Hs.1787 |
| PLP2 | 201136_at | NM_002668.1 | 5355 | Hs.77422 |
| PLSCR1 | 241916_at | NM_021105 | 5359 | Hs.348478 |
| PLSCR1 | 244315_at | NM_021105 | 5359 | Hs.348478 |
| PLSCR1 | 202430_s_at | NM_021105.1 | 5359 | Hs.130759 |
| PLSCR1 | 202446_s_at | NM_021105.1 | 5359 | Hs.130759 |
| PLSCR2 | 207374_at | NM_020359.1 | 57047 | Hs.147305 |
| PLSCR3 | 218828_at | NM_020360.2 | 57048 | Hs.433154 |
| PLSCR3 | 56197_at | NM_020360.2 | 57048 | Hs.433154 |
| PLSCR4 | 218901_at | NM_020353.1 | 57088 | Hs.477869 |
| PLTP | 202075_s_at | NM_182676.1 | 5360 | Hs.439312 |
| PLUNC | 220542_s_at | NM_016583.2 | 51297 | Hs.211092 |
| PLVAP | 221529_s_at | NM_031310.1 | 83483 | Hs.107125 |
| PLXDC1 | 214081_at | NM_020405.3 | 57125 | Hs.125036 |
| PLXDC1 | 219700_at | NM_020405.3 | 57125 | Hs.125036 |
| PLXDC2 | 227276_at | NM_032812.7 | 84898 | Hs.498939 |
| PLXNA1 | 221537_at | NM_032242.2 | 5361 | Hs.432329 |
| PLXNA1 | 221538_s_at | NM_032242.2 | 5361 | Hs.432329 |
| PLXNA1 | 1558140_at | NM_032242.2 | 5361 | Hs.432329 |
| PLXNA2 | 227032_at | NM_153014 | 5362 | Hs.350065 |
| PLXNA2 | 207290_at | NM_025179 | 5362 | Hs.350065 |
| PLXNA2 | 213030_s_at | NM_025179.2 | 5362 | Hs.350065 |
| PLXNA3 | 1553139_s_at | NM_017514.2 | 55558 | Hs.21432 |
| PLXNA3 | 203623_at | NM_017514.2 | 55558 | Hs.21432 |
| PLXNA4 | 1562240_at | AB046770 | 57671 | Hs.169129 |
| PLXNA4A | 228104_at | AB046770.3 | 57671 | Hs.511454 |
| PLXNA4A | 232317_at | AB046770.3 | 57671 | Hs.511454 |
| PLXNB1 | 215668_s_at | NM_002673.3 | 5364 | Hs.476209 |
| PLXNB1 | 215807_s_at | NM_002673.3 | 5364 | Hs.476209 |
| PLXNB2 | 208890_s_at | NM_012401 | 23654 | Hs.3989 |
| PLXNB2 | 211472_at | NM_012401 | 23654 | Hs.3989 |
| PLXNB3 | 205957_at | NM_005393.1 | 5365 | Hs.380742 |
| PLXNC1 | 213241_at | NM_005761 | 10154 | Hs.554795 |
| PLXNC1 | 235328_at | NM_005761 | 10154 | Hs.286229 |
| PLXNC1 | 206470_at | NM_005761.1 | 10154 | Hs.554795 |
| PLXNC1 | 206471_s_at | NM_005761.1 | 10154 | Hs.554795 |
| PLXND1 | 38671_at | NM_015103.1 | 23129 | Hs.301685 |
| PLXND1 | 1563657_at | NM_015103 | 23129 | Hs.301685 |
| PLXND1 | 212235_at | NM_015103.1 | 23129 | Hs.301685 |
| PM20D1 | 239929_at | NM_152491.3 | 148811 | Hs.177744 |
| PMCH | 206942_s_at | NM_002674.2 | 5367 | Hs.2182 |
| PMCHL1 | 217123_x_at | NM_031887.2 | 5369 | Hs.247975 |
| PMCHL1 | 224418_x_at | NM_031887.2 | 5369 | Hs.247975 |
| PMCHL1 | 224419_x_at | NM_031887.2 | 5369 | Hs.247975 |
| PMCHL1 | 224421_x_at | NM_031887.2 | 5369 | Hs.247975 |
| PMP2 | 235127_at | NM_002677.3 | 5375 | Hs.2868 |
| PMP2 | 206826_at | NM_002677.3 | 5375 | Hs.2868 |
| PMP22 | 210139_s_at | NM_153322.1 | 5376 | Hs.372031 |
| PNLIP | 205912_at | NM_000936.2 | 5406 | Hs.501135 |
| PNLIPRP1 | 206694_at | NM_006229.1 | 5407 | Hs.73923 |
| PNLIPRP1 | 210722_at | NM_006229 | 5407 | Hs.73923 |
| PNLIPRP2 | 217343_at | AL034369 | 5408 | 423598 |
| PNLIPRP2 | 211766_s_at | NM_005396 | 5408 | Hs.423598 |
| PNLIPRP3 | 1558846_at | NM_001011709.1 | 119548 | Hs.276724 |
| PNOC | 205901_at | NM_006228.3 | 5368 | Hs.88218 |
| PNPLA2 | 39854_r_at | NM_020376.2 | 57104 | Hs.118463 |
| PNPLA2 | 212705_x_at | NM_020376.2 | 57104 | Hs.118463 |
| PODN | 226522_at | NM_153703.3 | 127435 | Hs.136664 |
| PODN | 233148_at | AL122104.1 | 127435 | Hs.136664 |
| PODNL1 | 220411_x_at | NM_024825.2 | 79883 | Hs.448497 |
| PODXL | 201578_at | NM_001018111.1 | 5420 | Hs.16426 |
| PODXL2 | 219152_at | NM_015720.1 | 50512 | Hs.145416 |
| POLR2J2 | 217529_at | NM_032958 | 246721 | Hs.696339 |
| POMC | 205720_at | NM_001035256.1 | 5443 | Hs.1897 |
| PON1 | 206344_at | NM_000446.3 | 5444 | Hs.370995 |
| PON1 | 206345_s_at | NM_000446.3 | 5444 | Hs.370995 |
| PON2 | 236795_at | NM_000305 | 5445 | Hs.165598 |
| PON2 | 242700_at | NM_000305 | 5445 | Hs.165598 |
| PON2 | 201876_at | NM_000305.2 | 5445 | Hs.530077 |
| PON2 | 210830_s_at | NM_000305.2 | 5445 | Hs.530077 |
| PON3 | 213695_at | NM_000940.2 | 5446 | Hs.440967 |
| POPDC2 | 219647_at | NM_022135.2 | 64091 | Hs.16297 |
| POPDC3 | 219926_at | NM_022361.3 | 64208 | Hs.458336 |
| PORIMIN | 211967_at | NM_052932 | 114908 | Hs.503709 |
| POSTN | 1555777_at | NM_006475 | 10631 | Hs.136348 |
| POSTN | 1555778_a_at | NM_006475.1 | 10631 | Hs.136348 |
| POSTN | 210809_s_at | NM_006475.1 | 10631 | Hs.136348 |
| PPAP2A | 209147_s_at | NM_003711.2 | 8611 | Hs.435122 |
| PPAP2A | 210946_at | NM_176895.1 | 8611 | Hs.435122 |
| PPAP2B | 209355_s_at | NM_003713.3 | 8613 | Hs.405156 |
| PPAP2B | 212226_s_at | NM_003713.3 | 8613 | Hs.405156 |
| PPAP2B | 212230_at | NM_003713.3 | 8613 | Hs.405156 |
| PPAPDC1A | 236044_at | NM_001030059.1 | 196051 | Hs.40479 |
| PPAPDC1B | 223568_s_at | NM_032483.2 | 84513 | Hs.437179 |
| PPAPDC1B | 223569_at | NM_032483.2 | 84513 | Hs.437179 |
| PPAPDC1B | 226384_at | BC033025.1 | 84513 | Hs.437179 |
| PPAPDC2 | 227385_at | NM_203453.2 | 403313 | Hs.107510 |
| PPAPDC3 | 224506_s_at | NM_032728.2 | 84814 | Hs.134292 |
| PPBP | 214146_s_at | NM_002704.2 | 5473 | Hs.2164 |
| PPBPL2 | 208346_at | XM_926381.1 | 10895 | Hs.3134 |
| PPIA | 217346_at | NM_001008741 | 128192 | 647216 |
| PPIA | 217602_at | NM_021130 | 5478 | Hs.356331 |
| PPIA | 226336_at | NM_021130 | 5478 | Hs.356331 |
| PPIA | 235741_at | NM_021130 | 5478 | Hs.381244 |
| PPIA | 201293_x_at | NM_021130.2 | 5478 | Hs.356331 |
| PPIA | 211378_x_at | NM_021130.2 | 5478 | Hs.356331 |
| PPIA | 211765_x_at | NM_021130.2 | 5478 | Hs.356331 |
| PPIA | 211978_x_at | NM_021130.2 | 5478 | Hs.356331 |
| PPIA | 212661_x_at | NM_001008741.1 | 388817 | Hs.517207 |
| PPL | 203407_at | NM_002705.3 | 5493 | Hs.192233 |
| PPP1R3A | 206895_at | NM_002711.2 | 5506 | Hs.458309 |
| PPP1R3A | 211169_s_at | NM_002711.2 | 5506 | Hs.458309 |
| PPT1 | 200975_at | NM_000310.2 | 5538 | Hs.3873 |
| PPY | 210670_at | NM_002722.3 | 5539 | Hs.558368 |
| PPYR1 | 210956_at | NM_005972.3 | 5540 | Hs.524719 |
| PQLC1 | 218208_at | AK026031.1 | 80148 | Hs.288284 |
| PQLC2 | 220453_at | AK000327.1 | 54896 | Hs.523036 |
| PQLC2 | 1555781_at | NM_001040125.1 | 54896 | Hs.523036 |
| PQLC2 | 1555783_x_at | NM_001040126.1 | 54896 | Hs.523036 |
| PQLC3 | 225579_at | NM_152391.3 | 130814 | Hs.274415 |
| PRAF2 | 203456_at | NM_007213.1 | 11230 | Hs.29595 |
| PRAP1 | 243669_s_at | NM_145202.3 | 118471 | Hs.15951 |
| PRB3 | 206998_x_at | NM_006249.3 | 5544 | Hs.73031 |
| PRB4 | 216881_x_at | NM_002723.3 | 5545 | Hs.528651 |
| PREI3 | 202918_s_at | NM_015387.2 | 25843 | Hs.205173 |
| PREI3 | 202919_at | NM_199482.1 | 25843 | Hs.205173 |
| PRELP | 228224_at | NM_002725.3 | 5549 | Hs.76494 |
| PRELP | 37022_at | NM_002725.3 | 5549 | Hs.76494 |
| PRELP | 204223_at | NM_002725.3 | 5549 | Hs.76494 |
| PRF1 | 214617_at | NM_005041.3 | 5551 | Hs.2200 |
| PRF1 | 1553681_a_at | NM_005041.3 | 5551 | Hs.2200 |
| PRG1 | 1554676_at | NM_002727 | 5552 | Hs.1908 |
| PRG1 | 201858_s_at | NM_002727.2 | 5552 | Hs.1908 |
| PRG1 | 201859_at | NM_002727.2 | 5552 | Hs.1908 |
| PRG2 | 220798_x_at | NM_024888.1 | 79948 | Hs.546439 |
| PRG2 | 211743_s_at | NM_002728.4 | 5553 | Hs.512633 |
| PRH2 | 205272_s_at | NM_005042.2 | 5555 | Hs.73952 |
| PRIMA1 | 230087_at | NM_178013.2 | 145270 | Hs.432401 |
| PRKCA | 215194_at | NM_002737 | 5578 | Hs.279856 |
| PRKCA | 215195_at | NM_002737.2 | 5578 | Hs.531704 |
| PRKCA | 1560074_at | NM_002737.2 | 5578 | Hs.531704 |
| PRKCA | 206923_at | NM_002737.2 | 5578 | Hs.531704 |
| PRKCA | 213093_at | NM_002737.2 | 5578 | Hs.531704 |
| PRKCABP | 204746_s_at | NM_001039583.1 | 9463 | Hs.180871 |
| PRKD1 | 205880_at | NM_002742.1 | 5587 | Hs.508999 |
| PRL | 205445_at | NM_000948.2 | 5617 | Hs.1905 |
| PRLH | 221443_x_at | NM_015893.1 | 51052 | Hs.247710 |
| PRLR | 216638_s_at | NM_000949.2 | 5618 | Hs.368587 |
| PRLR | 227629_at | NM_000949 | 5618 | Hs.212892 |
| PRLR | 231981_at | NM_000949 | 5618 | Hs.212892 |
| PRLR | 243755_at | NM_000949.2 | 5618 | Hs.368587 |
| PRLR | 206346_at | NM_000949.2 | 5618 | Hs.368587 |
| PRLR | 210476_s_at | NM_000949.2 | 5618 | Hs.368587 |
| PRLR | 211917_s_at | NM_000949.2 | 5618 | Hs.368587 |
| PRND | 222106_at | NM_012409.2 | 23627 | Hs.406696 |
| PRND | 223813_at | NM_012409.2 | 23627 | Hs.406696 |
| PRNT | 233975_at | NM_177549.2 | 149830 | Hs.126516 |
| PROC | 206259_at | NM_000312 | 5624 | Hs.224698 |
| PROCR | 203650_at | NM_006404.3 | 10544 | Hs.82353 |
| PROK1 | 229124_at | NM_032414.2 | 84432 | Hs.514793 |
| PROK2 | 232629_at | NM_021935 | 60675 | Hs.203181 |
| PROL1 | 208004_at | NM_021225.3 | 58503 | Hs.479905 |
| PROM1 | 204304_s_at | NM_006017.1 | 8842 | Hs.479220 |
| PROM2 | 239528_at | NM_144707 | 150696 | Hs.145582 |
| PROM2 | 1552797_s_at | NM_144707.1 | 150696 | Hs.469313 |
| PROM2 | 1562378_s_at | NM_144707.1 | 150696 | Hs.469313 |
| PROS1 | 207808_s_at | NM_000313.1 | 5627 | Hs.64016 |
| PROZ | 208034_s_at | NM_003891.1 | 8858 | Hs.1011 |
| PRR4 | 204919_at | NM_007244.1 | 11272 | Hs.408153 |
| PRR7 | 219742_at | NM_030567.2 | 80758 | Hs.534492 |
| PRRG1 | 1566779_at | AL359566 | 5638 | 190341 |
| PRRG1 | 1566780_at | AL359566 | 5638 | 190341 |
| PRRG1 | 205618_at | NM_000950.1 | 5638 | Hs.190341 |
| PRRG2 | 205617_at | NM_000951.1 | 5639 | Hs.35101 |
| PRRG3 | 220433_at | NM_024082.2 | 79057 | Hs.209253 |
| PRRT1 | 229890_at | NM_030651.3 | 80863 | Hs.549204 |
| PRRT1 | 231739_at | NM_030651.3 | 80863 | Hs.549204 |
| PRRT2 | 227192_at | NM_145239.1 | 112476 | Hs.556007 |
| PRSS12 | 205515_at | NM_003619.2 | 8492 | Hs.445857 |
| PRSS2 | 216470_x_at | NM_002770.2 | 5645 | Hs.367767 |
| PRSS2 | 205402_x_at | NM_002770.2 | 5645 | Hs.367767 |
| PRSS21 | 220051_at | NM_006799.2 | 10942 | Hs.72026 |
| PRSS22 | 205847_at | NM_022119.3 | 64063 | Hs.459709 |
| PRSS23 | 226279_at | NM_007173.3 | 11098 | Hs.25338 |
| PRSS23 | 202458_at | NM_007173.3 | 11098 | Hs.25338 |
| PRSS27 | 232074_at | NM_031948.3 | 83886 | Hs.332878 |
| PRSS3 | 213421_x_at | NM_002771.2 | 5646 | Hs.435699 |
| PRSS3 | 207463_x_at | NM_002771.2 | 5646 | Hs.435699 |
| PRSS33 | 1552348_at | NM_152891.1 | 260429 | Hs.280658 |
| PRSS33 | 1552349_a_at | NM_152891.1 | 260429 | Hs.280658 |
| PRSS35 | 235874_at | NM_153362.1 | 167681 | Hs.98381 |
| PRSS36 | 1552555_at | NM_173502.2 | 146547 | Hs.256632 |
| PRSS7 | 217269_s_at | NM_002772.1 | 5651 | Hs.149473 |
| PRSS7 | 207638_at | NM_002772.1 | 5651 | Hs.149473 |
| PRSS8 | 202525_at | NM_002773.2 | 5652 | Hs.75799 |
| PRTG | 1562590_at | NM_173814.3 | 283659 | Hs.130957 |
| PRX | 220024_s_at | NM_020956.1 | 57716 | Hs.205457 |
| PRX | 222255_at | NM_020956 | 57716 | Hs.205457 |
| PSCA | 1560011_at | NM_005672 | 8000 | Hs.379010 |
| PSCA | 205319_at | NM_005672 | 8000 | Hs.379010 |
| PSD2 | 223536_at | NM_032289.1 | 84249 | Hs.21963 |
| PSEN1 | 226577_at | NM_000021 | 5663 | Hs.3260 |
| PSEN1 | 238816_at | NM_000021 | 5663 | Hs.3260 |
| PSEN1 | 1559206_at | NM_000021 | 5663 | Hs.3260 |
| PSEN1 | 1567440_at | NM_000021 | 5663 | Hs.3260 |
| PSEN1 | 1567443_x_at | NM_000021 | 5663 | Hs.3260 |
| PSEN1 | 203460_s_at | NM_000021.2 | 5663 | Hs.3260 |
| PSEN1 | 207782_s_at | NM_000021.2 | 5663 | Hs.3260 |
| PSEN2 | 204261_s_at | NM_000447.1 | 5664 | Hs.25363 |
| PSEN2 | 204262_s_at | NM_000447.1 | 5664 | Hs.25363 |
| PSEN2 | 211373_s_at | NM_000447.1 | 5664 | Hs.25363 |
| PSENEN | 218302_at | NM_172341.1 | 55851 | Hs.529100 |
| PSG1 | 208257_x_at | NM_006905 | 5669 | Hs.466848 |
| PSG1 | 210195_s_at | NM_006905.2 | 5669 | Hs.466848 |
| PSG1 | 210196_s_at | NM_006905.2 | 5669 | Hs.466848 |
| PSG11 | 206570_s_at | M69245.1 | 5680 | Hs.502097 |
| PSG2 | 208134_x_at | NM_031246.1 | 5670 | Hs.466854 |
| PSG3 | 215821_x_at | NM_021016 | 5671 | Hs.555887 |
| PSG3 | 203399_x_at | NM_021016 | 5671 | Hs.555887 |
| PSG3 | 211741_x_at | NM_021016 | 5671 | Hs.555887 |
| PSG4 | 208191_x_at | NM_002780.3 | 5672 | Hs.466848 |
| PSG6 | 239910_at | NM_002782 | 5675 | Hs.386788 |
| PSG6 | 208106_x_at | NM_002782.3 | 5675 | Hs.466849 |
| PSG6 | 209738_x_at | NM_001031850.1 | 5675 | Hs.466849 |
| PSG7 | 205602_x_at | NM_002783.1 | 5676 | Hs.512647 |
| PSG9 | 237372_at | N30169 | 5678 | 502092 |
| PSG9 | 207733_x_at | NM_002784.2 | 5678 | Hs.502092 |
| PSG9 | 209594_x_at | NM_002784.2 | 5678 | Hs.502092 |
| PSG9 | 210126_at | NM_002784 | 5678 | Hs.502092 |
| PSORS1C2 | 220635_at | NM_014069.1 | 170680 | Hs.146824 |
| PSPN | 221373_x_at | NM_004158.2 | 5623 | Hs.248159 |
| PSPN | 1564240_at | AK055490.1 | 5623 | Hs.248159 |
| PTAFR | 206278_at | NM_000952.3 | 5724 | Hs.46 |
| PTAFR | 211661_x_at | NM_000952.3 | 5724 | Hs.46 |
| PTCH | 1555520_at | BC043542.1 | 5727 | Hs.494538 |
| PTCH | 208522_s_at | NM_000264.2 | 5727 | Hs.494538 |
| PTCH | 209815_at | AK124593.1 | 5727 | Hs.494538 |
| PTCH2 | 221292_at | NM_003738.3 | 8643 | Hs.249164 |
| PTCHD1 | 1552848_a_at | NM_173495.1 | 139411 | Hs.319503 |
| PTCHD2 | 230019_s_at | XM_938154.1 | 57540 | Hs.202355 |
| PTCHD2 | 234355_s_at | XM_052561.8 | 57540 | Hs.202355 |
| PTCRA | 215492_x_at | NM_138296 | 171558 | Hs.169002 |
| PTCRA | 211252_x_at | NM_138296 | 171558 | Hs.169002 |
| PTCRA | 211837_s_at | NM_138296 | 171558 | Hs.169002 |
| PTDSS1 | 201433_s_at | NM_014754.1 | 9791 | Hs.292579 |
| PTDSS2 | 221005_s_at | NM_030783.1 | 81490 | Hs.12851 |
| PTGDR | 215894_at | NM_000953.2 | 5729 | Hs.306831 |
| PTGDR | 234165_at | NM_000953.2 | 5729 | Hs.306831 |
| PTGDS | 211663_x_at | NM_000954 | 5730 | Hs.558373 |
| PTGDS | 211748_x_at | NM_000954.5 | 5730 | Hs.558373 |
| PTGDS | 212187_x_at | NM_000954.5 | 5730 | Hs.558373 |
| PTGER1 | 214391_x_at | NM_000955.2 | 5731 | Hs.159360 |
| PTGER1 | 231201_at | NM_000955 | 5731 | Hs.159360 |
| PTGER1 | 207650_x_at | NM_000955.2 | 5731 | Hs.159360 |
| PTGER2 | 206631_at | NM_000956.2 | 5732 | Hs.2090 |
| PTGER3 | 213933_at | NM_198715.2 | 5733 | Hs.445000 |
| PTGER3 | 231030_at | NM_000957 | 5733 | Hs.27860 |
| PTGER3 | 208169_s_at | NM_198712.2 | 5733 | Hs.445000 |
| PTGER3 | 210374_x_at | NM_198715.2 | 5733 | Hs.445000 |
| PTGER3 | 210375_at | NM_198719.1 | 5733 | Hs.445000 |
| PTGER3 | 210831_s_at | NM_000957.2 | 5733 | Hs.445000 |
| PTGER3 | 210832_x_at | NM_198714.1 | 5733 | Hs.445000 |
| PTGER3 | 210833_at | NM_198715.2 | 5733 | Hs.445000 |
| PTGER3 | 210834_s_at | NM_198715.2 | 5733 | Hs.445000 |
| PTGER3 | 211265_at | NM_198720.1 | 5733 | Hs.445000 |
| PTGER3 | 211909_x_at | NM_198714.1 | 5733 | Hs.445000 |
| PTGER4 | 204896_s_at | NM_000958.2 | 5734 | Hs.199248 |
| PTGER4 | 204897_at | NM_000958.2 | 5734 | Hs.199248 |
| PTGES | 207388_s_at | NM_004878.3 | 9536 | Hs.146688 |
| PTGES | 210367_s_at | NM_004878.3 | 9536 | Hs.146688 |
| PTGFR | 1555097_a_at | NM_000959.3 | 5737 | Hs.292843 |
| PTGFR | 207177_at | NM_000959.3 | 5737 | Hs.292843 |
| PTGFRN | 224937_at | NM_020440.2 | 5738 | Hs.418093 |
| PTGFRN | 224950_at | NM_020440.2 | 5738 | Hs.418093 |
| PTGIR | 1555620_a_at | NM_000960.3 | 5739 | Hs.458324 |
| PTGIR | 206187_at | NM_000960.3 | 5739 | Hs.458324 |
| PTGS1 | 215813_s_at | NM_000962.2 | 5742 | Hs.201978 |
| PTGS1 | 238669_at | NM_000962.2 | 5742 | Hs.201978 |
| PTGS1 | 205127_at | NM_080591.1 | 5742 | Hs.201978 |
| PTGS1 | 205128_x_at | NM_080591.1 | 5742 | Hs.201978 |
| PTH | 206977_at | NM_000315.2 | 5741 | Hs.37045 |
| PTHLH | 1556773_at | NM_002820 | 5744 | Hs.89626 |
| PTHLH | 206300_s_at | NM_002820.2 | 5744 | Hs.89626 |
| PTHLH | 210355_at | NM_198965.1 | 5744 | Hs.89626 |
| PTHLH | 211756_at | NM_198964.1 | 5744 | Hs.89626 |
| PTHR1 | 205911_at | NM_000316.2 | 5745 | Hs.1019 |
| PTHR2 | 206772_at | NM_005048.2 | 5746 | Hs.159499 |
| PTK7 | 1555324_at | BC046109.2 | 5754 | Hs.90572 |
| PTK7 | 207011_s_at | NM_152880.2 | 5754 | Hs.90572 |
| PTN | 208408_at | NM_002825 | 5764 | Hs.44 |
| PTN | 209465_x_at | NM_002825.5 | 5764 | Hs.371249 |
| PTN | 209466_x_at | NM_002825.5 | 5764 | Hs.371249 |
| PTN | 211737_x_at | NM_002825.5 | 5764 | Hs.371249 |
| PTPLA | 219654_at | NM_014241.3 | 9200 | Hs.114062 |
| PTPLA | 1554376_s_at | NM_014241 | 9200 | Hs.114062 |
| PTPLAD1 | 217777_s_at | NM_016395.1 | 51495 | Hs.512973 |
| PTPLAD1 | 222404_x_at | NM_016395.1 | 51495 | Hs.512973 |
| PTPLAD1 | 222405_at | AK074857.1 | 51495 | Hs.512973 |
| PTPLAD1 | 234000_s_at | NM_016395.1 | 51495 | Hs.512973 |
| PTPLAD2 | 244050_at | NM_001010915.1 | 401494 | Hs.136247 |
| PTPRA | 213795_s_at | NM_002836.2 | 5786 | Hs.269577 |
| PTPRA | 213799_s_at | NM_002836.2 | 5786 | Hs.269577 |
| PTPRA | 233313_at | NM_002836 | 5786 | Hs.306676 |
| PTPRA | 239419_at | NM_002836 | 5786 | Hs.306676 |
| PTPRB | 230250_at | BX648771.1 | 5787 | Hs.434375 |
| PTPRB | 244343_at | NM_002837 | 5787 | Hs.434375 |
| PTPRB | 1560105_at | NM_002837 | 5787 | Hs.434375 |
| PTPRB | 205846_at | NM_002837.2 | 5787 | Hs.434375 |
| PTPRC | 1552480_s_at | NM_080923.1 | 5788 | Hs.192039 |
| PTPRC | 207238_s_at | NM_002838.2 | 5788 | Hs.192039 |
| PTPRC | 212587_s_at | NM_002838.2 | 5788 | Hs.192039 |
| PTPRC | 212588_at | NM_080921.1 | 5788 | Hs.192039 |
| PTPRCAP | 204960_at | NM_005608.2 | 5790 | Hs.155975 |
| PTPRD | 213362_at | NM_002839 | 5789 | Hs.323079 |
| PTPRD | 214043_at | NM_002839 | 5789 | Hs.323079 |
| PTPRD | 1567859_at | NM_001040712 | 5789 | 446083 |
| PTPRD | 1567860_at | NM_001040712 | 5789 | 446083 |
| PTPRD | 205712_at | NM_002839 | 5789 | Hs.446083 |
| PTPRE | 221840_at | NM_006504.3 | 5791 | Hs.558374 |
| PTPRE | 1559018_at | NM_006504 | 5791 | Hs.558374 |
| PTPRE | 1566474_at | NM_006504 | 5791 | 127022 |
| PTPRE | 1566475_at | NM_006504 | 5791 | 127022 |
| PTPRF | 215066_at | NM_002840 | 5792 | Hs.272062 |
| PTPRF | 200635_s_at | NM_002840 | 5792 | Hs.272062 |
| PTPRF | 200636_s_at | NM_002840 | 5792 | Hs.272062 |
| PTPRF | 200637_s_at | NM_002840 | 5792 | Hs.272062 |
| PTPRG | 1569323_at | NM_002841 | 5793 | Hs.89627 |
| PTPRG | 204944_at | NM_002841 | 5793 | Hs.148340 |
| PTPRH | 208300_at | NM_002842.2 | 5794 | Hs.179770 |
| PTPRJ | 210173_at | NM_002843.2 | 5795 | Hs.318547 |
| PTPRK | 233609_at | NM_002844 | 5796 | Hs.354262 |
[truncated: 148,900 more chars]
